# Global burden, risk factors, and trends of non‐Hodgkin lymphoma: A worldwide analysis of cancer registries

**DOI:** 10.1002/cam4.7056

**Published:** 2024-03-13

**Authors:** Junjie Huang, Sze Chai Chan, Veeleah Lok, Lin Zhang, Don Eliseo Lucero‐Prisno, Wanghong Xu, Zhi‐Jie Zheng, Edmar Elcarte, Mellissa Withers, Martin C. S. Wong

**Affiliations:** ^1^ The Jockey Club School of Public Health and Primary Care, Faculty of Medicine Chinese University of Hong Kong Hong Kong SAR China; ^2^ Centre for Health Education and Health Promotion, Faculty of Medicine The Chinese University of Hong Kong Hong Kong SAR China; ^3^ Department of Global Public Health Karolinska Institute, Karolinska University Hospital Stockholm Sweden; ^4^ Suzhou Industrial Park Monash Research Institute of Science and Technology Suzhou China; ^5^ The School of Public Health and Preventive Medicine Monash University Melbourne Victoria Australia; ^6^ Department of Global Health and Development London School of Hygiene and Tropical Medicine London UK; ^7^ School of Public Health Fudan University Shanghai China; ^8^ Department of Global Health, School of Public Health Peking University Beijing China; ^9^ University of the Philippines Manila Philippines; ^10^ Department of Population and Health Sciences, Institute for Global Health University of Southern California Los Angeles California USA

**Keywords:** incidence, mortality, non‐Hodgkin lymphoma, risk factors, temporal trends

## Abstract

**Background:**

Non‐Hodgkin lymphoma (NHL) accounts for 90% of all malignant lymphomas. This study aimed to evaluate the global incidence, mortality, associated risk factors, and temporal trends of NHL by sex, age, and country.

**Methods:**

Data from 185 countries globally were used for analysis. NHL incidence and mortality were collected via the *GLOBOCAN* (2020), *CI5* series I‐X, *WHO mortality database*, the *Nordic Cancer Registries*, and the *SEER Program*. The *WHO Global Health Observatory* provided country‐level, age‐standardized prevalence of lifestyle and metabolic risk factors. Trends were examined and reported based on average annual percentage change (AAPC) calculated using Joinpoint regression analysis. Incidence and AAPC are based on data for the last 10 years across countries.

**Results:**

Globally, age‐standardized incidence and mortality rates for NHL were recorded at 5.8 and 2.6 per 100,000 individuals, respectively. At country‐level, NHL incidence was significantly associated with various factors, including HDI (Human Development Index), GDP per capita, prevalence of tobacco and alcohol consumption, sedentary lifestyle, obesity, hypertension, diabetes and hypercholesterolaemia. Rising trend in NHL incidence was observed, with the highest increase recorded in Estonia (AAPC_male_ = 4.15, AAPC_female_ = 5.14), Belarus (AAPC_female_ = 5.13), and Lithuania (AAPC_female_ = 4.68). While overall NHL mortality has been decreasing, certain populations experienced increased mortality over the decade. In Thailand, AAPC for mortality was 31.28% for males and 30.26% for females. Estonia saw an AAPC of 6.46% for males, while Slovakia experienced an AAPC of 4.24% for females. Colombia's AAPC was 1.29% for males and 1.51% for females.

**Conclusions:**

This study indicates a rising trend of NHL incidence over the past decade‐ particularly in developed countries, older males, and younger populations. Further research should investigate deeper insights into specific etiology and prognosis of NHL across subtypes, and potential contributors towards these epidemiologic trends.

## 1 INTRODUCTION

Non‐Hodgkin lymphoma (NHL) is a blood‐related malignancy originating from lymphocytes, and accounts for 90% of all malignant lymphomas.^1^ Main types of NHL include diffuse large B‐cell lymphoma and follicular lymphoma. In 2020, NHL represented approximately 2.8% of the total cancer burden, and contributed to 2.6% of all cancer‐related mortality.^2^ Western countries experience a relatively favorable 5‐year survival rate of approximately 70% for NHL, owing to chemotherapy, radiation, immunotherapy, and targeted therapy.^3^

Possible risk factors for NHL involved a range of demographics such as age, gender, race, ethnicity, geography, family history.^4^, ^5^ Individuals with weakened immune systems, autoimmune diseases, and certain infections are particularly at a higher risk of developing NHL.^4^, ^5^ Moreover, chemical and radiation exposure, whether environmental occupationally‐related, are recognized as risk factors for NHL.^4^, ^5^ Several studies further indicate being overweight or obese may potentially increase the risk of specific NHL subtypes.^6^, ^7^ However, it is worth mentioning that association between NHL and other common lifestyle and metabolic risk factors, such as smoking,^8^ alcohol intake,^8^ physical activity, and metabolic syndrome, have been established based on individual‐level data rather than country‐level data.^9^, ^10^, ^11^

Assessing latest global disease distribution, risk factors, and temporal trends of NHL are central to develop targeted preventive strategies addressing country‐specific challenges. Although prior studies have investigated epidemiological trends of NHL, they often relied on outdated data or narrowed focus on specific countries.^12^, ^13^, ^14^ These studies were likely to neglect examination of age‐based disease distribution, analysis of subtle age trends at country level, and exploration of associated lifestyle and metabolic risk factors.^15^, ^16^, ^17^

This study aims to tackle the existing knowledge gap by (1) analyzing global NHL incidence and mortality rates based on latest available data, focused on varied subgroups; (2) investigating relationships between common lifestyle and metabolic risk factors with the disease burden of NHL; and (3) examining temporal trends in NHL incidence and mortality by age, gender, and geographical location; (4) providing evidence that facilitates policy developments aimed at reducing burden of NHL in diverse geographical contexts.

## 2 METHODS

The GLOBOCAN database appraised an updated summary of key findings regarding NHL in 2020.^18^ The Human Development Index (HDI) was obtained from the United Nations, while the gross domestic products (GDP) per capita for each country was sourced from the World Bank.^19^ The Global Health Data Exchange (GHDx) serves as a comprehensive database of global health‐related information, aggregating data from various sources including surveys, registries, and research studies. For this study, data on risk factors including smoking prevalence, alcohol consumption, unhealthy dietary habits, physical inactivity, hypertension, diabetes, and hypercholesterolaemia were extracted from the GHDx. While GHDx data collection methods and quality may vary across countries, standardization techniques were adopted in this study to mitigate data heterogeneity and minimize impacts of data collection technologies.

The *Cancer Incidence in Five Continents* (*CI5*), volumes I‐XI comprised of reliable cancer registries on global, regional, and national levels, encompassing a sizable portion of the global population.^20^ NHL incidence data from CI5 was utilized for trend analysis, as it offers validated incidence data within a specified time range for each reported cancer case. For mortality trend analysis, the WHO mortality database was employed to collect data on cancer‐related deaths for various countries and regions.^21^ Cancer mortality data for this study was sourced from national civil cancer registries. These registries follow a process to clinically verify the mortalities associated with cancer and their causes prior to their annual data submissions to the World Health Organization (WHO). Data accuracy and comprehensiveness were ensured by rigorous standards applied in using medium to high quality figures. Cancer incidence and mortality data for the United States and Scandinavian region were retrieved from the *Surveillance, Epidemiology, and End Results* (*SEER*) Program and *Nordic Cancer Registries* (*NORDCAN*), respectively.^22^, ^23^

AAPC for cancer incidence and mortality were calculated using the CI5, SEER, WHO mortality database, and NORDCAN. While each database covers different time frames, all of them had a complete overlap in our selected range of 1980–2019. A comprehensive description of the sources used can be found in Table S1. Age‐Standardized Rate (ASR) is determined by taking the weighted average of age‐specific rates per 100,000 individuals, with the weights reflecting the age group distribution in a standard population. NHL incidence and mortality figures were standardized by age using the Segi‐Doll world reference population, thereby obtaining ASRs for various countries.^24^

### 2.1 Statistical analysis

Descriptive analysis provided information on NHL incidence and mortality rates, and two choropleth maps created to depict the global NHL incidence and mortality rates in 2020.

Epidemiological variations for NHL in this study were examined based on three categories: sex, age, and geographical regions. The categorization included gender (males and females), different age groups (≥50 years, <50 years, and < 40 years), and varied regions (Asia, Oceania, North America, South America, Northern Europe, Western Europe, Southern Europe, Eastern Europe, and Africa). Further ensuring the robustness of the analysis in this study, the age of 50 years was selected as the cutoff point for early cancer onset, aligning with common practice in cancer research.^25^ Age groups below 40 years were established as a sensitivity analysis in this study for additional reliability.

Joinpoint regression analysis, using the Joinpoint Regression Programme (Version 4.9.0.0), was utilized in identifying time‐based patterns of incidence and mortality within each sub‐group and country over the past decade.^26^ Incidence and mortality rates were logarithmically transformed, with corresponding standard errors estimated via binomial approximation. Weights were assigned to each segment based on their length within the selected timeframe. Joinpoint guidelines recommend a maximum of one joinpoint for studies comprising 7–11 data points, which was adopted in the analysis here.^27^ Subgroups categorized by age were subjected to geometric weighing, and AAPCs then estimated as the average of Annual Percent Changes (APCs) with their corresponding 95% CIs. Previous studies examining different cancer types, epidemiologic trends of cancer incidence and mortality were assessed using a similar approach.^28^, ^29^, ^30^, ^31^, ^32^

Univariable linear regression analysis was applied assess the associations between NHL burden and various factors (HDI, GDP per capita, the prevalence of smoking, alcohol drinking, unhealthy dietary habits, physical inactivity, hypertension, diabetes, plasma lipid levels). Beta coefficients (*β*) and corresponding 95% confidence intervals (95% CI) were estimated to quantify the degree of change in ASR for NHL incidence and mortality per unit increase in the respective risk factors. Threshold for statistical significance was set at *p*‐values less than 0.05 (*p* < 0.05).^33^

## 3 RESULTS

### 3.1 NHL in 2020: Global incidence

A total of 544,352 new cases of NHL and an ASR of incidence was 5.8 per 100,000 population in 2020. Males experienced a substantially higher incidence rate compared to females (ASR_male_ = 6.9, ASR_female_ = 4.8). Considerable regional disparities in NHL incidence were seen, an approximate five‐fold contrast among different regions, with notable differences between regions with highest and lowest rates. Australia and New Zealand (ASR = 12.5), North America (ASR = 12.0), and Northern Europe (ASR = 11.4) reported highest incidence rates, while Asia and Africa predominantly had lower rates. In particular, South‐Central Asia (ASR = 2.7), Middle Africa (ASR = 3.6), and Western Africa (ASR = 4.5) had the lowest. Furthermore, HDI was significantly associated with NHL incidence. Regions with very high HDI had a significantly higher incidence rate compared to those with relatively lower HDI levels (ASR_very high HDI_ = 9.3, ASR_high HDI_ = 4.8, ASR_medium HDI_ = 3.1, ASR_low HDI_ = 4.5).

### 3.2 NHL in 2020: Global mortality

**FIGURE 1:**
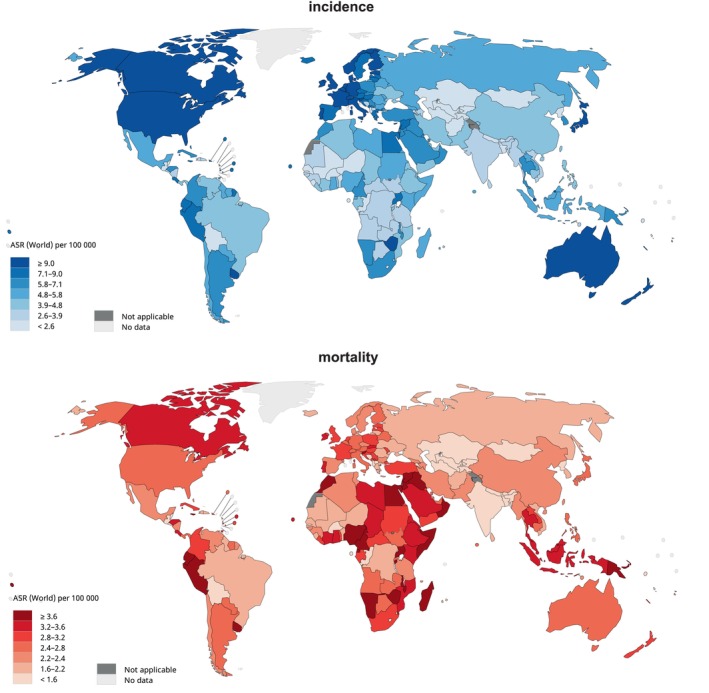
Global incidence and mortality of non‐Hodgkin lymphoma in 2020. Data source: GLOBOCAN 2020 Graph production: IARC (http://gco.iarc.fr/today) World Health Organization.

In 2020, 259,793 new NHL‐related deaths were reported on a global scale, with a mortality ASR of 2.6 per 100,000 people. There was a relatively small variation among geographical regions in terms of mortality compared to incidence. Highest mortality rates were reported in the African regions and the Pacific Islands, particularly Melanesia (ASR = 4.0), Northern Africa (ASR = 3.7), and Micronesia (ASR =3.6) the highest. Contrastingly, South‐Central Asia (ASR = 1.6), Central America (ASR = 2.2), and Central and Eastern Europe (ASR = 2.3) reported the lowest mortality rates. Regarding the association between HDI and mortality, countries with low HDI had a slightly higher ASR than compared to regions with other HDI levels (ASR_very high HDI_ = 2,7, ASR_high HDI_ = 2.6, ASR_medium HDI_ = 1.8, ASR_low HDI_ = 3.2). A visual representation of the global NHL incidence and mortality can be found in Figure 1; Figure S1.

### 3.3 Associations between NHL burden and risk factors

**FIGURE 2:**
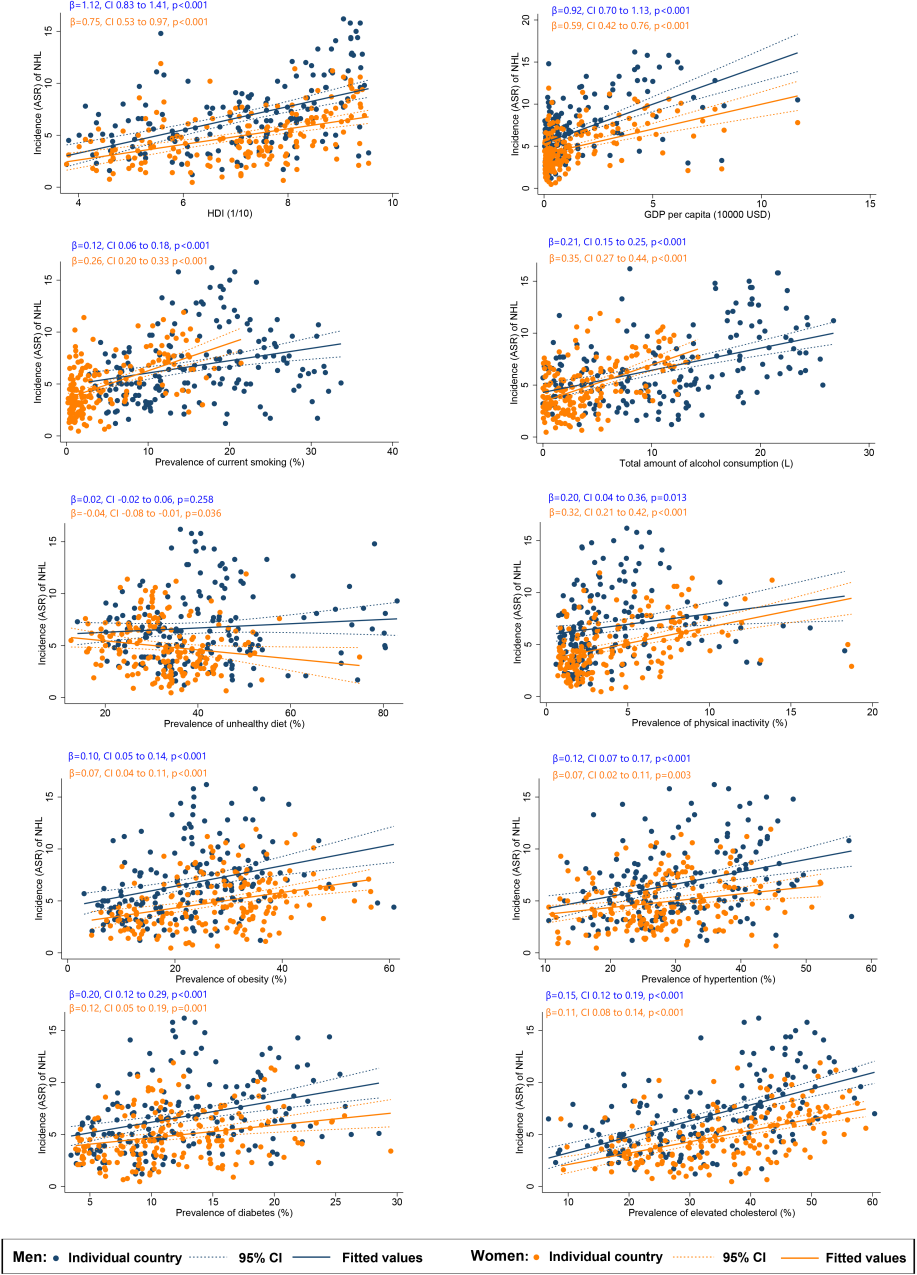
Associations between risk factors and incidence of non‐Hodgkin lymphoma.

**FIGURE 3:**
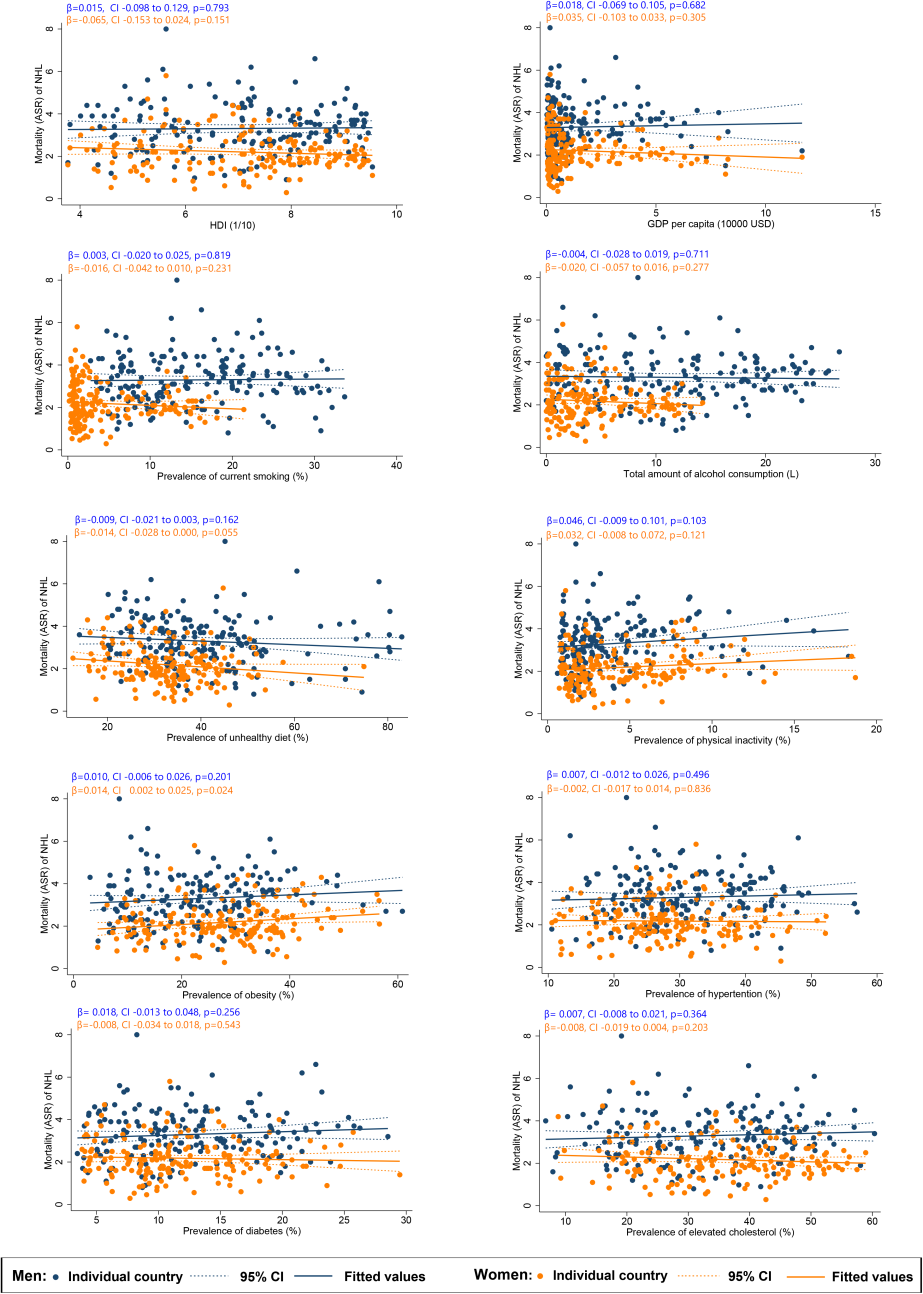
Associations between risk factors and mortality of non‐Hodgkin lymphoma.

Among males, a higher ASR of NHL was associated with a higher HDI (*β* = 1.12, CI 0.83 to 1.41, *p* < 0.001), GDP per capita (*β* = 0.92, CI 0.70 to 1.13, *p* < 0.001); and higher prevalence of smoking (*β* = 0.12, CI 0.06 to 0.18, *p* < 0.001), alcohol drinking (*β* = 0.21, CI 0.15 to 0.28, *p* < 0.001), physical inactivity (*β* = 0.20, CI 0.04 to 0.36, *p* = 0.013), obesity (*β* = 0.10, CI 0.05 to 0.14, *p* < 0.001), hypertension (*β* = 0.12, CI 0.07 to 0.17, *p* < 0.001), diabetes (*β* = 0.20, CI 0.12 to 0.29, *p* < 0.001), and hypercholesterolaemia (*β* = 0.15, CI 0.12 to 0.19, *p* < 0.001; Figure 2). However, no risk factors were identified at the country level associated to male NHL mortality (Figure 3).

For females, a higher NHL incidence of NHL was also associated with a higher HDI (*β* = 0.75, CI 0.53 to 0.97, *p* < 0.001), GDP per capita (*β* = 0.59, CI 0.42 to 0.76, *p* < 0.001); and higher prevalence of smoking (*β* = 0. 26, CI 0.20 to 0.33, *p* < 0.001), alcohol drinking (*β* = 0.35, CI 0.27 to 0.44, *p* < 0.001), physical inactivity (*β* = 0.32, CI 0.21–0.42, *p* < 0.001), obesity (*β* = 0.07, CI 0.04 to 0.11, *p* < 0.001), hypertension (*β* = 0.07, CI 0.02 to 0.11, *p* = 0.003), diabetes (*β* = 0.12, CI 0.05 to 0.19, *p* = 0.001), and hypercholesterolaemia (*β* = 0.11, CI 0.08 to 0.14, *p* < 0.001). However, among these risk factors, only the prevalence of obesity (*β* = 0.01, CI 0.001 to 0.025, *p* = 0.024) was associated with female NHL mortality at a country level.

### 3.4 Incidence trends in individuals aged 0–85+

Fifteen countries depicted significant increases in male NHL incidence, with 10 countries within the European continent. Most notable increases were found in Estonia (AAPC = 4.15, 95% CI = 0.49 to 7.94, *p* = 0.031), Ecuador (AAPC = 3.78, 95% CI = 0.76 to 6.88, *p* = 0.020), and Bulgaria (AAPC = 3.30, 95% CI = 0.89 to 5.77, *p* = 0.013). Conversely, five countries had significant decreases, with largest reductions seen in Cyprus (AAPC = −4.07, 95% CI = −6.60 to −1.48, *p* = 0.007), Colombia (AAPC = −3.19, 95% CI = −5.22 to −1.12, *p* = 0.008) and the Philippines (AAPC = −2.51, 95% CI = −4.76 to −0.21, *p* = 0.036).

**FIGURE 4:**
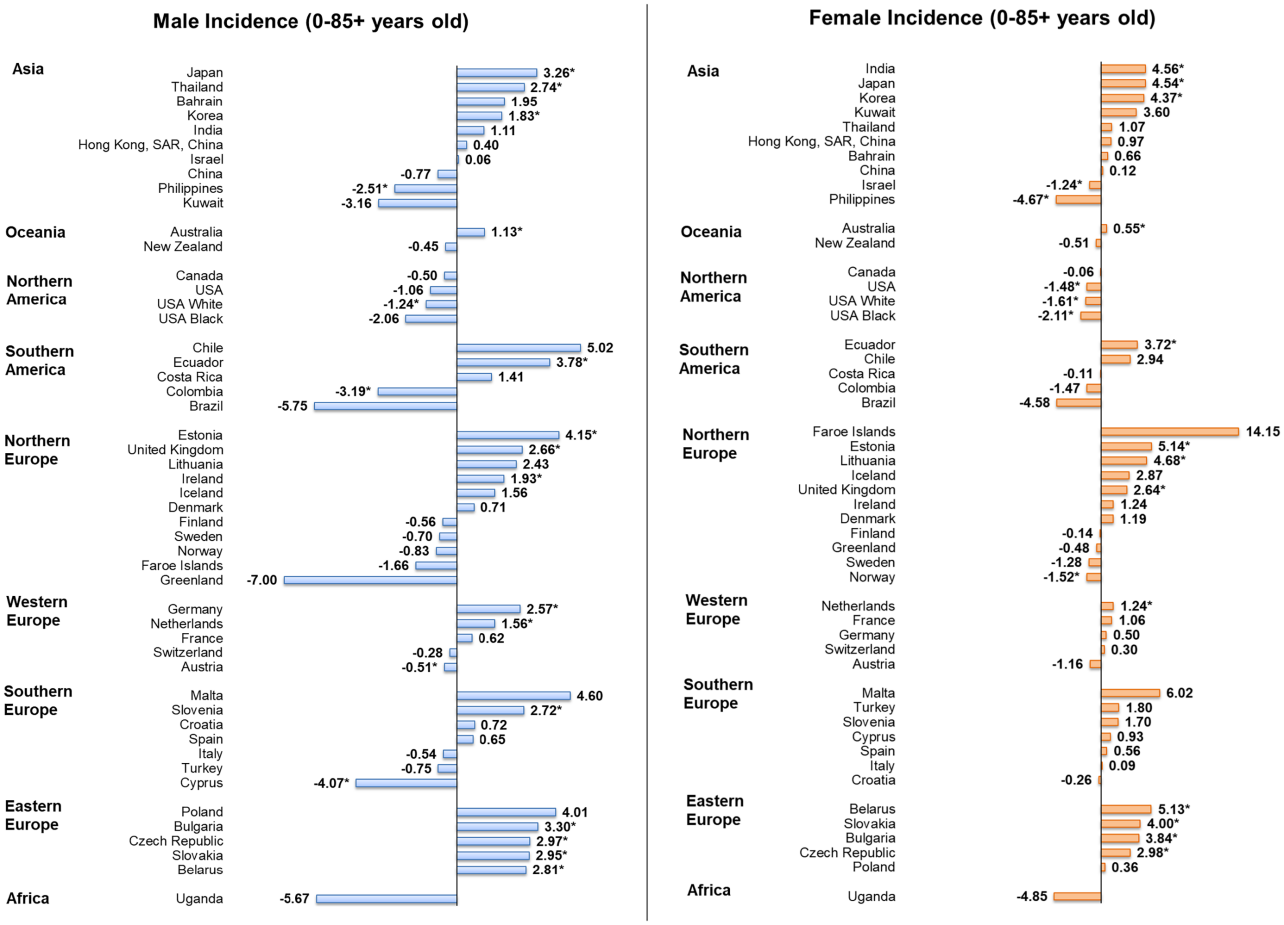
AAPC of incidence of non‐Hodgkin lymphoma of all ages. AAPC, annual percentage change. ^*^ *p* values less than 0.05.

Similar patterns were observed among females, with 13 countries exhibiting significant rises in incidence, 8 of which were European countries. The highest increases were reported in Estonia (AAPC = 5.14, 95% CI = 1.38 to 9.03, *p* = 0.013), Belarus (AAPC = 5.13, 95% CI 2.78 to 7.53, *p* = 0.001), and Lithuania (AAPC = 4.68, 95% CI = 1.85 to 7.59, *p* = 0.005). Decreases in incidence were observed in six populations, including the Philippines (AAPC = −4.67, 95% CI = −7.79 to −1.44, *p* = 0.011), as well as two populations in the United States (Black: AAPC = −2.11, 95% CI = −3.75 to −0.45, *p* = 0.019; White: AAPC = −1.61, 95% CI = −2.14 to −1.07, *p* < 0.001). Visualizations of the sex‐ and country‐specific AAPC trends can be referred to Figure 4. Please see Figure S2 for the global distribution of NHL incidence and mortality.

### 3.5 Mortality trends in individuals aged 0–85+

Sixteen countries, almost entirely in Northern Europe, reported significantly lowered mortality in males. Three countries leading highest mortality were Greenland (AAPC = −8.05, 95% CI = −14.89 to −0.66, *p* = 0.037), Norway (AAPC = −3.60, 95% CI = −6.23 to −0.89, *p* = 0.016), and Denmark (AAPC = −3.16, 95% CI −4.54 to −1.76, *p* = 0.001). In comparison, only three countries reported significant increases in male NHL mortality. Thailand (AAPC = 31.28, 95% CI = 6.40 to 61.98, *p* = 0.011) reported the most significant increase, followed by Estonia (AAPC = 6.46, 95% CI = 1.41 to 11.77, *p* = 0.018), and Colombia (1.29, 95% CI = 0.08 to 2.51, *p* = 0.039).

**FIGURE 5:**
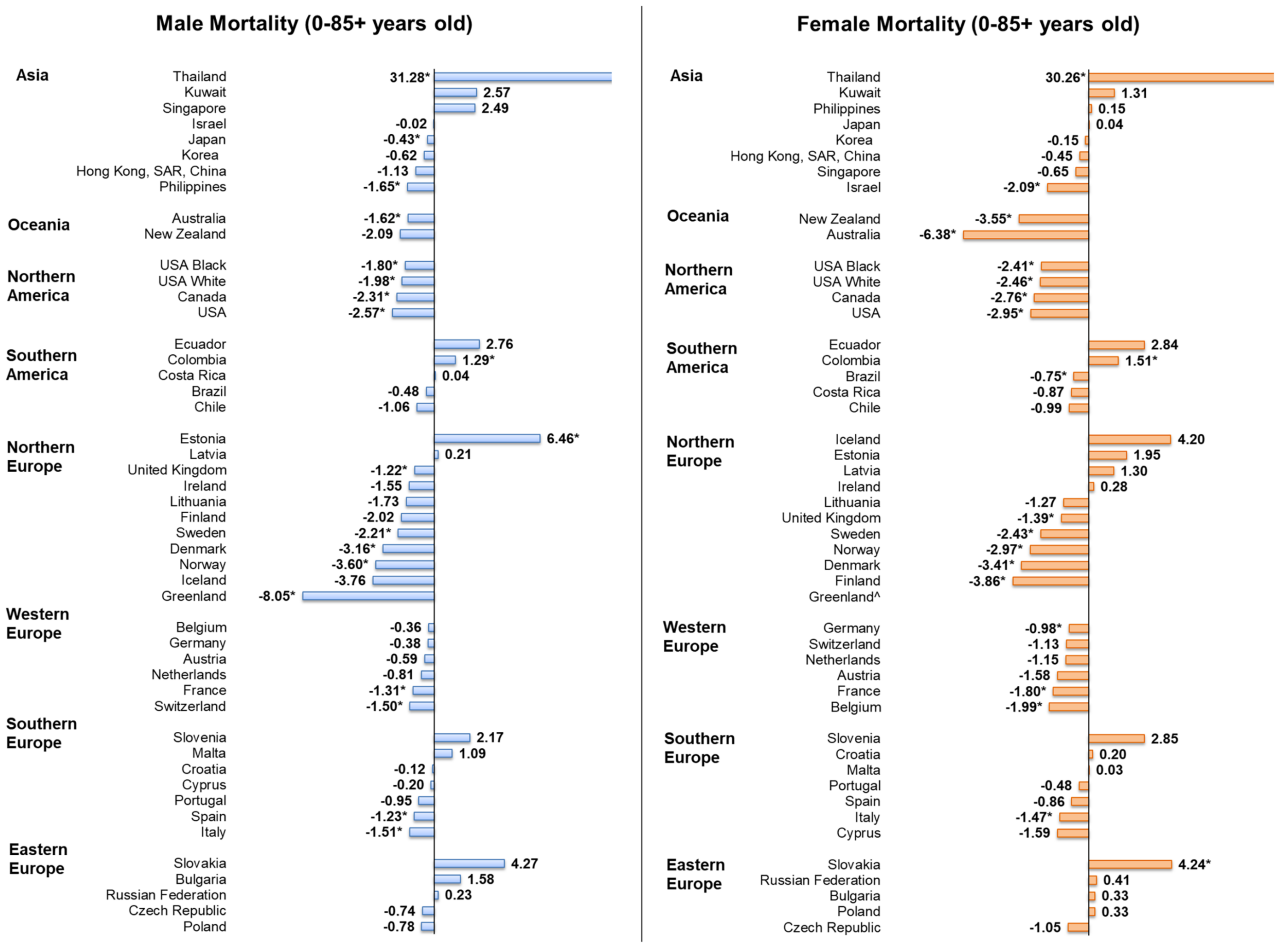
AAPC of mortality of non‐Hodgkin lymphoma of all ages. AAPC, annual percentage change. ^*^ *p* values less than 0.05.

Likewise, female mortality rates in 17 countries showed significant reduction. The greatest fall was observed in Australia (AAPC = −6.38, 95% CI = −7.39 to −5.36, *p* < 0.001), followed by Finland (AAPC = −3.86, 95% CI = −6.69 to −0.94, *p* = 0.010), and New Zealand (AAPC = −3.55, 95% CI = −6.71 to −0.29, *p* = 0.037). On the other hand, only three out of the 17 countries had significant female mortality: Thailand (AAPC = 30.26, 95% CI = 13.37 to 49.67, *p* = 0.002), Slovakia (AAPC = 4.24, 95% CI = 2.52 to 5.99, *p* < 0.001), and Colombia (AAPC = 1.51, 95% CI = 0.15 to 2.89, *p* = 0.034). For country‐specific mortality AAPC trends by sex please refer to Figure 5.

### 3.6 Incidence trends by specific age groups

For men aged 50 or above, a notable pattern emerged with 15 countries reporting significant increases in NHL incidence. Among them, Ecuador experienced the largest increase (AAPC = 5.04, 95% CI = 2.53 to 7.61, *p* = 0.002). In contrast, the US stood out as the only country with a significantly declining incidence (AAPC = −1.35, 95% CI −2.31 to −0.37, *p* = 0.013), with a particularly pronounced decline among the black population (AAPC = −2.30, 95% CI = −4.30 to −0.25, *p* = 0.033). Considering younger men below the age of 50, distinct patterns were observed. Increases in NHL incidence were identified in Thailand (AAPC = 5.44, 95% CI = 0.92 to 10.16, *p* = 0.024) and five other countries, while a decrease in incidence was found in Cyprus (AAPC = −10.15, 95% CI = −13.65 to −6.50, *p* < 0.001) and five other countries.

Trends considerably diverge for younger counterparts below age 40. Estonia (AAPC = 10.37, 95% CI = 1.76 to 19.70, *p* = 0.023) reported the largest increase in NHL incidence, while four countries reported declines. Cyprus (AAPC = −16.48, 95% CI = −24.28 to −7.89, *p* = 0.003) showed the greatest decline among the three countries reporting decreasing trends.

For females aged 50 or above, significant trends emerged wherein 14 countries experienced rising NHL incidence compared to only three countries in decline. Among the countries with increasing trends, Malta recorded the largest AAPC (AAPC = 6.66, 95% CI = 0.80 to 12.86, *p* = 0.030), while the Philippines displayed a significant decrease (AAPC = −5.18, 95% CI = −7.85 to −2.44, *p* = 0.003).

In the younger female cohort (< 50 years of age), an identical trend was observed. Malta demonstrated the highest increase of female NHL incidence (AAPC = 18.26, 95% CI = 0.54 to 39.09, *p* = 0.044), with five other countries also showing significant increases. Only Uganda reported a significant reduction (AAPC = −9.09, 95% CI = −13.04 to −4.96, *p* = 0.001).

Trends remained consistent when considering young females below age 40. Lithuania had the greatest increase (AAPC = 11.86, 95% CI = 3.36 to 21.07, *p* = 0.011), while Uganda reported the most significant decline (AAPC = −10.02, 95% CI = −13.99 to −5.87, *p* = 0.001). Please refer to Figures S3–S5 for NHL incidence and mortality by specific age groups.

## 4 DISCUSSION

### 4.1 Summary and explanations of major findings

A current, comprehensive assessment of the global NHL landscape was achieved by this study. Leveraging extensive cancer databases and registries, key insights were derived regarding NHL disease burden, risk factors, and epidemiological trends across diverse ages, genders, and regions. Several significant findings were discovered from the analysis: (1) The disease burden of NHL displayed substantial disparities, with high‐income countries exhibiting higher incidence rates, whereas lower‐income countries reporting higher mortality rates. (2) NHL incidence showed positive associations with several factors, namely higher HDI, GDP per capita, prevalence of smoking, alcohol drinking, physical inactivity, obesity, hypertension, diabetes, and hypercholesterolaemia; (3) The past decade has undergone a significant rise in NHL incidence trends at large, especially among older males, while NHL mortality rates have continued to decline.

The regional distribution of NHL in 2020 exhibited significant variations. Incidence of NHL was increased among countries possessing higher HDI and GDP per capita. These regions tend to have a higher prevalence of sedentary lifestyle and obesity,^34^, ^35^ which are known risk factors for NHL.^36^, ^37^ Additionally, availability and access regarding medical resources are crucial in ensuring early and accurate diagnoses for NHL.^38^ NHL incidence variations based on HDI and GDP per capita observed in this study may be influenced by differences in health system infrastructure, cancer service delivery, and the extent of diagnostic and treatment facilities for NHL.^38^ It is worth noting that the lower incidence of NHL in less developed countries could be partially attributed to under‐reporting or misclassification. On the other hand, the higher mortality observed in low‐income countries can be attributed to the prevalence of aggressive NHL. Research conducted in South Africa found that HIV infection was associated with a higher likelihood of aggressive NHL,^39^ suggesting that higher HIV infection rates in low‐income countries may contribute to an epidemic of aggressive NHL and subsequently higher mortality rates.^40^

Moreover, greater NHL mortality rates in low‐income countries could arise from medical resource inequalities. Previous studies have indicated health services in low‐income countries are limited by geographical accessibility and the availability of health services.^41^ This could result in NHL patients not having timely access to diagnosis and treatment, leading to delays in diagnosis due to a lack of access to medical services. However, another study found NHL patients with delayed diagnosis may develop more advanced disease and have an increased likelihood of toxic death from subsequent treatment.^42^

Correlations between behavioral and metabolic risk indicators of risk and occurrence of NHL at a population level were identified, encompassing factors such as smoking, alcohol intake, physical inactivity, obesity, hypertension, diabetes, and hypercholesterolemia. These findings align with previous studies that have examined individual‐level data, demonstrating consistent results. A case–control study revealed dose–response associations between increased risk of NHL and individuals smoking 15 or more cigarettes per day (Odds Ratio = 1.42, 95% CI = 1.02–1.97), when compared with never smokers.^43^ Another study conducted in Canada demonstrated that participants engaged in lifetime vigorous‐intensity physical activity had a reduced risk of NHL by approximately 25%–30%.^37^ Moreover, a meta‐analysis of 16 studies found overweight subjects had a relatively lower summary Risk Ratio (Risk Ratio = 1.07, 95% CI = 1.01–1.14) compared to obese subjects (Risk Ratio of 1.20, 95% CI = 1.07–1.34).^36^ However, a study from International InterLympg Consortium emphasized that there is no clear evidence supporting a significant relationship between obesity and NHL overall.^44^ This discrepancy may be attributed to variations in study settings, study populations, and necessitates further verification. Positive associations were also found for hypertension,^36^ diabetes,^45^ and hypercholesterolaemia.^46^ Contrastingly, a previous meta‐analysis reported the favorable role of alcohol consumption on NHL risk, despite the absence of a biological explanation.^47^

Over the past decade, NHL incidence rates continue to escalate over the past decade, particularly for older males. This upward trend may partially result from the rise in environmental, lifestyle, and metabolic shifts, as well as improvements in cancer diagnosis and thoroughness of registration data collected.^48^ Rising NHL incidence may also associate with the HIV epidemic, as reported in previous studies.^49^

While the incidence of NHL has been increasing in the past decade, the overall trend in NHL mortality has been declining, mainly due to significant advancements and developments in effective novel cancer therapies.^50^ However, it is important to note that there has been a rising trend in mortality observed in Thailand, Colombia, and Estonia. This upward trend in mortality in these countries can be attributed to a notable surge in the elderly population resulting from demographic changes,^51^ as well as potential coexisting medical conditions and comorbidities among the elderly groups.^52^

### 4.2 Limitations

This study is constrained by several limitations. It is important to acknowledge the possibility of underestimating NHL incidence and mortality rates in developing countries due to insufficient manpower, infrastructure, and under‐reporting or missing cancer data reporting. Relying solely on data from GLOBOCAN 2020 does not consider potential influence of the COVID‐19 pandemic, and accuracy of the data itself may introduce uncertainties which could impact the conclusions drawn from this study. While the CI database relies on local cancer registries, the possibility of missing reports in certain regions exists. Incorporating quality control measures to ensure data accuracy and reliability. Cancer registration practices and coding can vary over time and across countries, necessitating caution when interpreting trends and making comparisons.

All selected databases for this study have implemented robust quality, which effectively mitigate the impact of data collection methods, coding templates, and reporting systems. It ensures that discrepancies between populations or over time are unlikely to be solely attributed to differences in data quality. Conducting trend analysis within the same country or region is commonly regarded more reliable as it minimizes potential confounding factors. It takes into account the unique characteristics, demographics, and healthcare systems specific to that particular area, enabling a more accurate assessment of changes occurring within that context.

Unfortunately, the availability of limited data prevented an analysis of trends in different subtypes of NHL. However, exploring such information could yield valuable insights into the epidemiology of NHL, including variations in geographical distribution, risk factors, and specific trends associated with different subtypes.

## 5 CONCLUSIONS

This study delves into complex changes in NHL incidence across age groups, revealing age‐specific patterns that have not been widely explored. Furthermore, investigating the relationship between NHL and lifestyle or metabolic factors adds a unique dimension to the understanding of NHL etiology. Trend analysis by age group contributes to a comprehensive understanding of the temporal pattern of NHL incidence.

Findings of this study reveal a consistent upward trend in NHL incidence over the past decade, particularly among older males, younger populations, and in developed countries. Underlying reasons for this trend are not yet fully understood, but may be related with the increasing prevalence of related risk factors and innovations in early NHL detection methods. NHL mortality rates have been declining in recent years, likely due to significant advancements in chemotherapy, radiation, immunotherapy, and targeted therapies specifically developed for NHL treatment. However, certain countries, including Thailand, Estonia, Colombia, and Slovakia, have experienced an increase in NHL mortality rates. This study verifies the effectiveness of NHL prevention strategies implemented in various countries in the past and provides a targeted trend analysis that can inform future NHL‐related policies. Further research is necessary in exploring possible contributors to these epidemiologic trends, and gain deeper insights into the specific causes and prognosis of NHL across different subtypes.

## AUTHOR CONTRIBUTIONS

**Junjie Huang:** Conceptualization (equal); data curation (equal); formal analysis (equal); supervision (equal); writing – original draft (equal). **Sze Chai Chan:** Data curation (equal); formal analysis (equal); writing – original draft (equal). **Veeleah Lok:** Writing – review and editing (equal). **Lin Zhang:** Writing – review and editing (equal). **Don Eliseo Lucero‐Prisno III:** Writing – review and editing (equal). **Wanghong Xu:** Writing – review and editing (equal). **Zhi‐Jie Zheng:** Writing – review and editing (equal). **Edmar Elcarte:** Writing – review and editing (equal). **Mellissa Withers:** Writing – review and editing (equal). **Martin C. S. Wong:** Conceptualization (equal); supervision (equal); writing – review and editing (equal).

## FUNDING INFORMATION

This research received no grant from any funding agency in the public, commercial, or not‐for profit sectors.

## CONFLICT OF INTEREST STATEMENT

The authors have declared no conflict of interest.

## ETHICS STATEMENT

This study was approved by the Survey and Behavioral Research Ethics Committee, The Chinese University of Hong Kong (No. SBRE‐20‐332).

## PATIENT CONSENT STATEMENT

Our results will be disseminated through media outlets and presentations at scientific conferences and academic events. Given that no patients were recruited for the study, there are no plans to disseminate the results to study participants.

## Supporting information


Figure S1.
Figure S2.
Figure S3.
Figure S4.
Figure S5.
Table S1.

## Data Availability

The data used for the analyses are available upon reasonable request from the corresponding authors.
