# Supplementary material for: Global burden, risk factors, and trends of non‐Hodgkin lymphoma: A worldwide analysis of cancer registries
Source: Cancer Med. 2024 Mar 13;13(5):e7056. doi: 10.1002/cam4.7056 (PMC10935876; doi:10.1002/cam4.7056)
Supplement: Supplementary file 1 — Figure S1. Figure S2. Figure S3. Figure S4. Figure S5. Table S1. [file CAM4-13-e7056-s001.pdf]

Supplementary Legends

**Supplementary Figure 1**	Incidence and mortality trends for individual countries
**Supplementary Figure 2**	Results of joinpoint regression for individual countries
**Supplementary Figure 3**	AAPC of incidence of NHL aged 50 years and older
**Supplementary Figure 4**	AAPC of incidence of NHL aged < 50 years old
**Supplementary Figure 5**	AAPC of incidence of NHL aged < 40 years old
**Supplementary Table 1**	Data source for the trend analysis.

Supplementary Figure 1: The plots of incidence and mortality trends for each country

Male

Asia

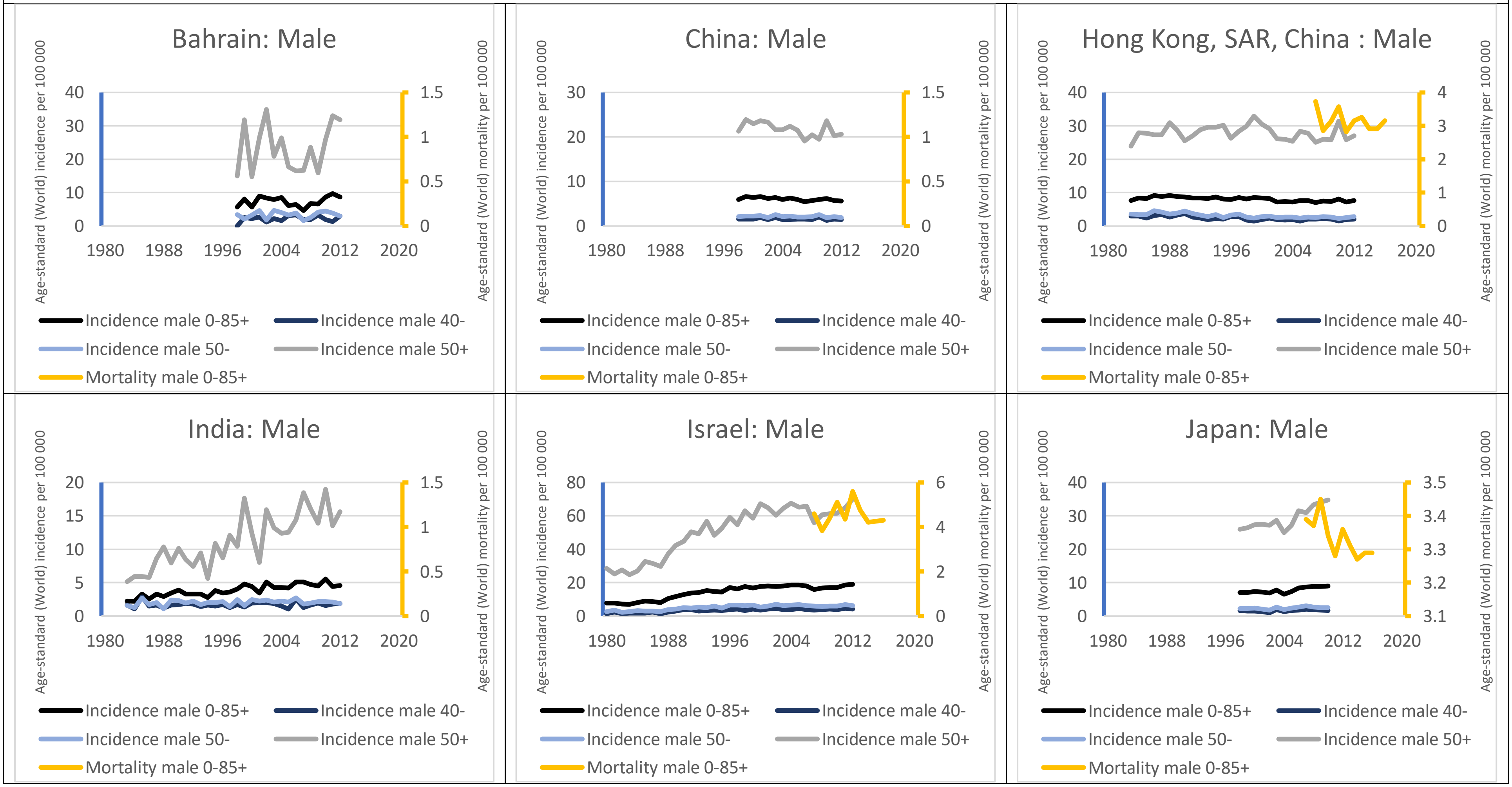

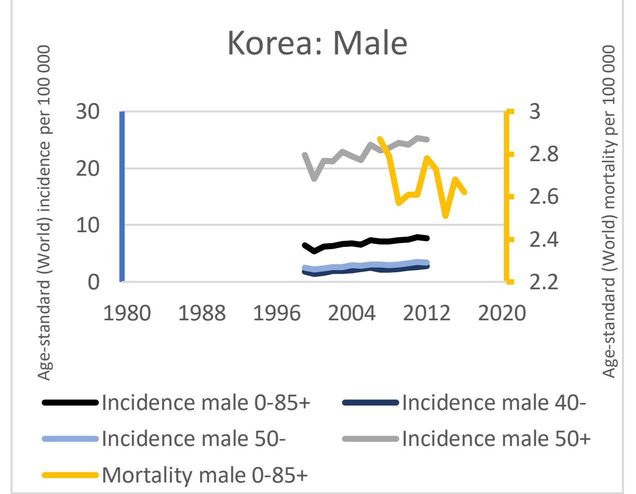
Korea: Male
Age-standard (World) incidence per 100 000
Age-standard (World) mortality per 100 000
30
20
10
0
3
2.8
2.6
2.4
2.2
1980 1988 1996 2004 2012 2020
Incidence male 0-85+
Incidence male 40-
Incidence male 50-
Incidence male 50+
Mortality male 0-85+

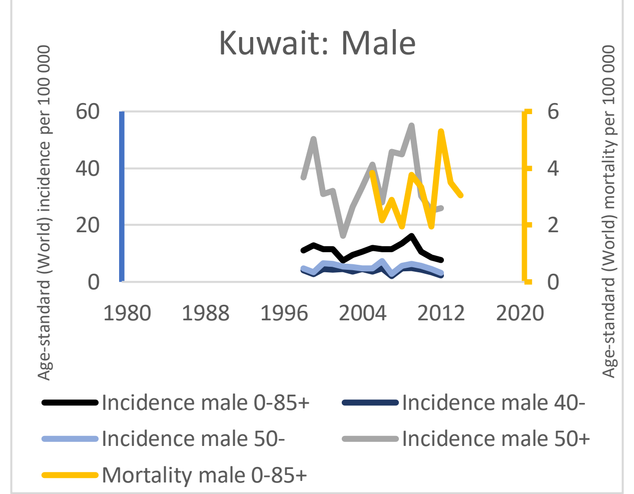
Kuwait: Male
Age-standard (World) incidence per 100 000
Age-standard (World) mortality per 100 000
60
40
20
0
6
4
2
0
1980 1988 1996 2004 2012 2020
Incidence male 0-85+
Incidence male 40-
Incidence male 50-
Incidence male 50+
Mortality male 0-85+

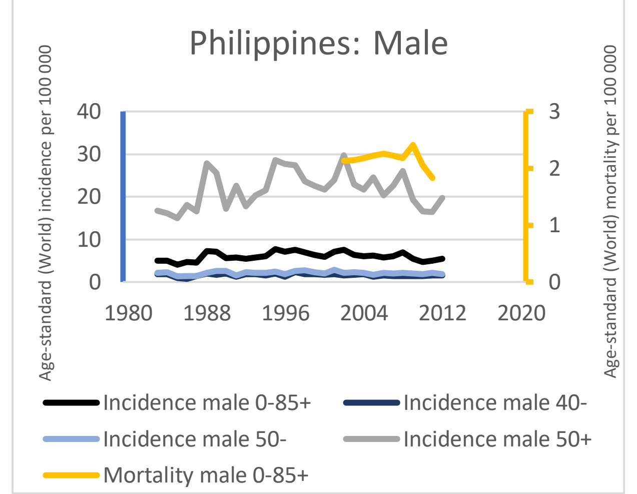
Philippines: Male
Age-standard (World) incidence per 100 000
Age-standard (World) mortality per 100 000
40
30
20
10
0
3
2
1
0
1980 1988 1996 2004 2012 2020
Incidence male 0-85+
Incidence male 40-
Incidence male 50-
Incidence male 50+
Mortality male 0-85+

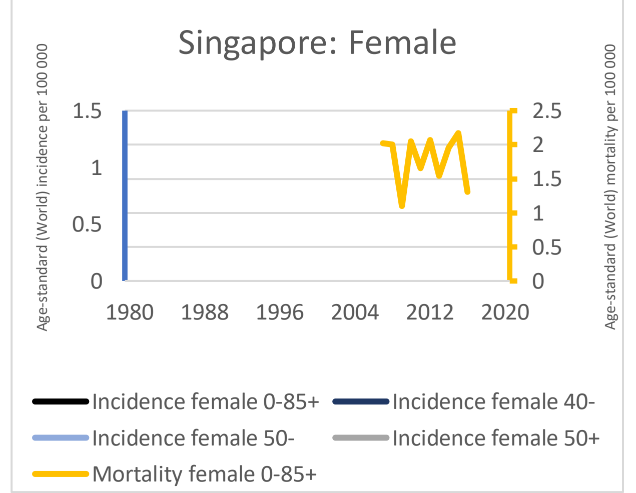
Singapore: Female
Age-standard (World) incidence per 100 000
Age-standard (World) mortality per 100 000
1.5
1
0.5
0
2.5
2
1.5
1
0.5
0
1980 1988 1996 2004 2012 2020
Incidence female 0-85+
Incidence female 40-
Incidence female 50-
Incidence female 50+
Mortality female 0-85+

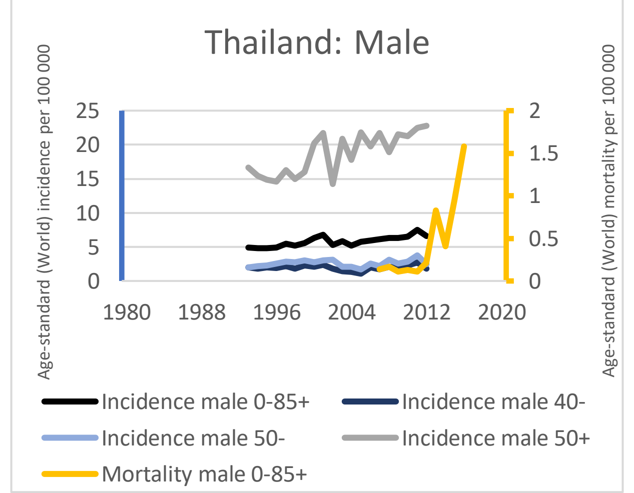
Thailand: Male
Age-standard (World) incidence per 100 000
Age-standard (World) mortality per 100 000
25
20
15
10
5
0
2
1.5
1
0.5
0
1980 1988 1996 2004 2012 2020
Incidence male 0-85+
Incidence male 40-
Incidence male 50-
Incidence male 50+
Mortality male 0-85+

Oceania

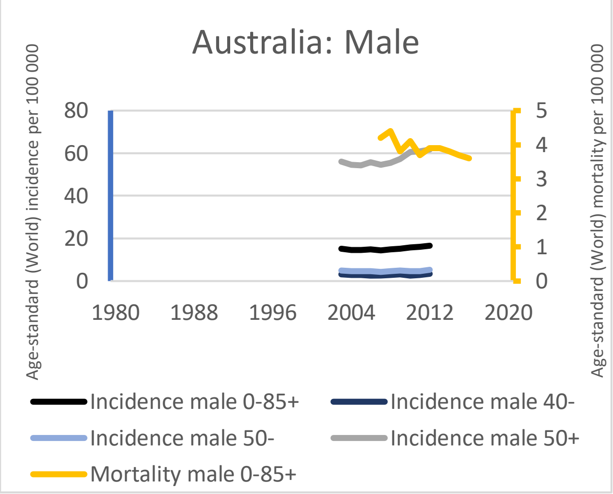

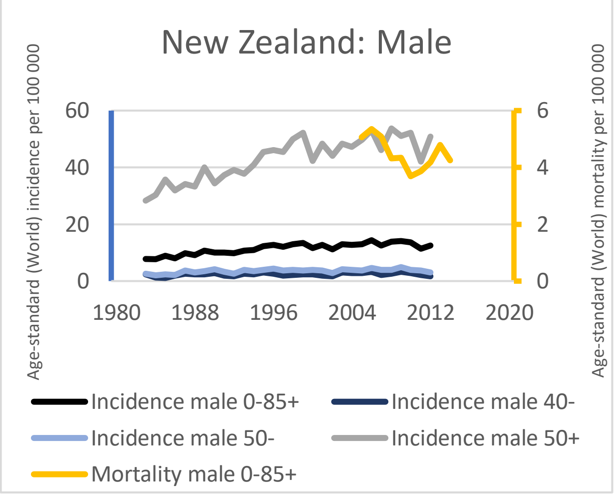

Northern America

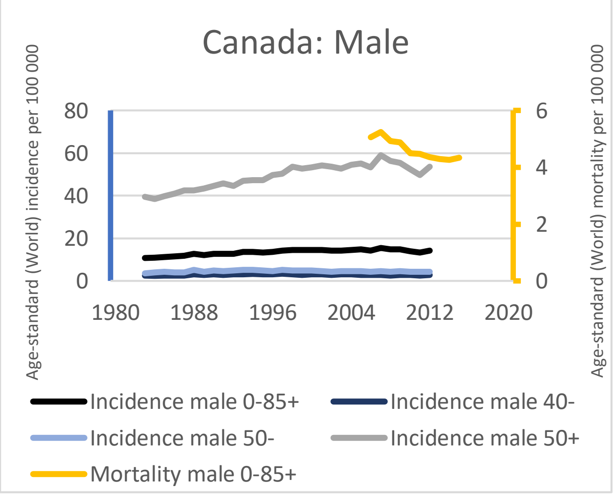

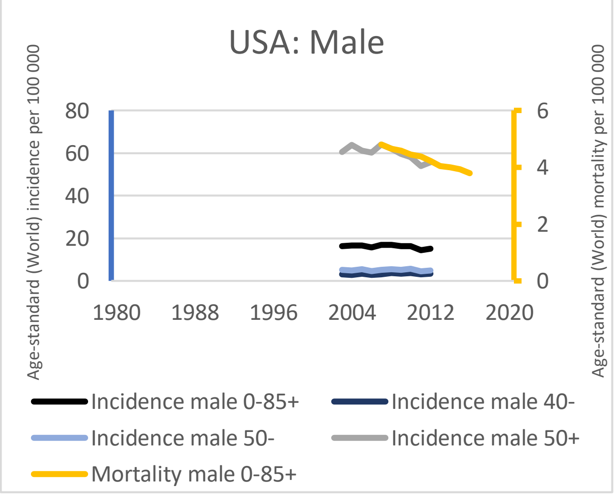

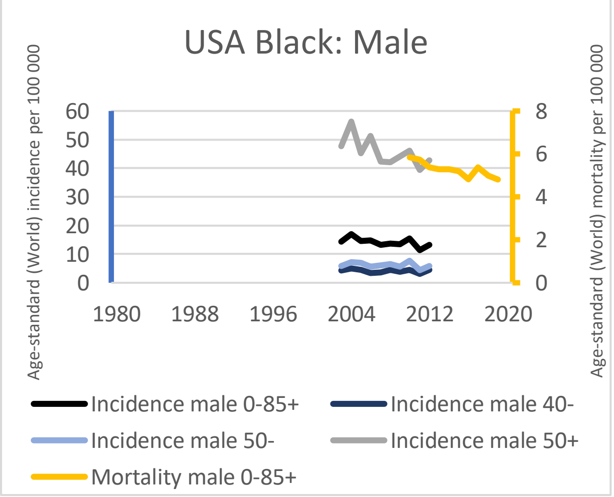

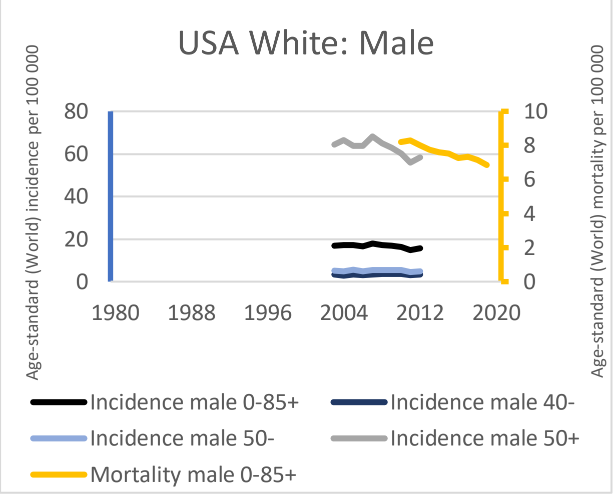

Southern America

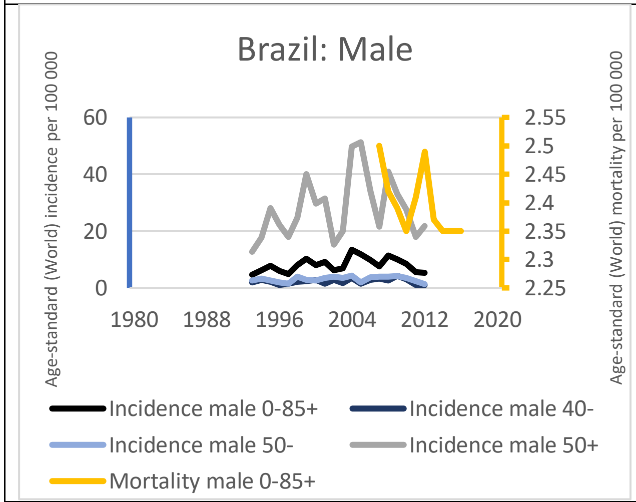

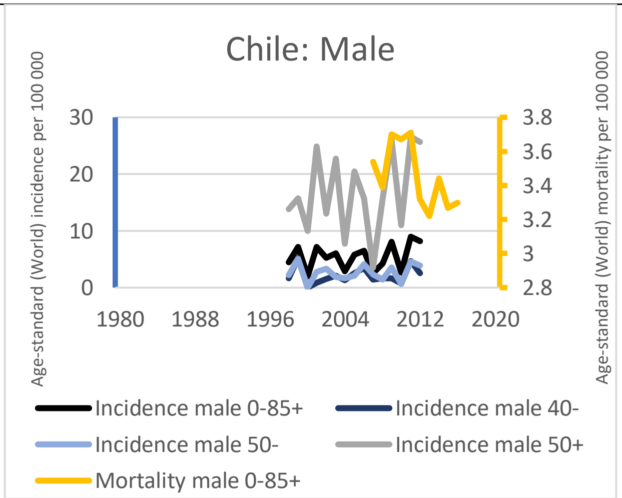

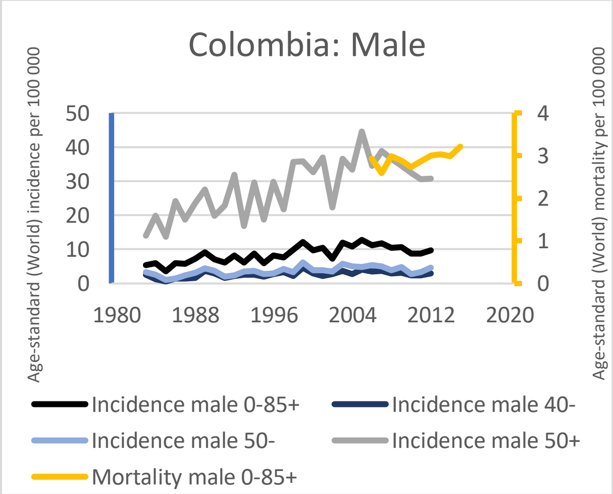

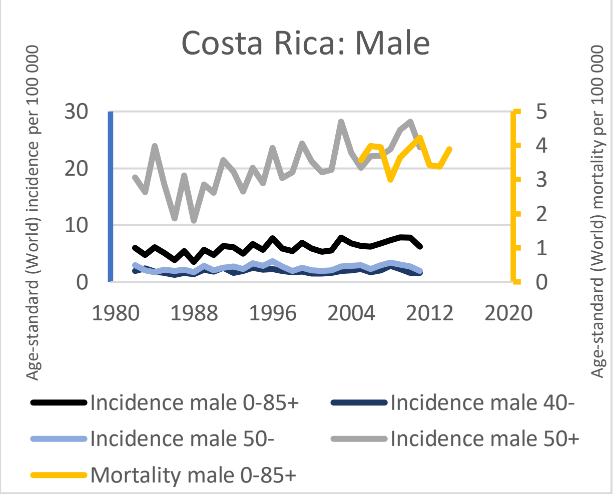

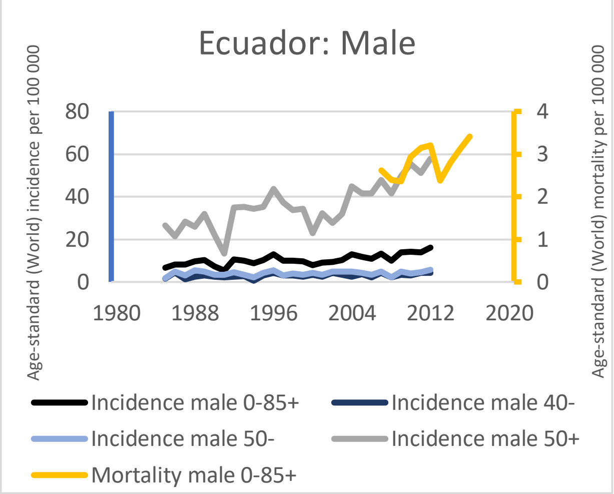

Northern Europe

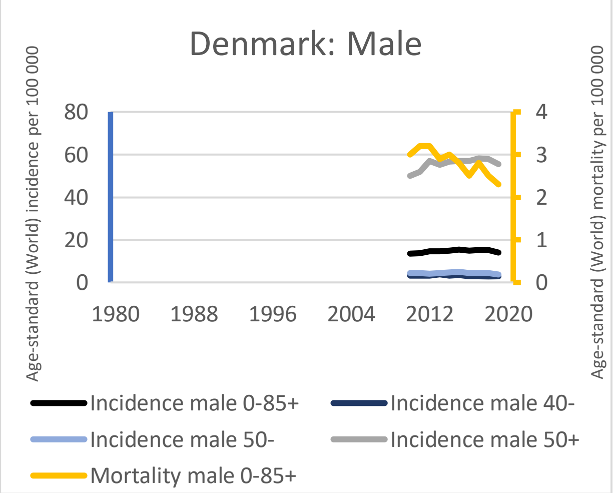

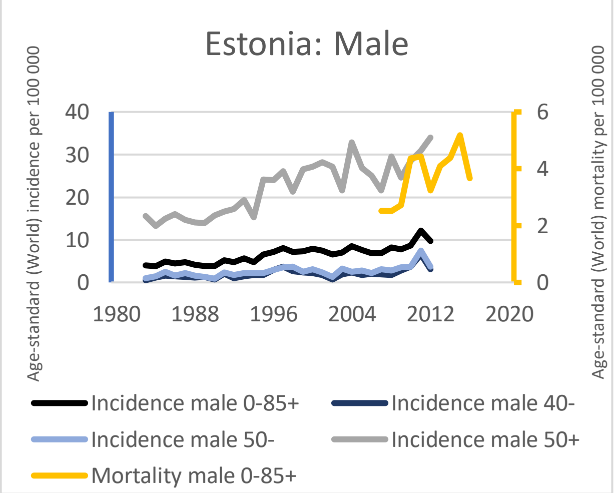

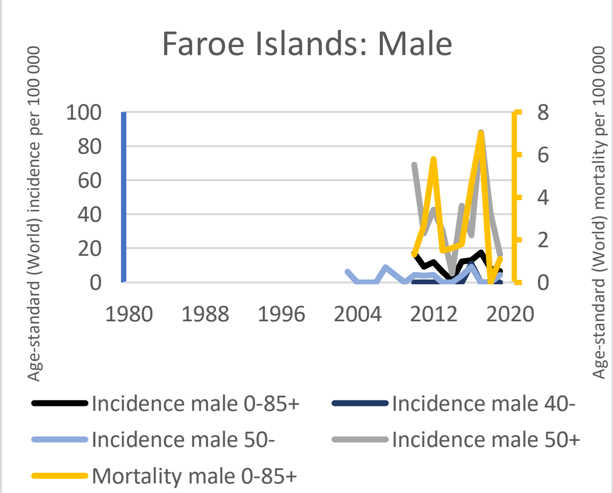

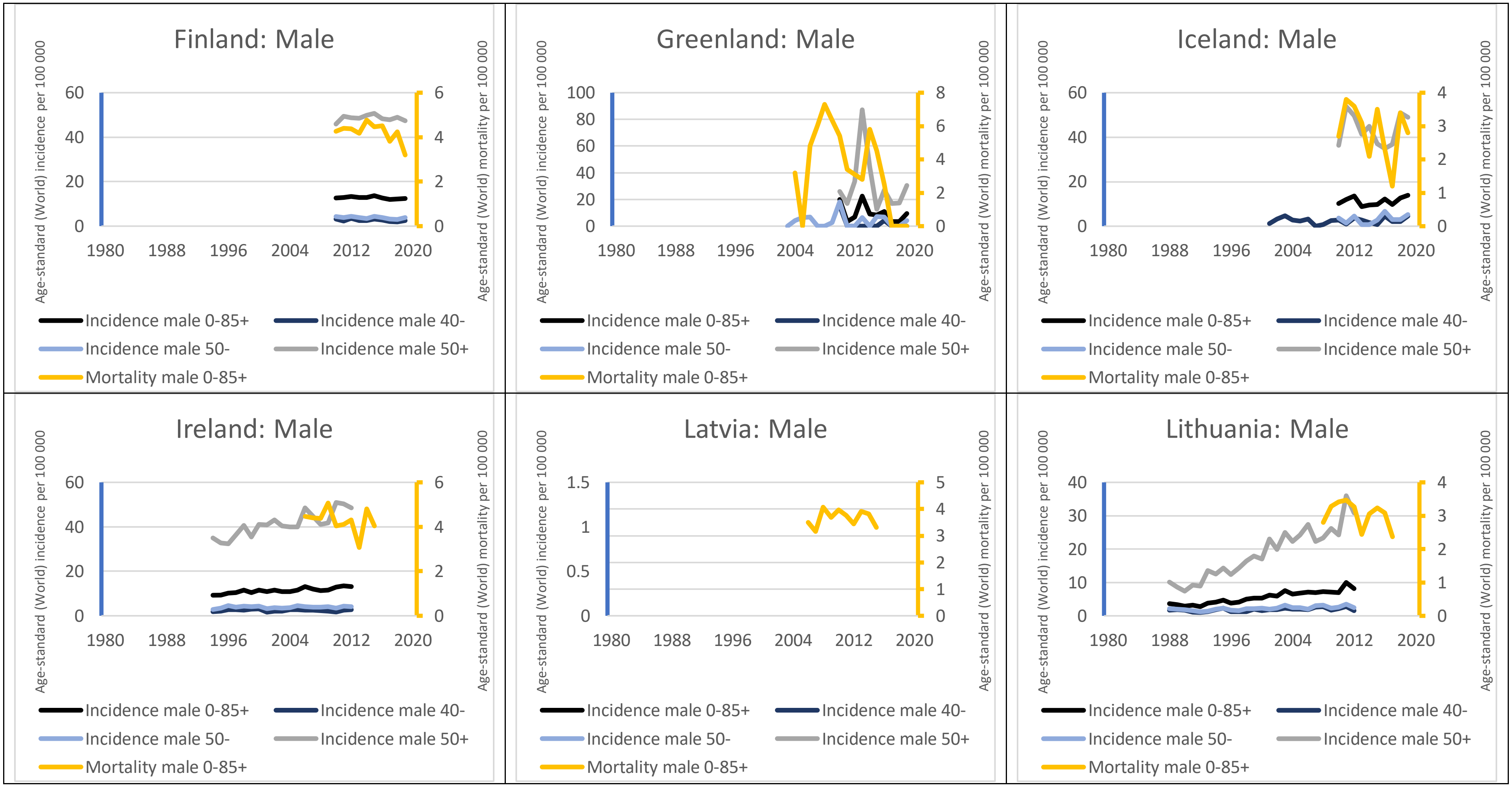
Finland: Male
Age-standard (World) incidence per 100 000
Age-standard (World) mortality per 100 000
60
40
20
0
6
4
2
0
1980
1988
1996
2004
2012
2020
Incidence male 0-85+
Incidence male 40-
Incidence male 50-
Incidence male 50+
Mortality male 0-85+
Greenland: Male
Age-standard (World) incidence per 100 000
Age-standard (World) mortality per 100 000
100
80
60
40
20
0
8
6
4
2
0
1980
1988
1996
2004
2012
2020
Incidence male 0-85+
Incidence male 40-
Incidence male 50-
Incidence male 50+
Mortality male 0-85+
Iceland: Male
Age-standard (World) incidence per 100 000
Age-standard (World) mortality per 100 000
60
40
20
0
4
3
2
1
0
1980
1988
1996
2004
2012
2020
Incidence male 0-85+
Incidence male 40-
Incidence male 50-
Incidence male 50+
Mortality male 0-85+
Ireland: Male
Age-standard (World) incidence per 100 000
Age-standard (World) mortality per 100 000
60
40
20
0
6
4
2
0
1980
1988
1996
2004
2012
2020
Incidence male 0-85+
Incidence male 40-
Incidence male 50-
Incidence male 50+
Mortality male 0-85+
Latvia: Male
Age-standard (World) incidence per 100 000
Age-standard (World) mortality per 100 000
1.5
1
0.5
0
5
4
3
2
1
0
1980
1988
1996
2004
2012
2020
Incidence male 0-85+
Incidence male 40-
Incidence male 50-
Incidence male 50+
Mortality male 0-85+
Lithuania: Male
Age-standard (World) incidence per 100 000
Age-standard (World) mortality per 100 000
40
30
20
10
0
4
3
2
1
0
1980
1988
1996
2004
2012
2020
Incidence male 0-85+
Incidence male 40-
Incidence male 50-
Incidence male 50+
Mortality male 0-85+

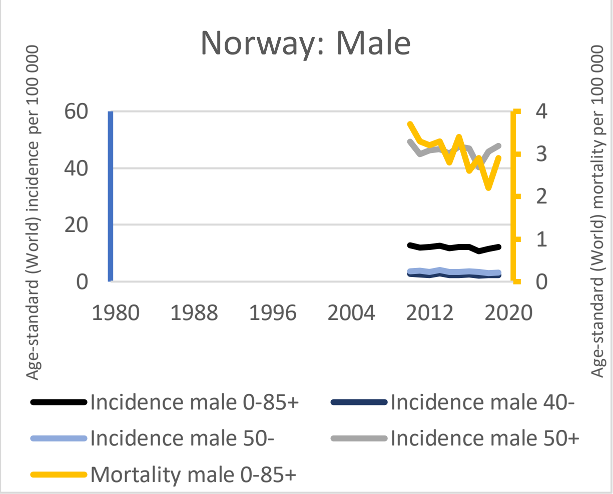

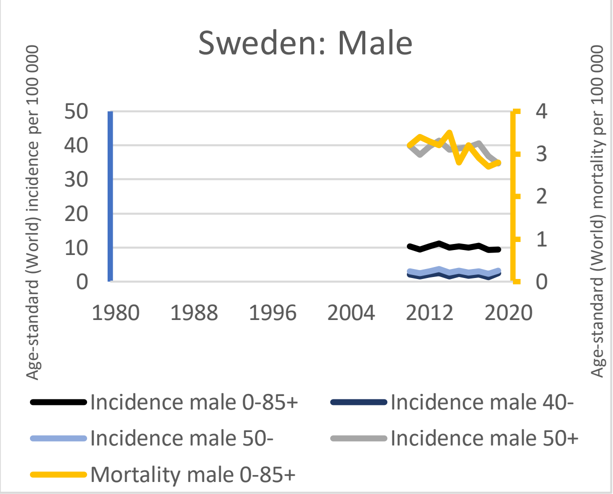

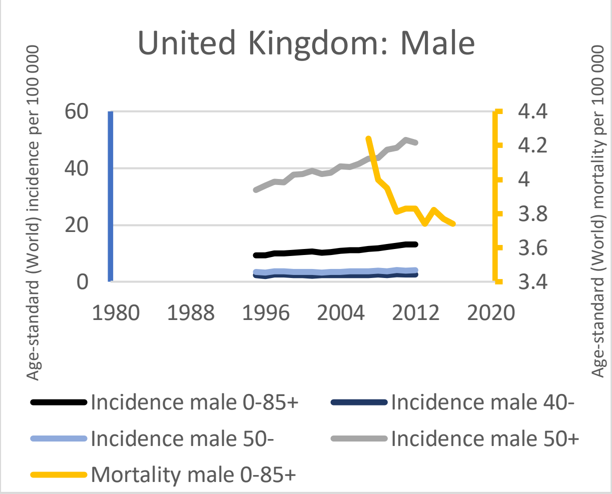

Western Europe

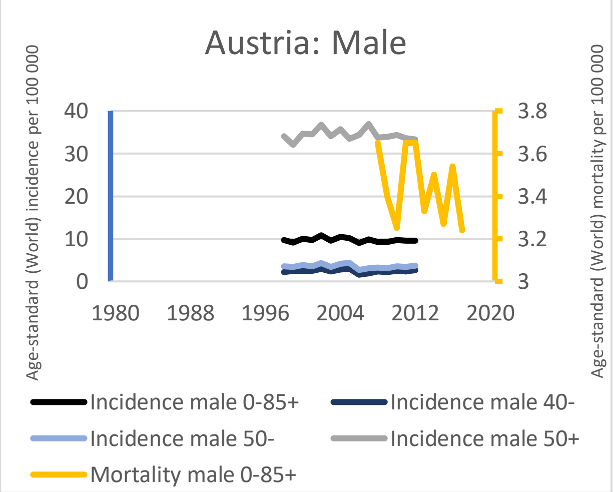

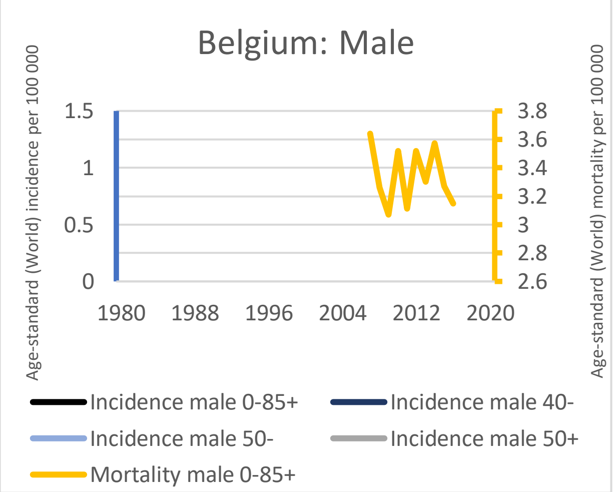

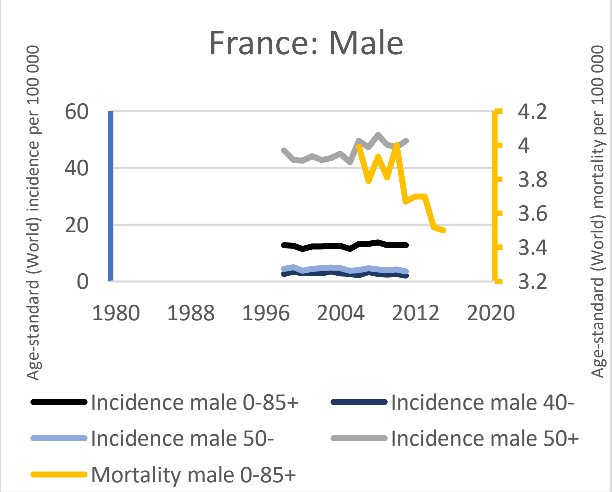

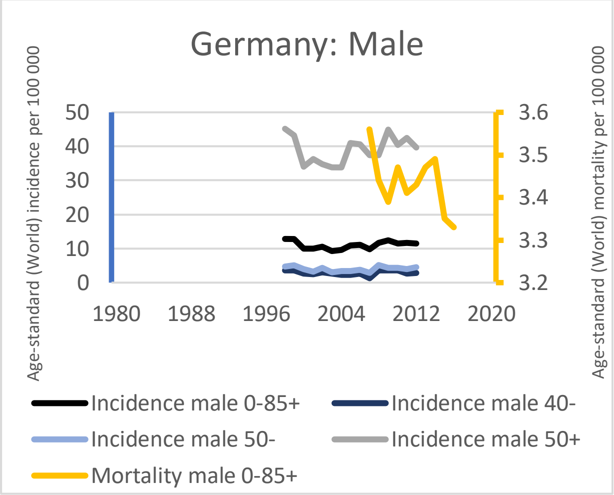

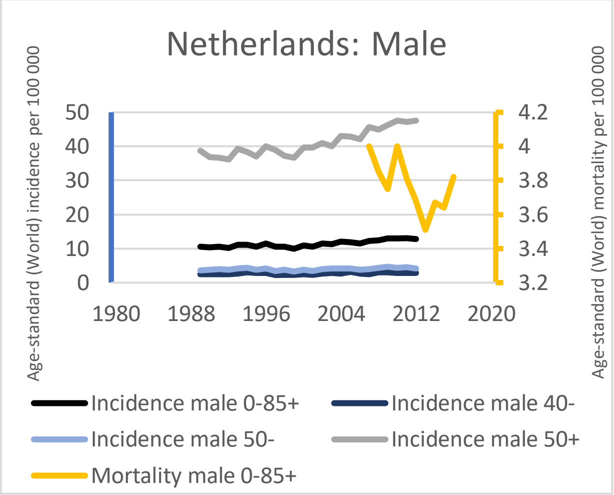

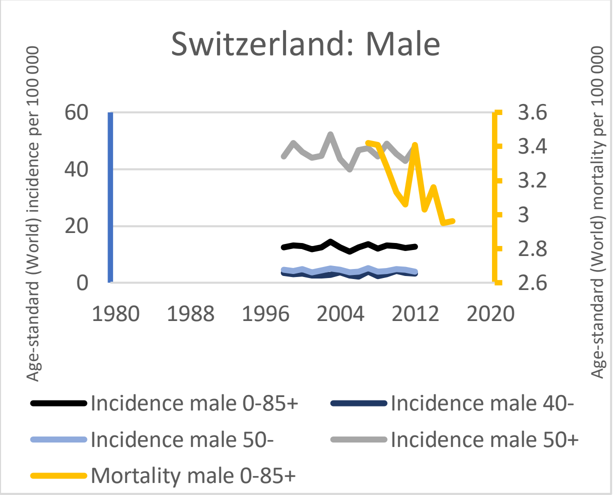

Southern Europe

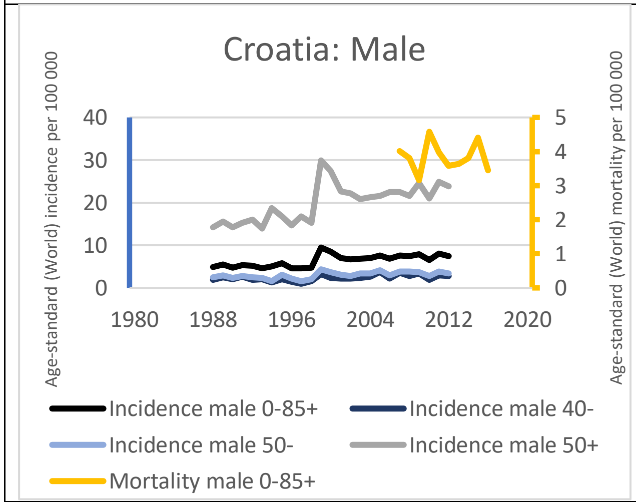

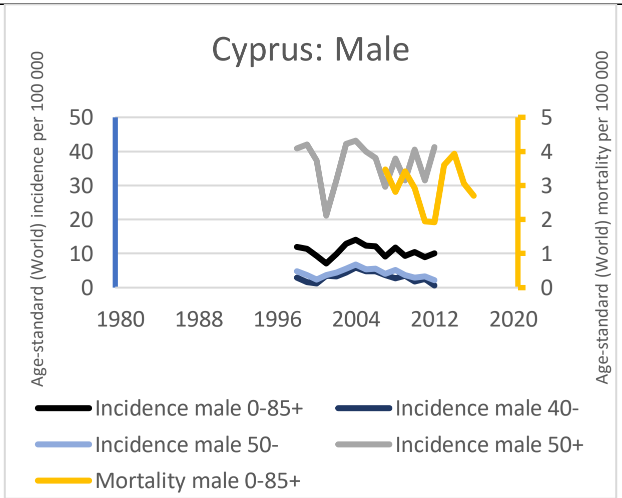

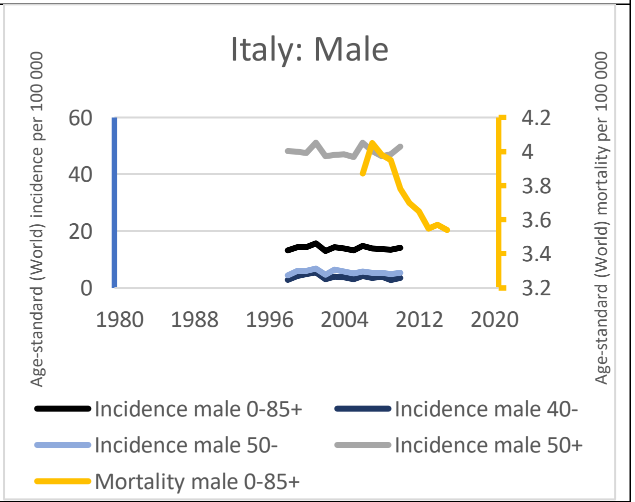

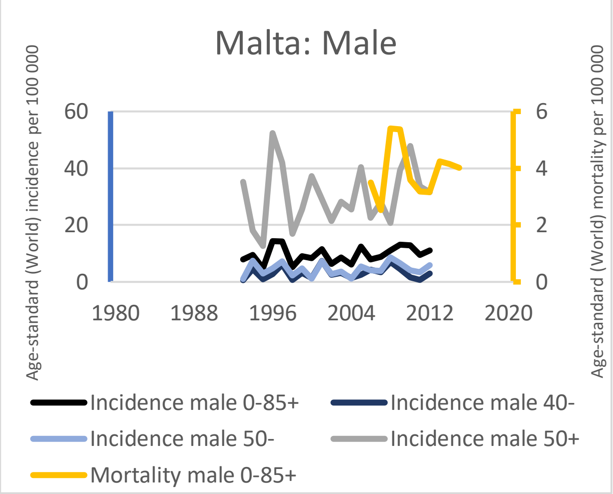
Malta: Male
Age-standard (World) incidence per 100 000
Age-standard (World) mortality per 100 000
60
40
20
0
6
4
2
0
1980 1988 1996 2004 2012 2020
Incidence male 0-85+
Incidence male 40-
Incidence male 50-
Incidence male 50+
Mortality male 0-85+

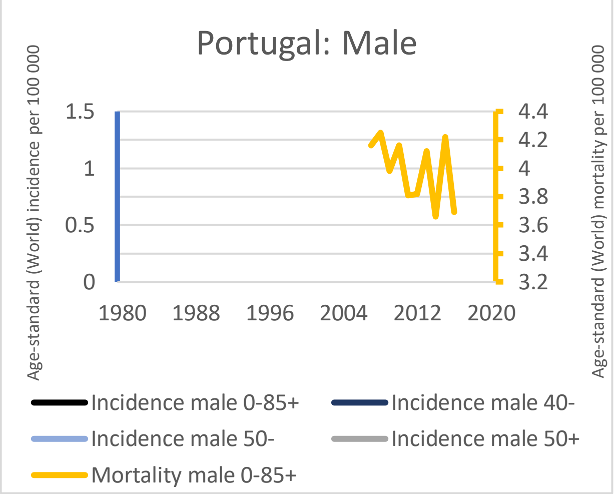
Portugal: Male
Age-standard (World) incidence per 100 000
Age-standard (World) mortality per 100 000
1.5
1
0.5
0
4.4
4.2
4
3.8
3.6
3.4
3.2
1980 1988 1996 2004 2012 2020
Incidence male 0-85+
Incidence male 40-
Incidence male 50-
Incidence male 50+
Mortality male 0-85+

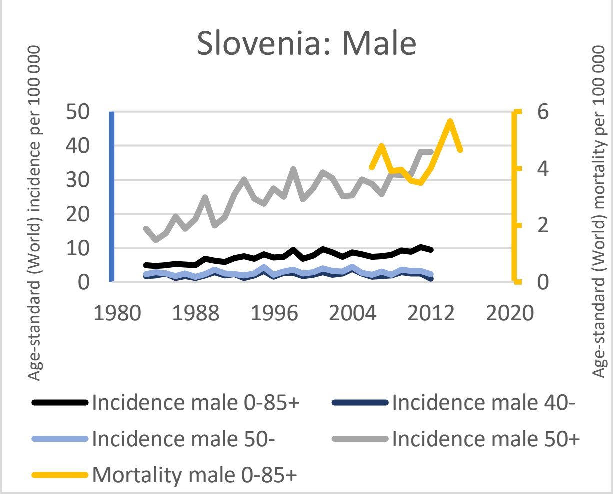
Slovenia: Male
Age-standard (World) incidence per 100 000
Age-standard (World) mortality per 100 000
50
40
30
20
10
0
6
4
2
0
1980 1988 1996 2004 2012 2020
Incidence male 0-85+
Incidence male 40-
Incidence male 50-
Incidence male 50+
Mortality male 0-85+

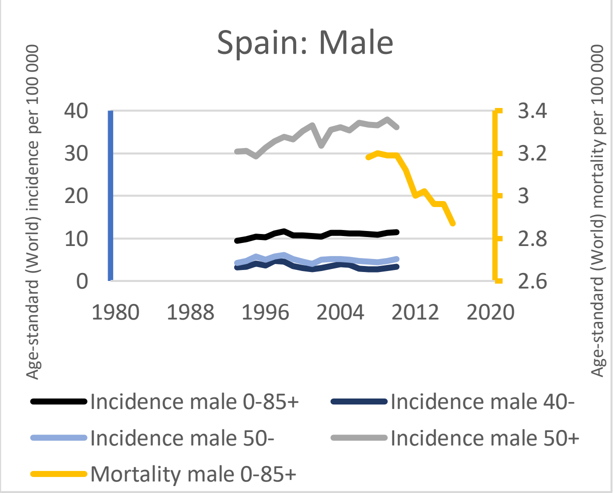
Spain: Male
Age-standard (World) incidence per 100 000
Age-standard (World) mortality per 100 000
40
30
20
10
0
3.4
3.2
3
2.8
2.6
1980 1988 1996 2004 2012 2020
Incidence male 0-85+
Incidence male 40-
Incidence male 50-
Incidence male 50+
Mortality male 0-85+

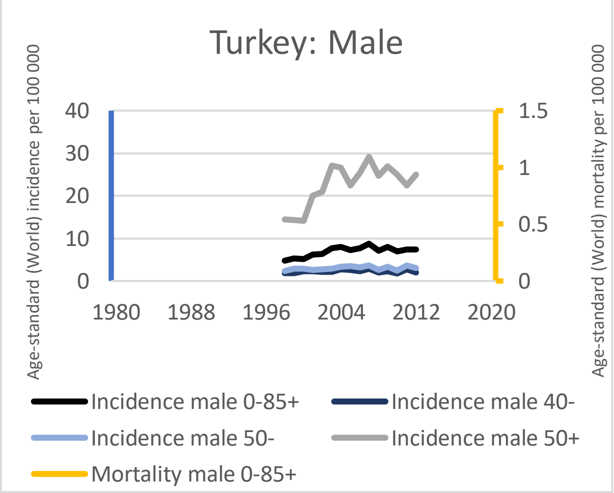
Turkey: Male
Age-standard (World) incidence per 100 000
Age-standard (World) mortality per 100 000
40
30
20
10
0
1.5
1
0.5
0
1980 1988 1996 2004 2012 2020
Incidence male 0-85+
Incidence male 40-
Incidence male 50-
Incidence male 50+
Mortality male 0-85+

Eastern Europe

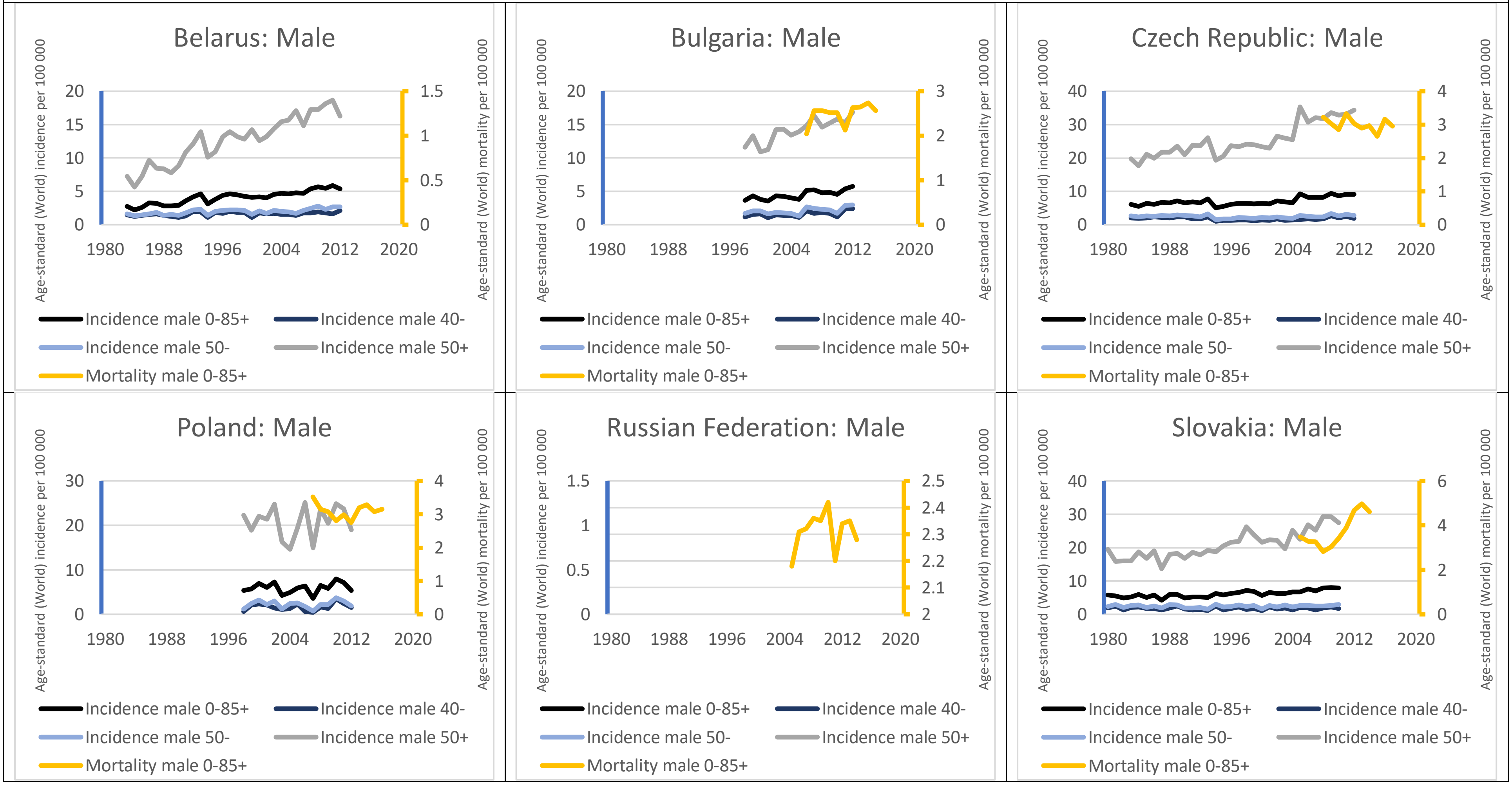

<table>
<tr><th colspan="3">Africa</th></tr>
<tr><td>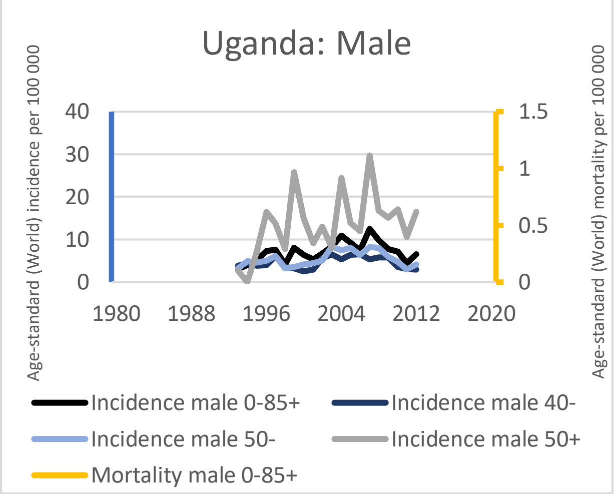
</td><td></td><td></td></tr>
</table>

Female

Asia

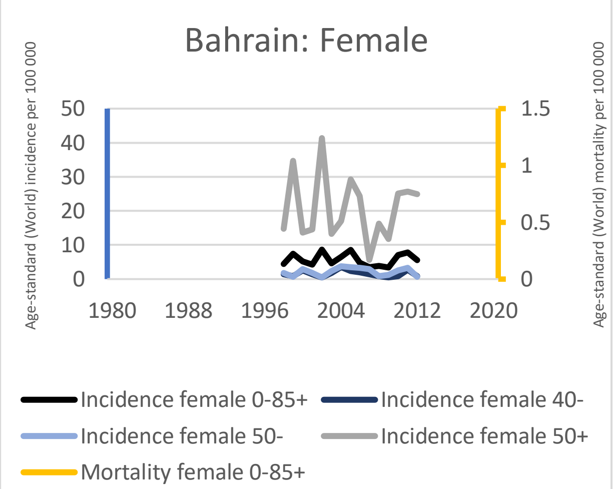
Bahrain: Female
Age-standard (World) incidence per 100 000
Age-standard (World) mortality per 100 000
50 40 30 20 10 0
1.5 1 0.5 0
1980 1988 1996 2004 2012 2020
Incidence female 0-85+
Incidence female 40-
Incidence female 50-
Incidence female 50+
Mortality female 0-85+

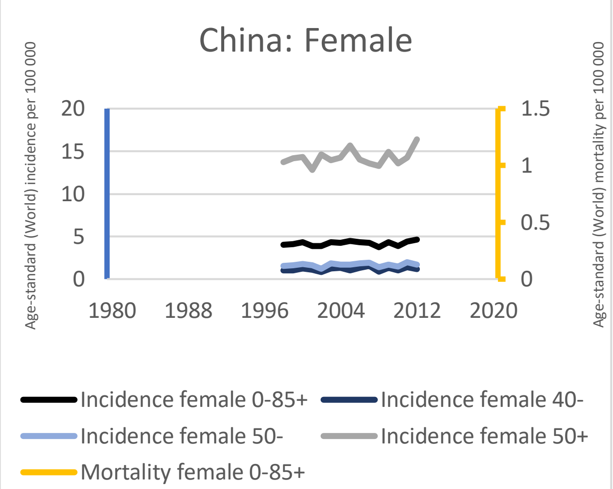
China: Female
Age-standard (World) incidence per 100 000
Age-standard (World) mortality per 100 000
20 15 10 5 0
1.5 1 0.5 0
1980 1988 1996 2004 2012 2020
Incidence female 0-85+
Incidence female 40-
Incidence female 50-
Incidence female 50+
Mortality female 0-85+

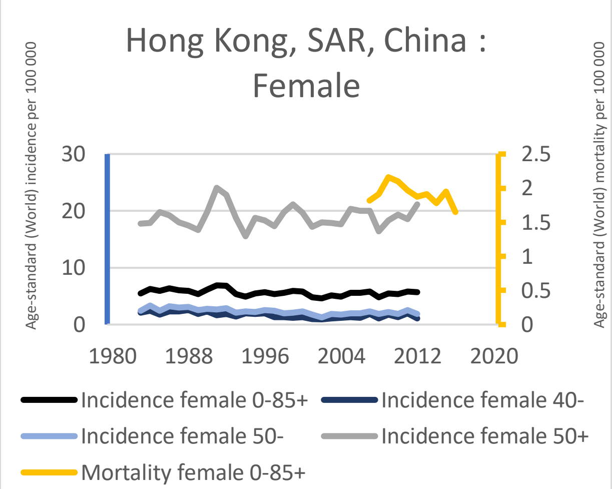
Hong Kong, SAR, China : Female
Age-standard (World) incidence per 100 000
Age-standard (World) mortality per 100 000
30 20 10 0
2.5 2 1.5 1 0.5 0
1980 1988 1996 2004 2012 2020
Incidence female 0-85+
Incidence female 40-
Incidence female 50-
Incidence female 50+
Mortality female 0-85+

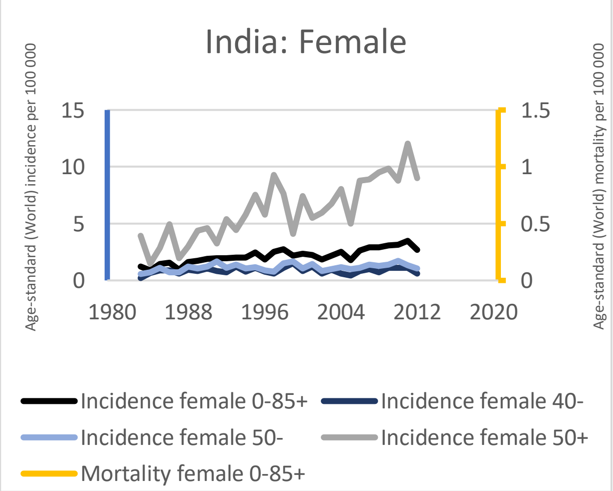
India: Female
Age-standard (World) incidence per 100 000
Age-standard (World) mortality per 100 000
15 10 5 0
1.5 1 0.5 0
1980 1988 1996 2004 2012 2020
Incidence female 0-85+
Incidence female 40-
Incidence female 50-
Incidence female 50+
Mortality female 0-85+

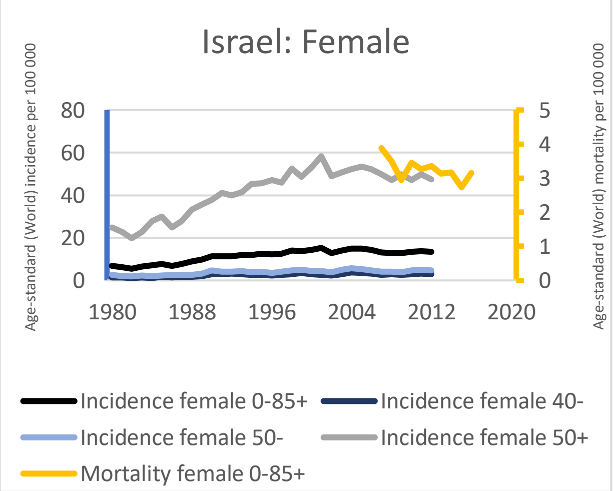
Israel: Female
Age-standard (World) incidence per 100 000
Age-standard (World) mortality per 100 000
80 60 40 20 0
5 4 3 2 1 0
1980 1988 1996 2004 2012 2020
Incidence female 0-85+
Incidence female 40-
Incidence female 50-
Incidence female 50+
Mortality female 0-85+

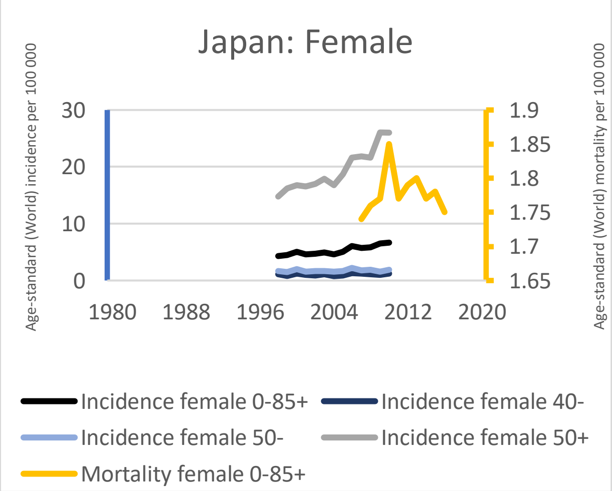
Japan: Female
Age-standard (World) incidence per 100 000
Age-standard (World) mortality per 100 000
30 20 10 0
1.9 1.85 1.8 1.75 1.7 1.65
1980 1988 1996 2004 2012 2020
Incidence female 0-85+
Incidence female 40-
Incidence female 50-
Incidence female 50+
Mortality female 0-85+

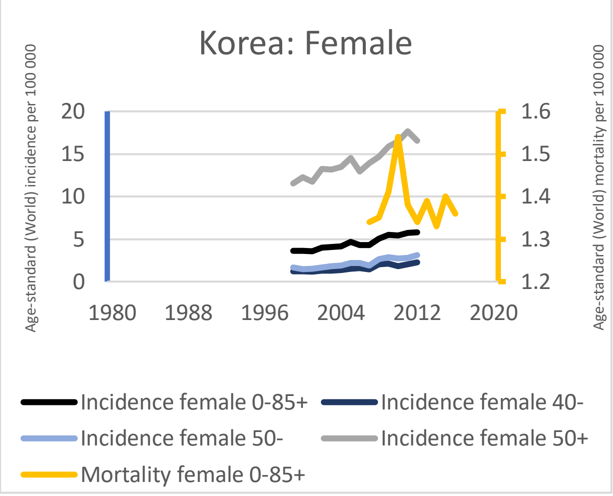
Korea: Female
Age-standard (World) incidence per 100 000
Age-standard (World) mortality per 100 000
20 15 10 5 0
1.6 1.5 1.4 1.3 1.2
1980 1988 1996 2004 2012 2020
Incidence female 0-85+
Incidence female 40-
Incidence female 50-
Incidence female 50+
Mortality female 0-85+

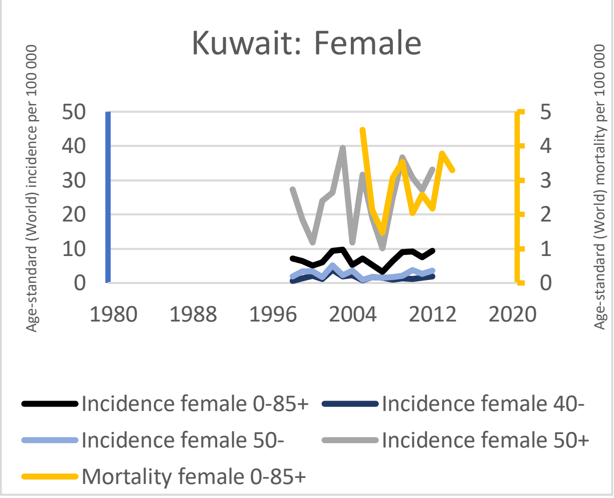
Kuwait: Female
Age-standard (World) incidence per 100 000
Age-standard (World) mortality per 100 000
50 40 30 20 10 0
5 4 3 2 1 0
1980 1988 1996 2004 2012 2020
Incidence female 0-85+
Incidence female 40-
Incidence female 50-
Incidence female 50+
Mortality female 0-85+

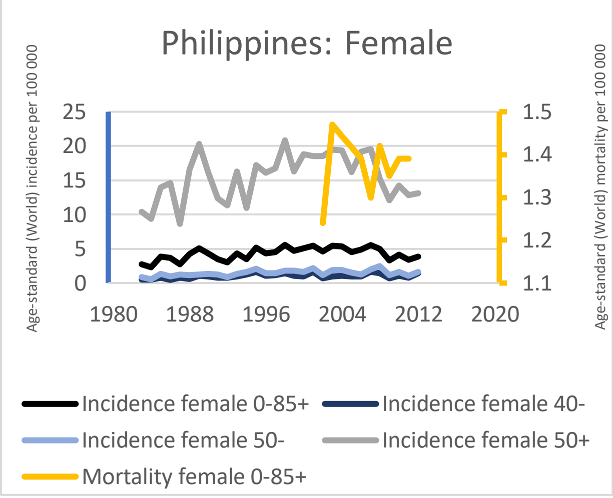
Philippines: Female
Age-standard (World) incidence per 100 000
Age-standard (World) mortality per 100 000
25 20 15 10 5 0
1.5 1.4 1.3 1.2 1.1
1980 1988 1996 2004 2012 2020
Incidence female 0-85+
Incidence female 40-
Incidence female 50-
Incidence female 50+
Mortality female 0-85+

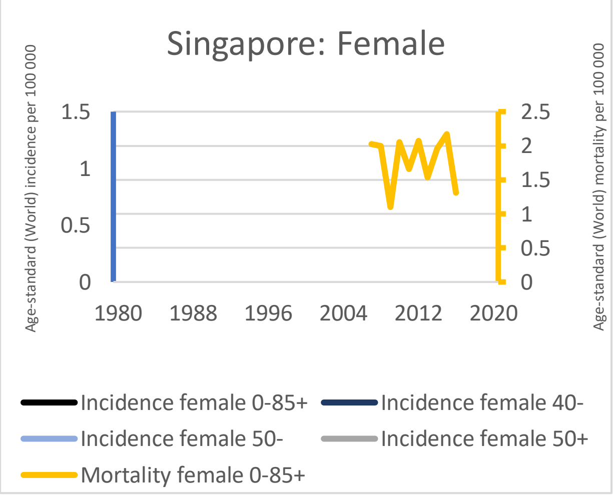
Singapore: Female
Age-standard (World) incidence per 100 000
Age-standard (World) mortality per 100 000
1.5 1 0.5 0
2.5 2 1.5 1 0.5 0
1980 1988 1996 2004 2012 2020
Incidence female 0-85+
Incidence female 40-
Incidence female 50-
Incidence female 50+
Mortality female 0-85+

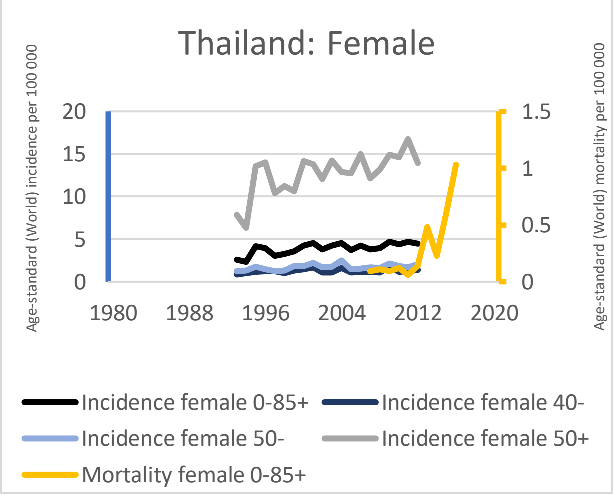
Thailand: Female
Age-standard (World) incidence per 100 000
Age-standard (World) mortality per 100 000
20 15 10 5 0
1.5 1 0.5 0
1980 1988 1996 2004 2012 2020
Incidence female 0-85+
Incidence female 40-
Incidence female 50-
Incidence female 50+
Mortality female 0-85+

Oceania

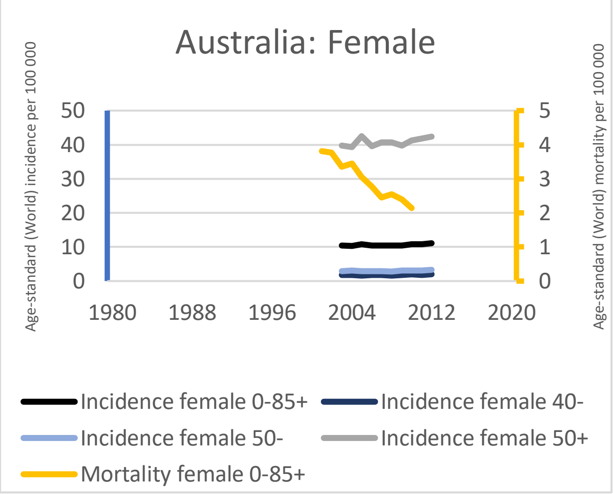

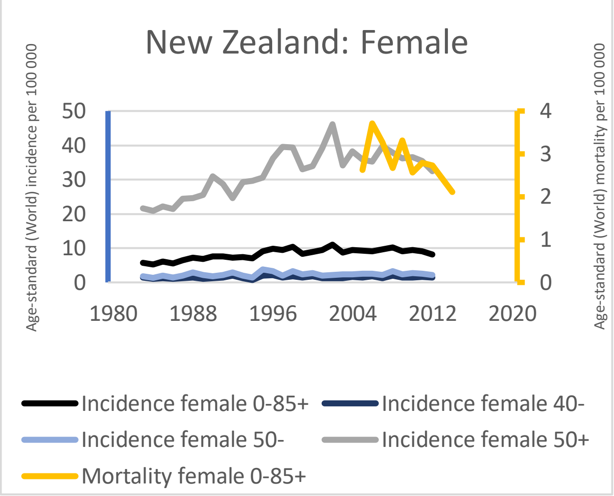

Northern America

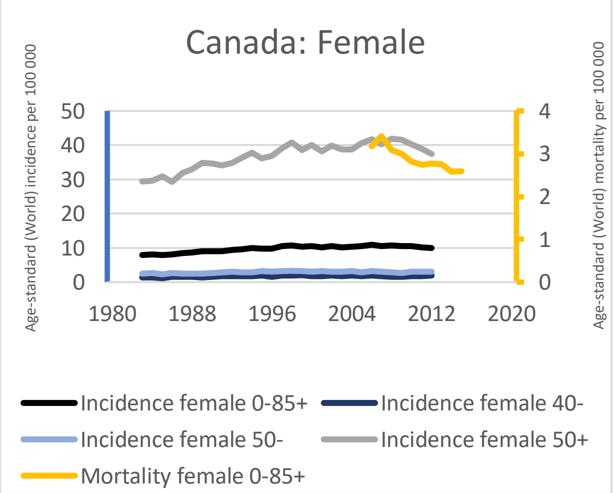

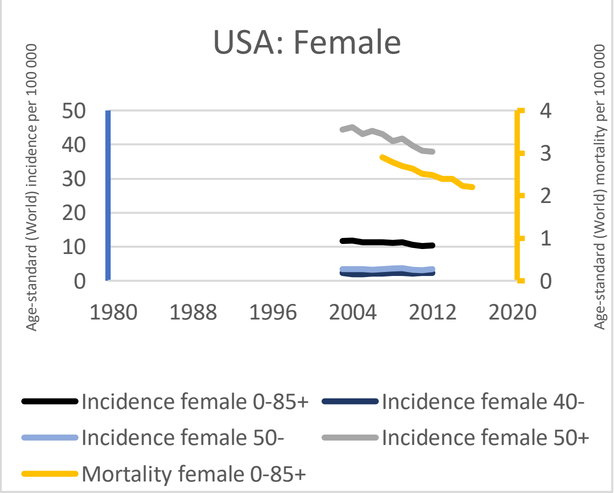

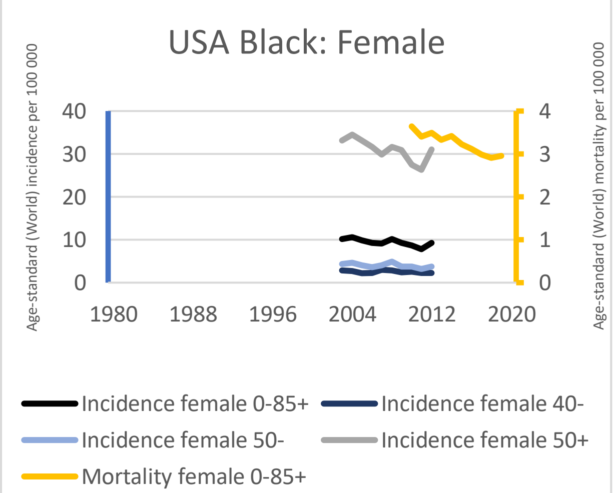

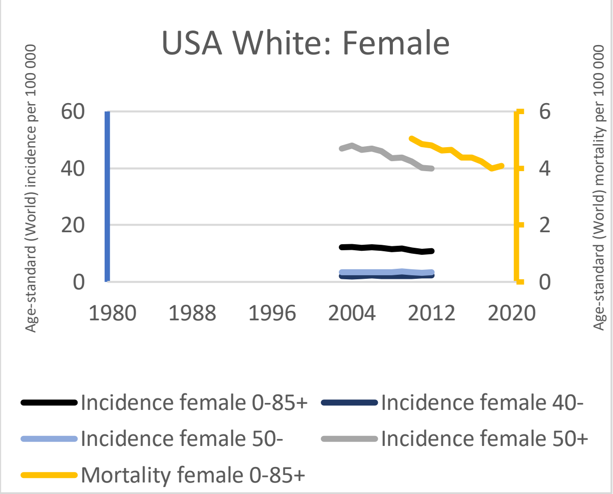

Southern America

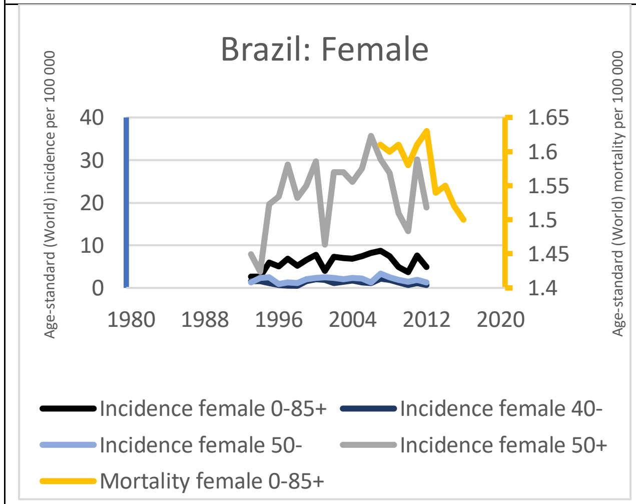

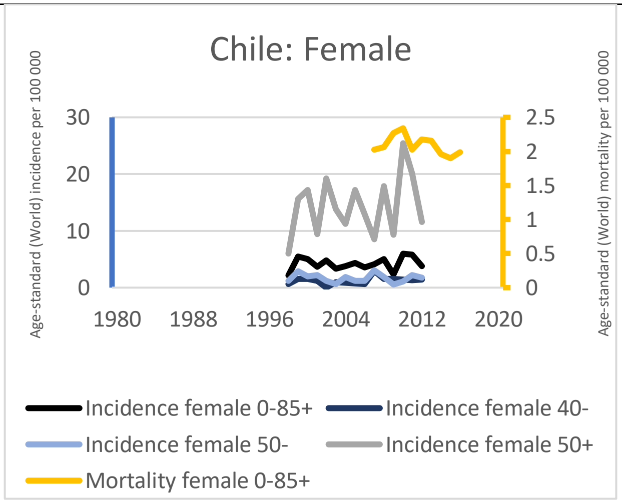

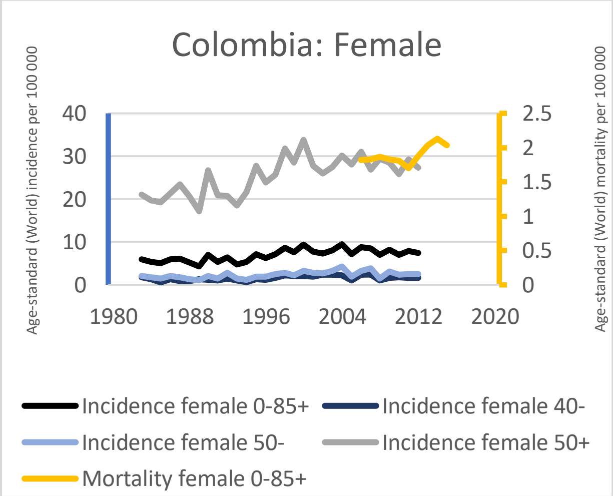

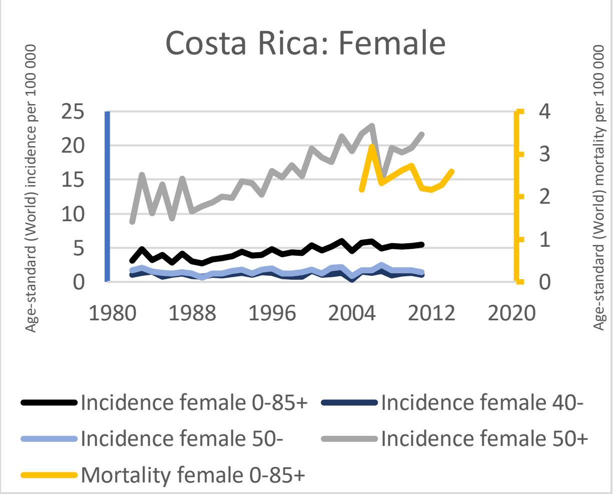

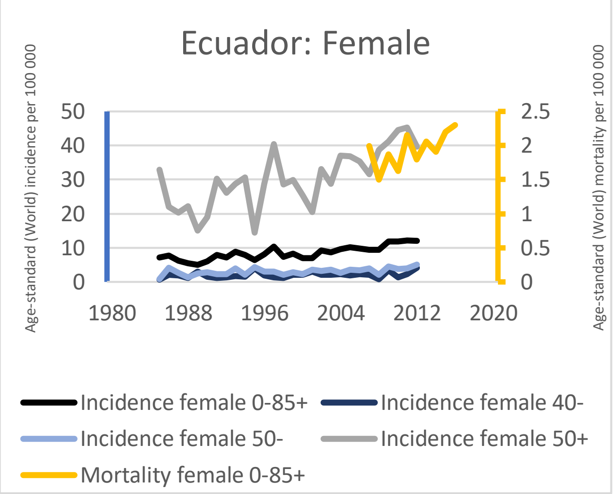

Northern Europe

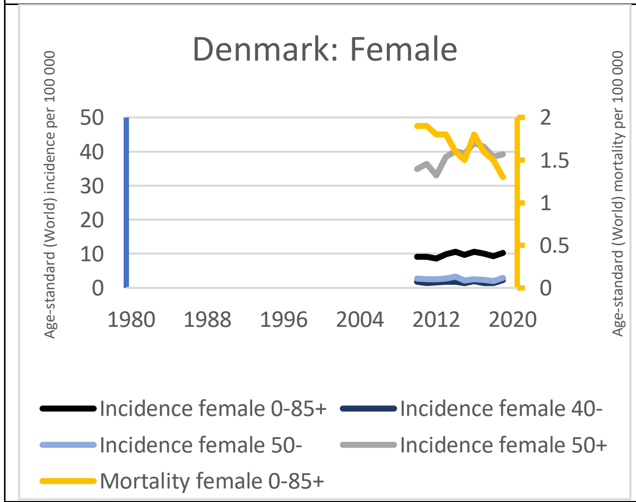

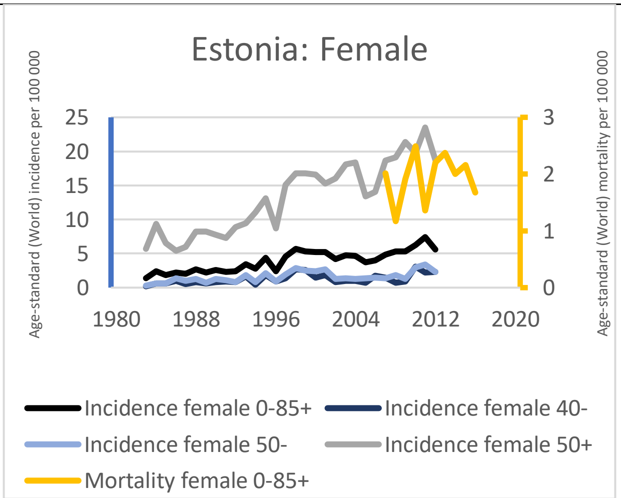

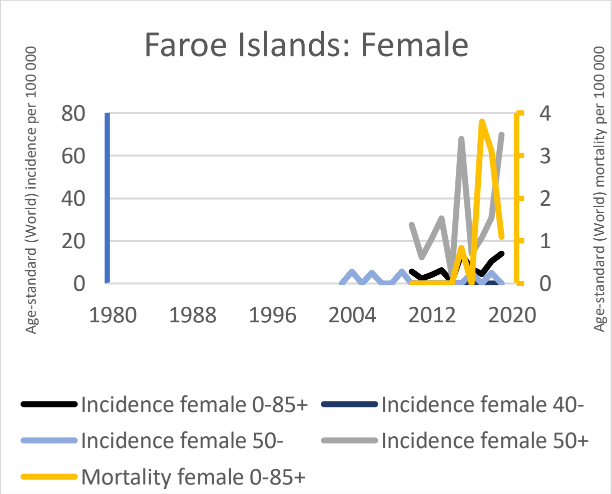

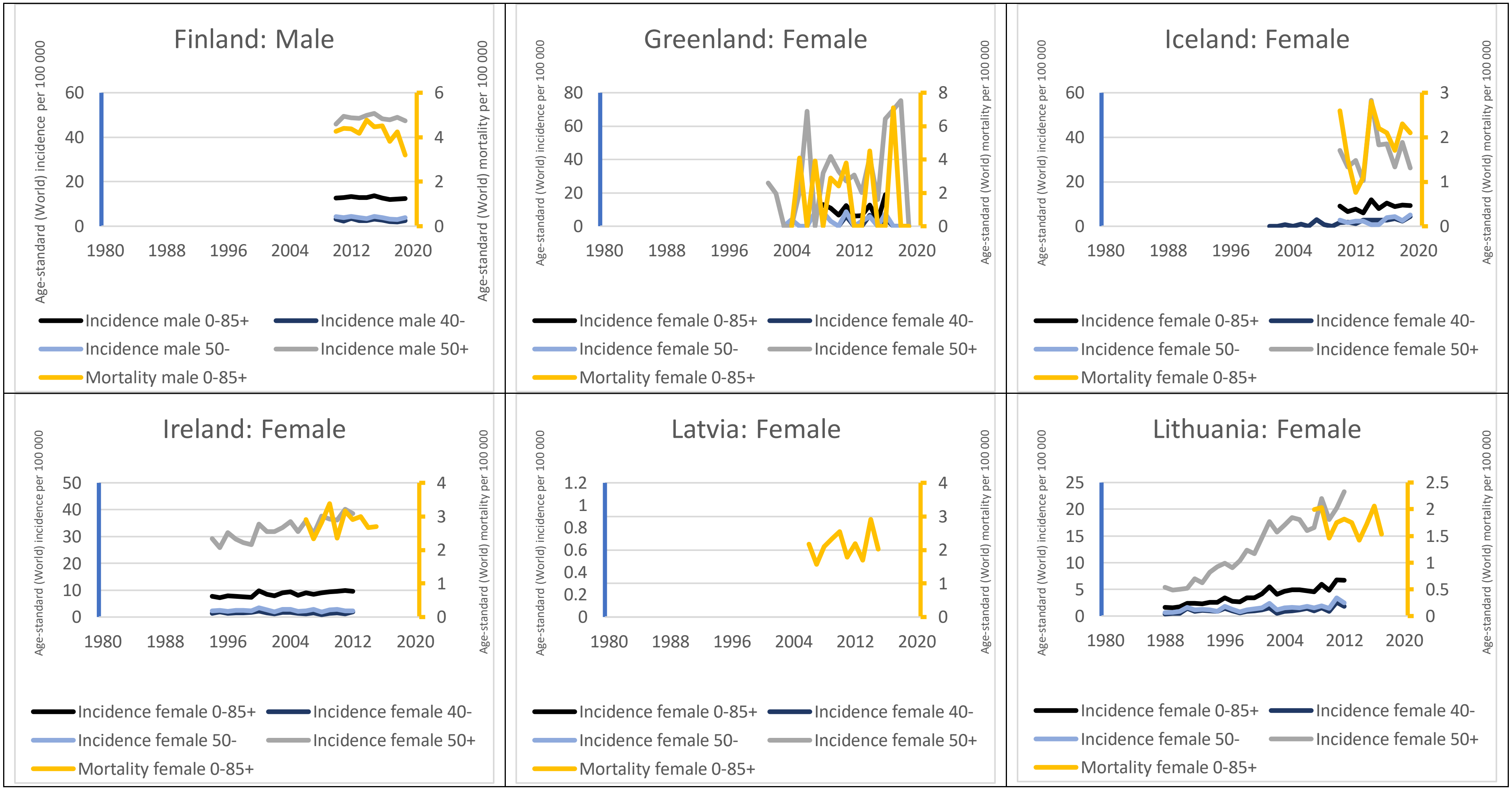
Finland: Male
Age-standard (World) incidence per 100 000
Age-standard (World) mortality per 100 000
60
40
20
0
6
4
2
0
1980
1988
1996
2004
2012
2020
Incidence male 0-85+
Incidence male 40-
Incidence male 50-
Incidence male 50+
Mortality male 0-85+
Greenland: Female
Age-standard (World) incidence per 100 000
Age-standard (World) mortality per 100 000
80
60
40
20
0
8
6
4
2
0
1980
1988
1996
2004
2012
2020
Incidence female 0-85+
Incidence female 40-
Incidence female 50-
Incidence female 50+
Mortality female 0-85+
Iceland: Female
Age-standard (World) incidence per 100 000
Age-standard (World) mortality per 100 000
60
40
20
0
3
2
1
0
1980
1988
1996
2004
2012
2020
Incidence female 0-85+
Incidence female 40-
Incidence female 50-
Incidence female 50+
Mortality female 0-85+
Ireland: Female
Age-standard (World) incidence per 100 000
Age-standard (World) mortality per 100 000
50
40
30
20
10
0
4
3
2
1
0
1980
1988
1996
2004
2012
2020
Incidence female 0-85+
Incidence female 40-
Incidence female 50-
Incidence female 50+
Mortality female 0-85+
Latvia: Female
Age-standard (World) incidence per 100 000
Age-standard (World) mortality per 100 000
1.2
1
0.8
0.6
0.4
0.2
0
4
3
2
1
0
1980
1988
1996
2004
2012
2020
Incidence female 0-85+
Incidence female 40-
Incidence female 50-
Incidence female 50+
Mortality female 0-85+
Lithuania: Female
Age-standard (World) incidence per 100 000
Age-standard (World) mortality per 100 000
25
20
15
10
5
0
2.5
2
1.5
1
0.5
0
1980
1988
1996
2004
2012
2020
Incidence female 0-85+
Incidence female 40-
Incidence female 50-
Incidence female 50+
Mortality female 0-85+

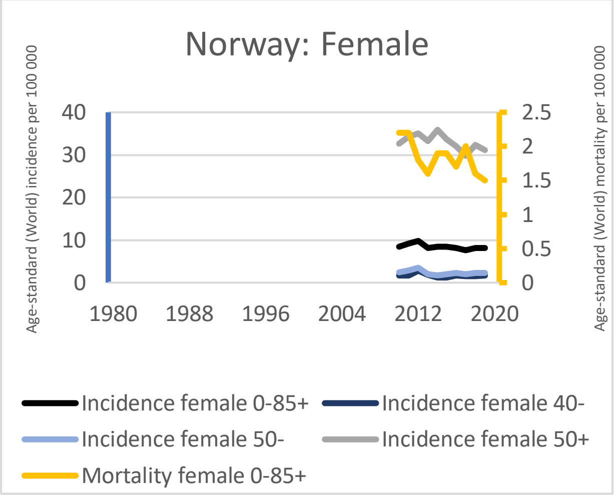

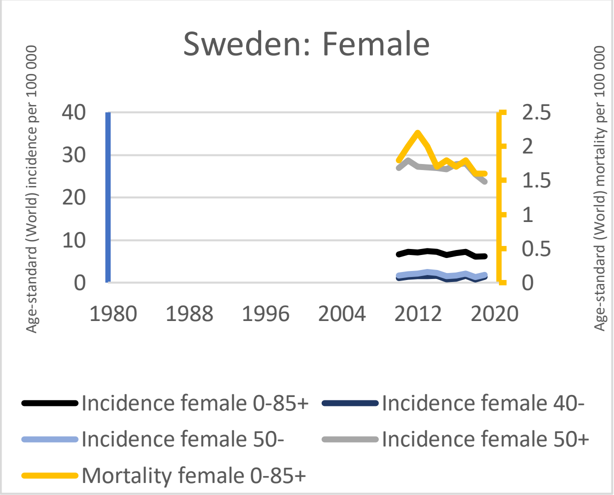

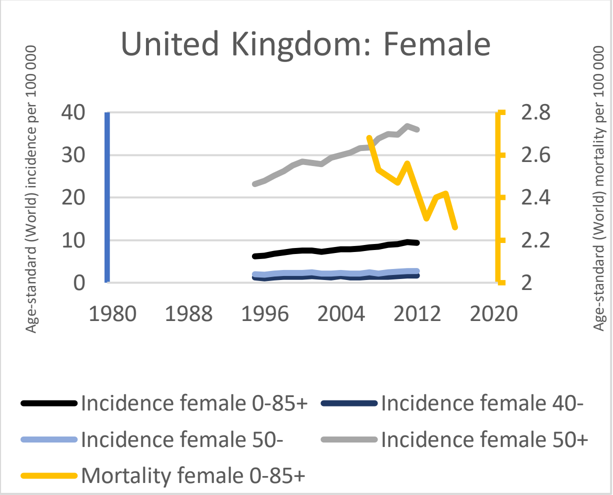

Western Europe

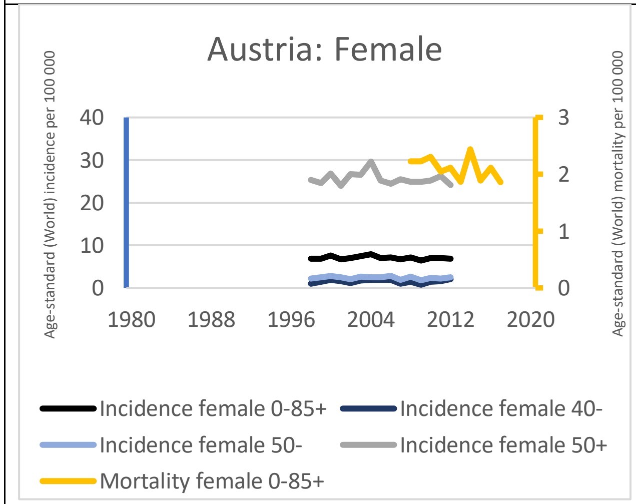

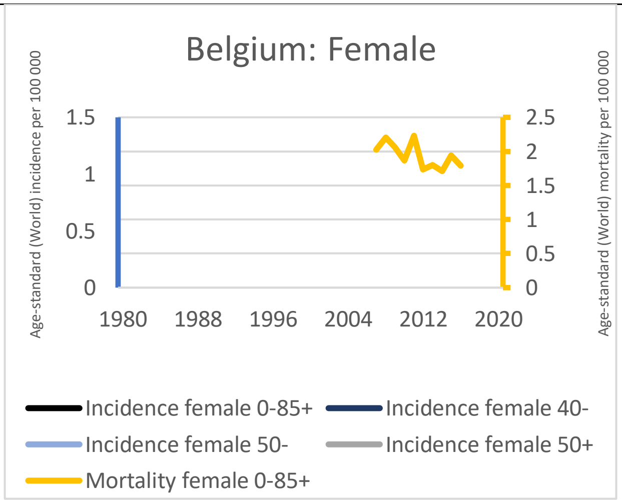

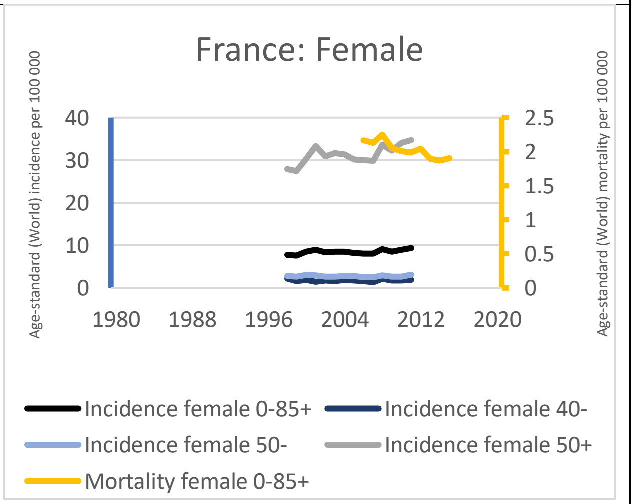

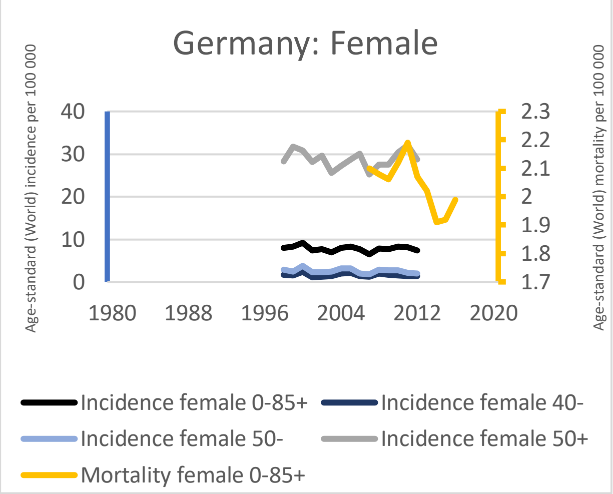

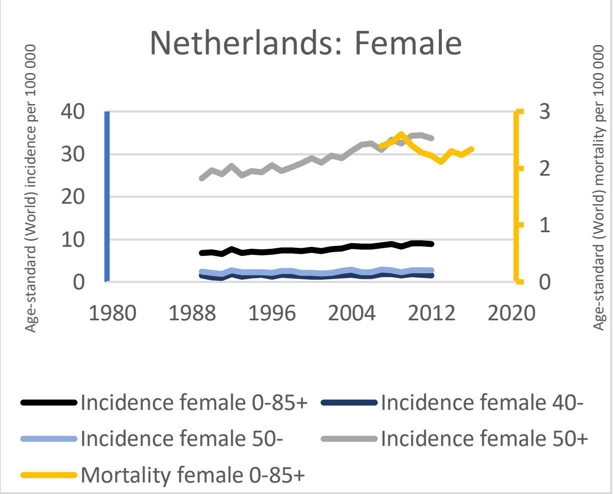

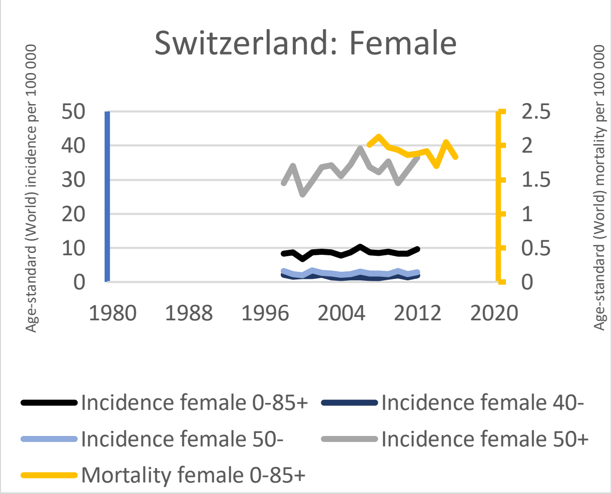

Southern Europe

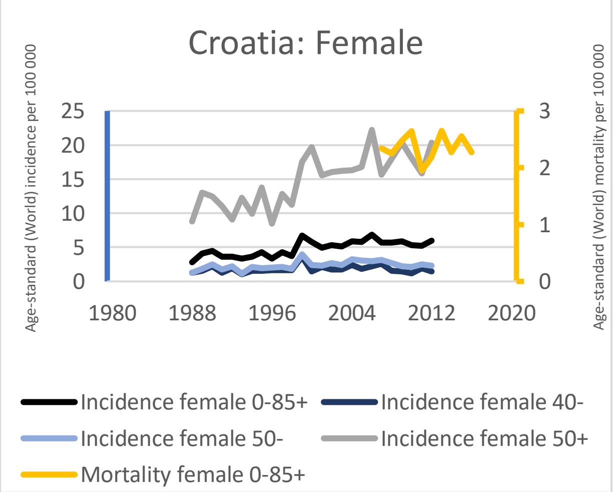

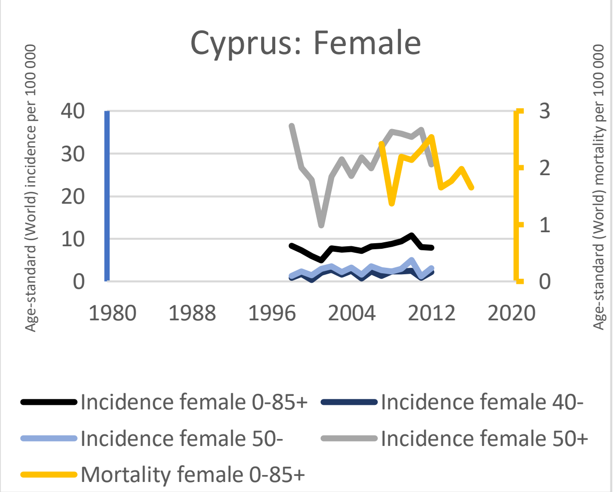

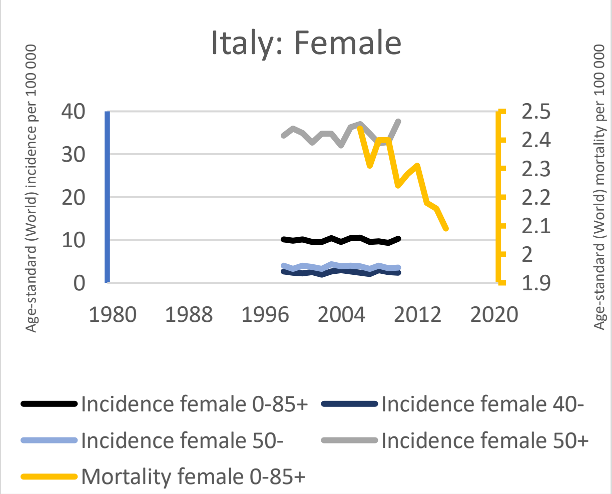

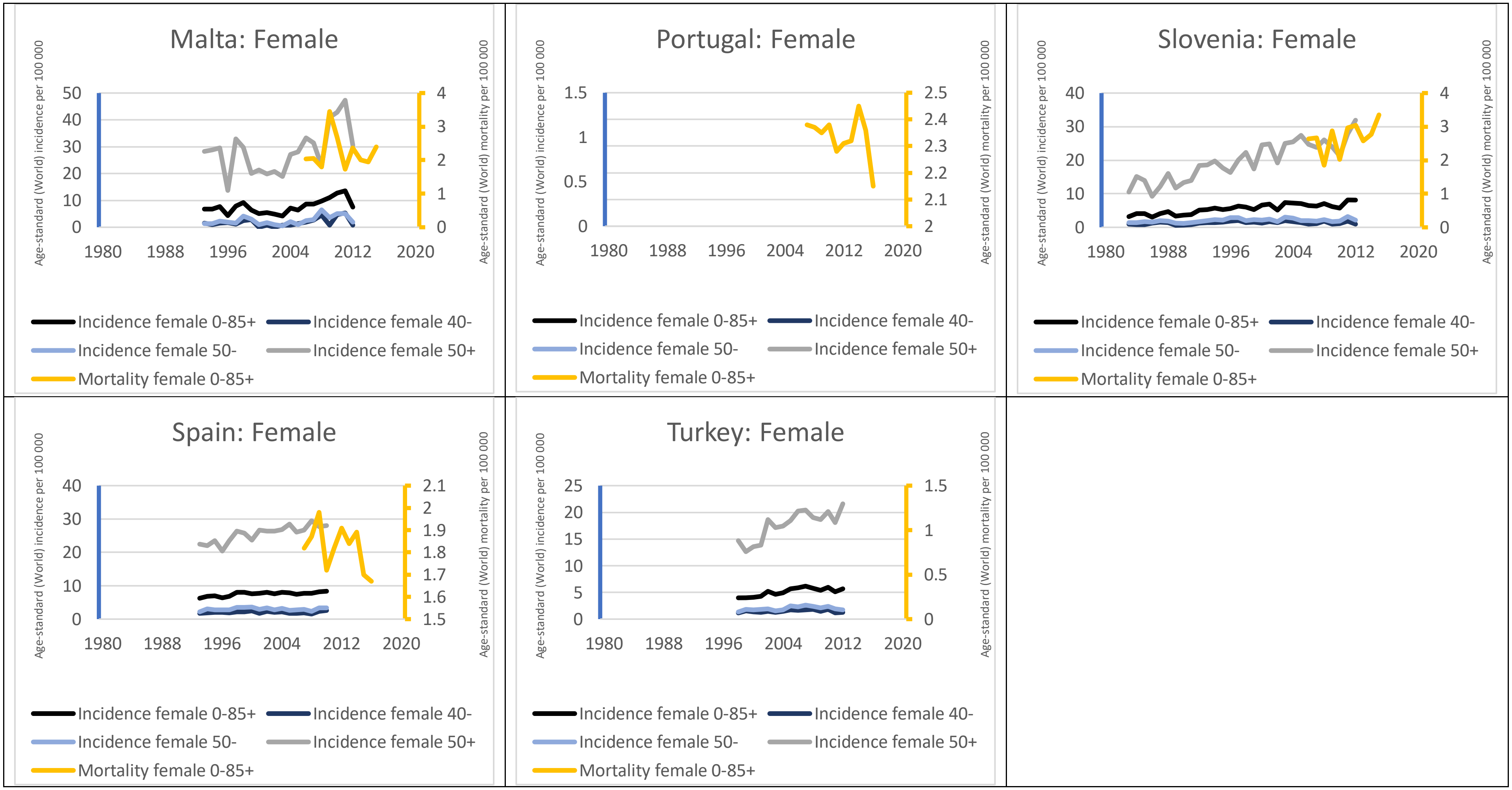
Malta: Female
Age-standard (World) incidence per 100 000
Age-standard (World) mortality per 100 000
0
10
20
30
40
50
0
1
2
3
4
1980
1988
1996
2004
2012
2020
Incidence female 0-85+
Incidence female 40-
Incidence female 50-
Incidence female 50+
Mortality female 0-85+
Portugal: Female
Age-standard (World) incidence per 100 000
Age-standard (World) mortality per 100 000
0
0.5
1
1.5
2
2.1
2.2
2.3
2.4
2.5
1980
1988
1996
2004
2012
2020
Incidence female 0-85+
Incidence female 40-
Incidence female 50-
Incidence female 50+
Mortality female 0-85+
Slovenia: Female
Age-standard (World) incidence per 100 000
Age-standard (World) mortality per 100 000
0
10
20
30
40
0
1
2
3
4
1980
1988
1996
2004
2012
2020
Incidence female 0-85+
Incidence female 40-
Incidence female 50-
Incidence female 50+
Mortality female 0-85+
Spain: Female
Age-standard (World) incidence per 100 000
Age-standard (World) mortality per 100 000
0
10
20
30
40
1.5
1.6
1.7
1.8
1.9
2
2.1
1980
1988
1996
2004
2012
2020
Incidence female 0-85+
Incidence female 40-
Incidence female 50-
Incidence female 50+
Mortality female 0-85+
Turkey: Female
Age-standard (World) incidence per 100 000
Age-standard (World) mortality per 100 000
0
5
10
15
20
25
0
0.5
1
1.5
1980
1988
1996
2004
2012
2020
Incidence female 0-85+
Incidence female 40-
Incidence female 50-
Incidence female 50+
Mortality female 0-85+

Eastern Europe

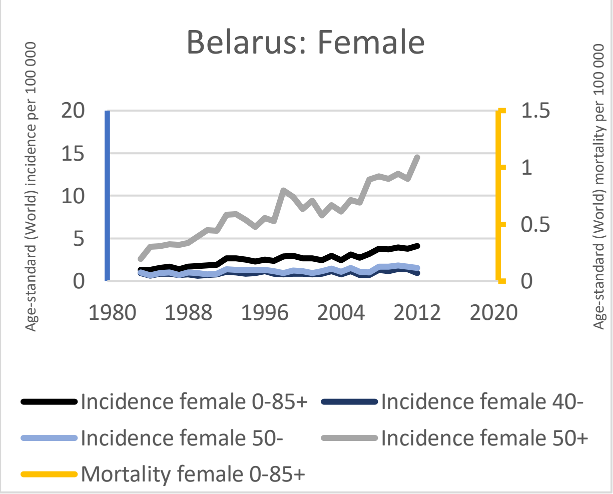

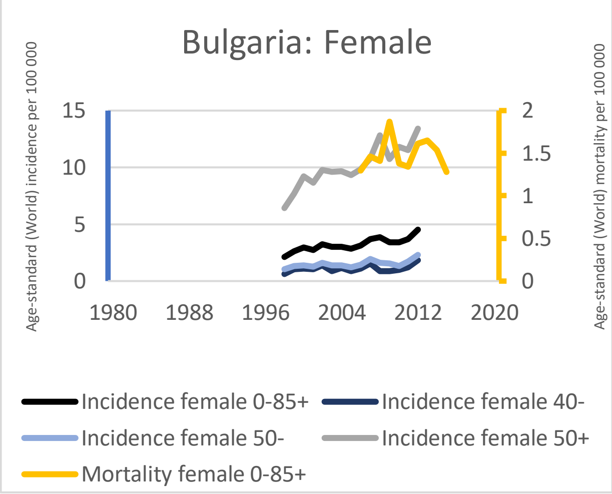

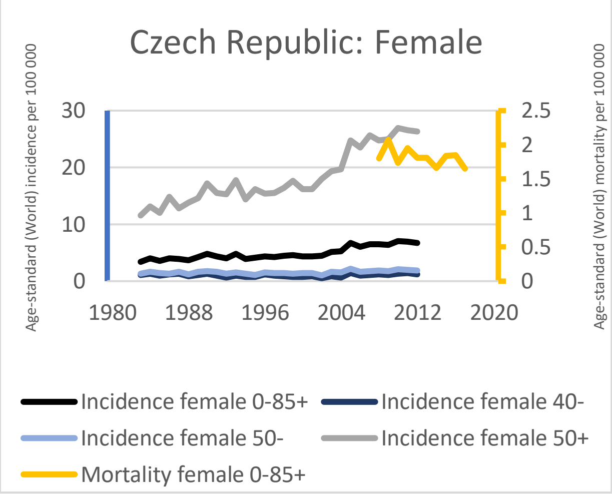

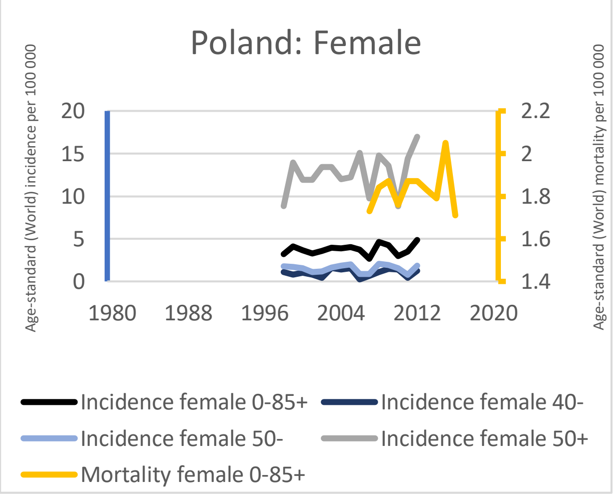

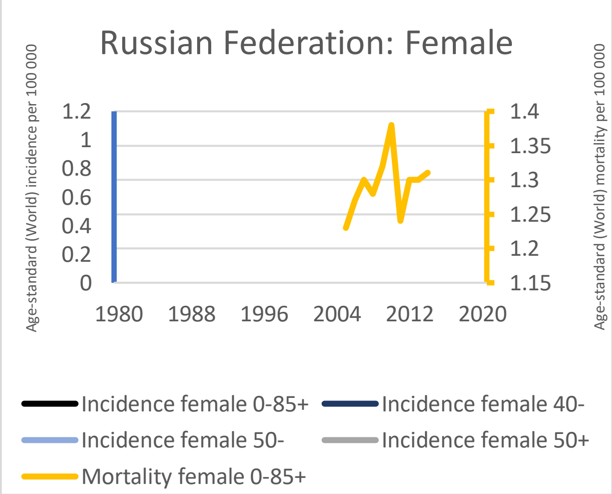

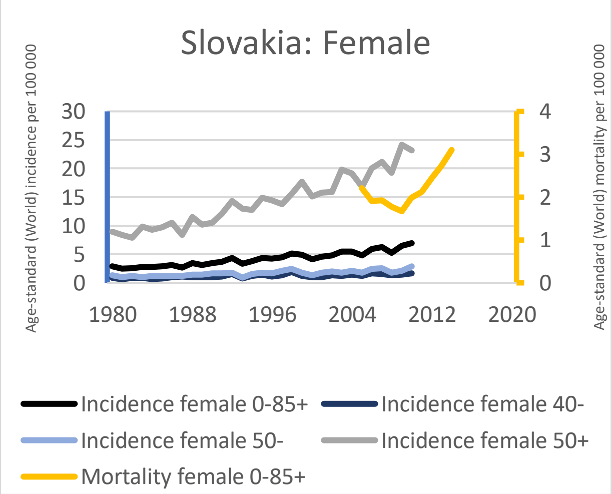

Africa		
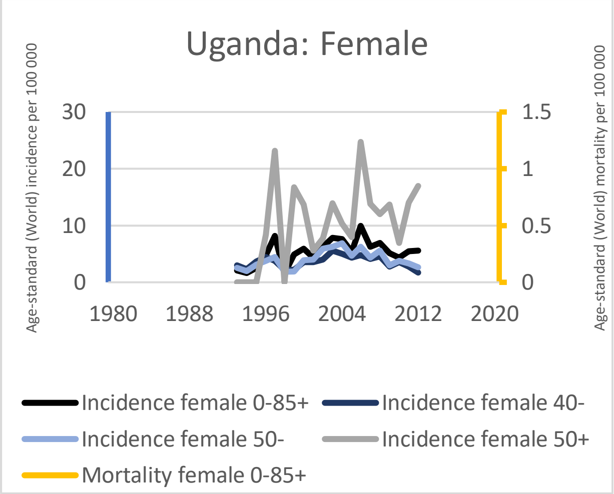<br>Uganda: Female<br>Age-standard (World) incidence per 100 000<br>30<br>20<br>10<br>0<br>1.5<br>1<br>0.5<br>0<br>1980<br>1988<br>1996<br>2004<br>2012<br>2020<br>Age-standard (World) mortality per 100 000<br>Incidence female 0-85+<br>Incidence female 40-<br>Incidence female 50-<br>Incidence female 50+<br>Mortality female 0-85+		

Supplementary Figure 2: The graphs of the joinpoint regression output

a.) Incidence male all ages

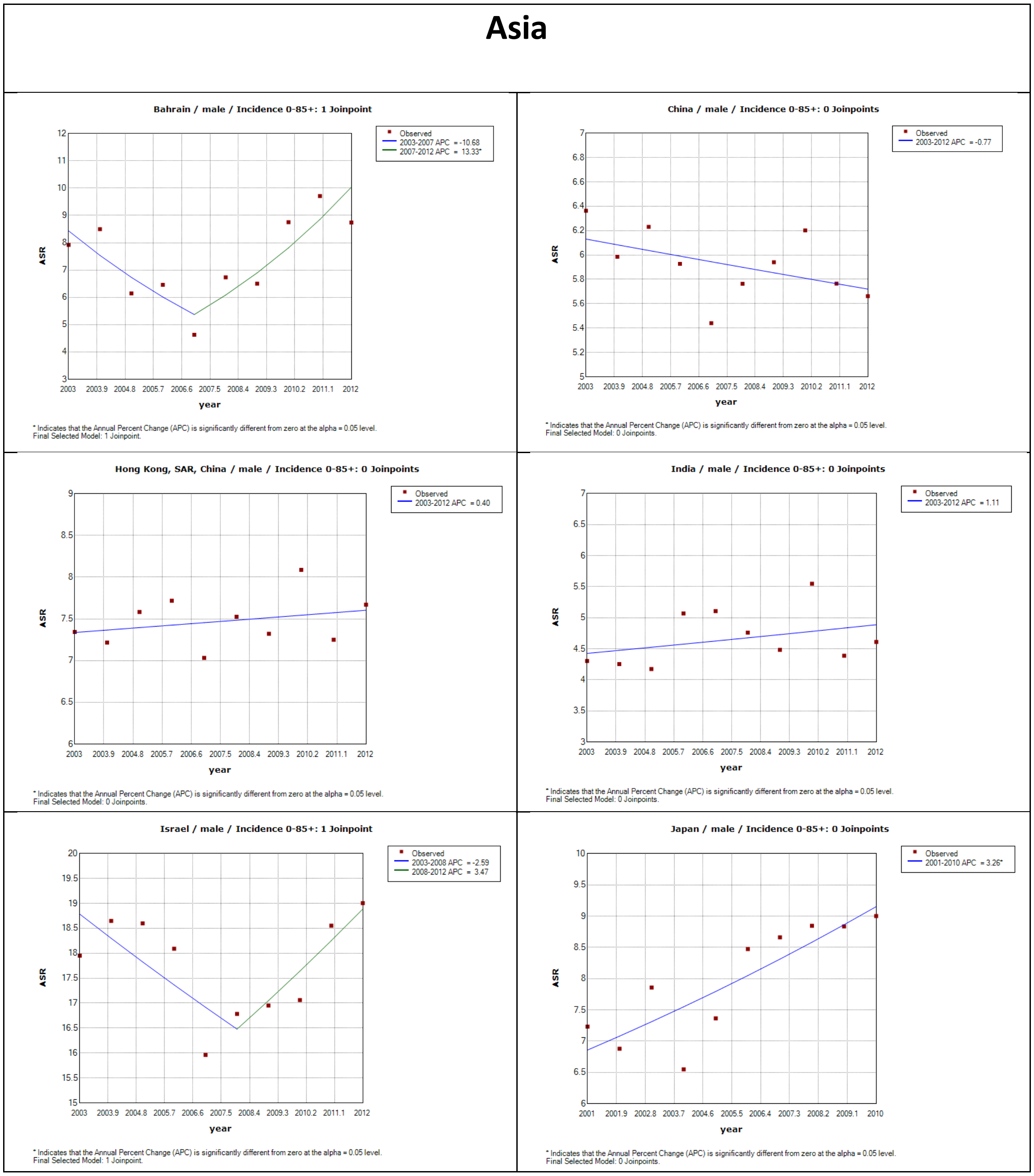

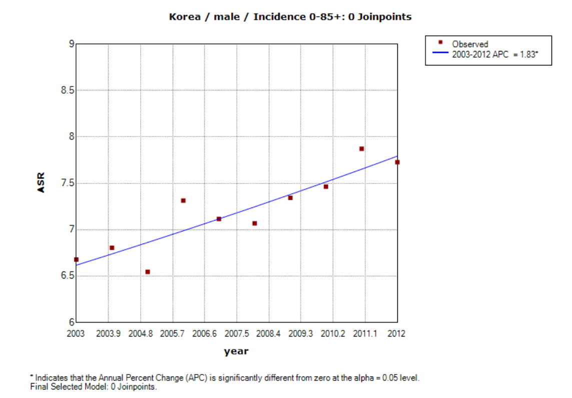
Korea / male / Incidence 0-85+: 0 Joinpoints
Observed
2003-2012 APC = 1.83*
ASR
year
* Indicates that the Annual Percent Change (APC) is significantly different from zero at the alpha = 0.05 level.
Final Selected Model: 0 Joinpoints.

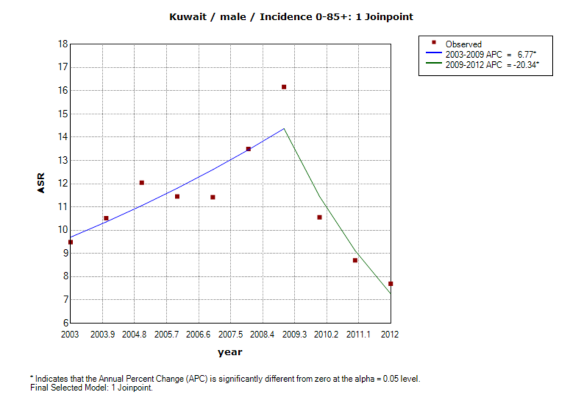
Kuwait / male / Incidence 0-85+: 1 Joinpoint
Observed
2003-2009 APC = 6.77*
2009-2012 APC = -20.34*
ASR
year
* Indicates that the Annual Percent Change (APC) is significantly different from zero at the alpha = 0.05 level.
Final Selected Model: 1 Joinpoint.

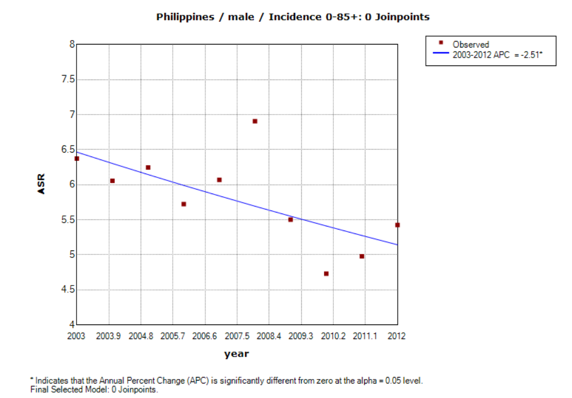
Philippines / male / Incidence 0-85+: 0 Joinpoints
Observed
2003-2012 APC = -2.51*
ASR
year
* Indicates that the Annual Percent Change (APC) is significantly different from zero at the alpha = 0.05 level.
Final Selected Model: 0 Joinpoints.

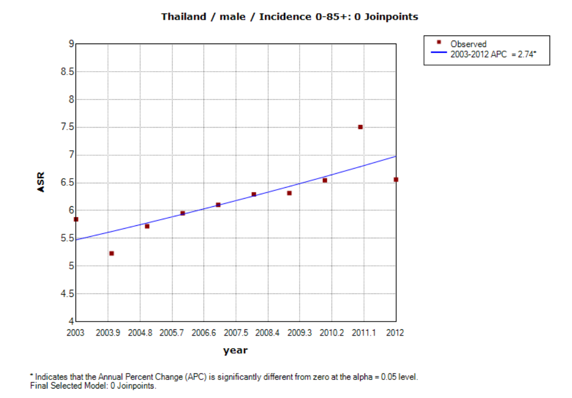
Thailand / male / Incidence 0-85+: 0 Joinpoints
Observed
2003-2012 APC = 2.74*
ASR
year
* Indicates that the Annual Percent Change (APC) is significantly different from zero at the alpha = 0.05 level.
Final Selected Model: 0 Joinpoints.

Oceania

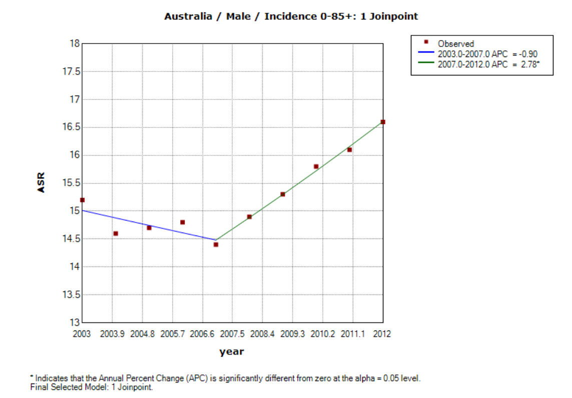
Australia / Male / Incidence 0-85+: 1 Joinpoint
Observed
2003.0-2007.0 APC = -0.90
2007.0-2012.0 APC = 2.78*
ASR
year
* Indicates that the Annual Percent Change (APC) is significantly different from zero at the alpha = 0.05 level.
Final Selected Model: 1 Joinpoint.

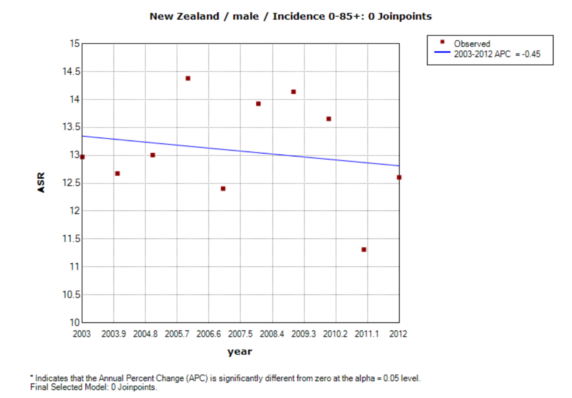
New Zealand / male / Incidence 0-85+: 0 Joinpoints
Observed
2003-2012 APC = -0.45
ASR
year
* Indicates that the Annual Percent Change (APC) is significantly different from zero at the alpha = 0.05 level.
Final Selected Model: 0 Joinpoints.

Northern America

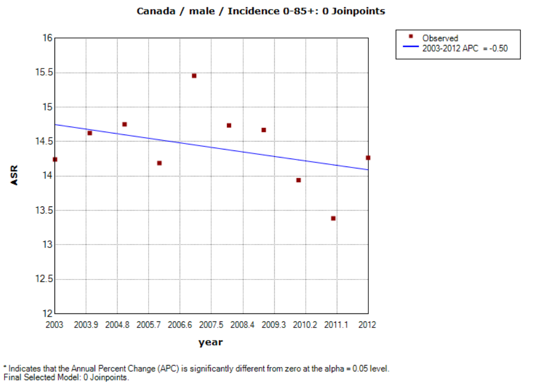

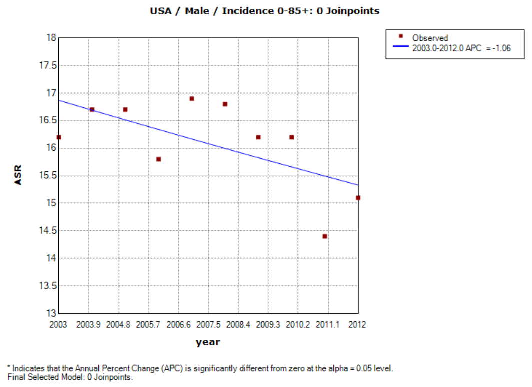

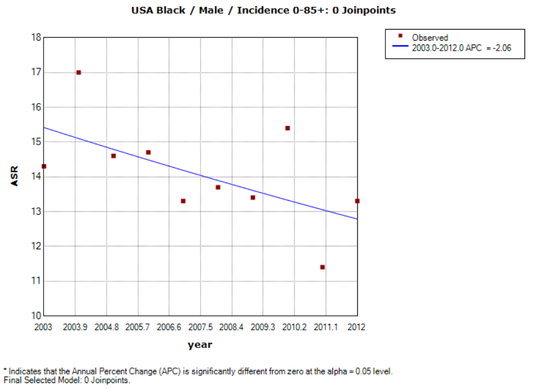

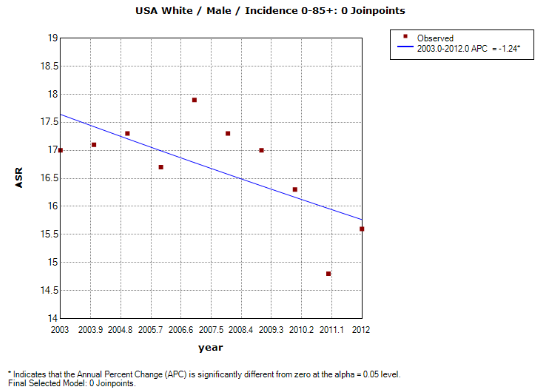

Southern America

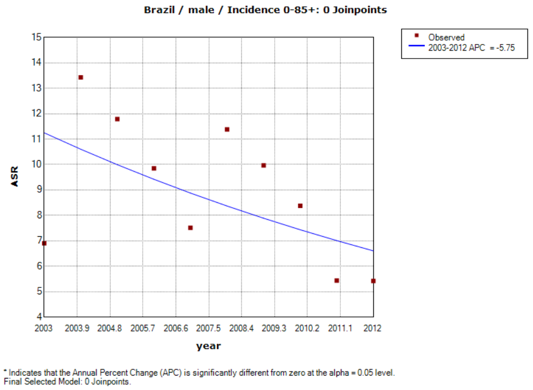

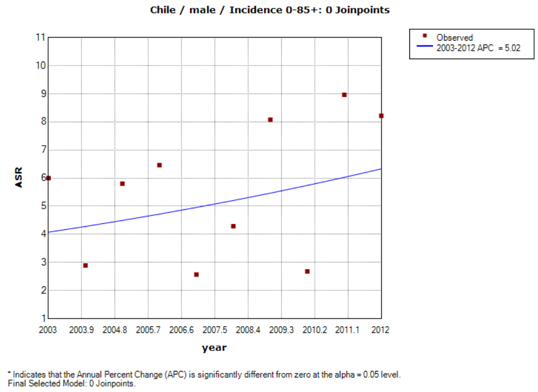

Colombia / male / Incidence 0-85+: 0 Joinpoints

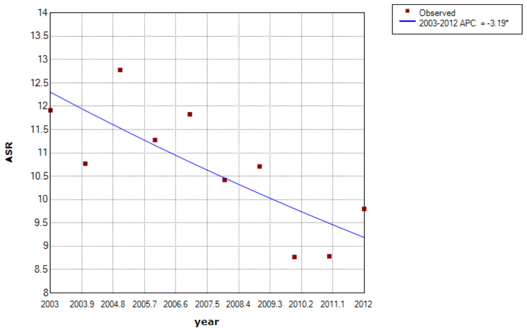

* Indicates that the Annual Percent Change (APC) is significantly different from zero at the alpha = 0.05 level.
Final Selected Model: 0 Joinpoints.

Costa Rica / male / Incidence 0-85+: 0 Joinpoints

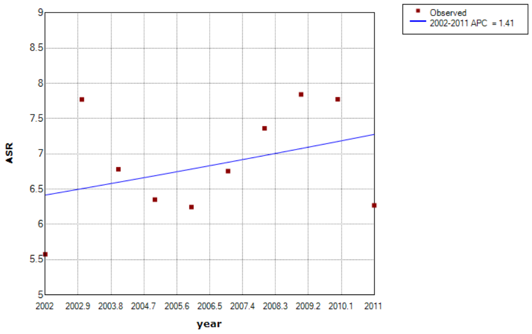

* Indicates that the Annual Percent Change (APC) is significantly different from zero at the alpha = 0.05 level.
Final Selected Model: 0 Joinpoints.

Ecuador / male / Incidence 0-85+: 0 Joinpoints

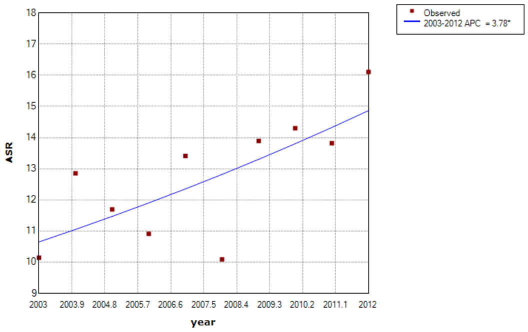

* Indicates that the Annual Percent Change (APC) is significantly different from zero at the alpha = 0.05 level.
Final Selected Model: 0 Joinpoints.

Northern Europe

Denmark / male / Incidence 0-85+: 1 Joinpoint

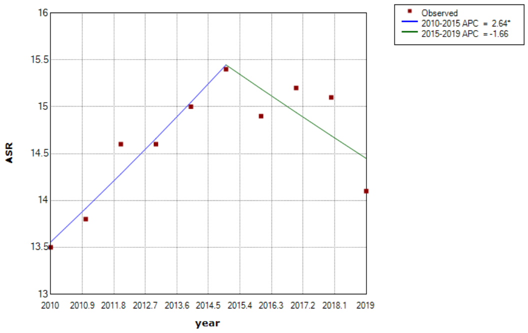

* Indicates that the Annual Percent Change (APC) is significantly different from zero at the alpha = 0.05 level.
Final Selected Model: 1 Joinpoint.

Estonia / male / Incidence 0-85+: 0 Joinpoints

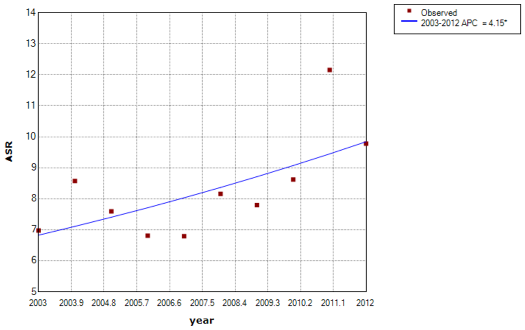

* Indicates that the Annual Percent Change (APC) is significantly different from zero at the alpha = 0.05 level.
Final Selected Model: 0 Joinpoints.

Faroe Islands / male / Incidence 0-85+: 0 Joinpoints

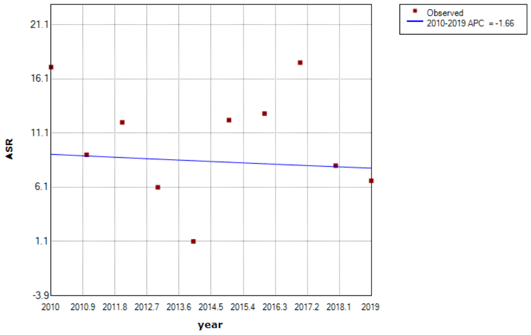

* Indicates that the Annual Percent Change (APC) is significantly different from zero at the alpha = 0.05 level.
Final Selected Model: 0 Joinpoints.

Finland / male / Incidence 0-85+: 0 Joinpoints

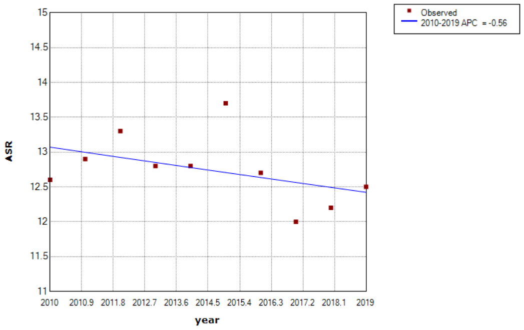

* Indicates that the Annual Percent Change (APC) is significantly different from zero at the alpha = 0.05 level.
Final Selected Model: 0 Joinpoints.

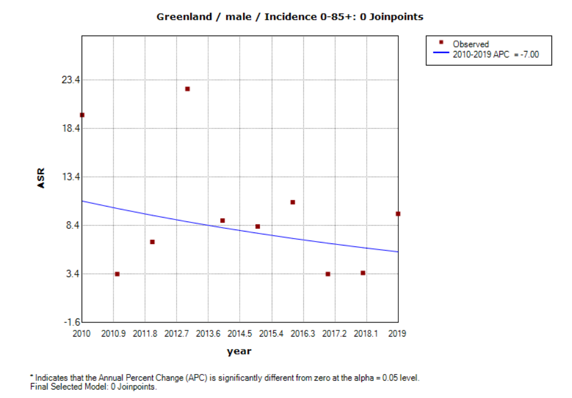

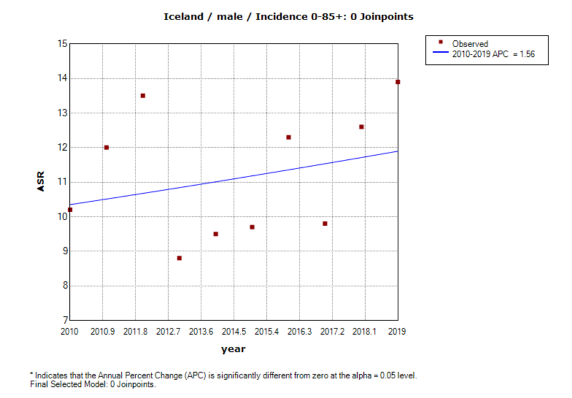

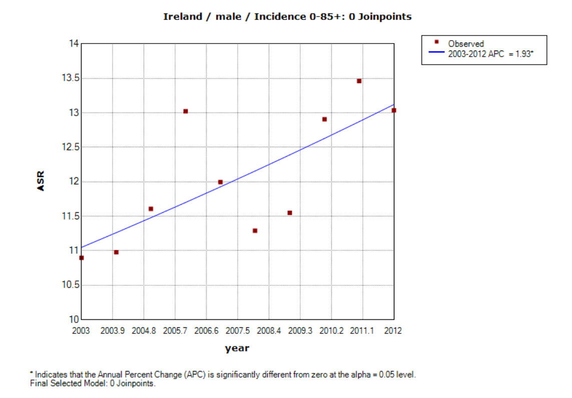

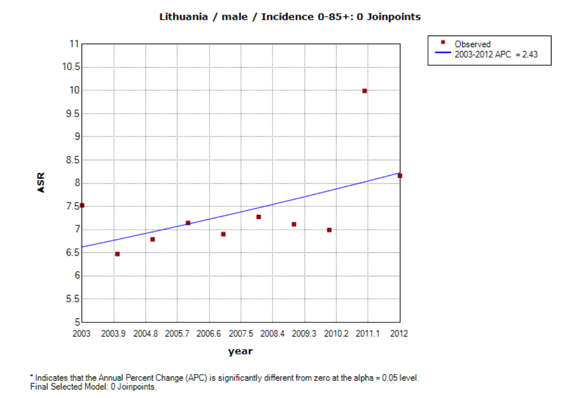

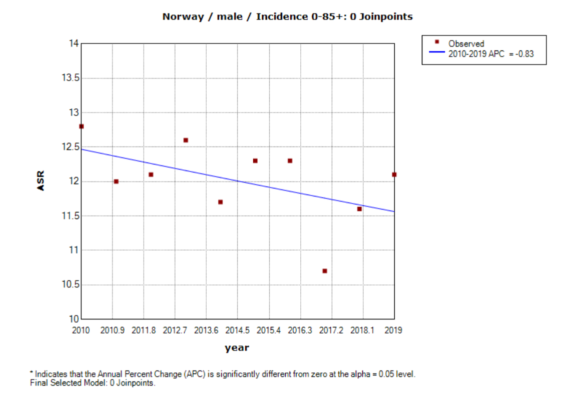

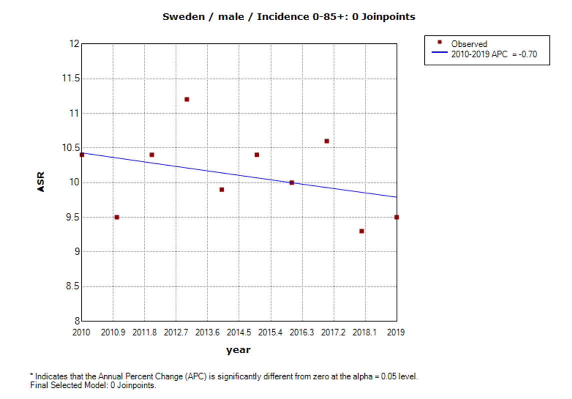

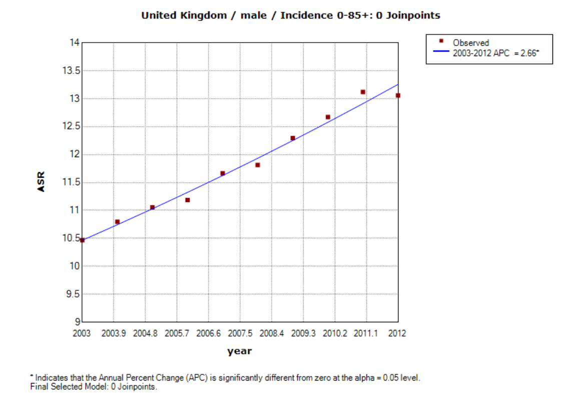

Western Europe

Austria / male / Incidence 0-85+: 0 Joinpoints

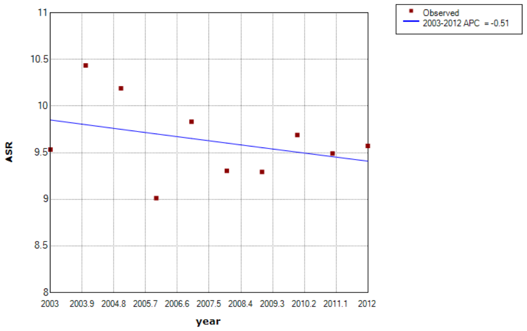

* Indicates that the Annual Percent Change (APC) is significantly different from zero at the alpha = 0.05 level.
Final Selected Model: 0 Joinpoints.

France / male / Incidence 0-85+: 0 Joinpoints

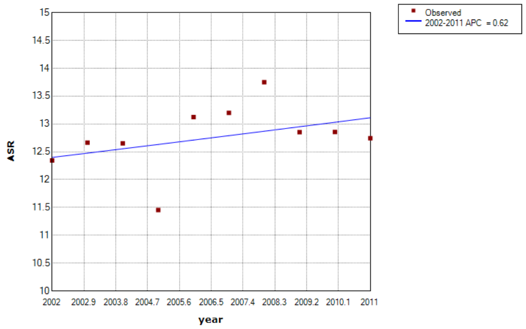

* Indicates that the Annual Percent Change (APC) is significantly different from zero at the alpha = 0.05 level.
Final Selected Model: 0 Joinpoints.

Germany / male / Incidence 0-85+: 0 Joinpoints

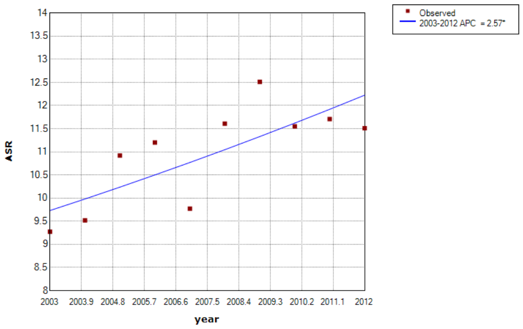

* Indicates that the Annual Percent Change (APC) is significantly different from zero at the alpha = 0.05 level.
Final Selected Model: 0 Joinpoints.

Netherlands / male / Incidence 0-85+: 0 Joinpoints

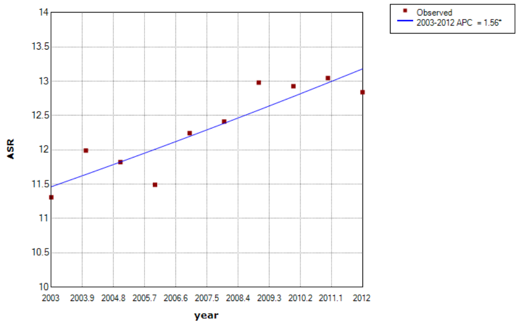

* Indicates that the Annual Percent Change (APC) is significantly different from zero at the alpha = 0.05 level.
Final Selected Model: 0 Joinpoints.

Switzerland / male / Incidence 0-85+: 0 Joinpoints

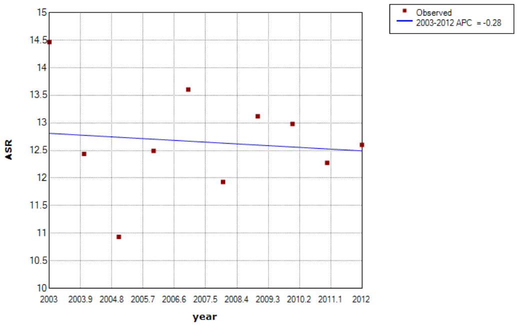

* Indicates that the Annual Percent Change (APC) is significantly different from zero at the alpha = 0.05 level.
Final Selected Model: 0 Joinpoints.

Southern Europe

Croatia / male / Incidence 0-85+: 0 Joinpoints

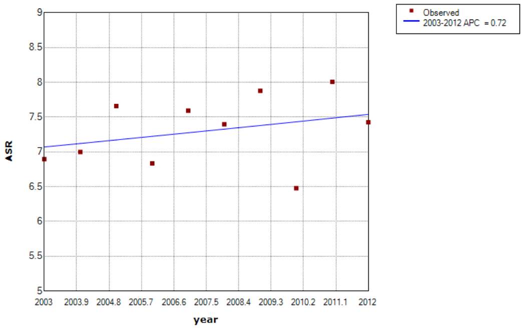

* Indicates that the Annual Percent Change (APC) is significantly different from zero at the alpha = 0.05 level.
Final Selected Model: 0 Joinpoints.

Cyprus / male / Incidence 0-85+: 0 Joinpoints

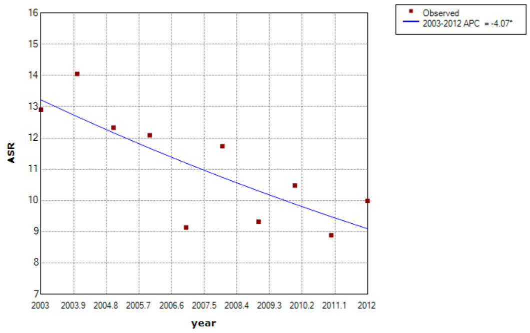

* Indicates that the Annual Percent Change (APC) is significantly different from zero at the alpha = 0.05 level.
Final Selected Model: 0 Joinpoints.

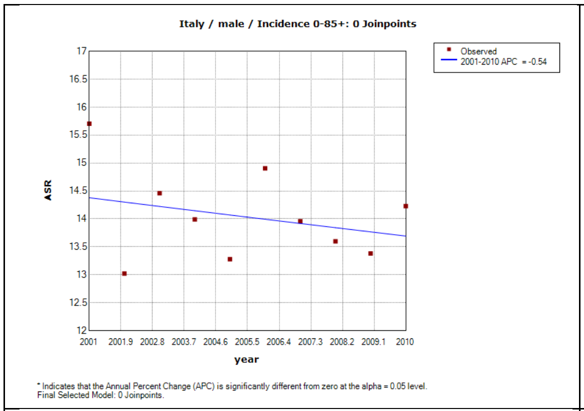

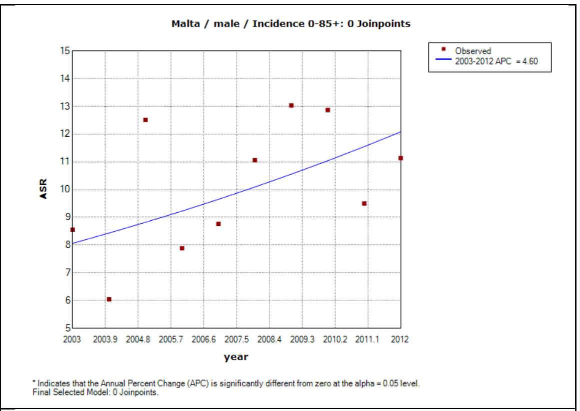

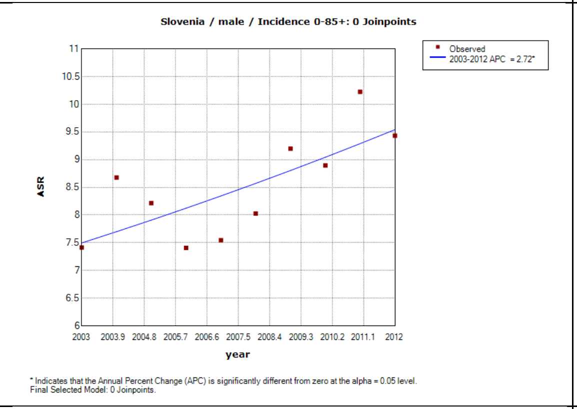

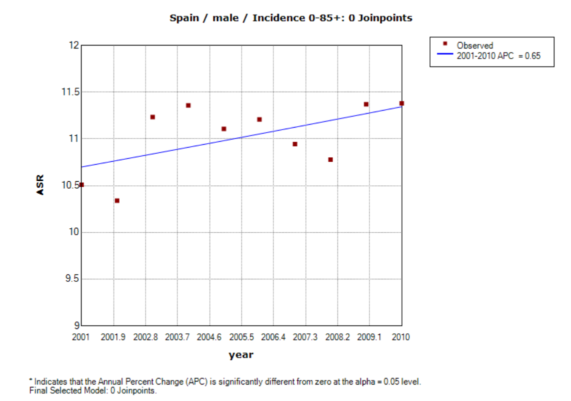

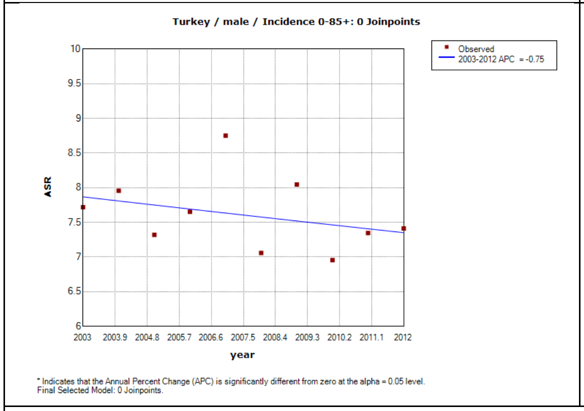

Eastern Europe

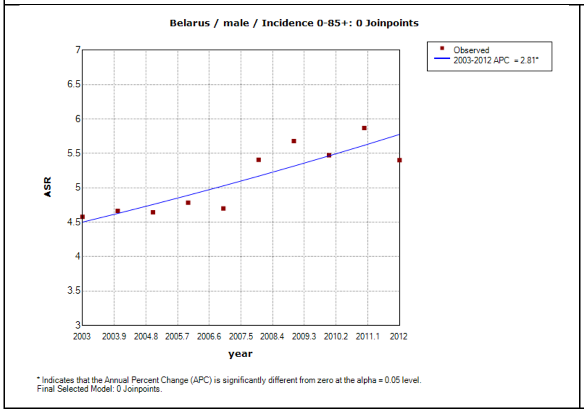

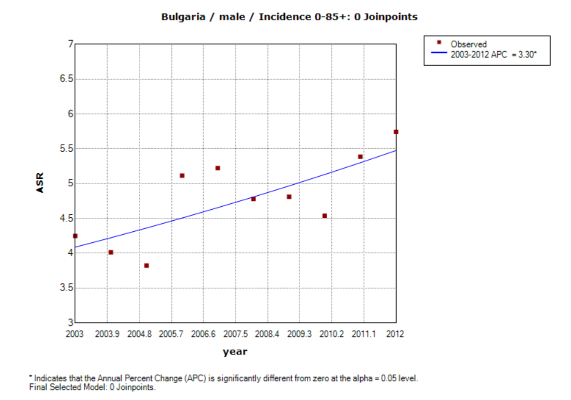

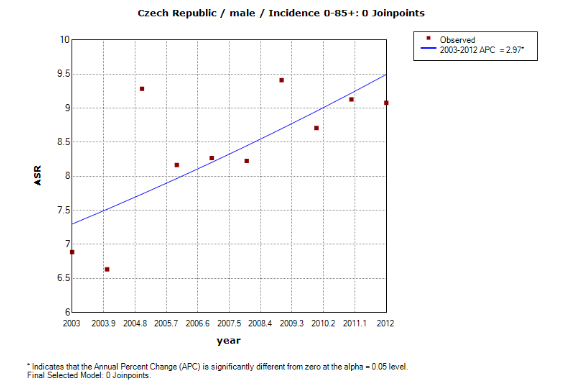
Czech Republic / male / Incidence 0-85+: 0 Joinpoints
Observed
2003-2012 APC = 2.97*
ASR
year
* Indicates that the Annual Percent Change (APC) is significantly different from zero at the alpha = 0.05 level.
Final Selected Model: 0 Joinpoints.

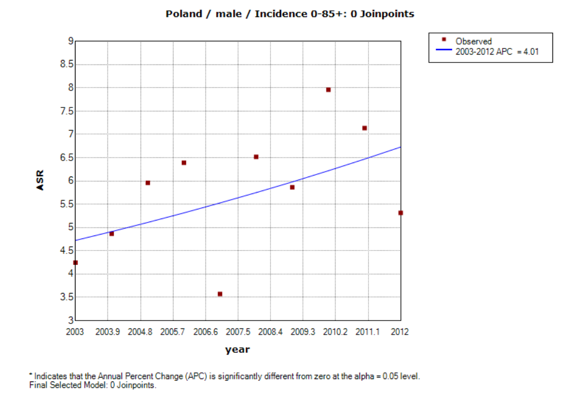
Poland / male / Incidence 0-85+: 0 Joinpoints
Observed
2003-2012 APC = 4.01
ASR
year
* Indicates that the Annual Percent Change (APC) is significantly different from zero at the alpha = 0.05 level.
Final Selected Model: 0 Joinpoints.

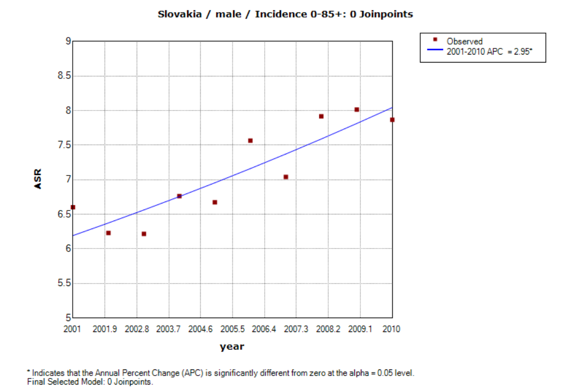
Slovakia / male / Incidence 0-85+: 0 Joinpoints
Observed
2001-2010 APC = 2.95*
ASR
year
* Indicates that the Annual Percent Change (APC) is significantly different from zero at the alpha = 0.05 level.
Final Selected Model: 0 Joinpoints.

Africa

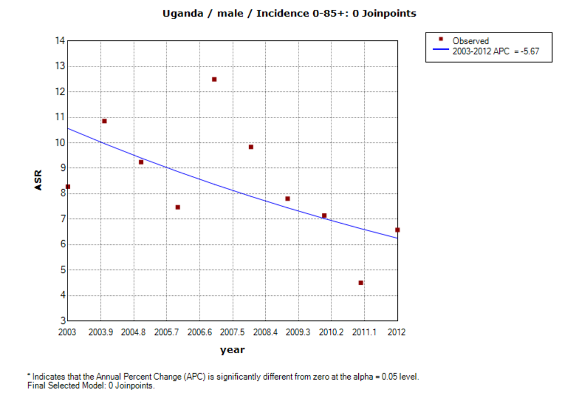
Uganda / male / Incidence 0-85+: 0 Joinpoints
Observed
2003-2012 APC = -5.67
ASR
year
* Indicates that the Annual Percent Change (APC) is significantly different from zero at the alpha = 0.05 level.
Final Selected Model: 0 Joinpoints.

b.) Incidence female all ages

Asia

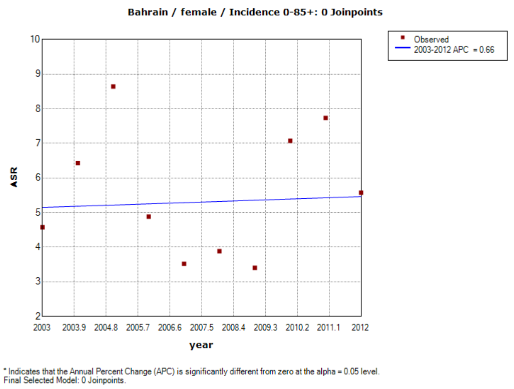

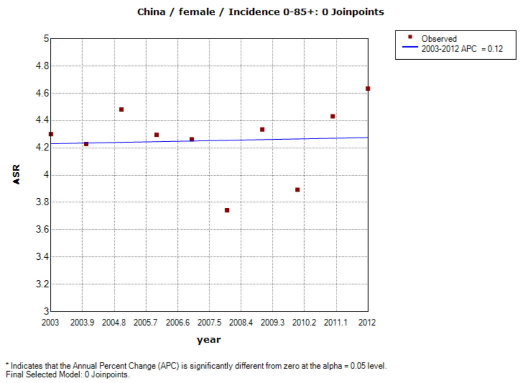

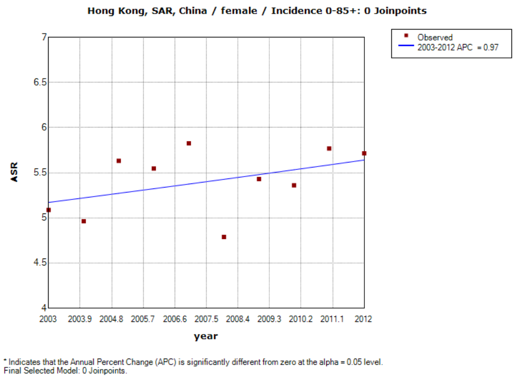

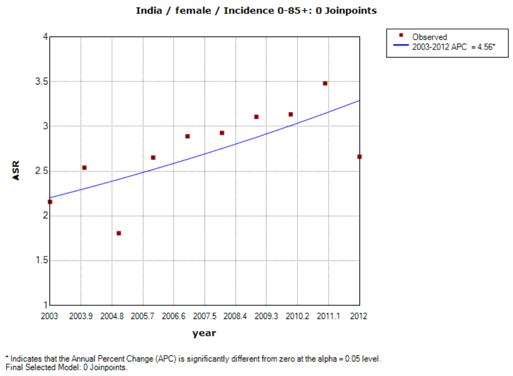

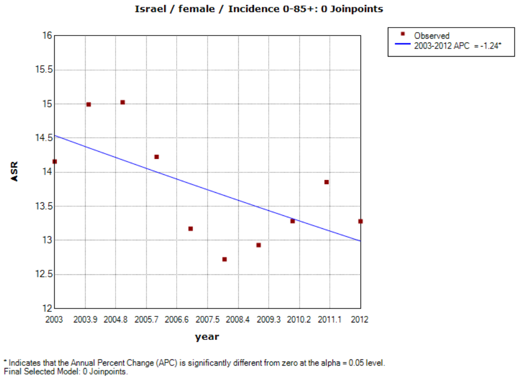

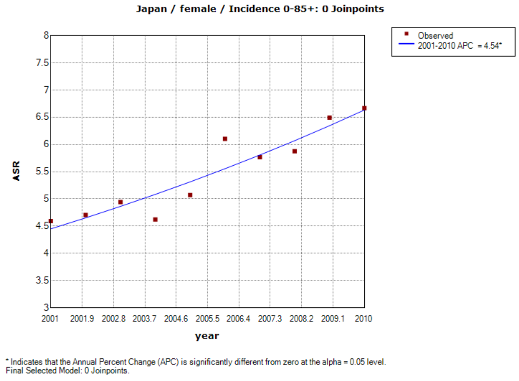

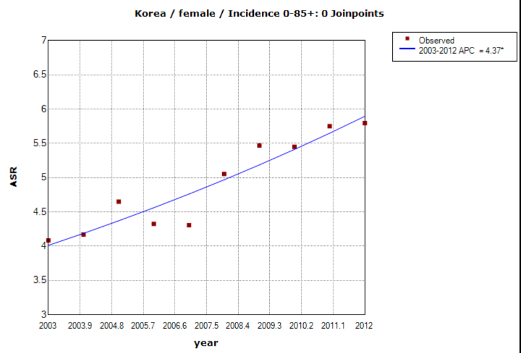

* Indicates that the Annual Percent Change (APC) is significantly different from zero at the alpha = 0.05 level.
Final Selected Model: 0 Joinpoints.

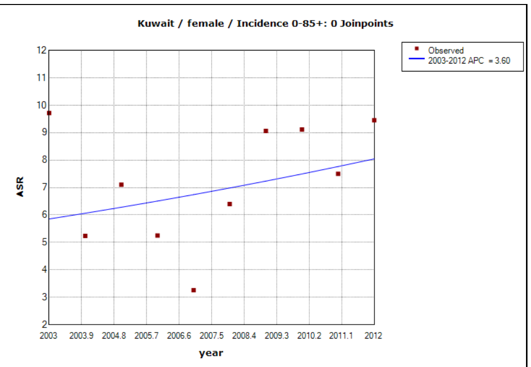

* Indicates that the Annual Percent Change (APC) is significantly different from zero at the alpha = 0.05 level.
Final Selected Model: 0 Joinpoints.

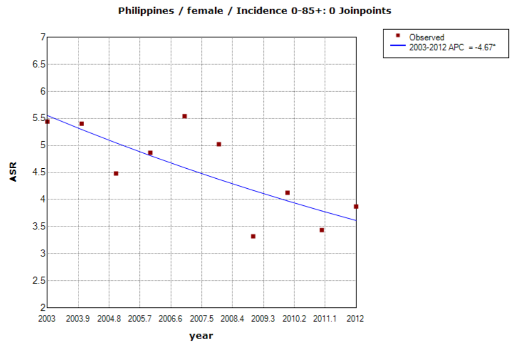

* Indicates that the Annual Percent Change (APC) is significantly different from zero at the alpha = 0.05 level.
Final Selected Model: 0 Joinpoints.

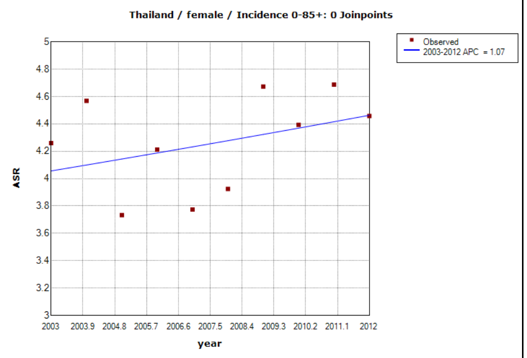

* Indicates that the Annual Percent Change (APC) is significantly different from zero at the alpha = 0.05 level.
Final Selected Model: 0 Joinpoints.

Oceania

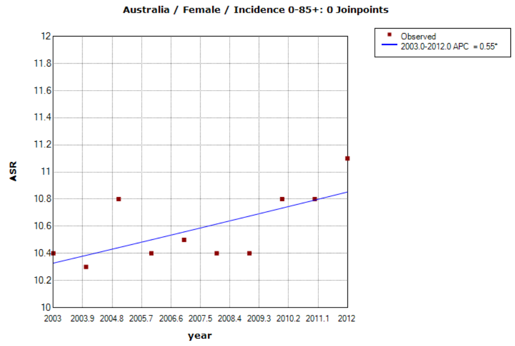

* Indicates that the Annual Percent Change (APC) is significantly different from zero at the alpha = 0.05 level.
Final Selected Model: 0 Joinpoints.

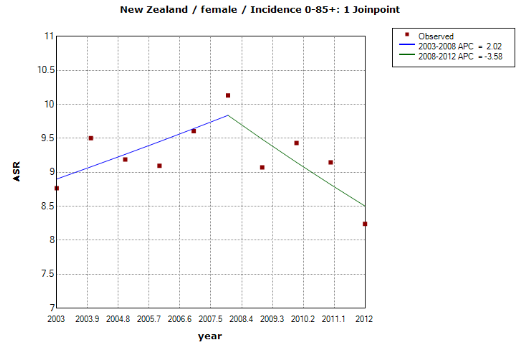

* Indicates that the Annual Percent Change (APC) is significantly different from zero at the alpha = 0.05 level.
Final Selected Model: 1 Joinpoint.

Northern America

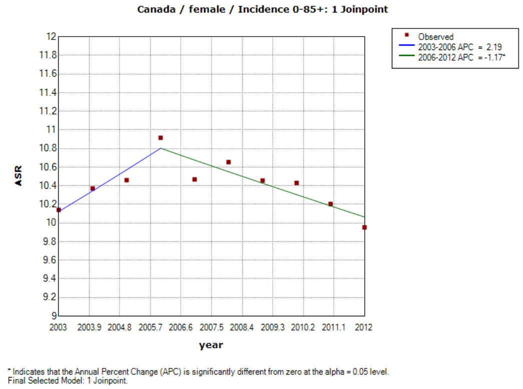

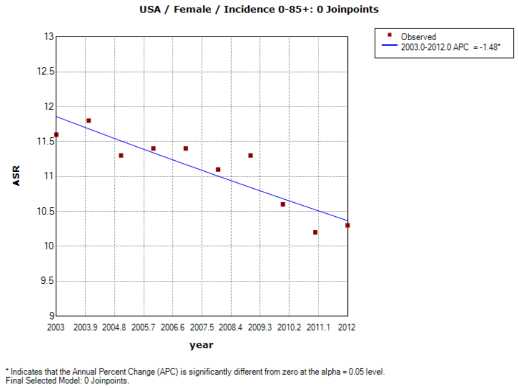

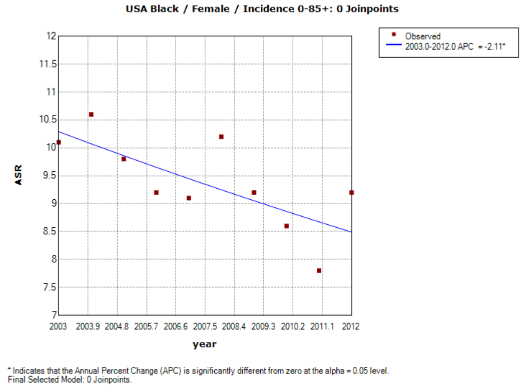

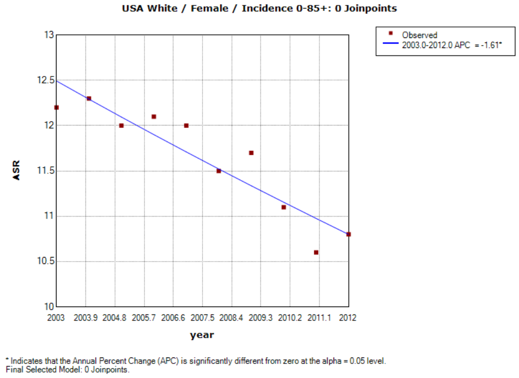

Southern America

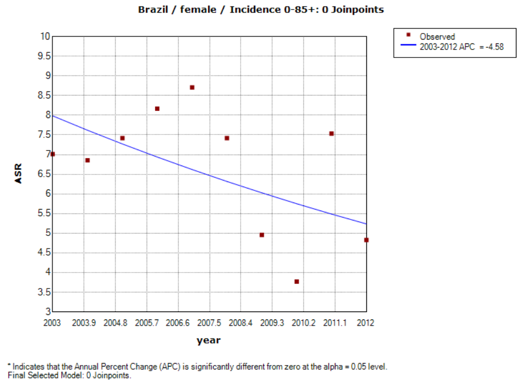

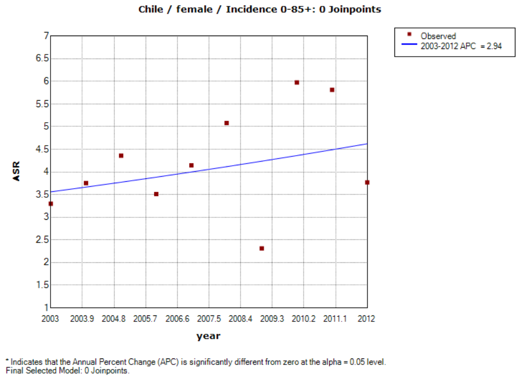

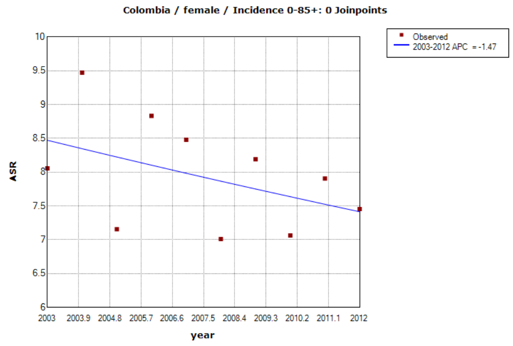

* Indicates that the Annual Percent Change (APC) is significantly different from zero at the alpha = 0.05 level.
Final Selected Model: 0 Joinpoints.

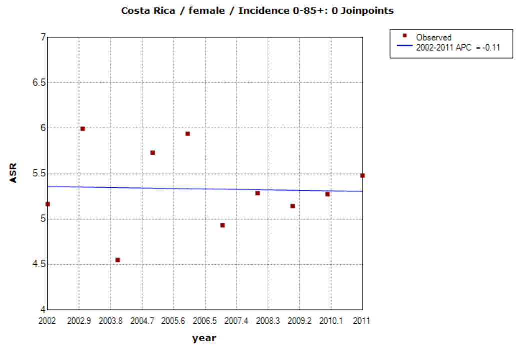

* Indicates that the Annual Percent Change (APC) is significantly different from zero at the alpha = 0.05 level.
Final Selected Model: 0 Joinpoints.

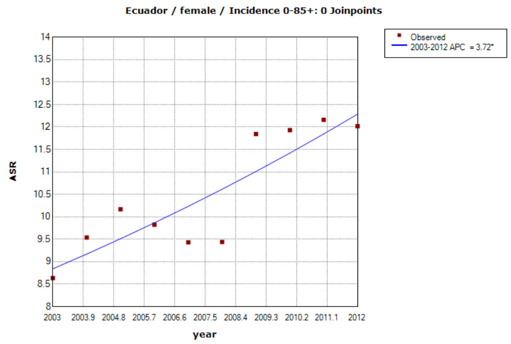

* Indicates that the Annual Percent Change (APC) is significantly different from zero at the alpha = 0.05 level.
Final Selected Model: 0 Joinpoints.

Northern Europe

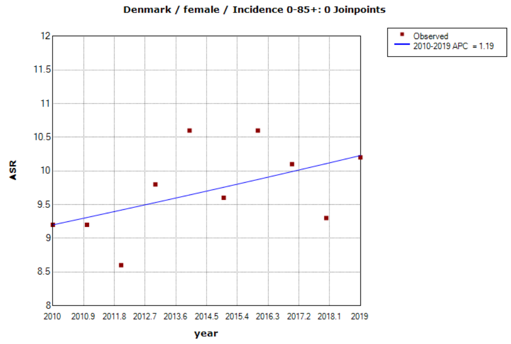

* Indicates that the Annual Percent Change (APC) is significantly different from zero at the alpha = 0.05 level.
Final Selected Model: 0 Joinpoints.

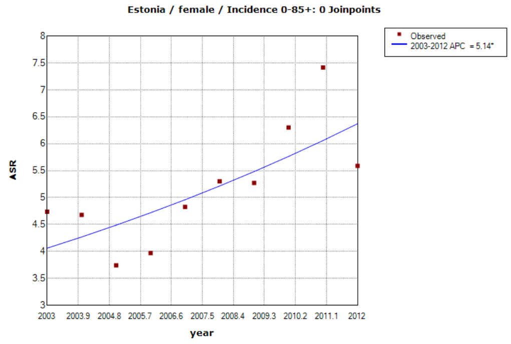

* Indicates that the Annual Percent Change (APC) is significantly different from zero at the alpha = 0.05 level.
Final Selected Model: 0 Joinpoints.

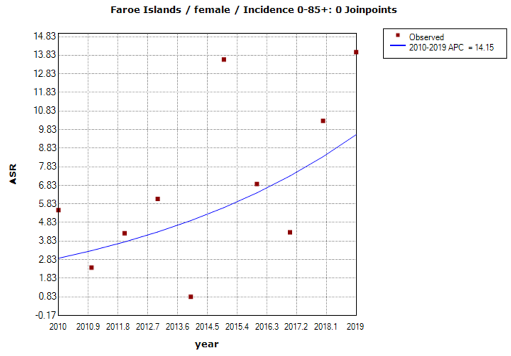

* Indicates that the Annual Percent Change (APC) is significantly different from zero at the alpha = 0.05 level.
Final Selected Model: 0 Joinpoints.

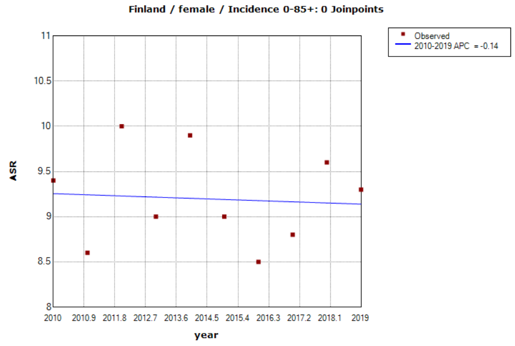

* Indicates that the Annual Percent Change (APC) is significantly different from zero at the alpha = 0.05 level.
Final Selected Model: 0 Joinpoints.

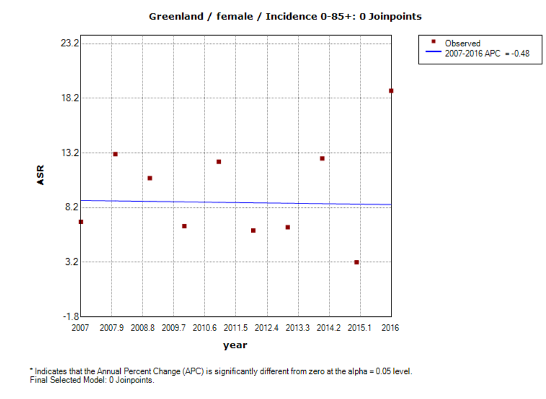

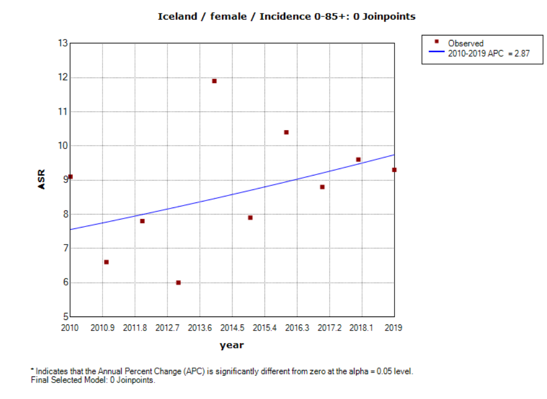

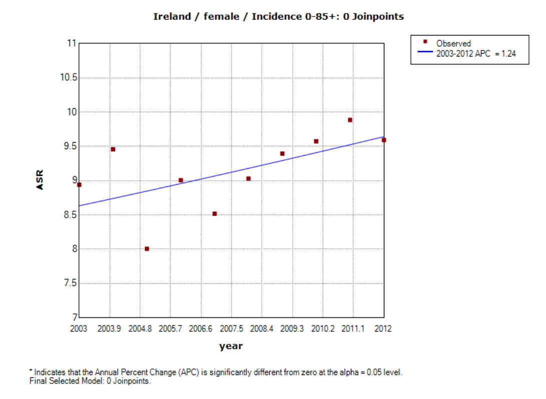

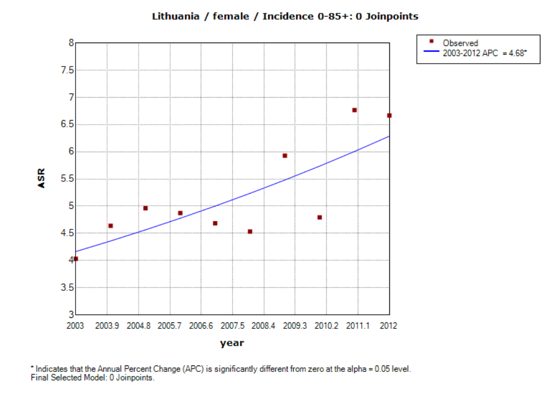

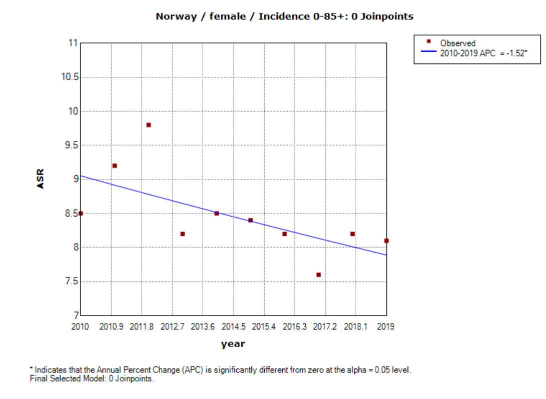

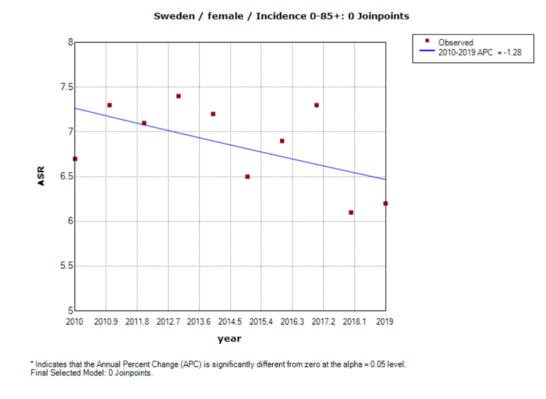

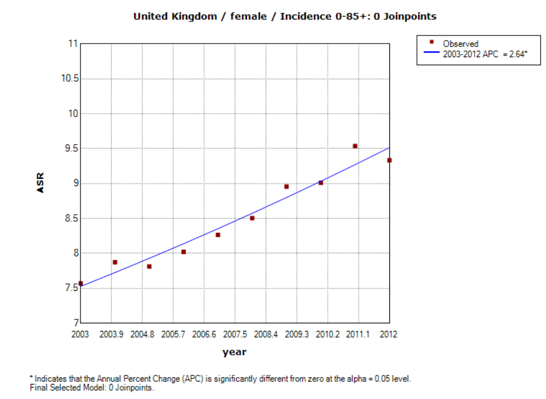

Western Europe

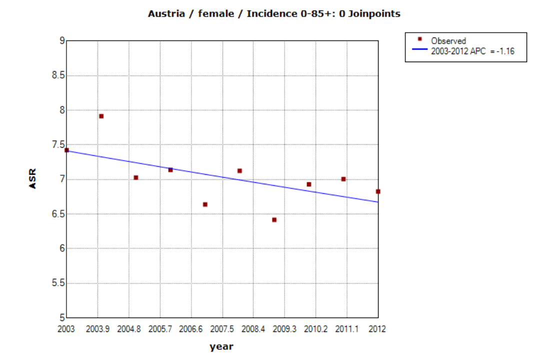

* Indicates that the Annual Percent Change (APC) is significantly different from zero at the alpha = 0.05 level.
Final Selected Model: 0 Joinpoints.

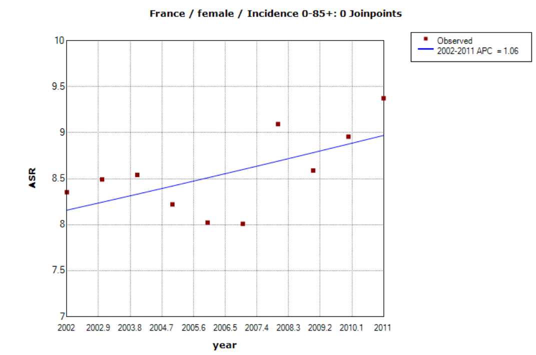

* Indicates that the Annual Percent Change (APC) is significantly different from zero at the alpha = 0.05 level.
Final Selected Model: 0 Joinpoints.

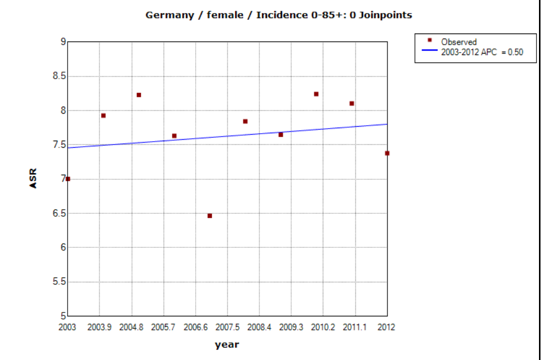

* Indicates that the Annual Percent Change (APC) is significantly different from zero at the alpha = 0.05 level.
Final Selected Model: 0 Joinpoints.

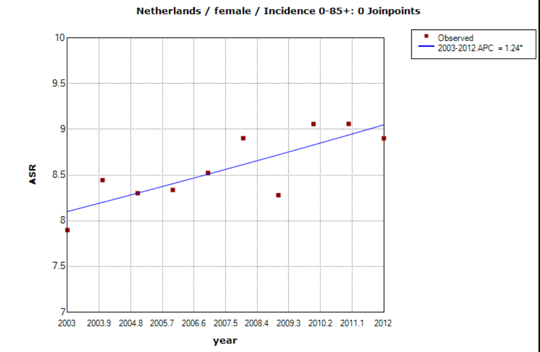

* Indicates that the Annual Percent Change (APC) is significantly different from zero at the alpha = 0.05 level.
Final Selected Model: 0 Joinpoints.

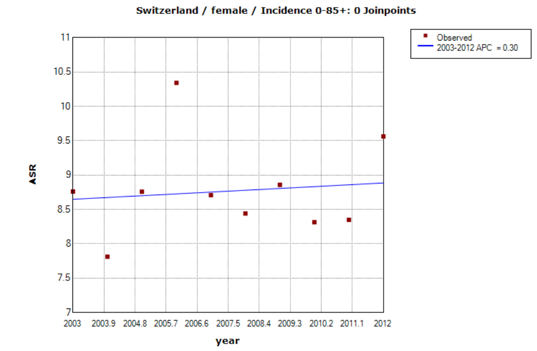

* Indicates that the Annual Percent Change (APC) is significantly different from zero at the alpha = 0.05 level.
Final Selected Model: 0 Joinpoints.

Southern Europe

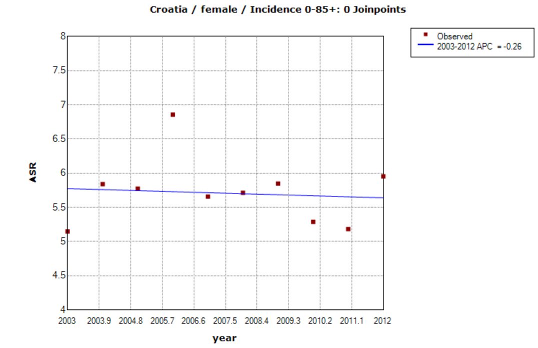

* Indicates that the Annual Percent Change (APC) is significantly different from zero at the alpha = 0.05 level.
Final Selected Model: 0 Joinpoints.

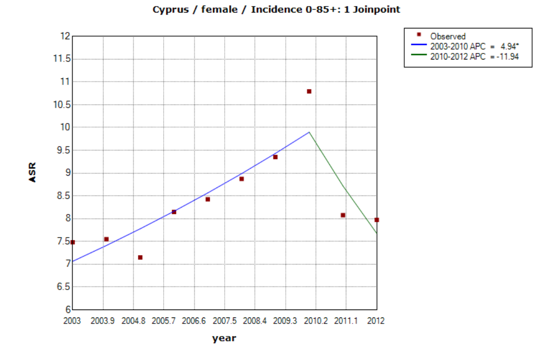

* Indicates that the Annual Percent Change (APC) is significantly different from zero at the alpha = 0.05 level.
Final Selected Model: 1 Joinpoint.

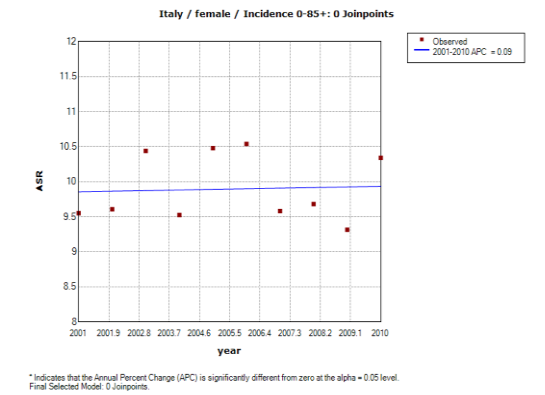

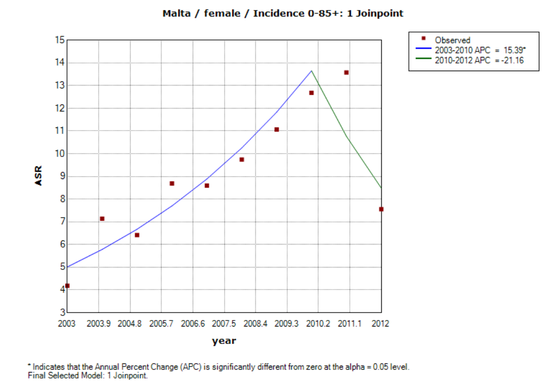

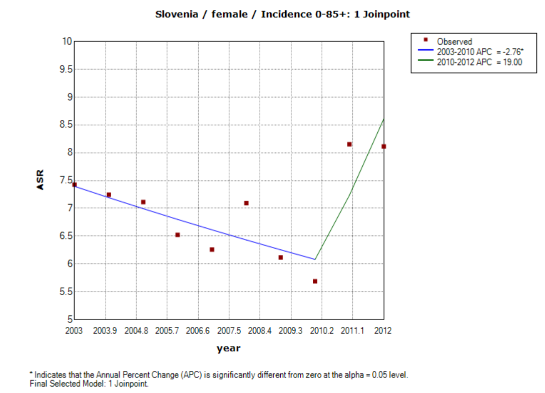

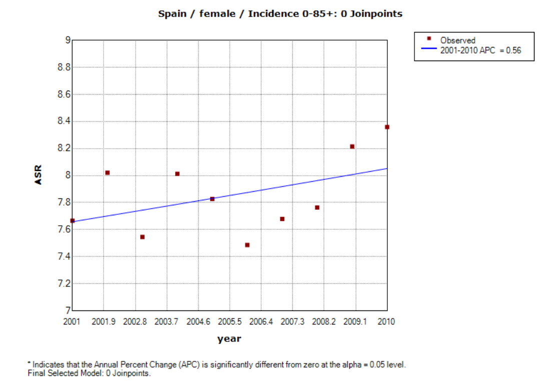

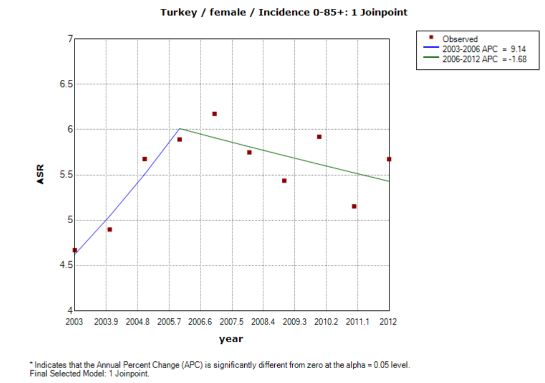

Eastern Europe

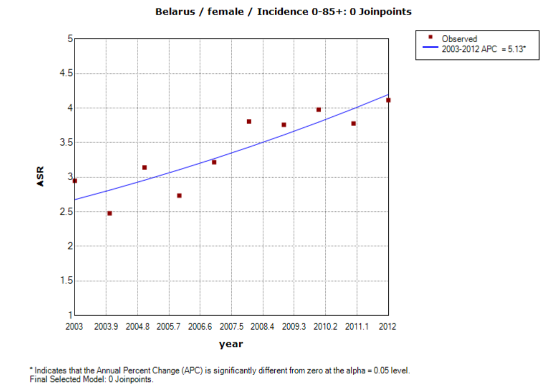

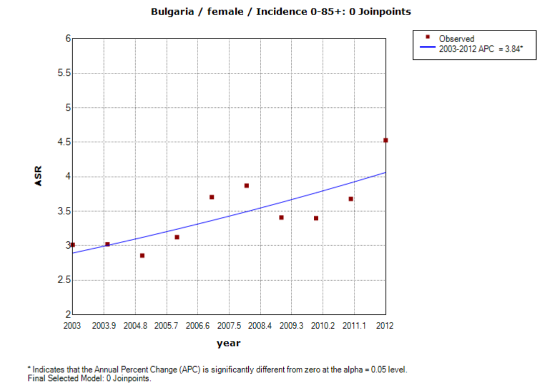

Czech Republic / female / Incidence 0-85+: 0 Joinpoints

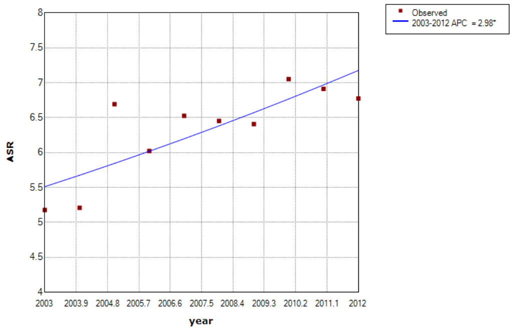

* Indicates that the Annual Percent Change (APC) is significantly different from zero at the alpha = 0.05 level.
Final Selected Model: 0 Joinpoints.

Poland / female / Incidence 0-85+: 0 Joinpoints

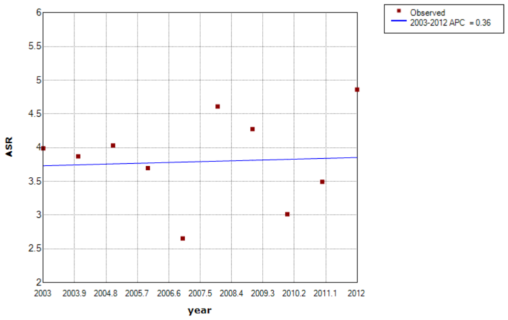

* Indicates that the Annual Percent Change (APC) is significantly different from zero at the alpha = 0.05 level.
Final Selected Model: 0 Joinpoints.

Slovakia / female / Incidence 0-85+: 0 Joinpoints

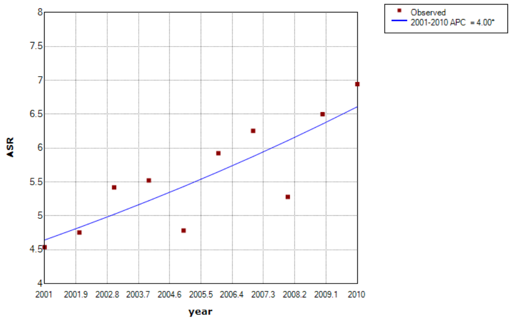

* Indicates that the Annual Percent Change (APC) is significantly different from zero at the alpha = 0.05 level.
Final Selected Model: 0 Joinpoints.

Africa

Uganda / female / Incidence 0-85+: 0 Joinpoints

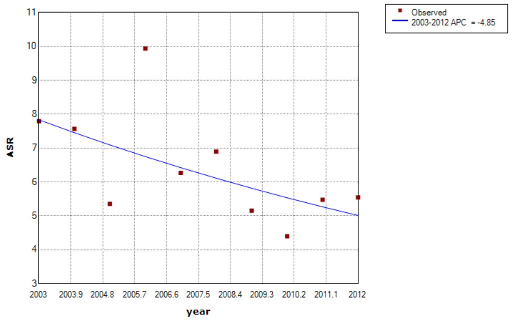

* Indicates that the Annual Percent Change (APC) is significantly different from zero at the alpha = 0.05 level.
Final Selected Model: 0 Joinpoints.

c.) Incidence male below 40 years old

Asia

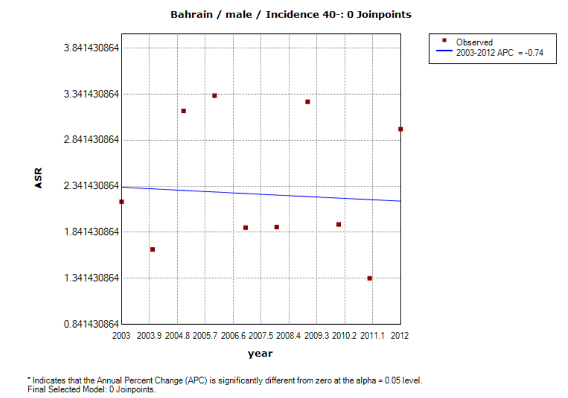

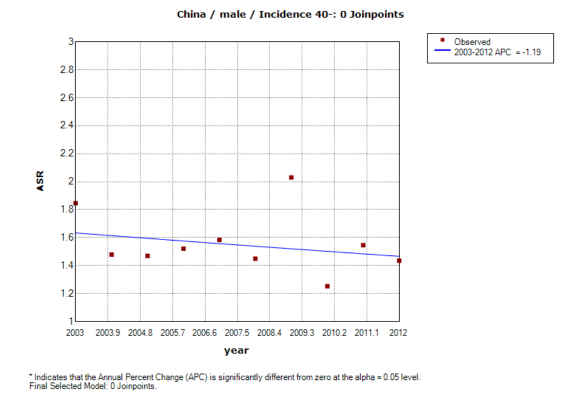

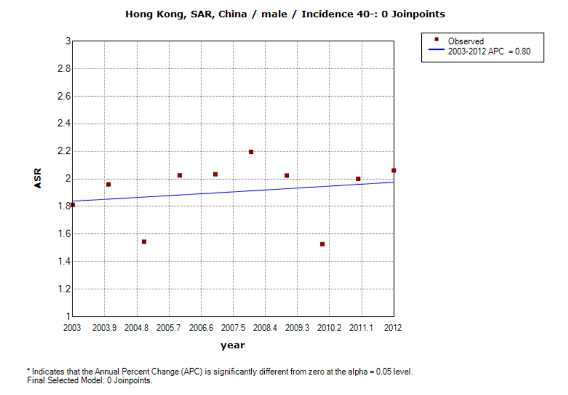

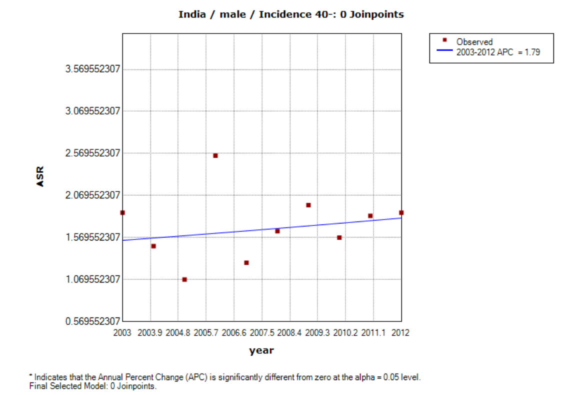

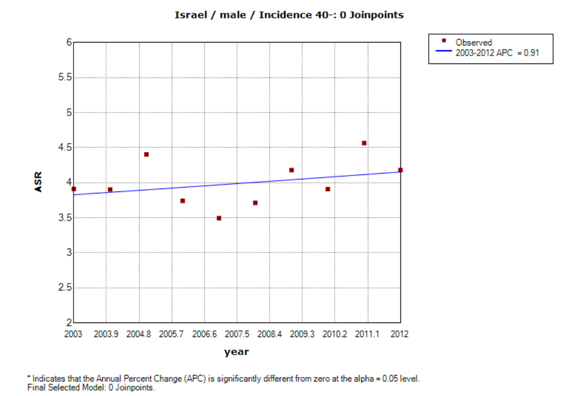

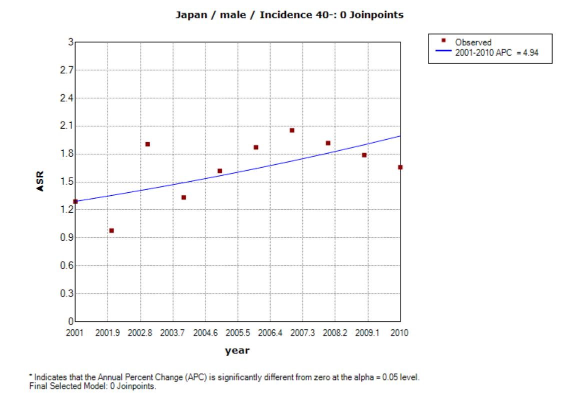

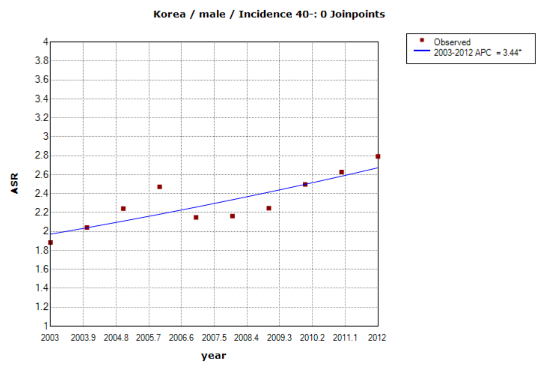

* Indicates that the Annual Percent Change (APC) is significantly different from zero at the alpha = 0.05 level.
Final Selected Model: 0 Joinpoints.

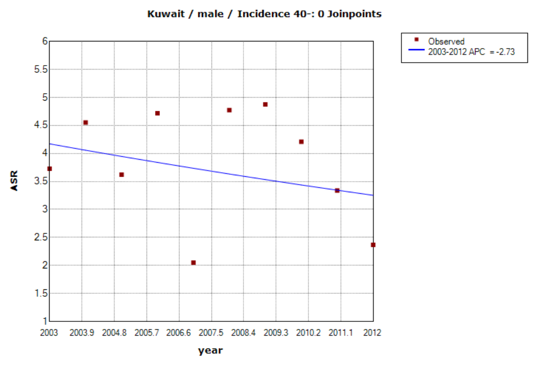

* Indicates that the Annual Percent Change (APC) is significantly different from zero at the alpha = 0.05 level.
Final Selected Model: 0 Joinpoints.

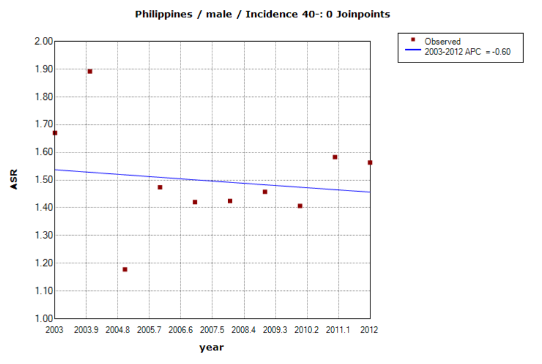

* Indicates that the Annual Percent Change (APC) is significantly different from zero at the alpha = 0.05 level.
Final Selected Model: 0 Joinpoints.

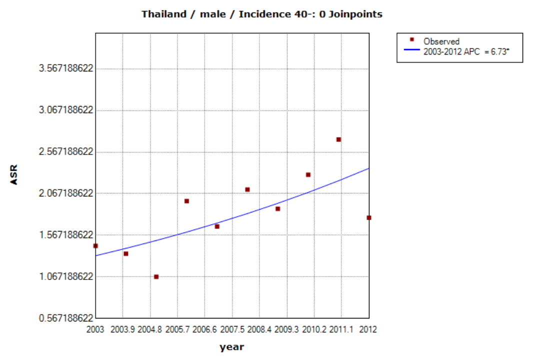

* Indicates that the Annual Percent Change (APC) is significantly different from zero at the alpha = 0.05 level.
Final Selected Model: 0 Joinpoints.

Oceania

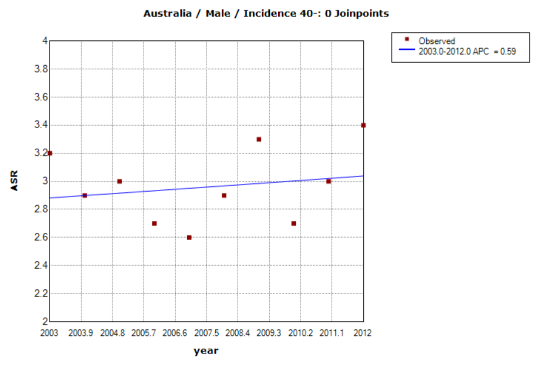

* Indicates that the Annual Percent Change (APC) is significantly different from zero at the alpha = 0.05 level.
Final Selected Model: 0 Joinpoints.

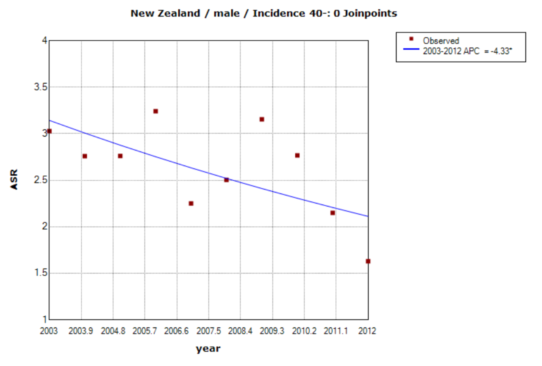

* Indicates that the Annual Percent Change (APC) is significantly different from zero at the alpha = 0.05 level.
Final Selected Model: 0 Joinpoints.

Northern America

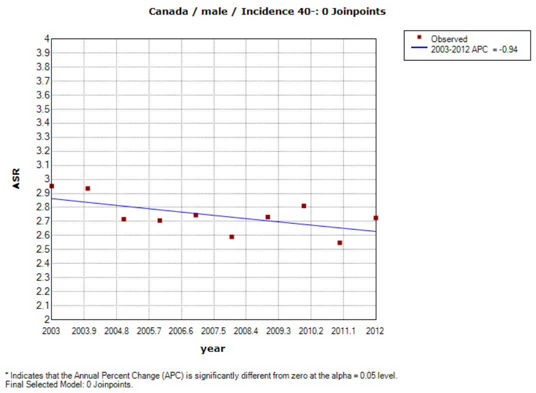

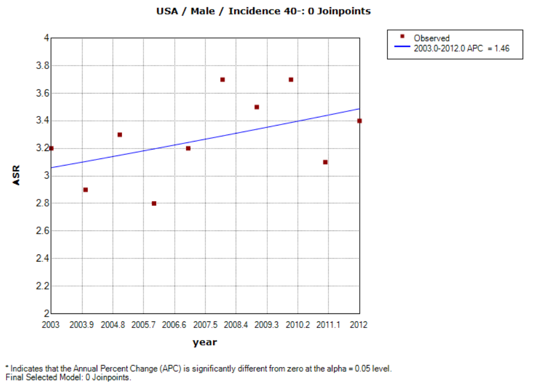

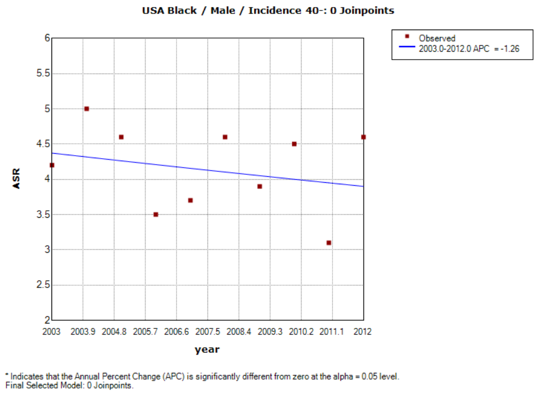

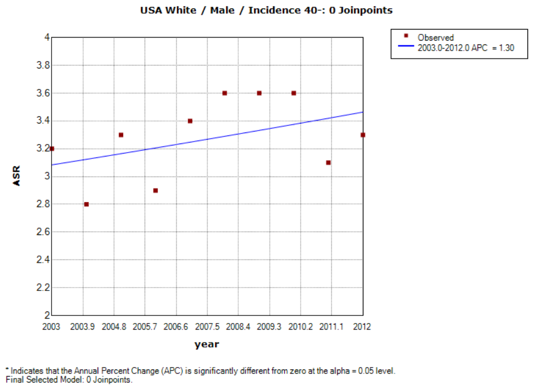

Southern America

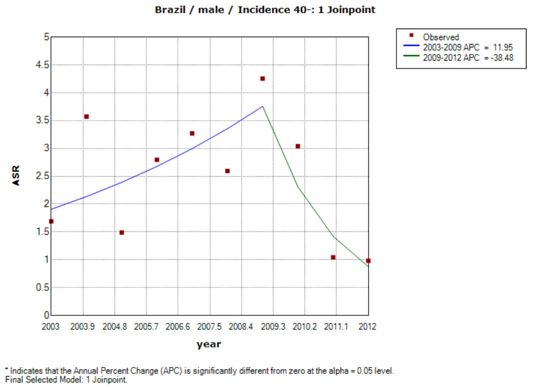

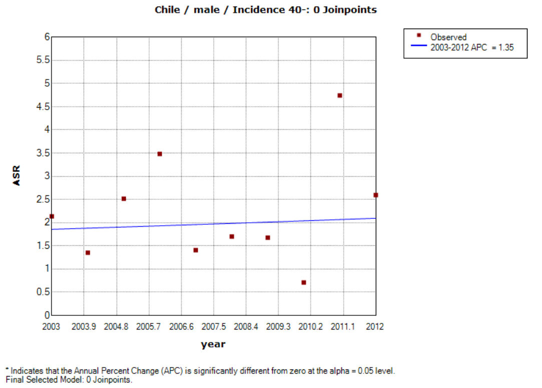

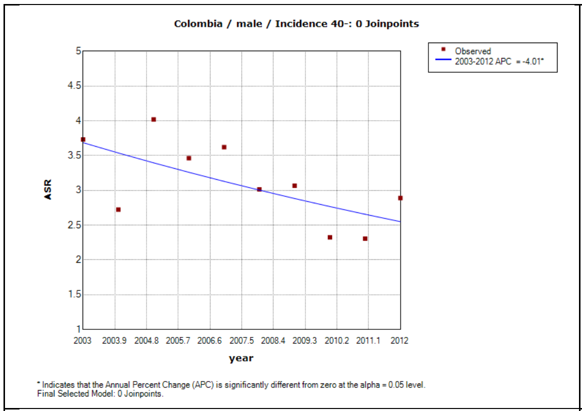

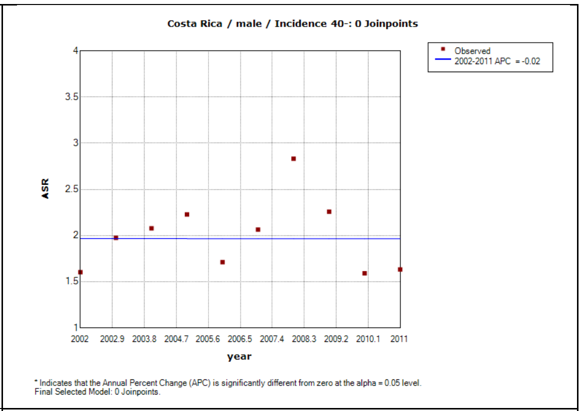

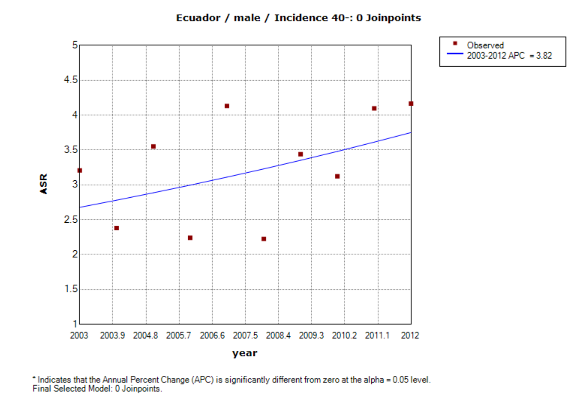

Northern Europe

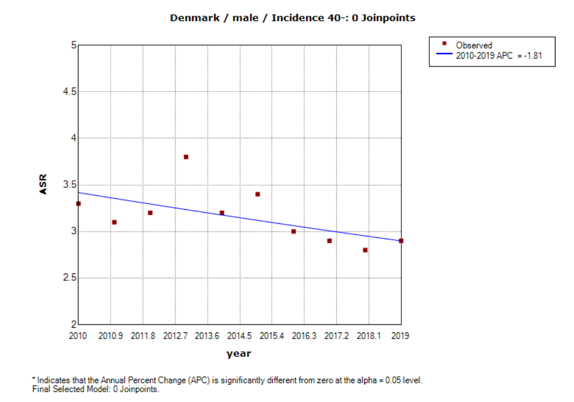

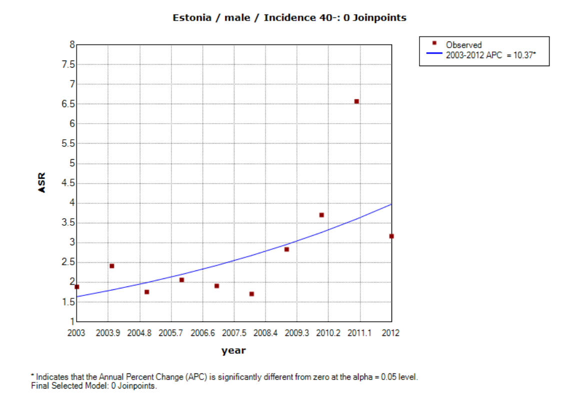

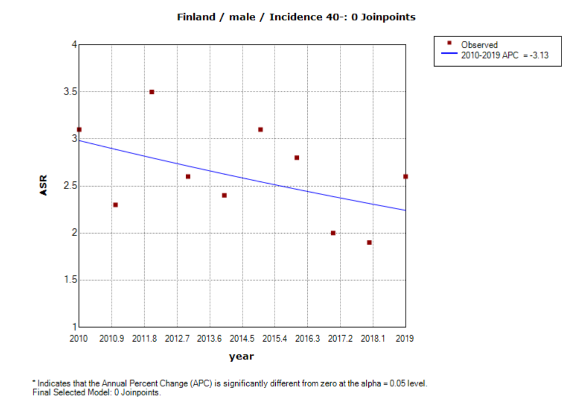

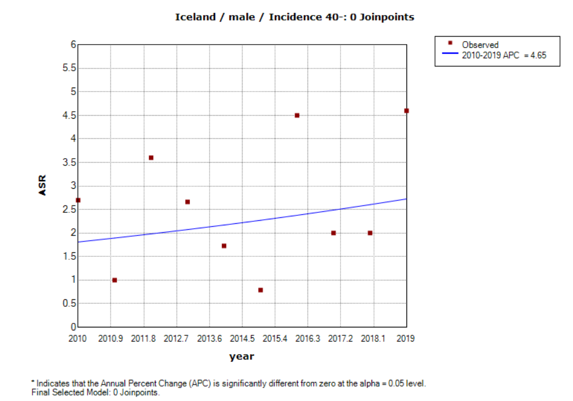

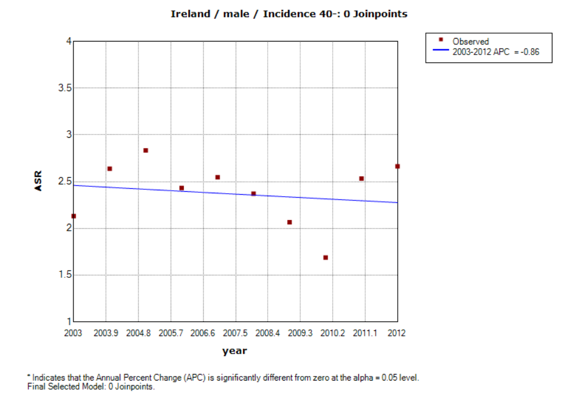

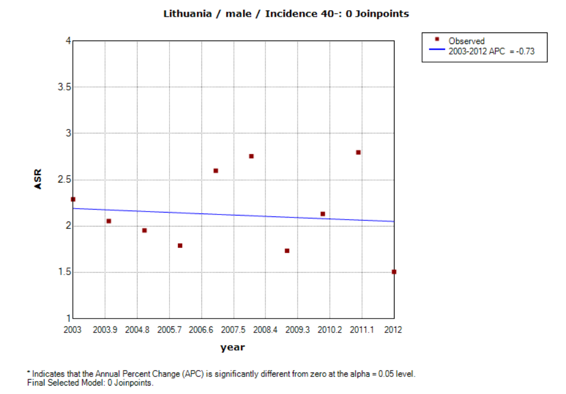

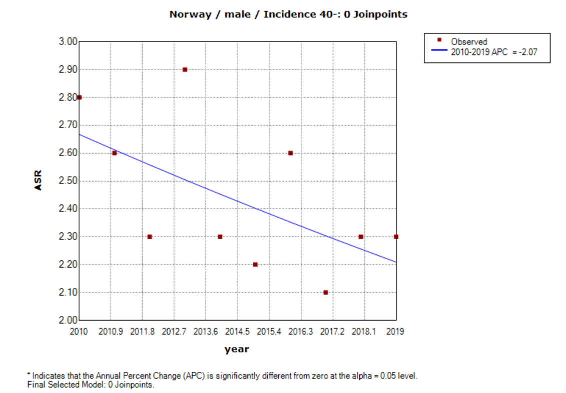

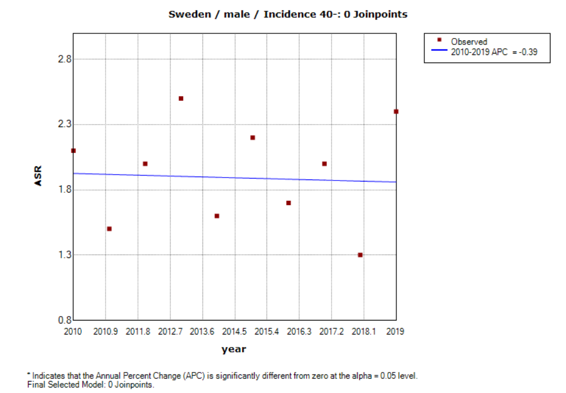

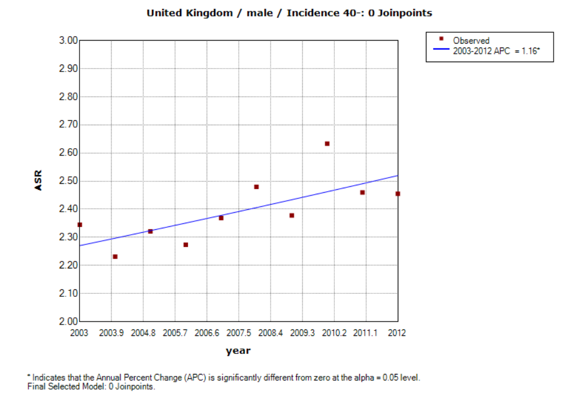

Western Europe

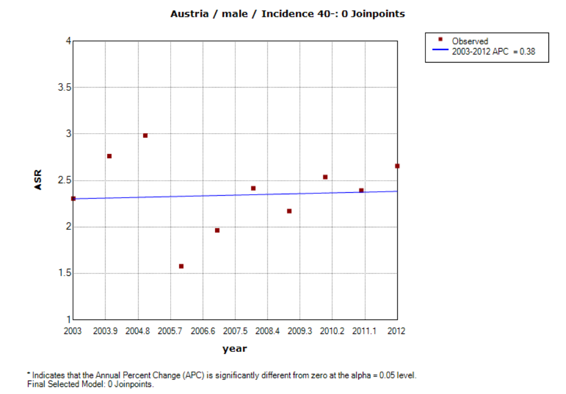

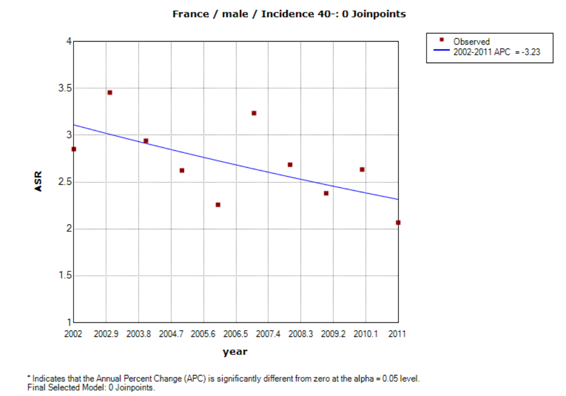

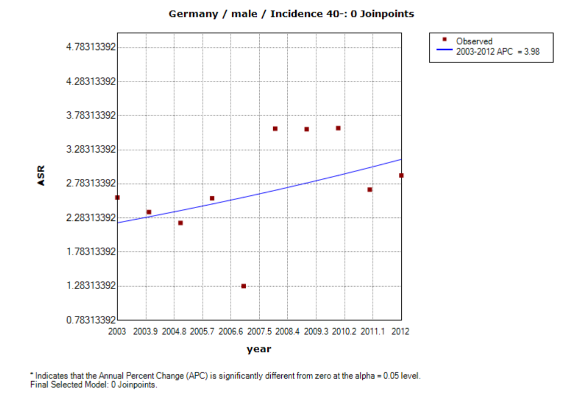

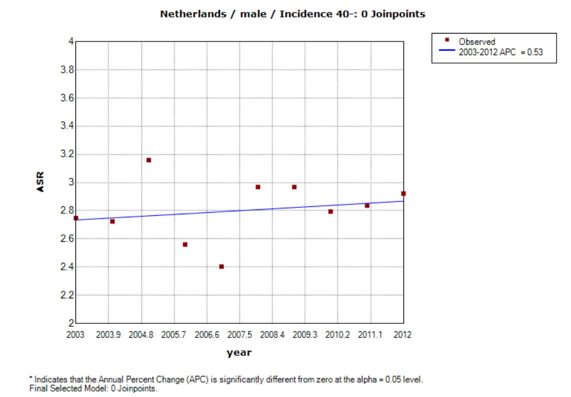

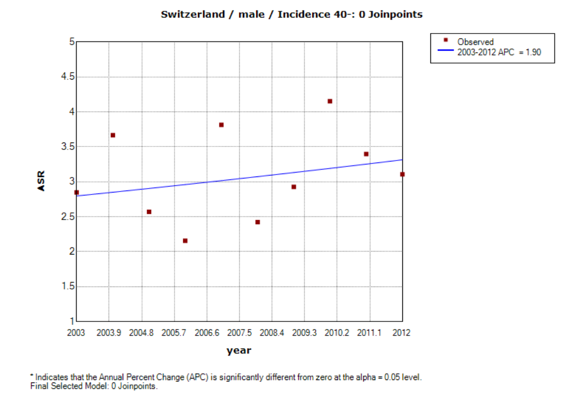

Southern Europe

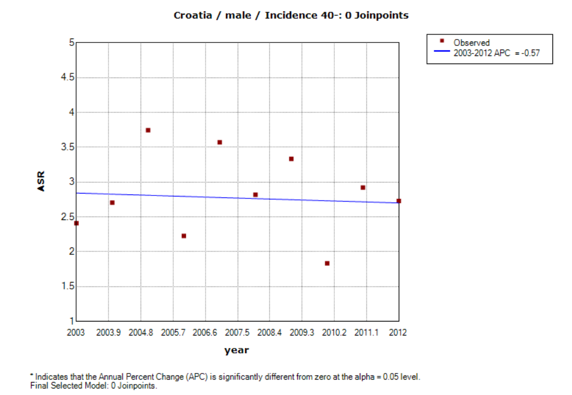

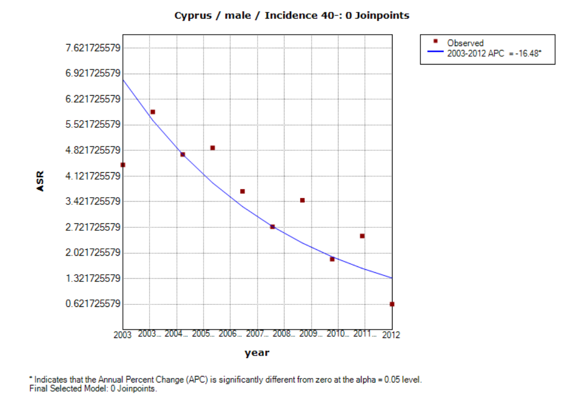

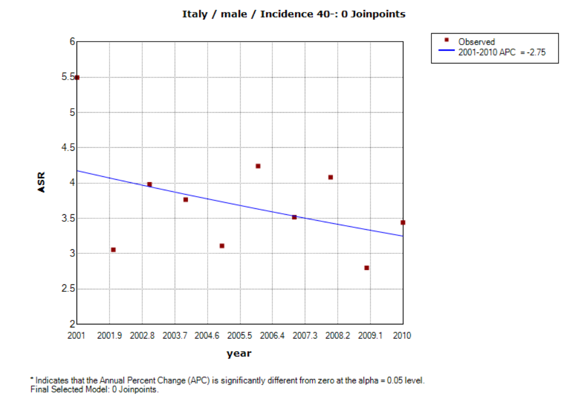

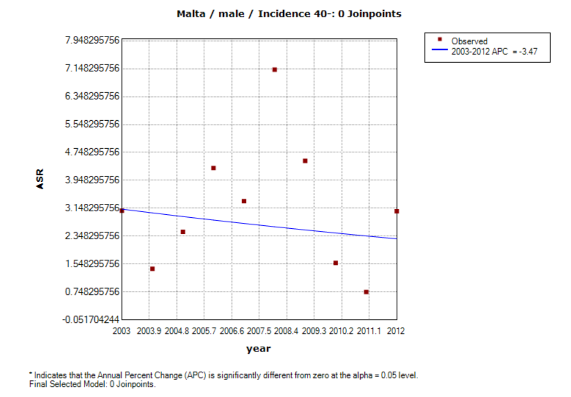

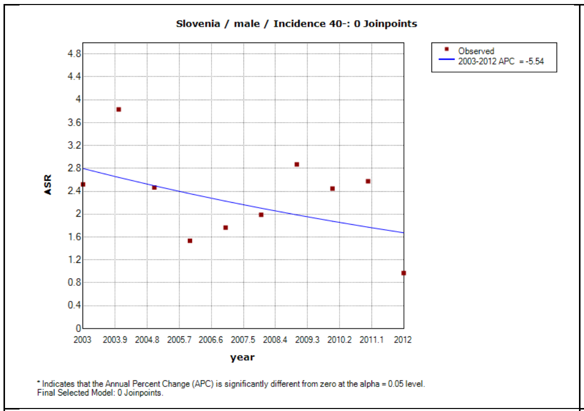

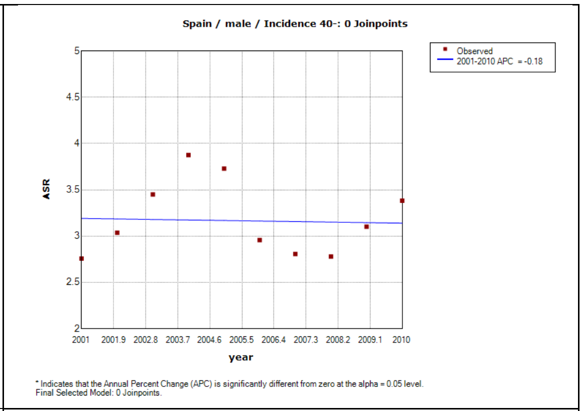

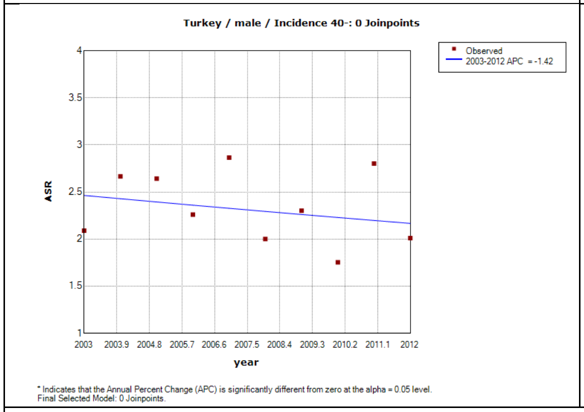

Eastern Europe

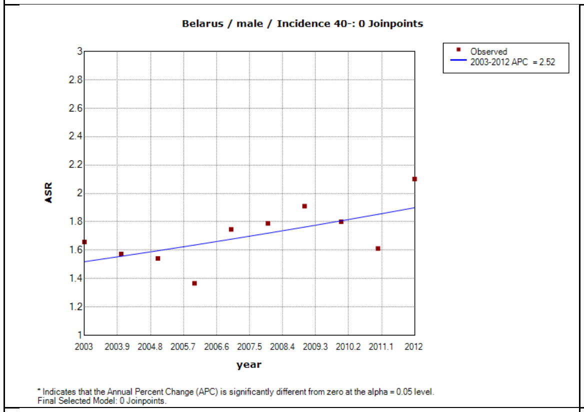

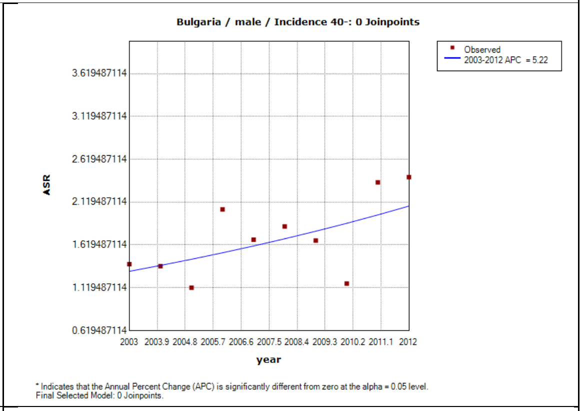

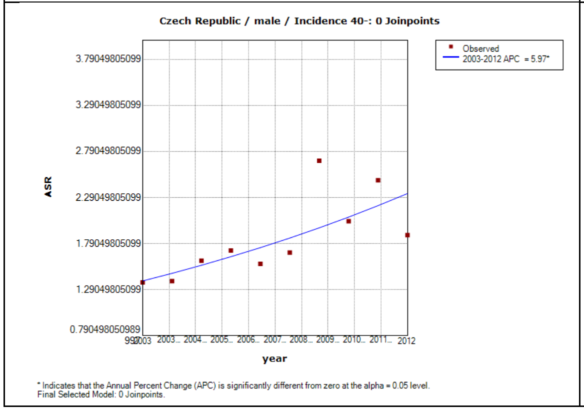

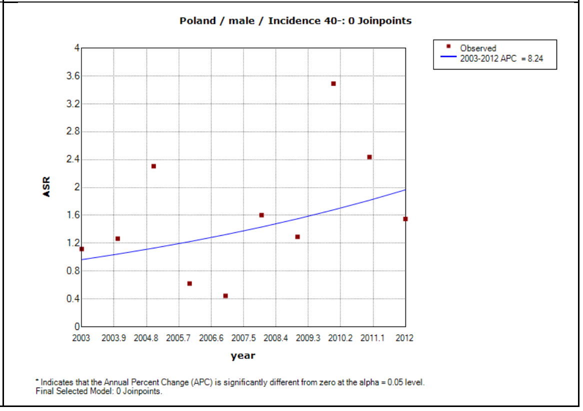

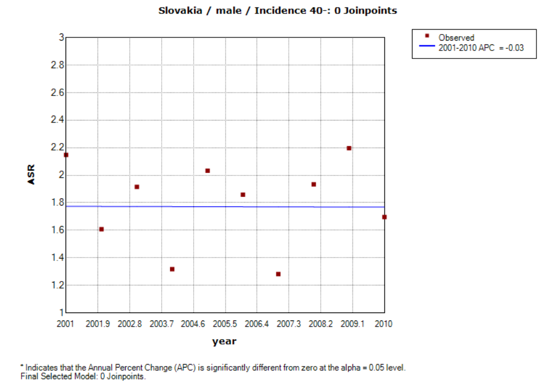

Slovakia / male / Incidence 40-: 0 Joinpoints
Observed
2001-2010 APC = -0.03
ASR
year
3
2.8
2.6
2.4
2.2
2
1.8
1.6
1.4
1.2
1
2001
2001.9
2002.8
2003.7
2004.6
2005.5
2006.4
2007.3
2008.2
2009.1
2010
* Indicates that the Annual Percent Change (APC) is significantly different from zero at the alpha = 0.05 level.
Final Selected Model: 0 Joinpoints.

Africa

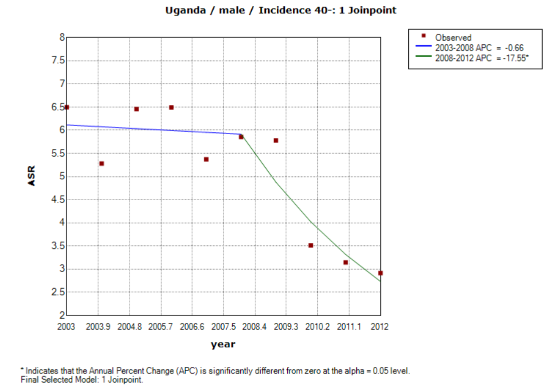

Uganda / male / Incidence 40-: 1 Joinpoint
Observed
2003-2008 APC = -0.66
2008-2012 APC = -17.55*
ASR
year
8
7.5
7
6.5
6
5.5
5
4.5
4
3.5
3
2.5
2
2003
2003.9
2004.8
2005.7
2006.6
2007.5
2008.4
2009.3
2010.2
2011.1
2012
* Indicates that the Annual Percent Change (APC) is significantly different from zero at the alpha = 0.05 level.
Final Selected Model: 1 Joinpoint.

d.) Incidence female below 40 years old

Asia

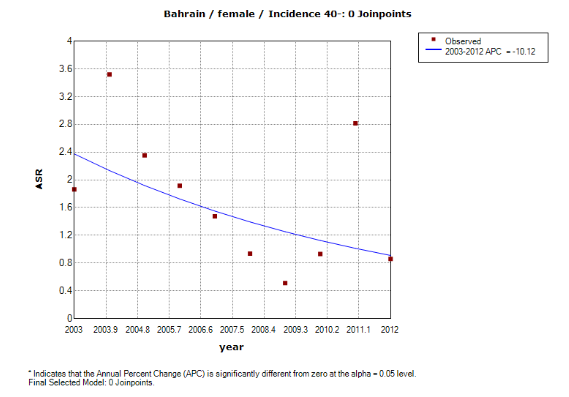

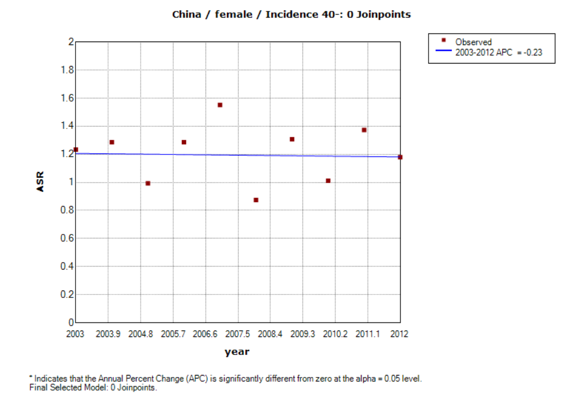

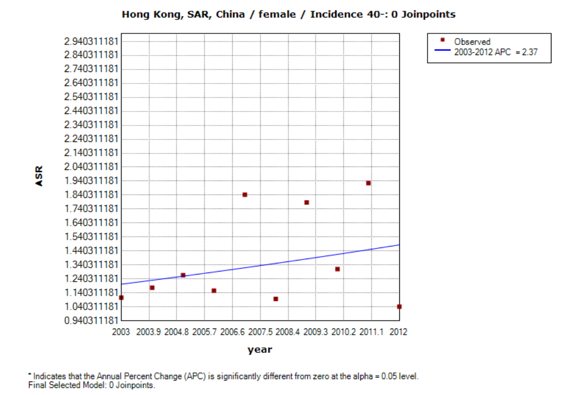

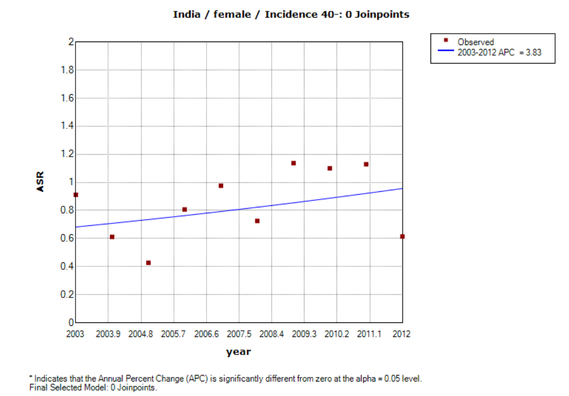

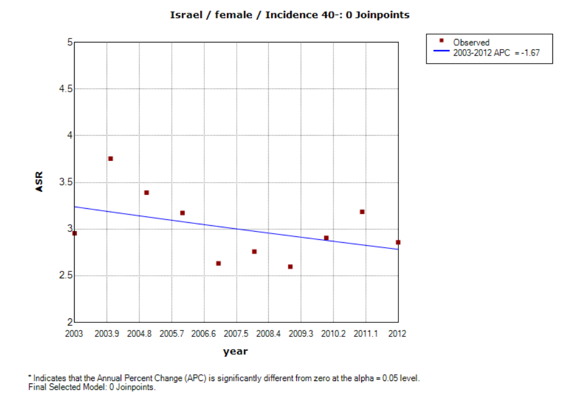

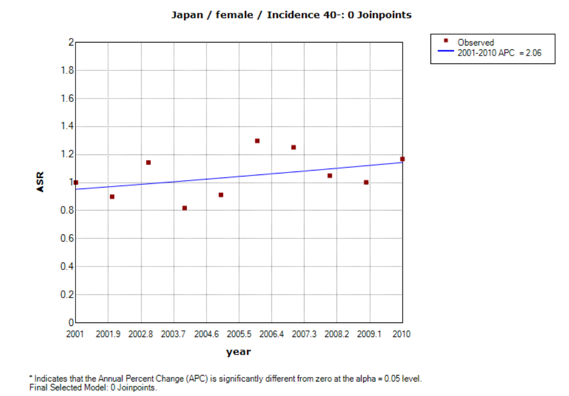

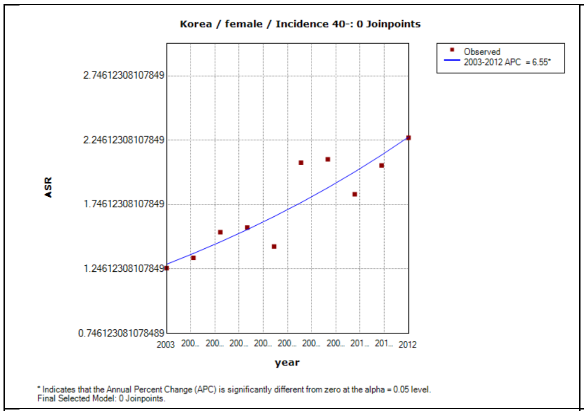
Korea / female / Incidence 40-: 0 Joinpoints
Observed
2003-2012 APC = 6.55*
ASR
year
* Indicates that the Annual Percent Change (APC) is significantly different from zero at the alpha = 0.05 level.
Final Selected Model: 0 Joinpoints.

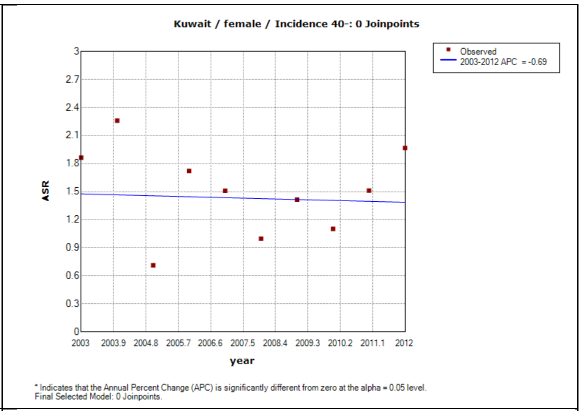
Kuwait / female / Incidence 40-: 0 Joinpoints
Observed
2003-2012 APC = -0.69
ASR
year
* Indicates that the Annual Percent Change (APC) is significantly different from zero at the alpha = 0.05 level.
Final Selected Model: 0 Joinpoints.

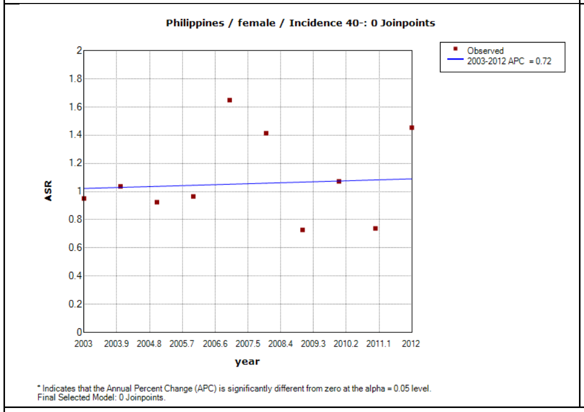
Philippines / female / Incidence 40-: 0 Joinpoints
Observed
2003-2012 APC = 0.72
ASR
year
* Indicates that the Annual Percent Change (APC) is significantly different from zero at the alpha = 0.05 level.
Final Selected Model: 0 Joinpoints.

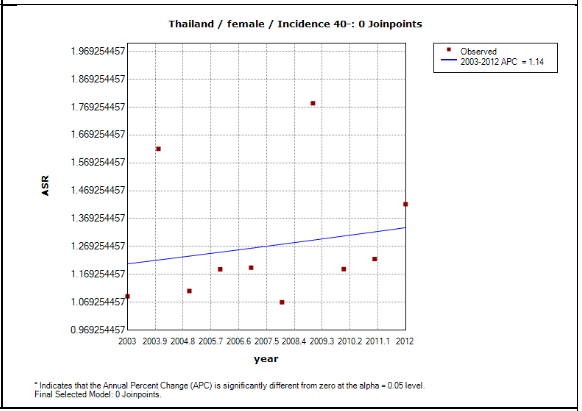
Thailand / female / Incidence 40-: 0 Joinpoints
Observed
2003-2012 APC = 1.14
ASR
year
* Indicates that the Annual Percent Change (APC) is significantly different from zero at the alpha = 0.05 level.
Final Selected Model: 0 Joinpoints.

Oceania

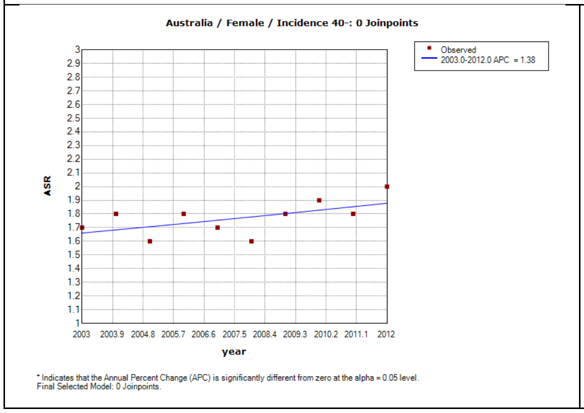
Australia / Female / Incidence 40-: 0 Joinpoints
Observed
2003.0-2012.0 APC = 1.38
ASR
year
* Indicates that the Annual Percent Change (APC) is significantly different from zero at the alpha = 0.05 level.
Final Selected Model: 0 Joinpoints.

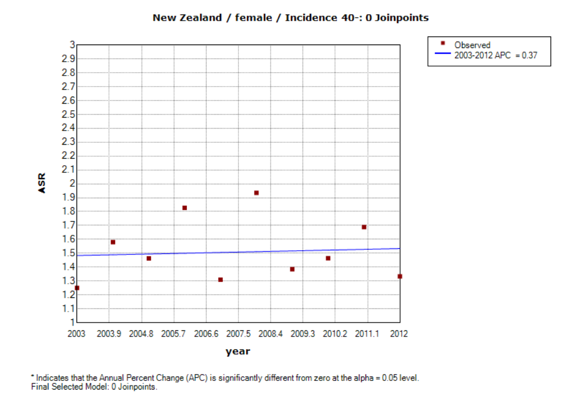
New Zealand / female / Incidence 40-: 0 Joinpoints
Observed
2003-2012 APC = 0.37
ASR
year
* Indicates that the Annual Percent Change (APC) is significantly different from zero at the alpha = 0.05 level.
Final Selected Model: 0 Joinpoints.

Northern America

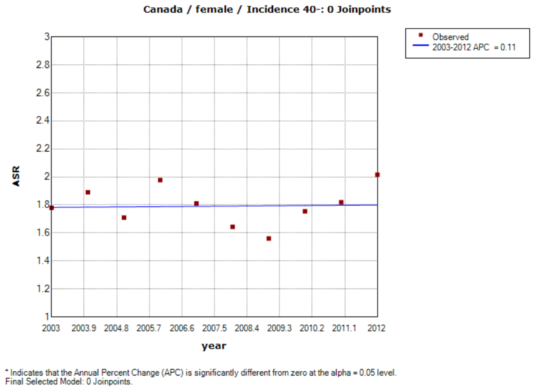

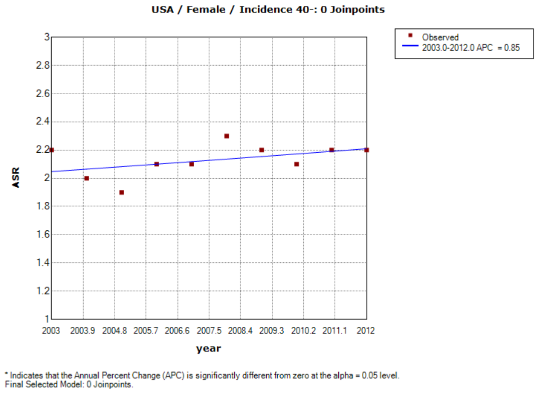

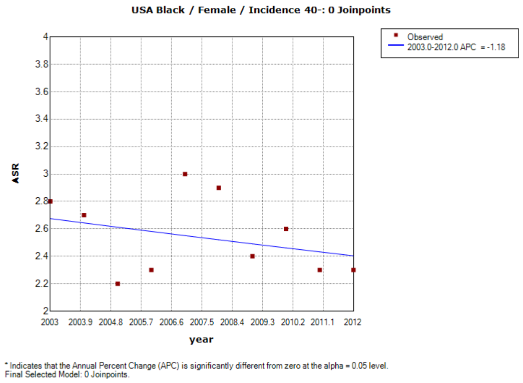

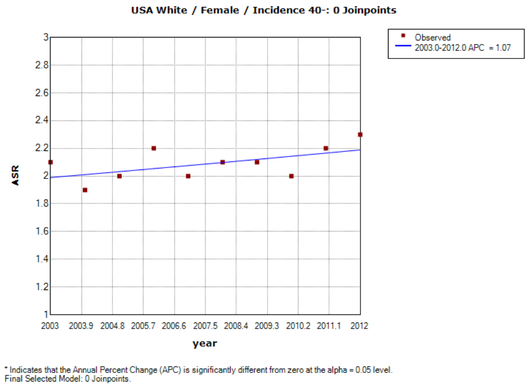

Southern America

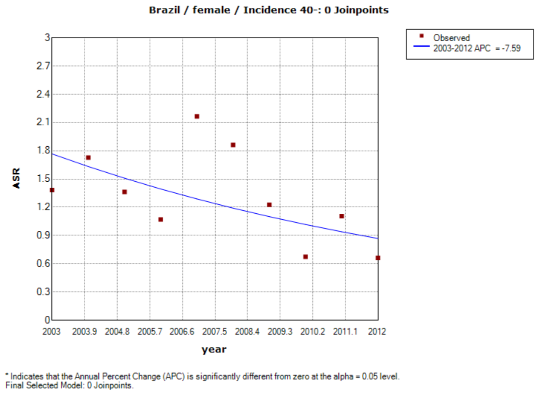

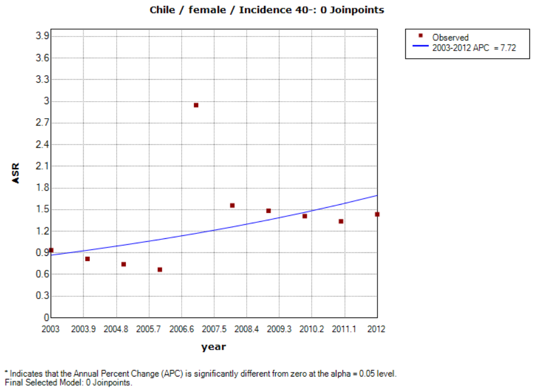

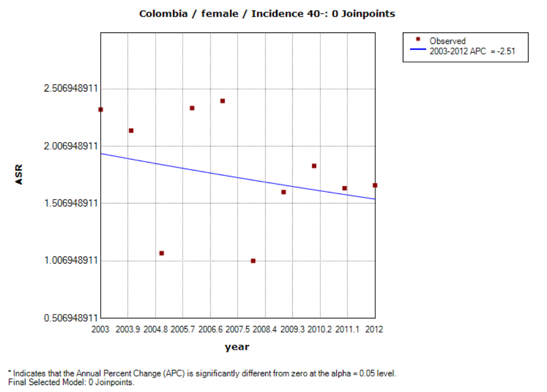

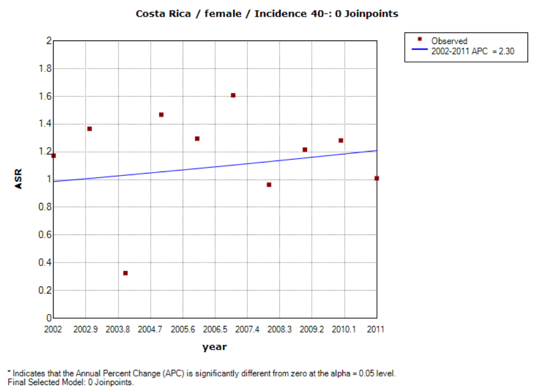

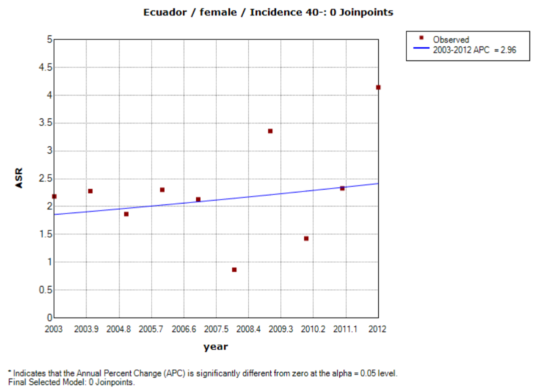

Northern Europe

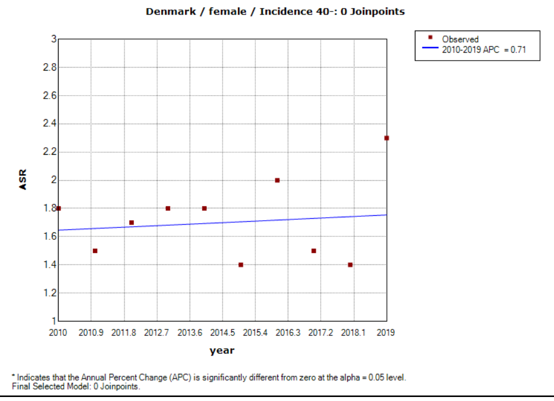

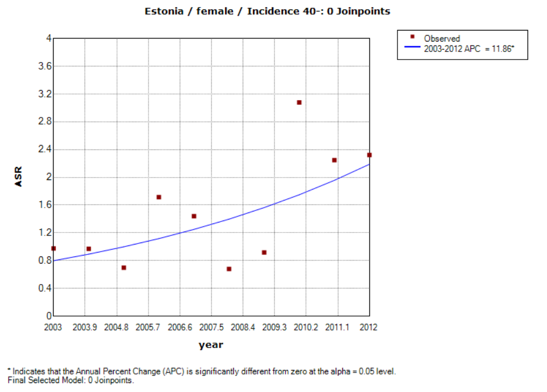

Finland / female / Incidence 40-: 0 Joinpoints

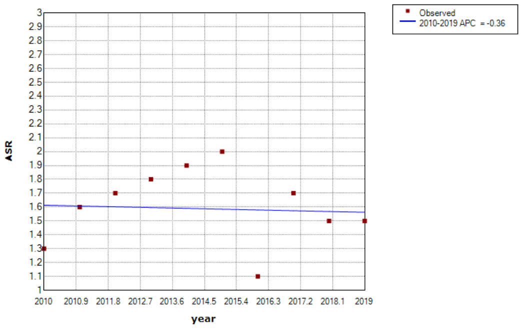

* Indicates that the Annual Percent Change (APC) is significantly different from zero at the alpha = 0.05 level.
Final Selected Model: 0 Joinpoints.

Iceland / female / Incidence 40-: 0 Joinpoints

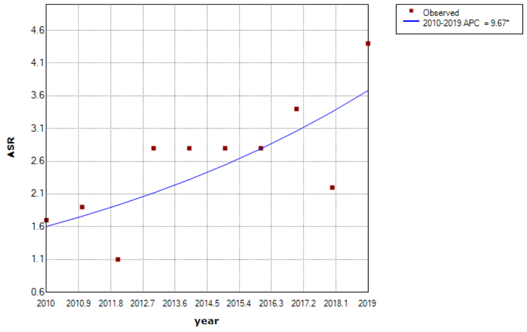

* Indicates that the Annual Percent Change (APC) is significantly different from zero at the alpha = 0.05 level.
Final Selected Model: 0 Joinpoints.

Ireland / female / Incidence 40-: 1 Joinpoint

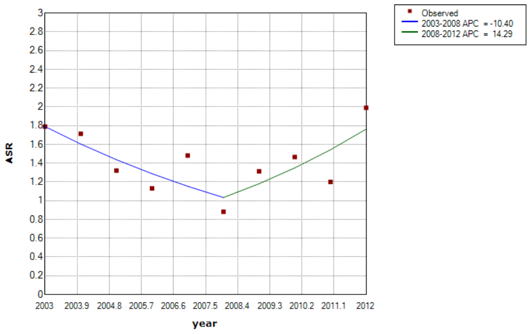

* Indicates that the Annual Percent Change (APC) is significantly different from zero at the alpha = 0.05 level.
Final Selected Model: 1 Joinpoint.

Lithuania / female / Incidence 40-: 0 Joinpoints

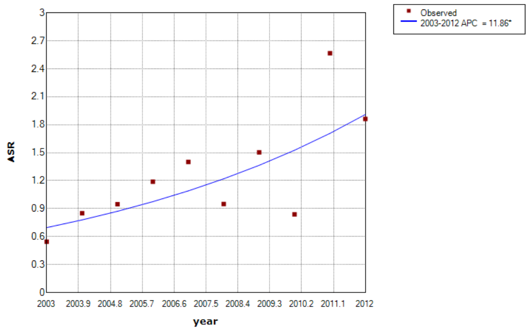

* Indicates that the Annual Percent Change (APC) is significantly different from zero at the alpha = 0.05 level.
Final Selected Model: 0 Joinpoints.

Norway / female / Incidence 40-: 0 Joinpoints

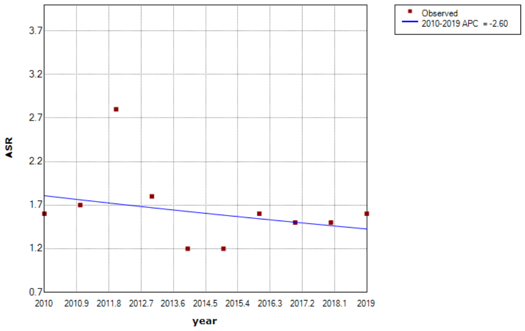

* Indicates that the Annual Percent Change (APC) is significantly different from zero at the alpha = 0.05 level.
Final Selected Model: 0 Joinpoints.

Sweden / female / Incidence 40-: 0 Joinpoints

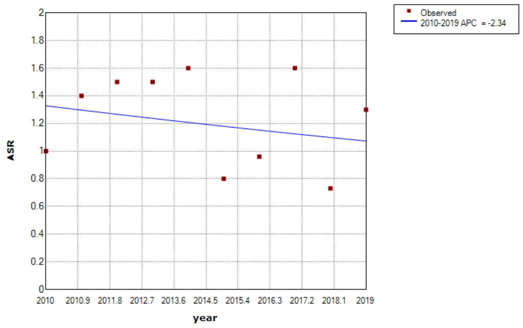

* Indicates that the Annual Percent Change (APC) is significantly different from zero at the alpha = 0.05 level.
Final Selected Model: 0 Joinpoints.

United Kingdom / female / Incidence 40-: 0 Joinpoints

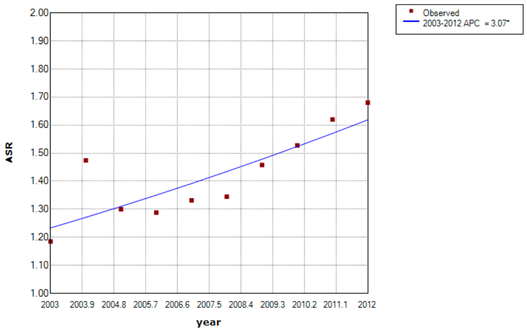

* Indicates that the Annual Percent Change (APC) is significantly different from zero at the alpha = 0.05 level.
Final Selected Model: 0 Joinpoints.

Western Europe

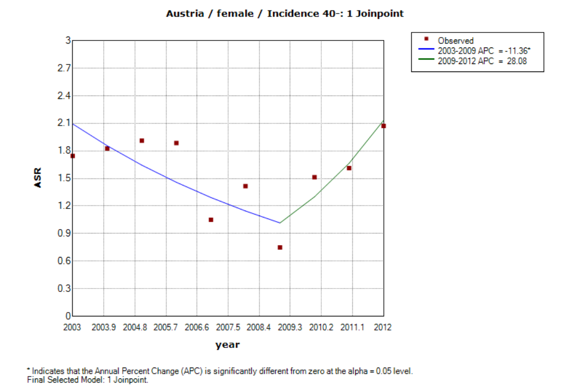

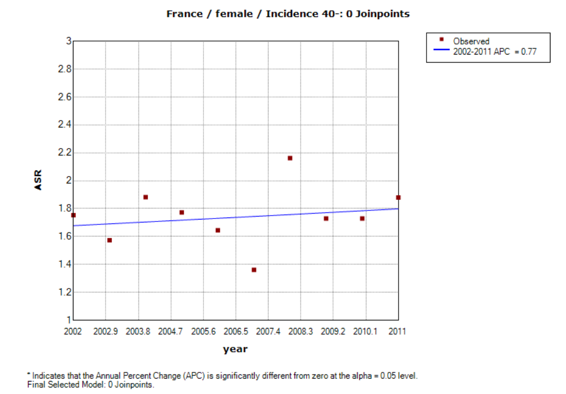

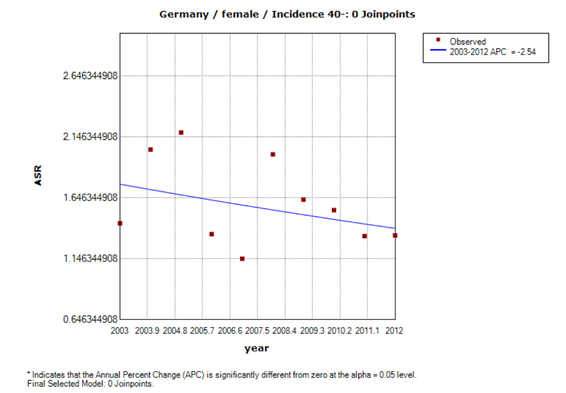

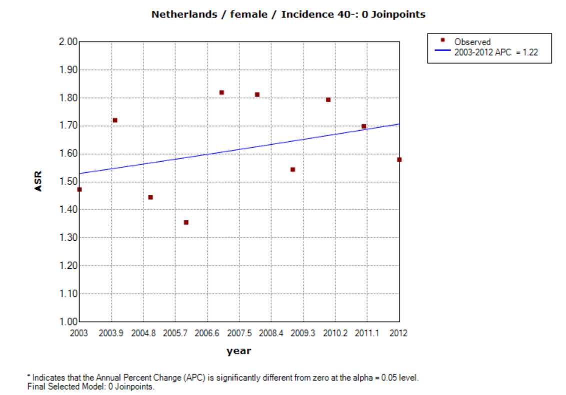

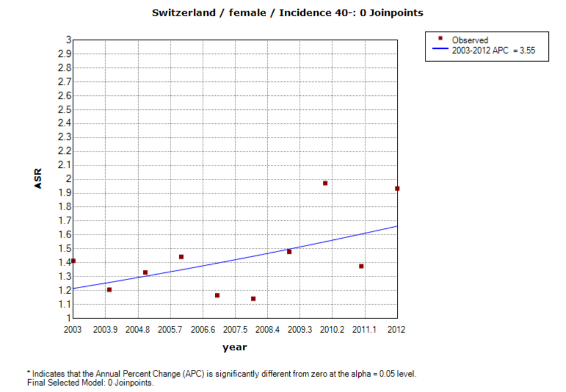

Southern Europe

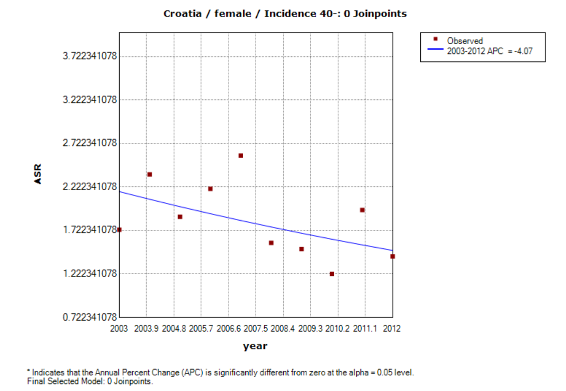

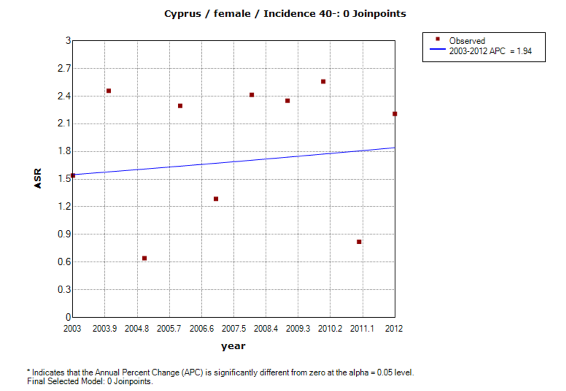

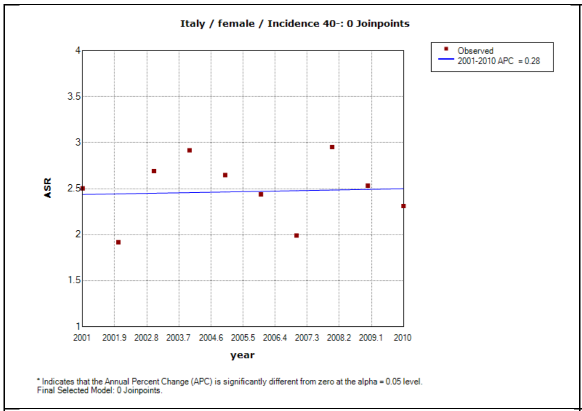

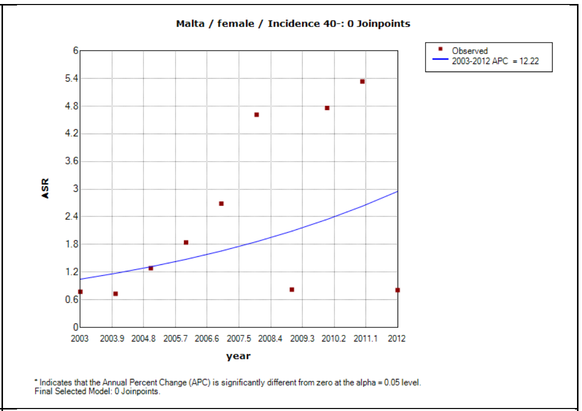

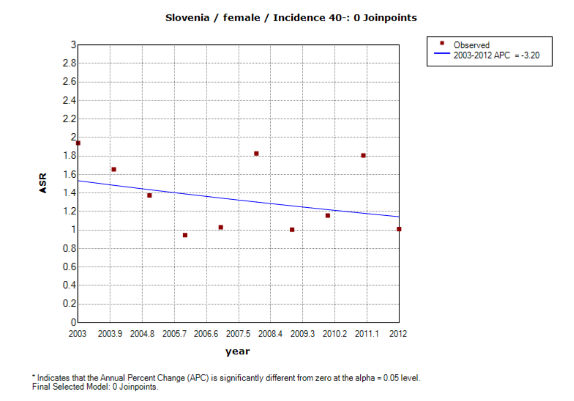

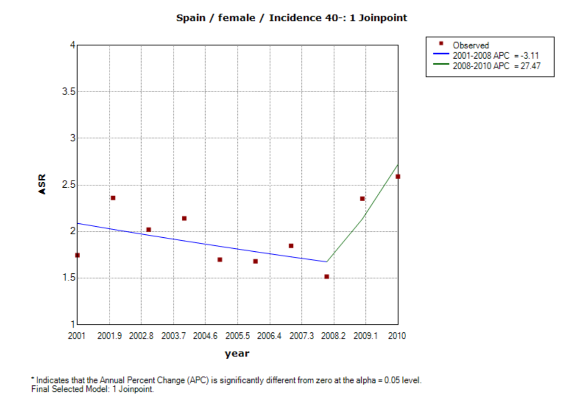

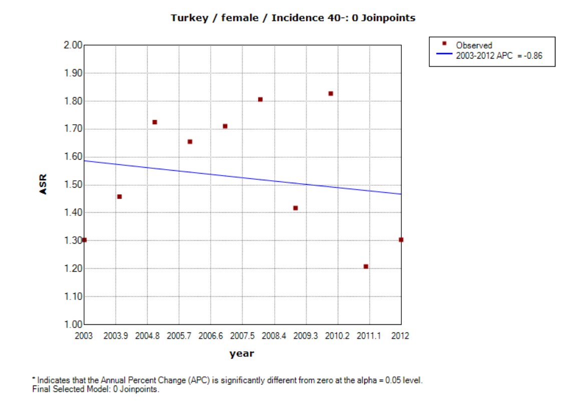

Eastern Europe

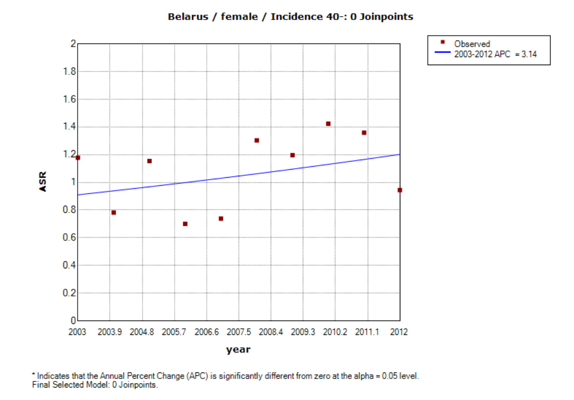

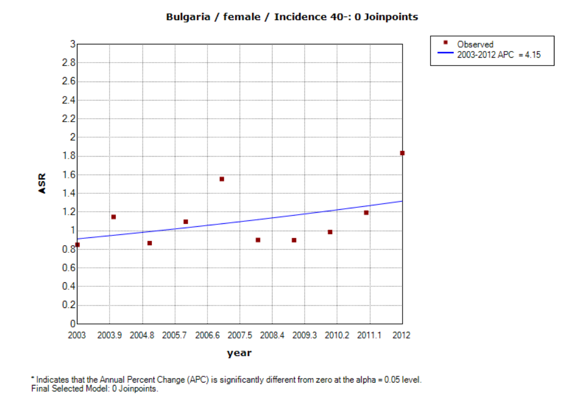

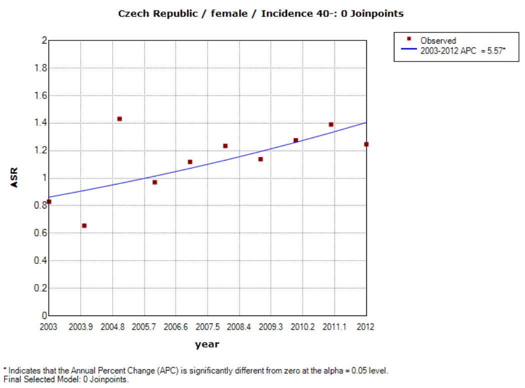
Czech Republic / female / Incidence 40-: 0 Joinpoints
Observed
2003-2012 APC = 5.57*
ASR
year
* Indicates that the Annual Percent Change (APC) is significantly different from zero at the alpha = 0.05 level.
Final Selected Model: 0 Joinpoints.

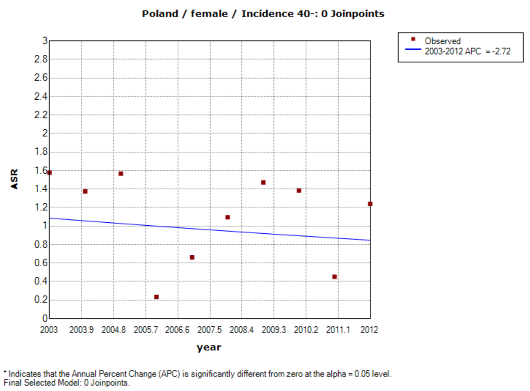
Poland / female / Incidence 40-: 0 Joinpoints
Observed
2003-2012 APC = -2.72
ASR
year
* Indicates that the Annual Percent Change (APC) is significantly different from zero at the alpha = 0.05 level.
Final Selected Model: 0 Joinpoints.

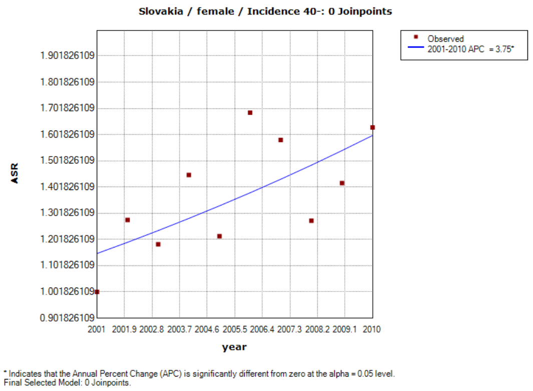
Slovakia / female / Incidence 40-: 0 Joinpoints
Observed
2001-2010 APC = 3.75*
ASR
year
* Indicates that the Annual Percent Change (APC) is significantly different from zero at the alpha = 0.05 level.
Final Selected Model: 0 Joinpoints.

Africa

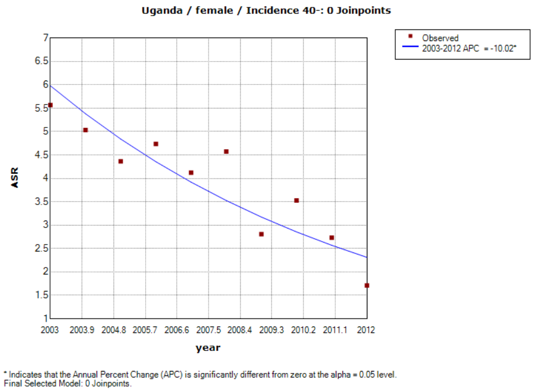
Uganda / female / Incidence 40-: 0 Joinpoints
Observed
2003-2012 APC = -10.02*
ASR
year
* Indicates that the Annual Percent Change (APC) is significantly different from zero at the alpha = 0.05 level.
Final Selected Model: 0 Joinpoints.

e.) Incidence male below 50 years old

Asia

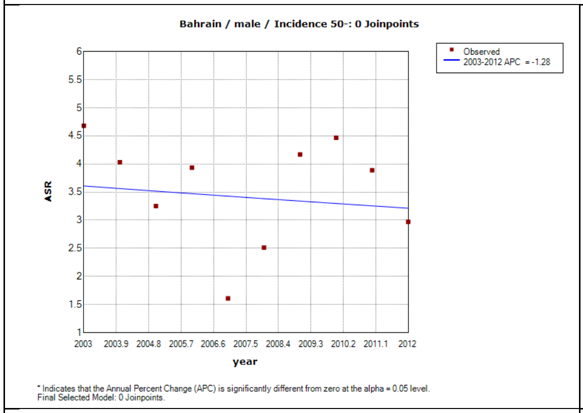

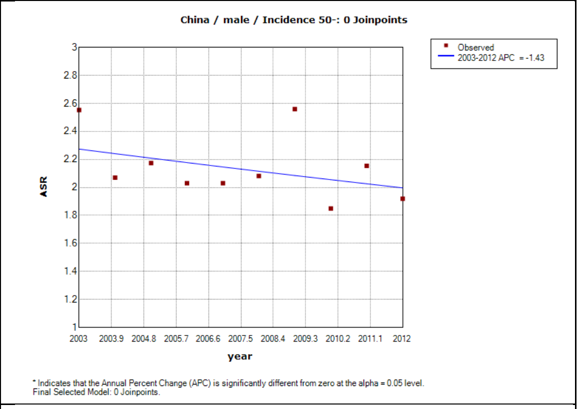

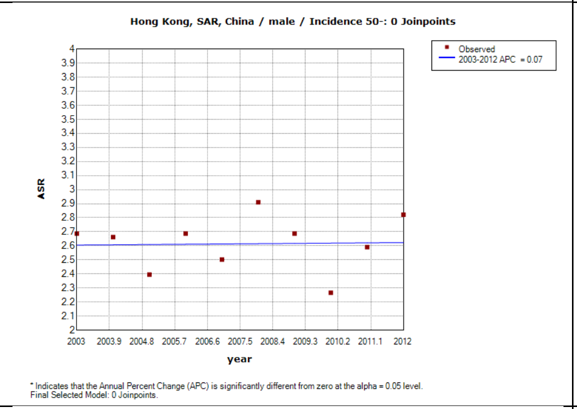

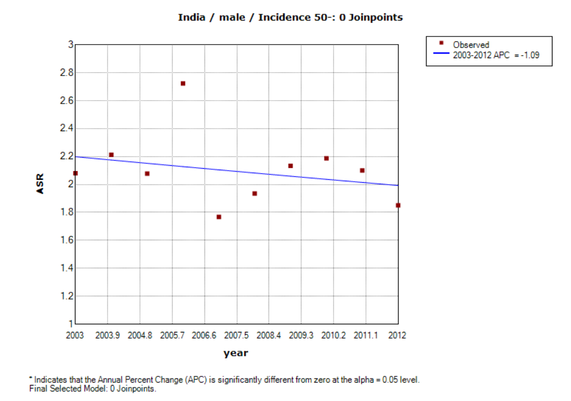

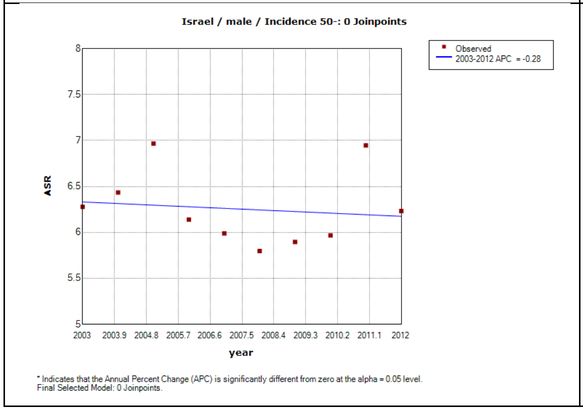

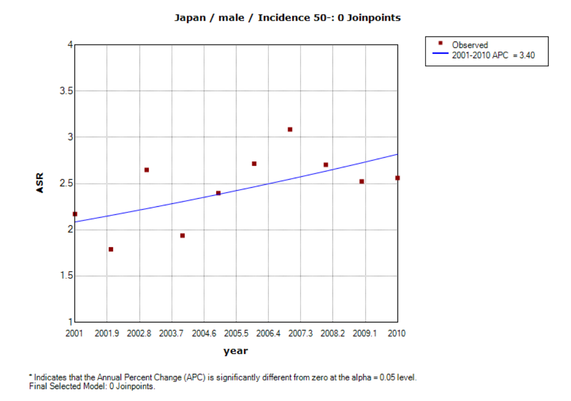

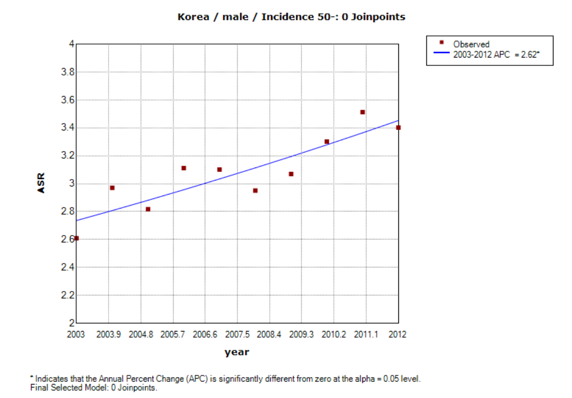
Korea / male / Incidence 50-: 0 Joinpoints
Observed
2003-2012 APC = 2.62*
ASR
year
* Indicates that the Annual Percent Change (APC) is significantly different from zero at the alpha = 0.05 level.
Final Selected Model: 0 Joinpoints.

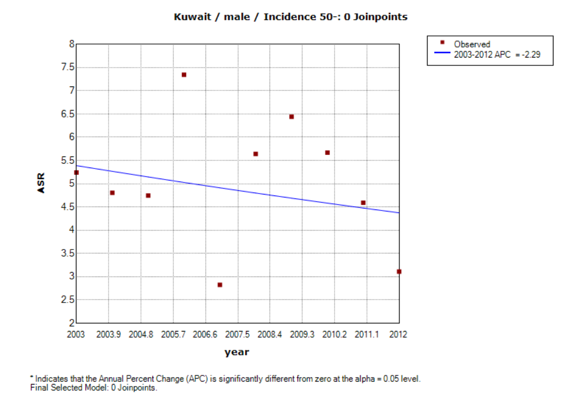
Kuwait / male / Incidence 50-: 0 Joinpoints
Observed
2003-2012 APC = -2.29
ASR
year
* Indicates that the Annual Percent Change (APC) is significantly different from zero at the alpha = 0.05 level.
Final Selected Model: 0 Joinpoints.

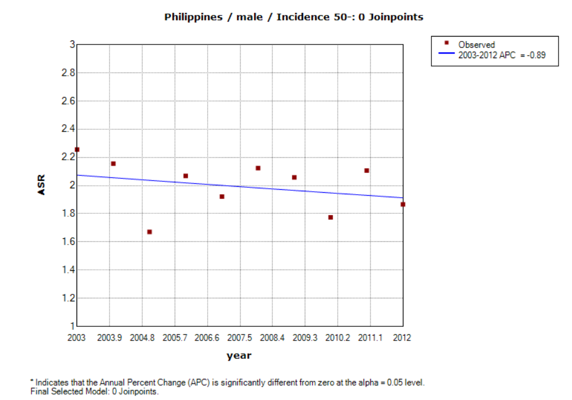
Philippines / male / Incidence 50-: 0 Joinpoints
Observed
2003-2012 APC = -0.89
ASR
year
* Indicates that the Annual Percent Change (APC) is significantly different from zero at the alpha = 0.05 level.
Final Selected Model: 0 Joinpoints.

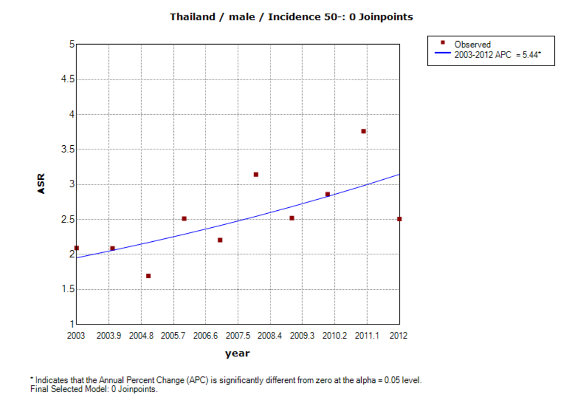
Thailand / male / Incidence 50-: 0 Joinpoints
Observed
2003-2012 APC = 5.44*
ASR
year
* Indicates that the Annual Percent Change (APC) is significantly different from zero at the alpha = 0.05 level.
Final Selected Model: 0 Joinpoints.

Oceania

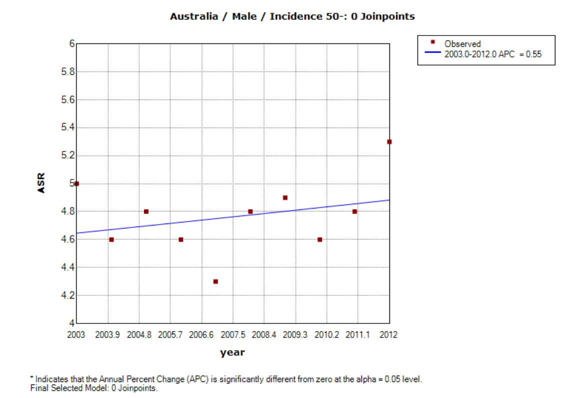
Australia / Male / Incidence 50-: 0 Joinpoints
Observed
2003.0-2012.0 APC = 0.55
ASR
year
* Indicates that the Annual Percent Change (APC) is significantly different from zero at the alpha = 0.05 level.
Final Selected Model: 0 Joinpoints.

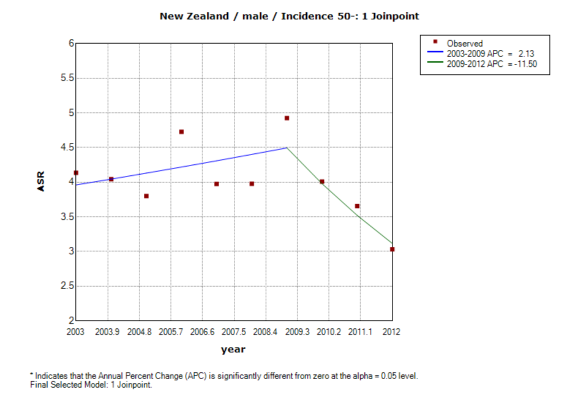
New Zealand / male / Incidence 50-: 1 Joinpoint
Observed
2003-2009 APC = 2.13
2009-2012 APC = -11.50
ASR
year
* Indicates that the Annual Percent Change (APC) is significantly different from zero at the alpha = 0.05 level.
Final Selected Model: 1 Joinpoint.

Northern America

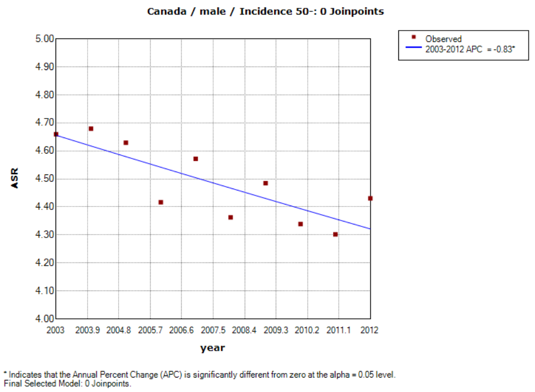

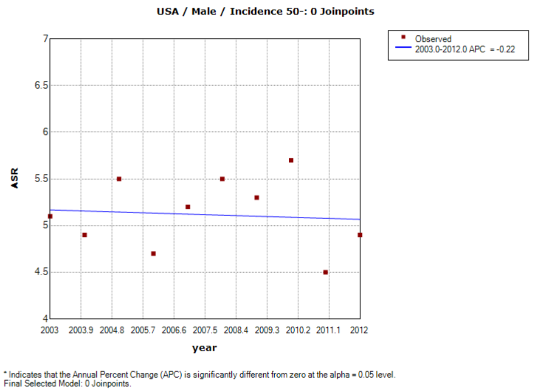

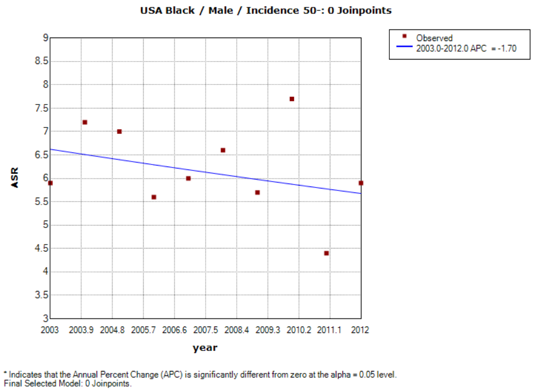

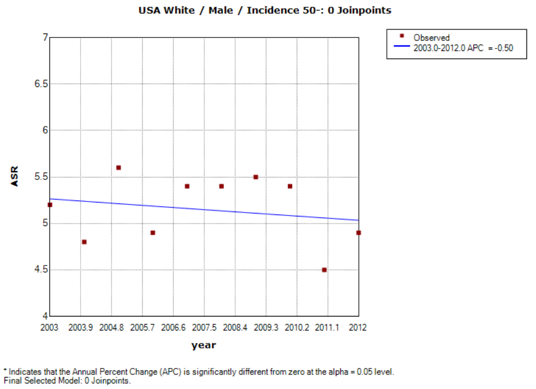

Southern America

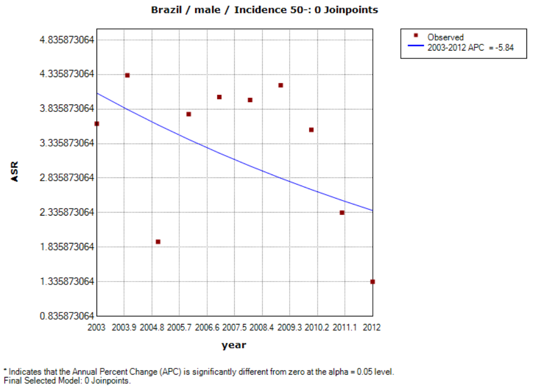

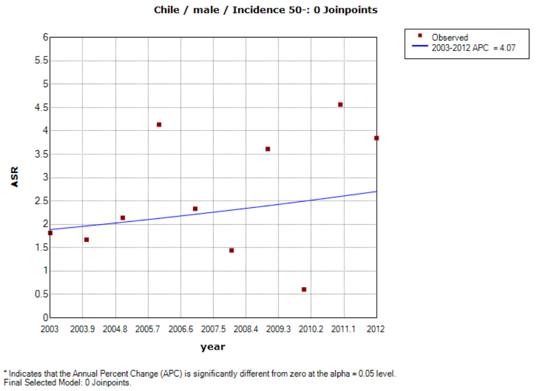

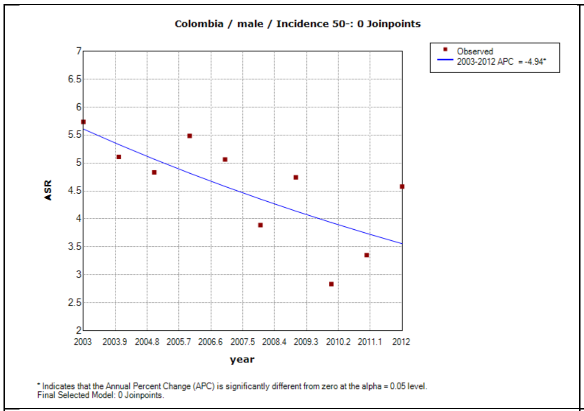

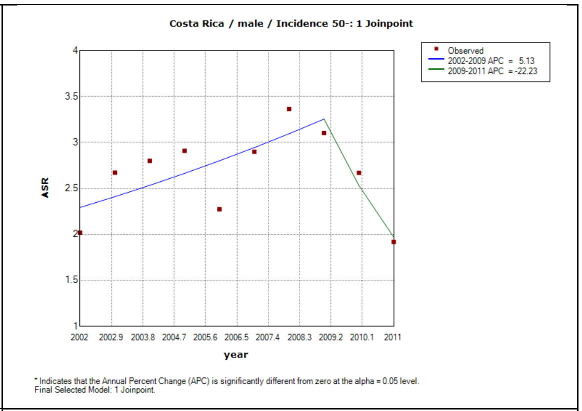

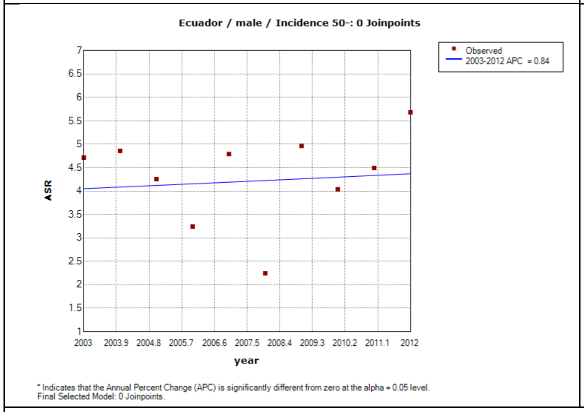

Northern Europe

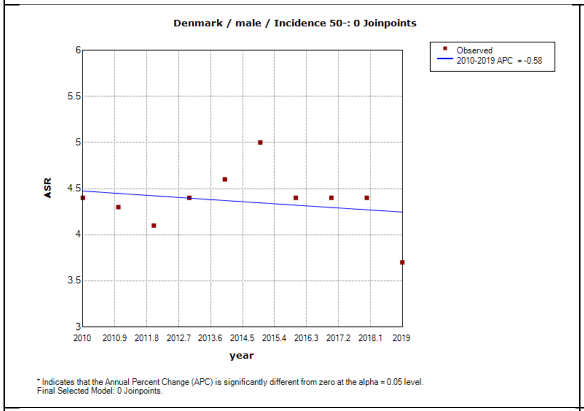

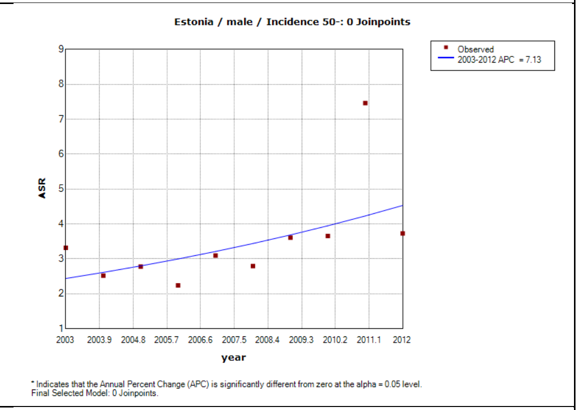

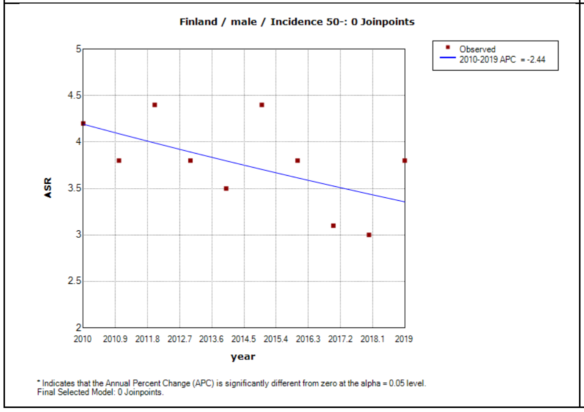

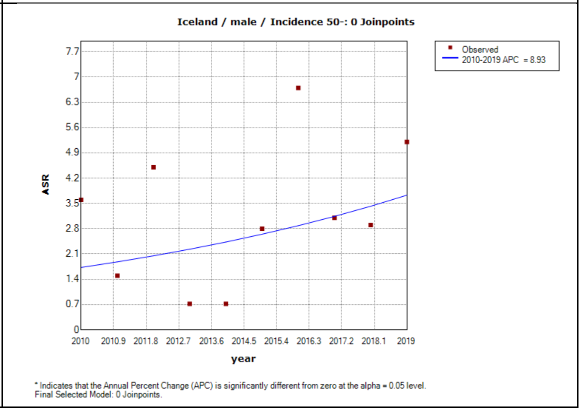

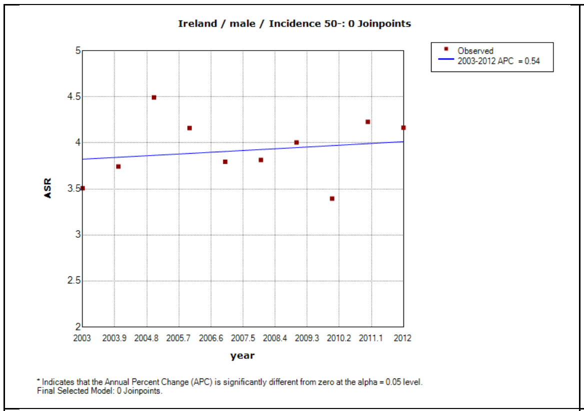

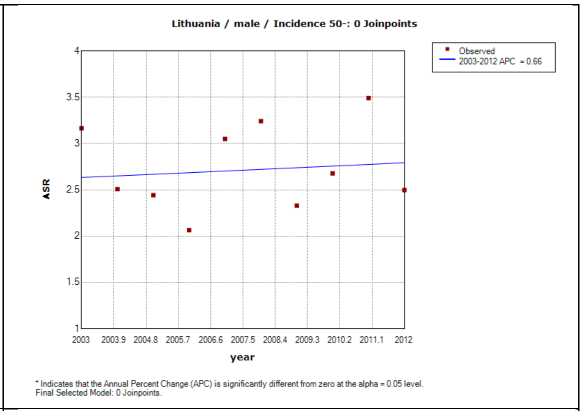

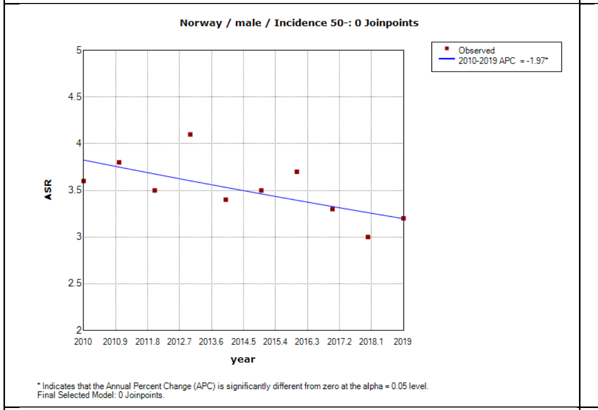

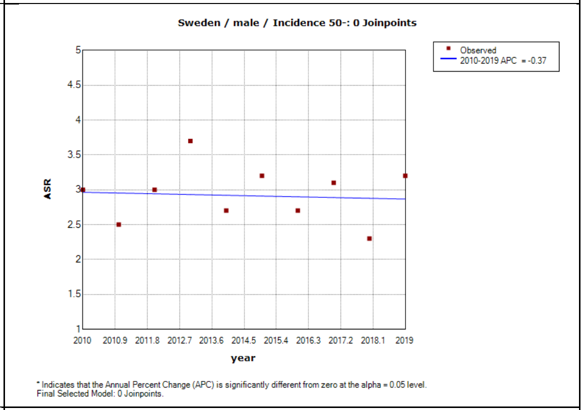

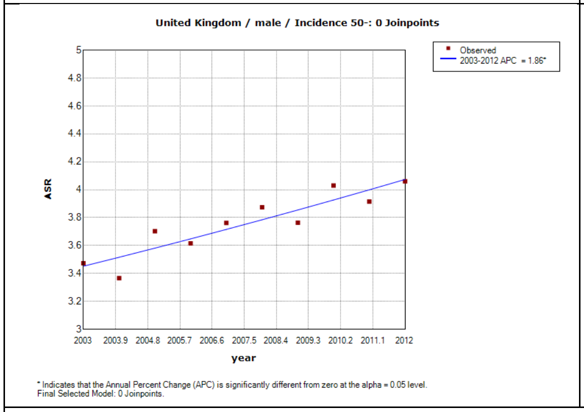

Western Europe

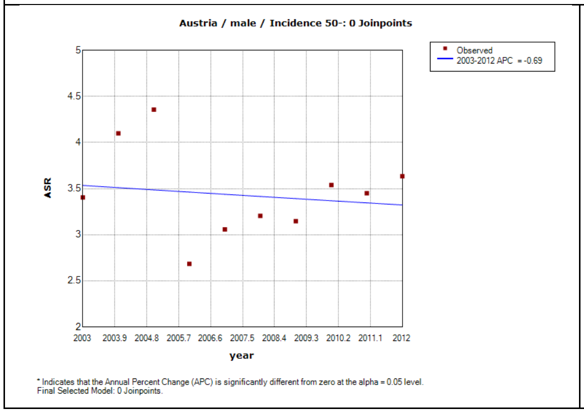

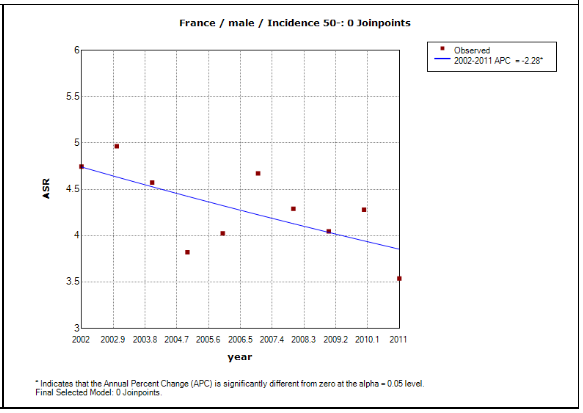

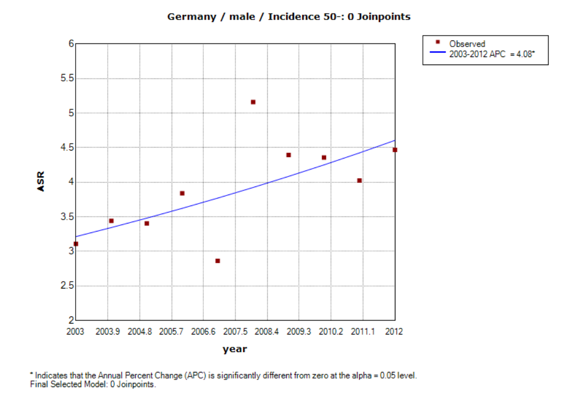

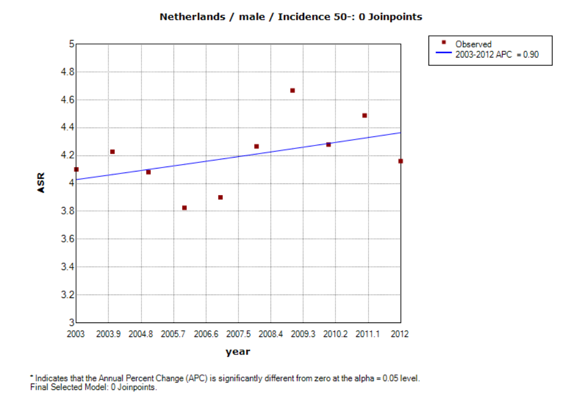

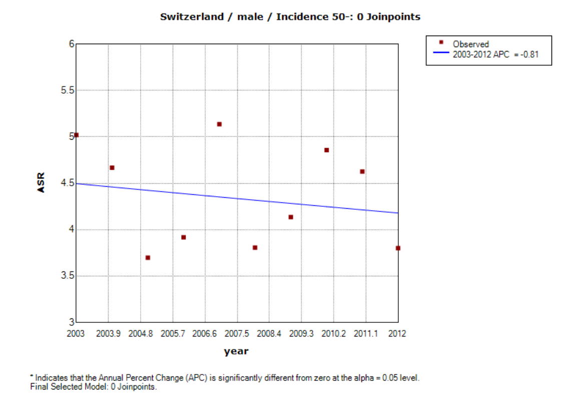

Southern Europe

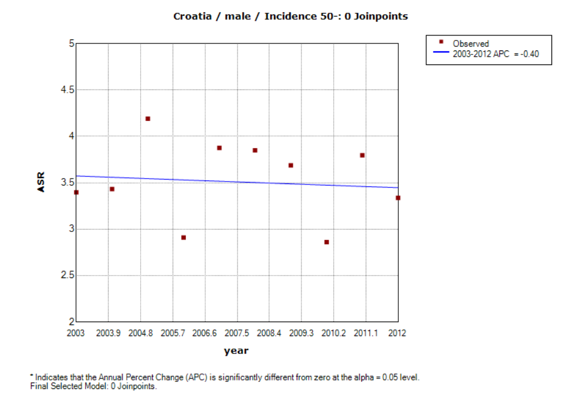

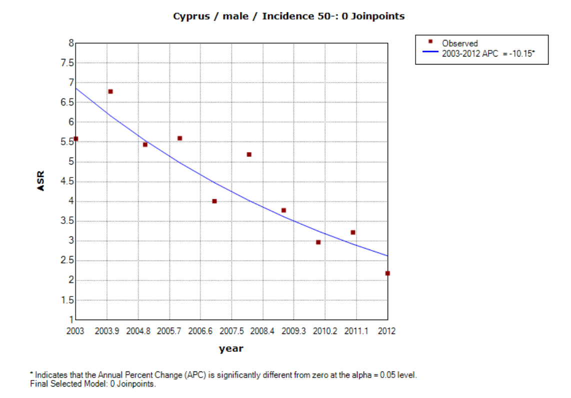

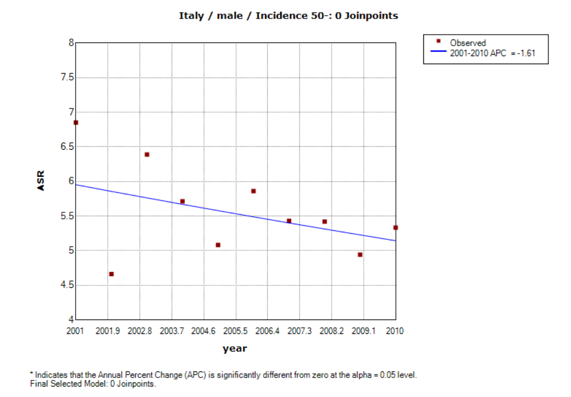

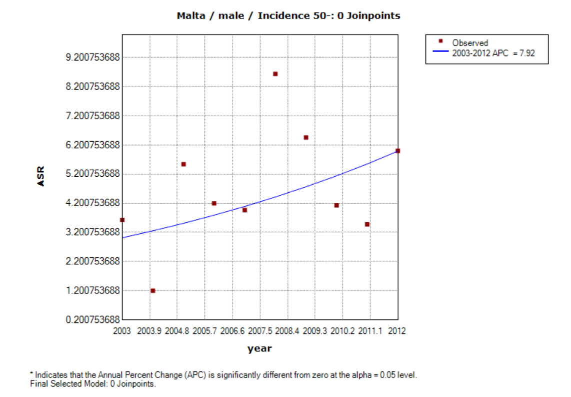

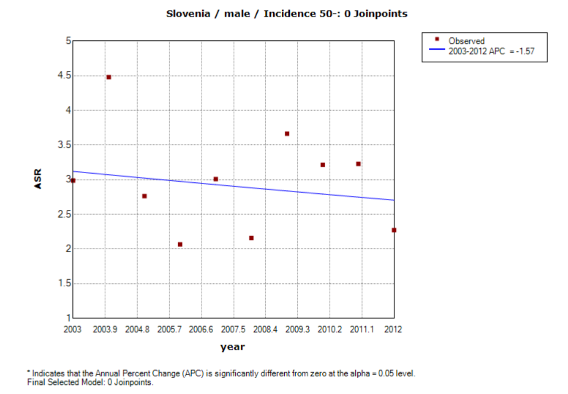

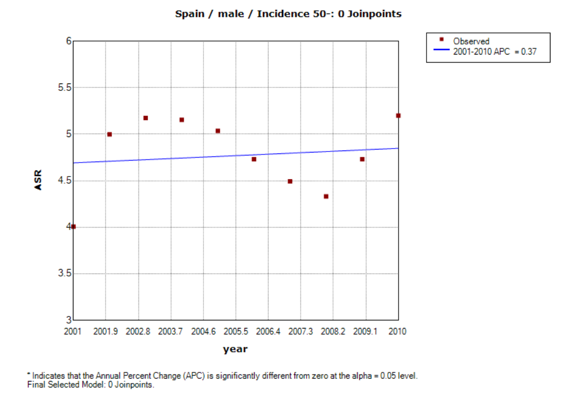

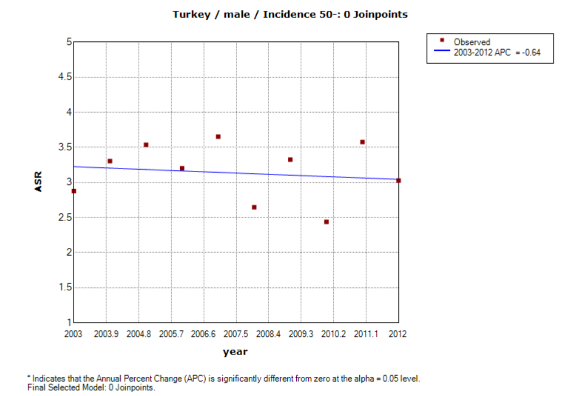

Eastern Europe

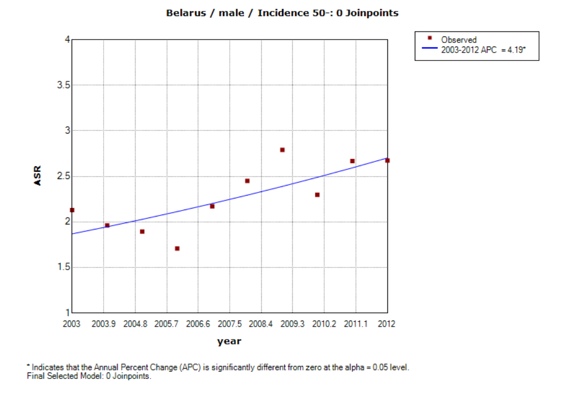

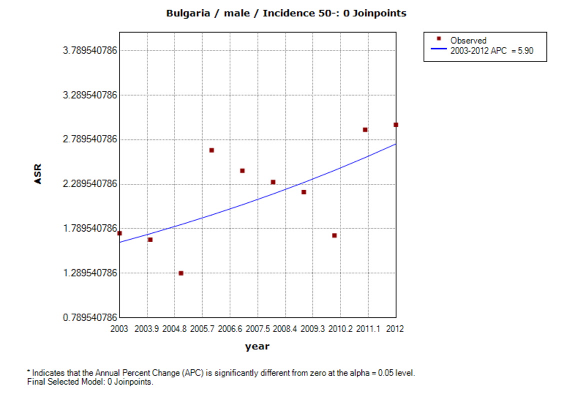

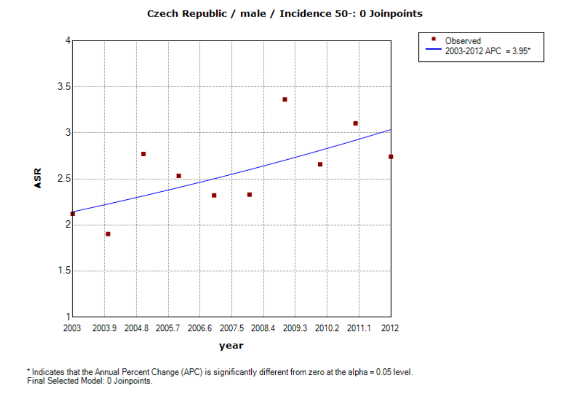

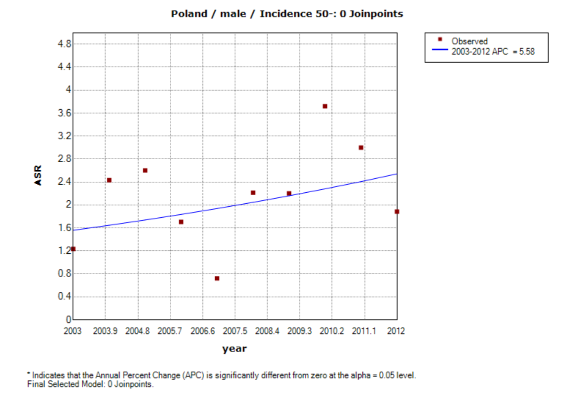

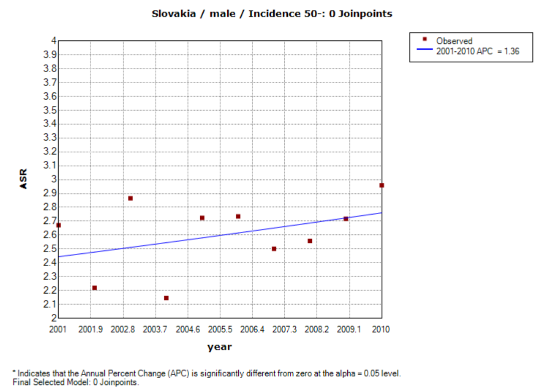
Slovakia / male / Incidence 50-: 0 Joinpoints
Observed
2001-2010 APC = 1.36
ASR
year
* Indicates that the Annual Percent Change (APC) is significantly different from zero at the alpha = 0.05 level.
Final Selected Model: 0 Joinpoints.

Africa

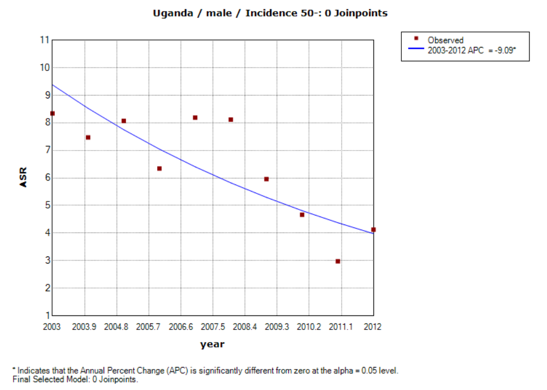
Uganda / male / Incidence 50-: 0 Joinpoints
Observed
2003-2012 APC = -9.09*
ASR
year
* Indicates that the Annual Percent Change (APC) is significantly different from zero at the alpha = 0.05 level.
Final Selected Model: 0 Joinpoints.

f.) Incidence female below 50 years old

Asia

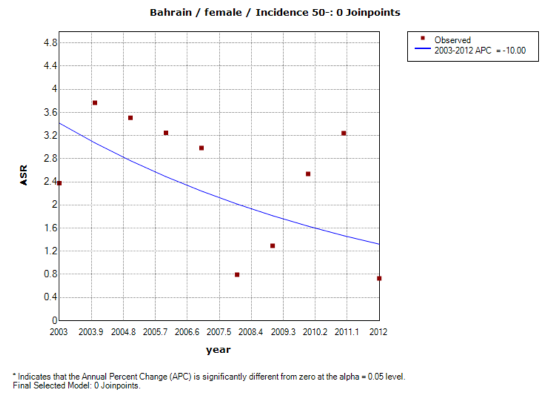

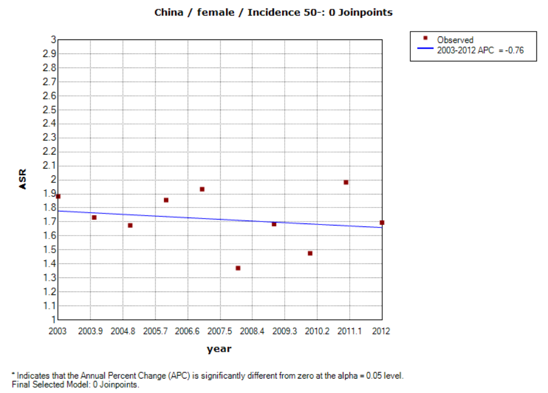

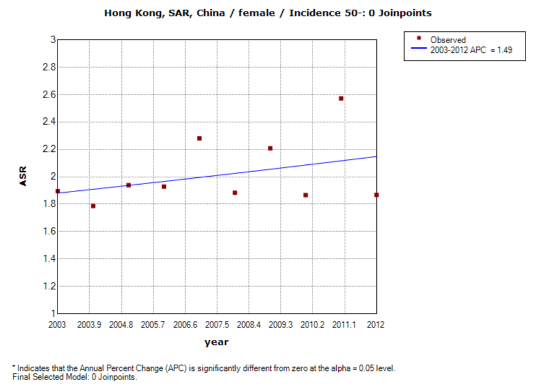

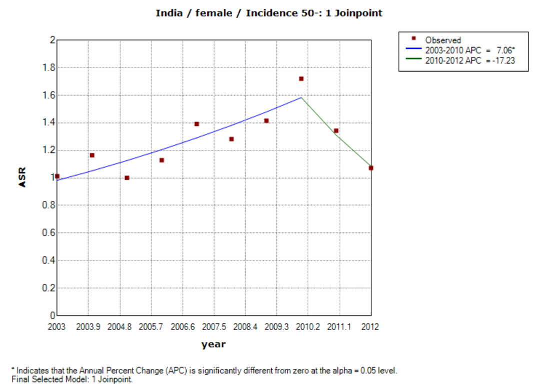

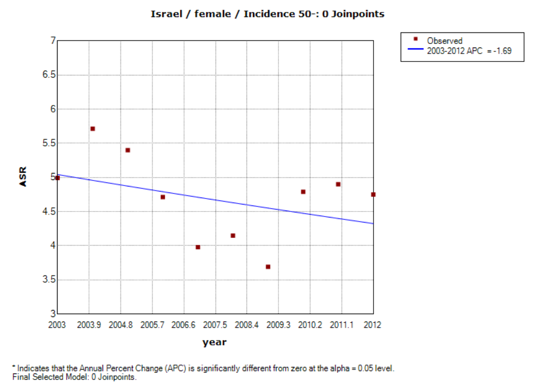

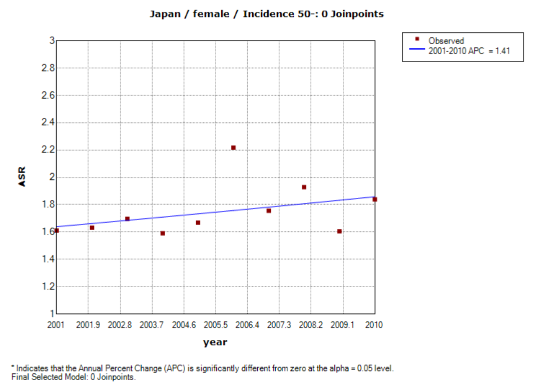

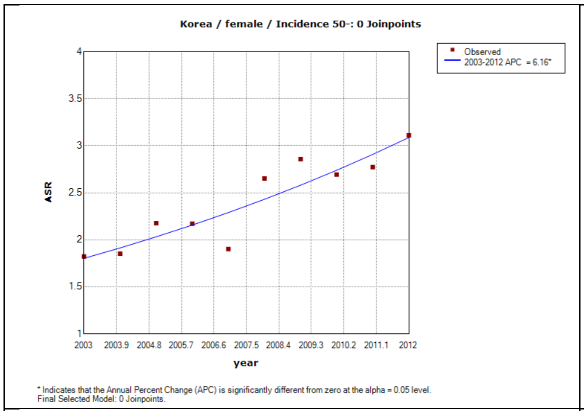
Korea / female / Incidence 50-: 0 Joinpoints
Observed
2003-2012 APC = 6.16*
ASR
year
* Indicates that the Annual Percent Change (APC) is significantly different from zero at the alpha = 0.05 level.
Final Selected Model: 0 Joinpoints.

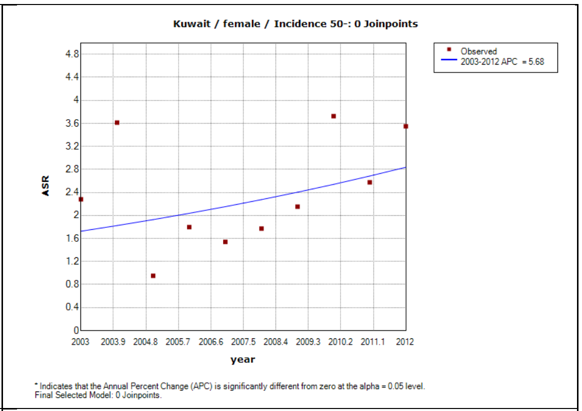
Kuwait / female / Incidence 50-: 0 Joinpoints
Observed
2003-2012 APC = 5.68
ASR
year
* Indicates that the Annual Percent Change (APC) is significantly different from zero at the alpha = 0.05 level.
Final Selected Model: 0 Joinpoints.

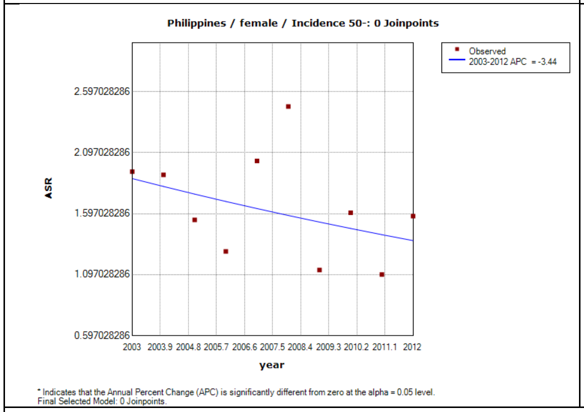
Philippines / female / Incidence 50-: 0 Joinpoints
Observed
2003-2012 APC = -3.44
ASR
year
* Indicates that the Annual Percent Change (APC) is significantly different from zero at the alpha = 0.05 level.
Final Selected Model: 0 Joinpoints.

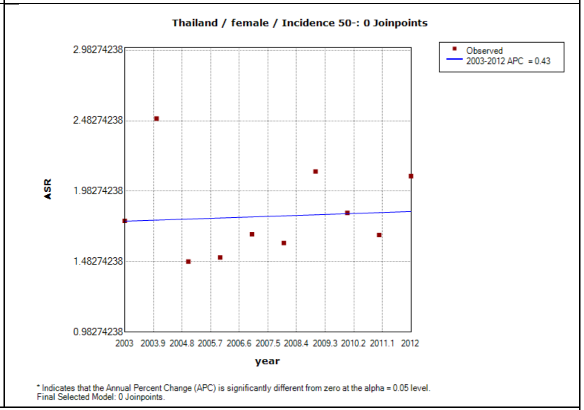
Thailand / female / Incidence 50-: 0 Joinpoints
Observed
2003-2012 APC = 0.43
ASR
year
* Indicates that the Annual Percent Change (APC) is significantly different from zero at the alpha = 0.05 level.
Final Selected Model: 0 Joinpoints.

Oceania

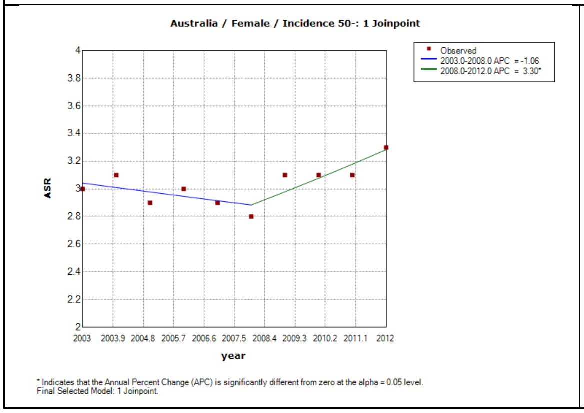
Australia / Female / Incidence 50-: 1 Joinpoint
Observed
2003.0-2008.0 APC = -1.06
2008.0-2012.0 APC = 3.30*
ASR
year
* Indicates that the Annual Percent Change (APC) is significantly different from zero at the alpha = 0.05 level.
Final Selected Model: 1 Joinpoint.

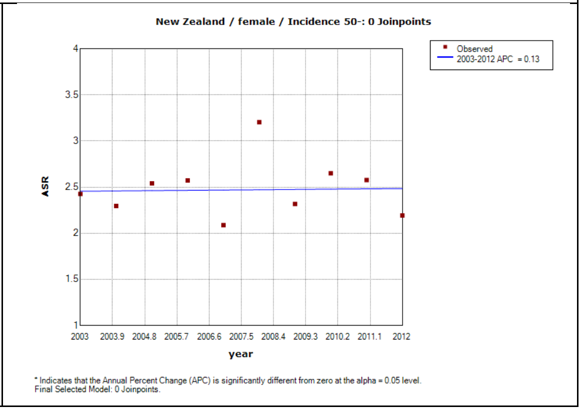
New Zealand / female / Incidence 50-: 0 Joinpoints
Observed
2003-2012 APC = 0.13
ASR
year
* Indicates that the Annual Percent Change (APC) is significantly different from zero at the alpha = 0.05 level.
Final Selected Model: 0 Joinpoints.

Northern America

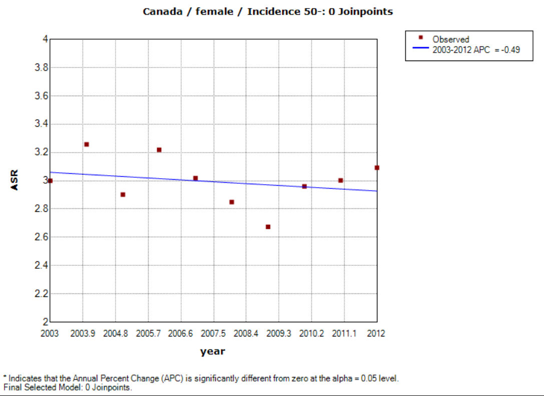

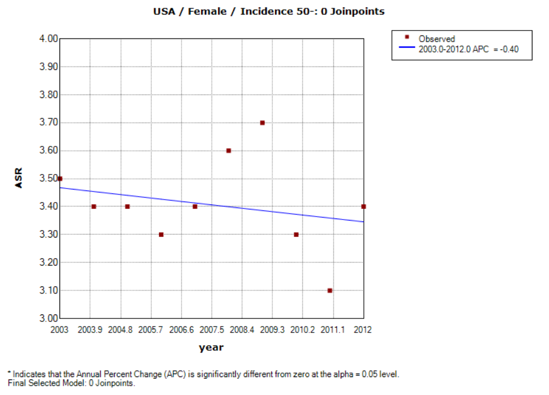

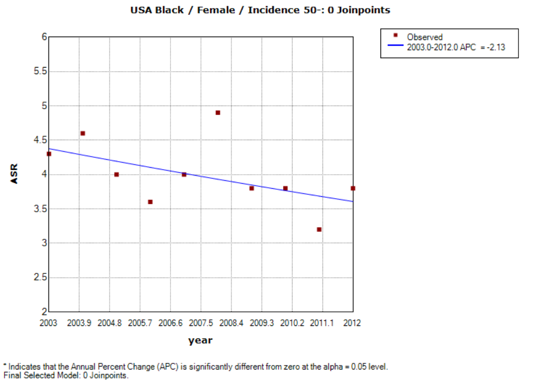

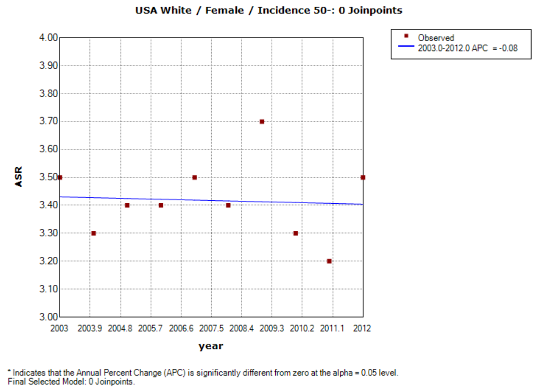

Southern America

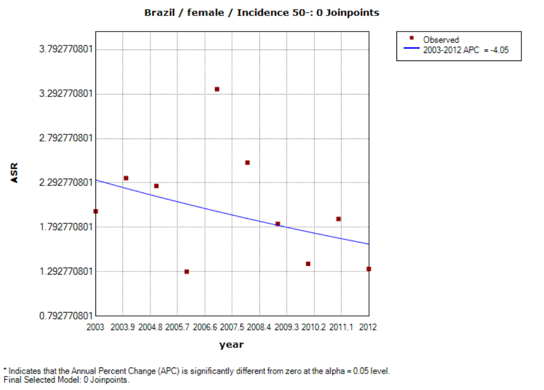

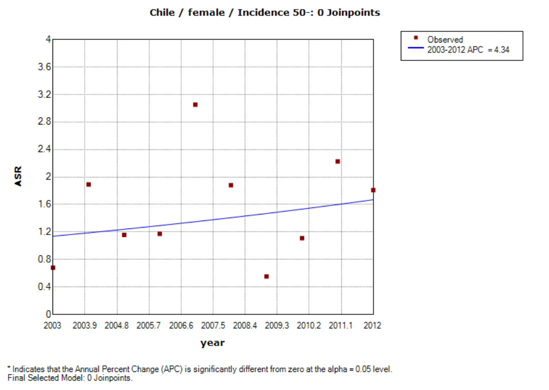

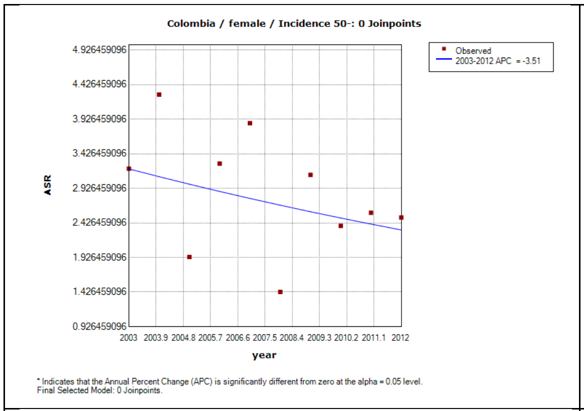

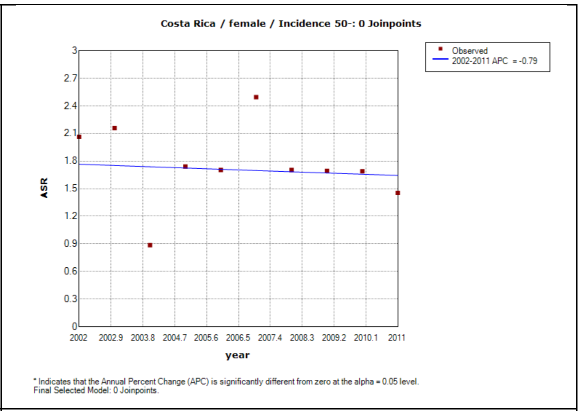

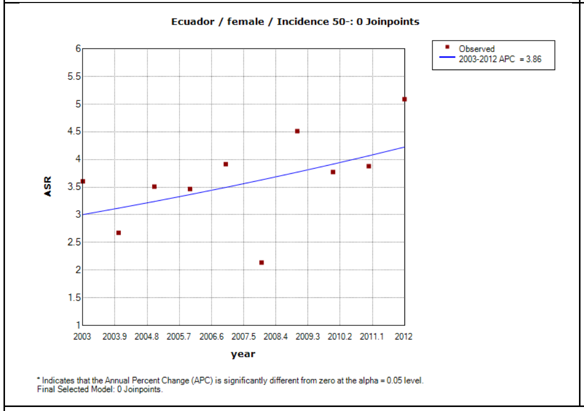

Northern Europe

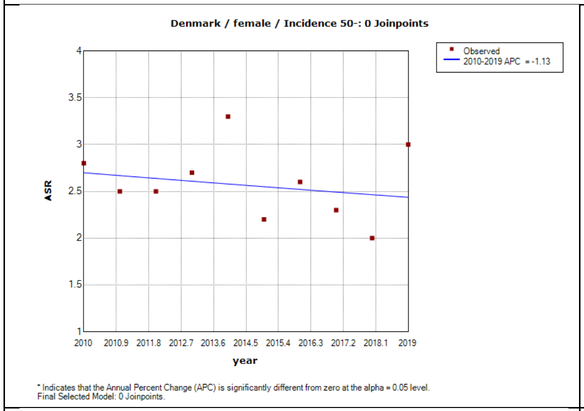

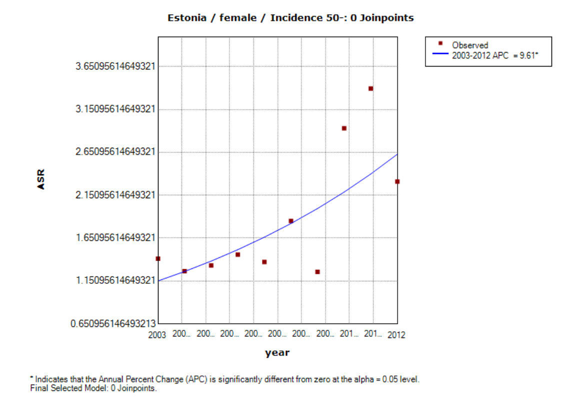

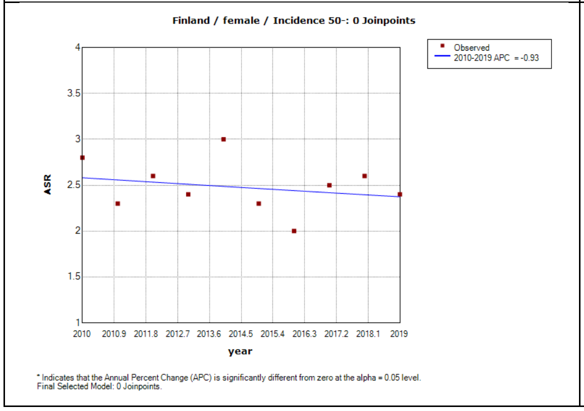

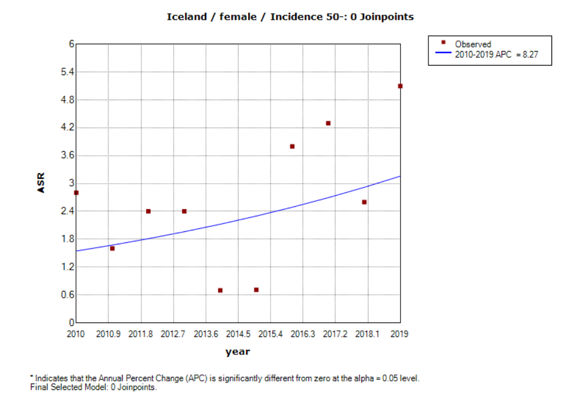

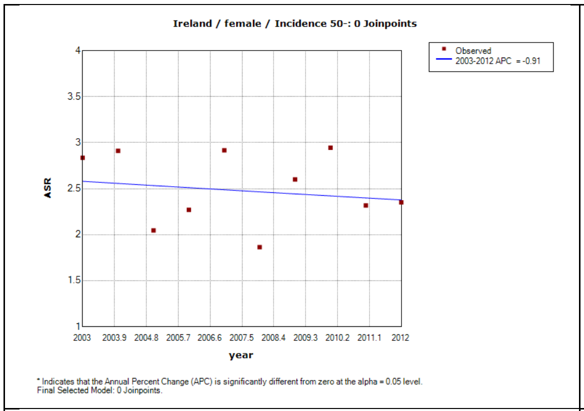

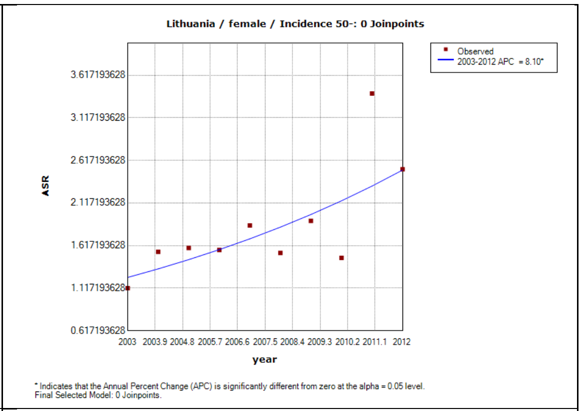

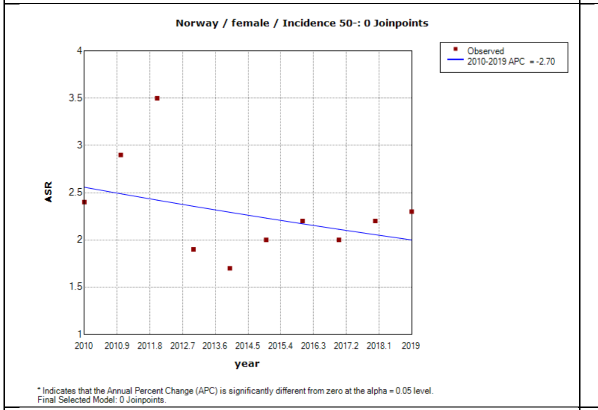

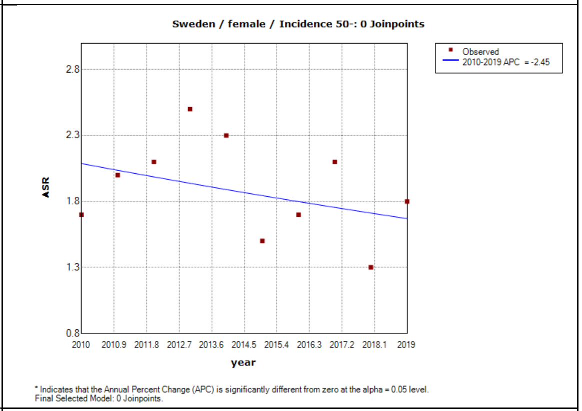

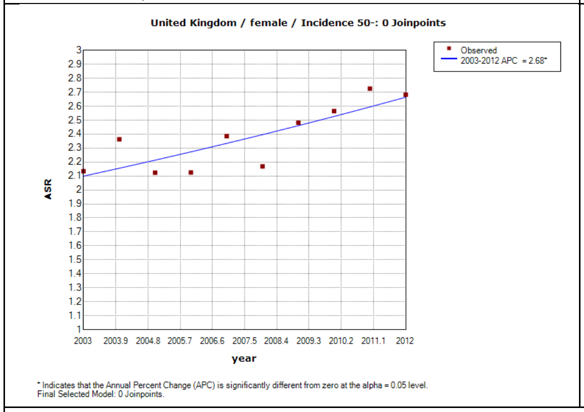

Western Europe

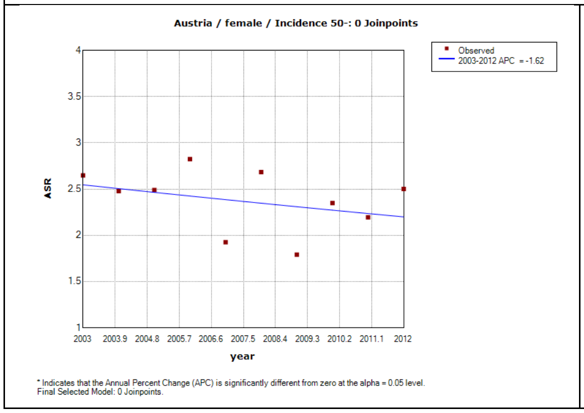

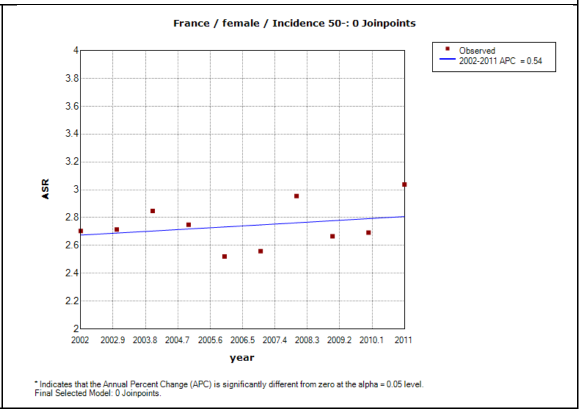

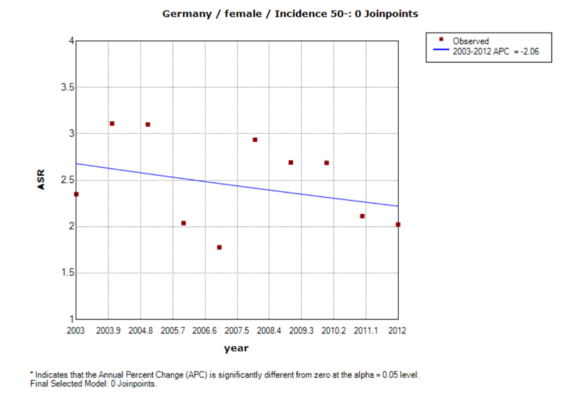

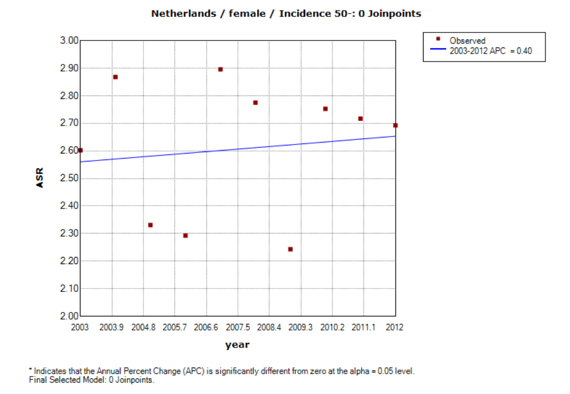

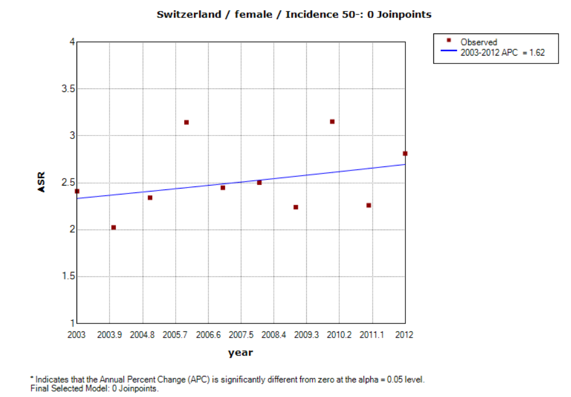

Southern Europe

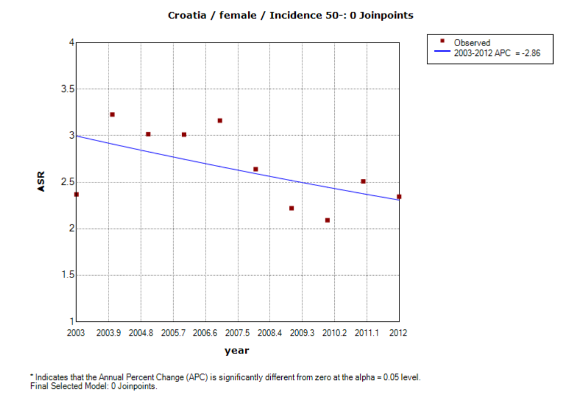

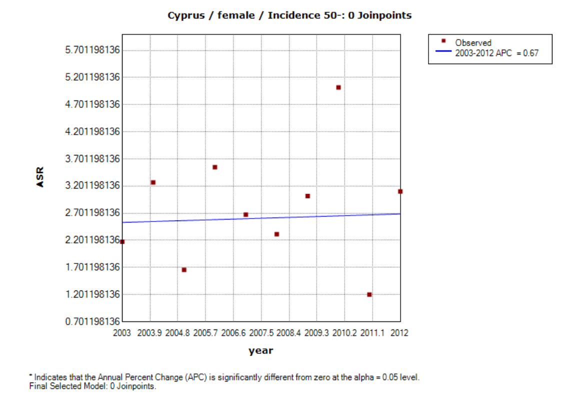

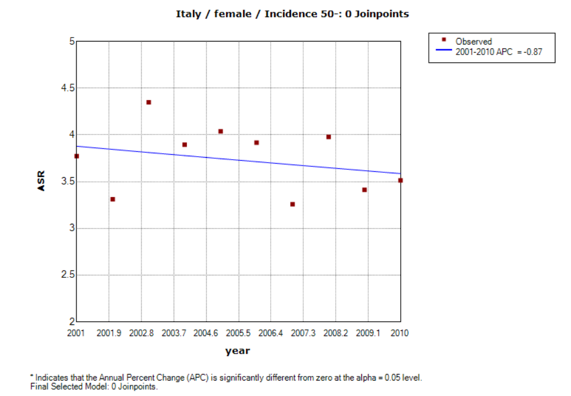

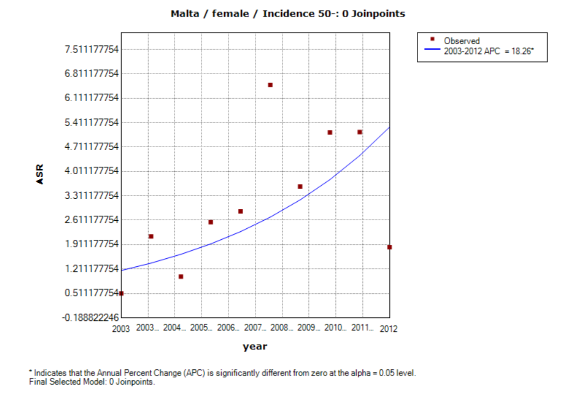

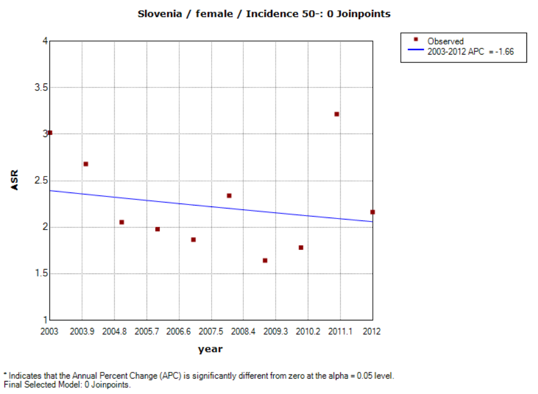

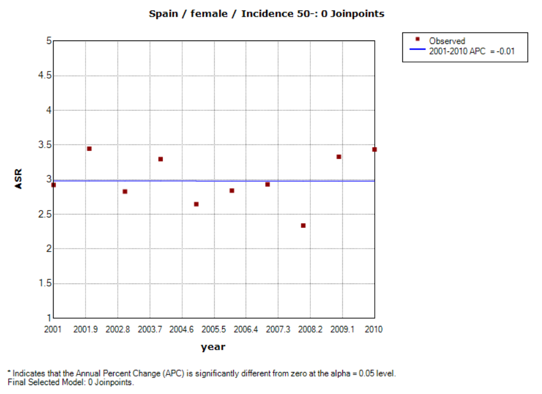

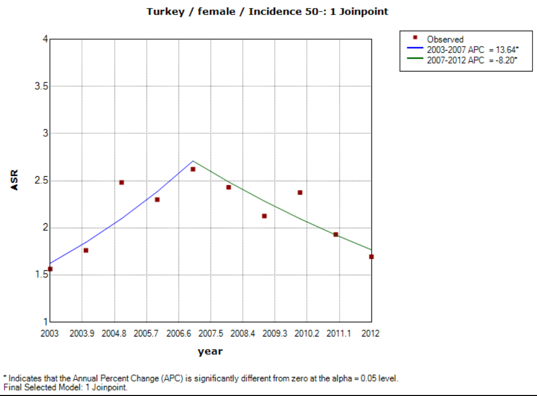

Eastern Europe

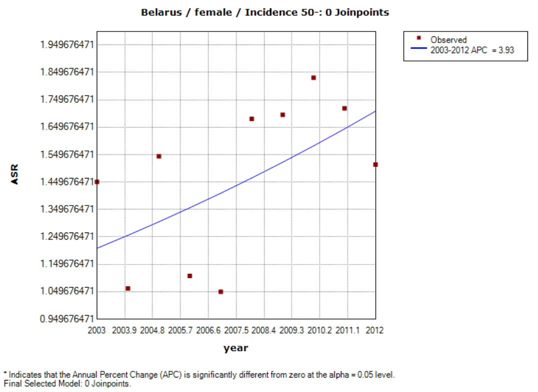

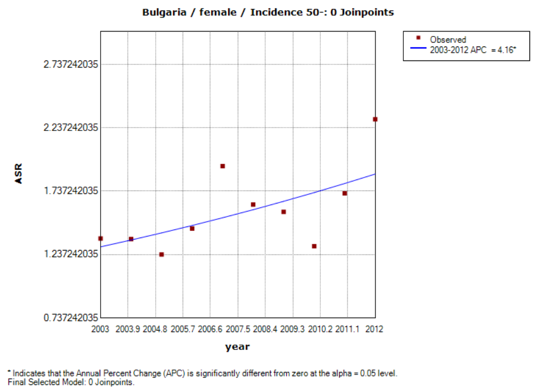

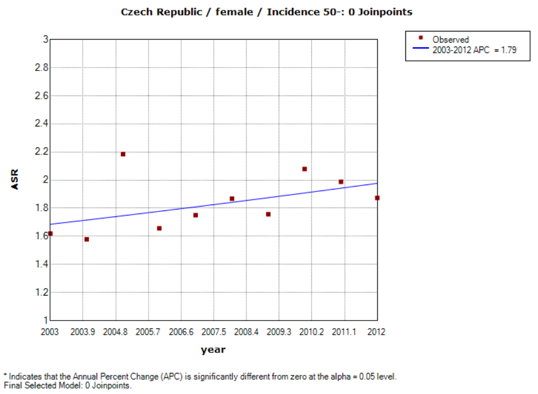

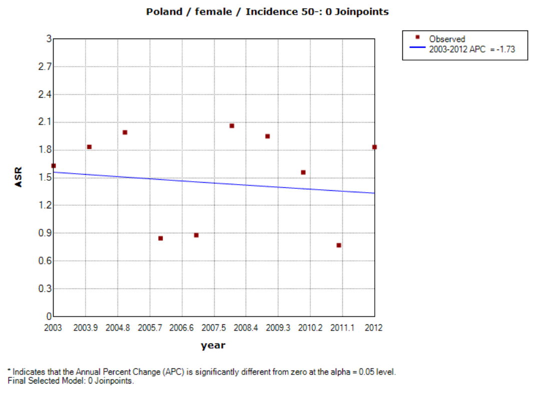

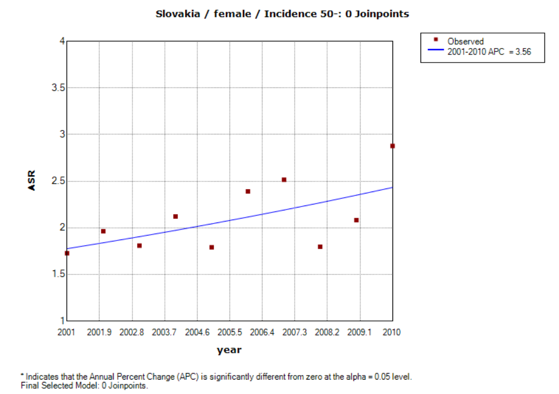
Slovakia / female / Incidence 50-: 0 Joinpoints
Observed
2001-2010 APC = 3.56
ASR
year
2001 2001.9 2002.8 2003.7 2004.6 2005.5 2006.4 2007.3 2008.2 2009.1 2010
1 1.5 2 2.5 3 3.5 4
* Indicates that the Annual Percent Change (APC) is significantly different from zero at the alpha = 0.05 level.
Final Selected Model: 0 Joinpoints.

Africa

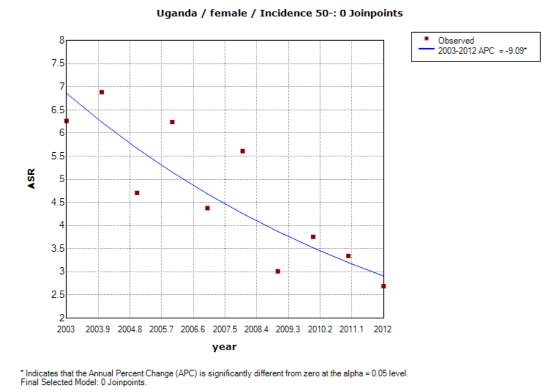
Uganda / female / Incidence 50-: 0 Joinpoints
Observed
2003-2012 APC = -9.09*
ASR
year
2003 2003.9 2004.8 2005.7 2006.6 2007.5 2008.4 2009.3 2010.2 2011.1 2012
2 2.5 3 3.5 4 4.5 5 5.5 6 6.5 7 7.5 8
* Indicates that the Annual Percent Change (APC) is significantly different from zero at the alpha = 0.05 level.
Final Selected Model: 0 Joinpoints.

g.) Incidence male above 50 years old

Asia

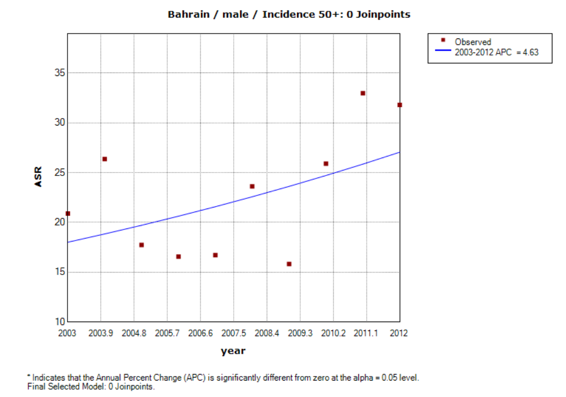

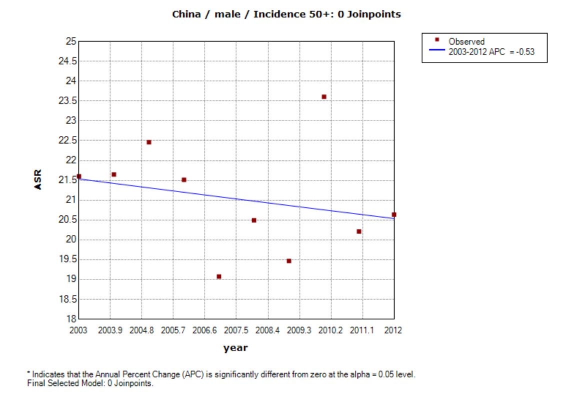

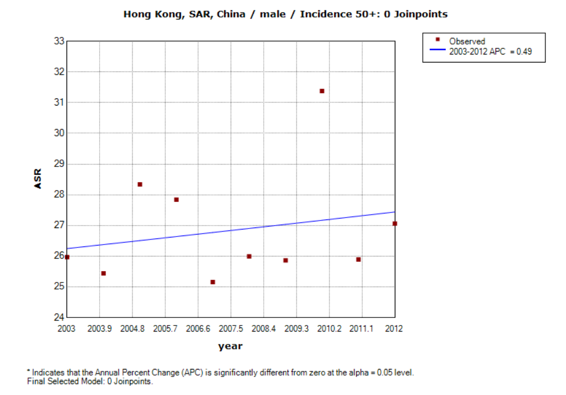

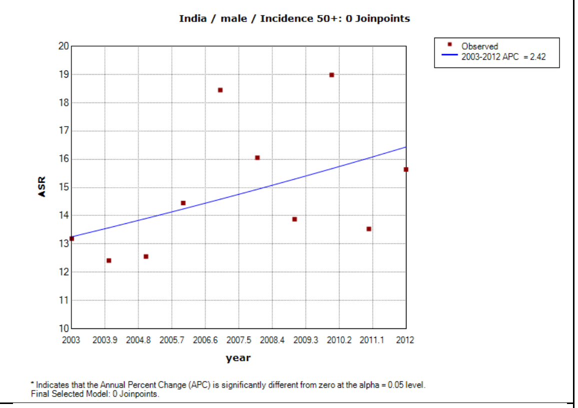

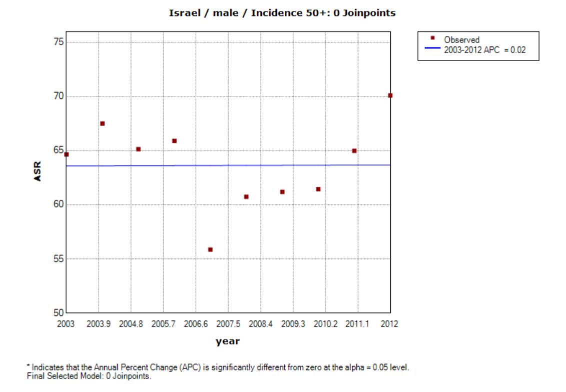

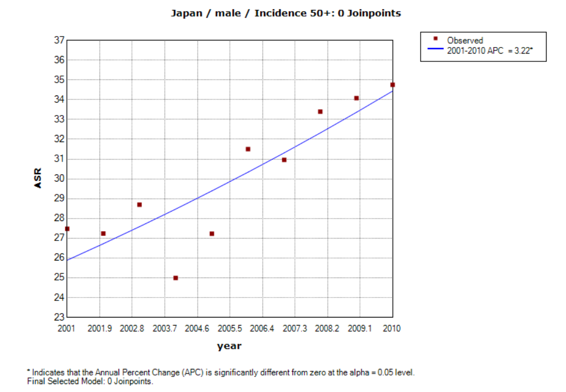

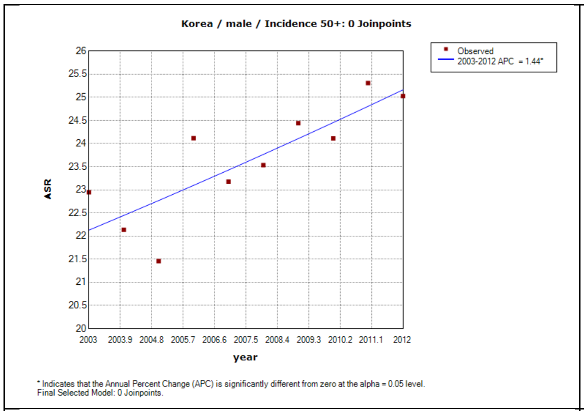
Korea / male / Incidence 50+: 0 Joinpoints
Observed
2003-2012 APC = 1.44*
ASR
year
* Indicates that the Annual Percent Change (APC) is significantly different from zero at the alpha = 0.05 level.
Final Selected Model: 0 Joinpoints.

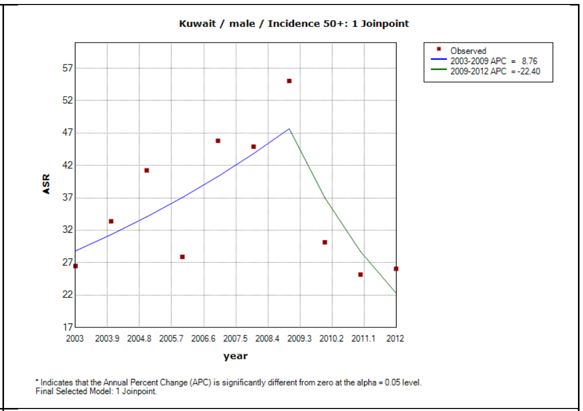
Kuwait / male / Incidence 50+: 1 Joinpoint
Observed
2003-2009 APC = 8.76
2009-2012 APC = -22.40
ASR
year
* Indicates that the Annual Percent Change (APC) is significantly different from zero at the alpha = 0.05 level.
Final Selected Model: 1 Joinpoint.

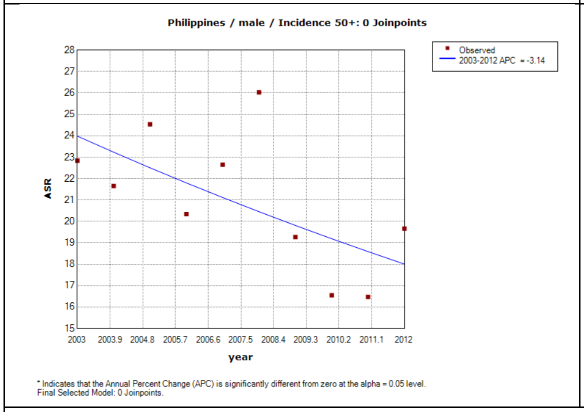
Philippines / male / Incidence 50+: 0 Joinpoints
Observed
2003-2012 APC = -3.14
ASR
year
* Indicates that the Annual Percent Change (APC) is significantly different from zero at the alpha = 0.05 level.
Final Selected Model: 0 Joinpoints.

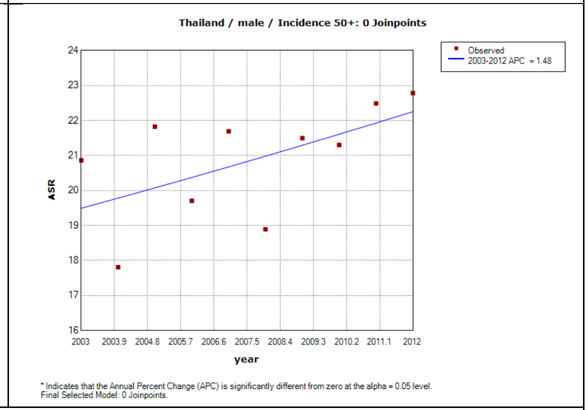
Thailand / male / Incidence 50+: 0 Joinpoints
Observed
2003-2012 APC = 1.48
ASR
year
* Indicates that the Annual Percent Change (APC) is significantly different from zero at the alpha = 0.05 level.
Final Selected Model: 0 Joinpoints.

Oceania

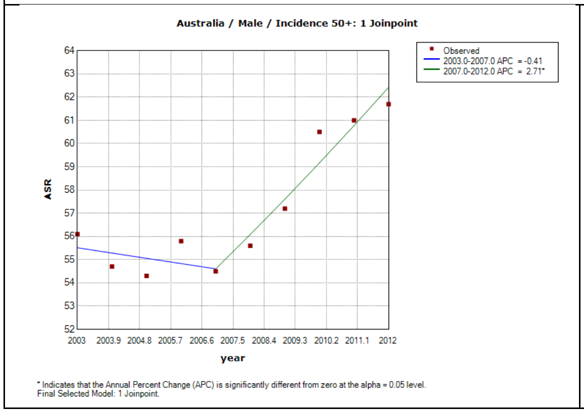
Australia / Male / Incidence 50+: 1 Joinpoint
Observed
2003.0-2007.0 APC = -0.41
2007.0-2012.0 APC = 2.71*
ASR
year
* Indicates that the Annual Percent Change (APC) is significantly different from zero at the alpha = 0.05 level.
Final Selected Model: 1 Joinpoint.

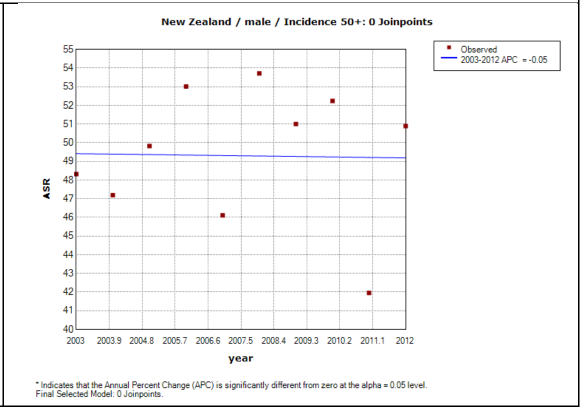
New Zealand / male / Incidence 50+: 0 Joinpoints
Observed
2003-2012 APC = -0.05
ASR
year
* Indicates that the Annual Percent Change (APC) is significantly different from zero at the alpha = 0.05 level.
Final Selected Model: 0 Joinpoints.

Northern America

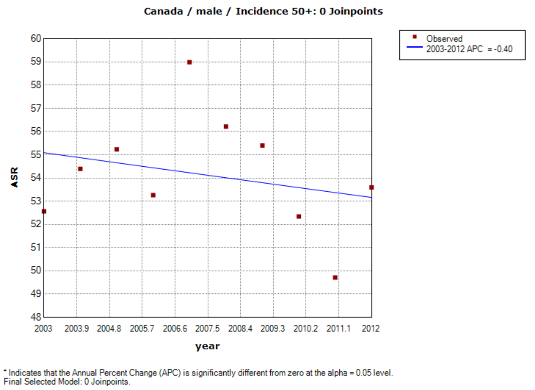

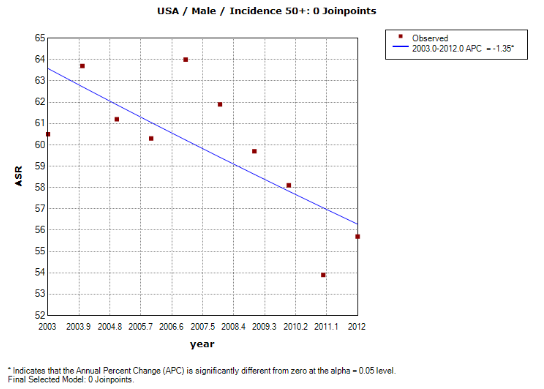

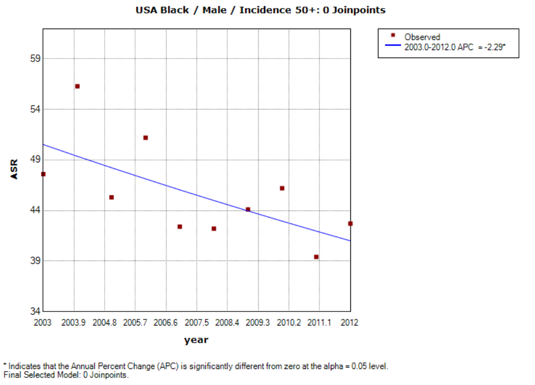

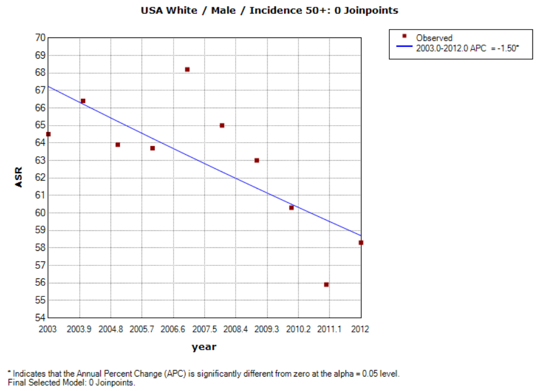

Southern America

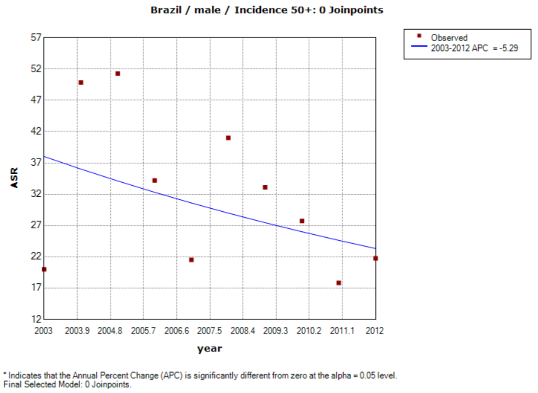

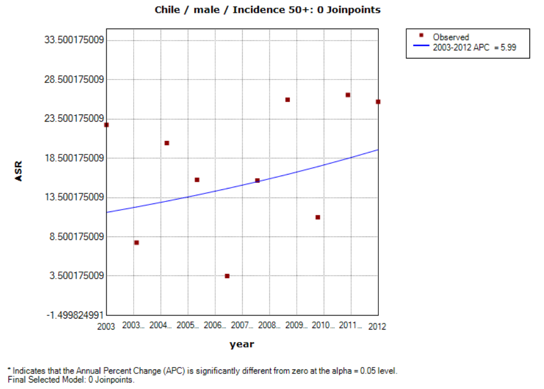

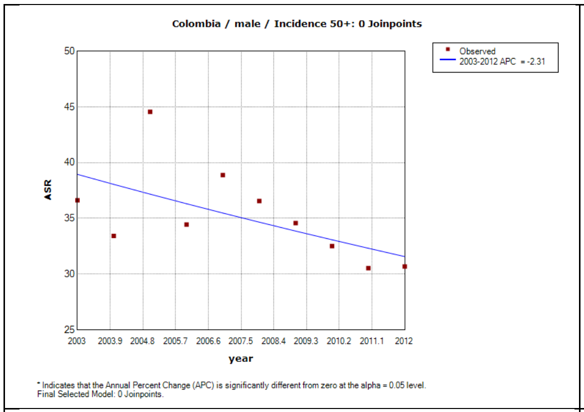

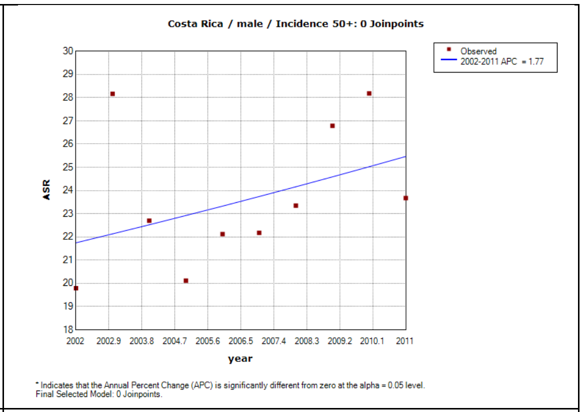

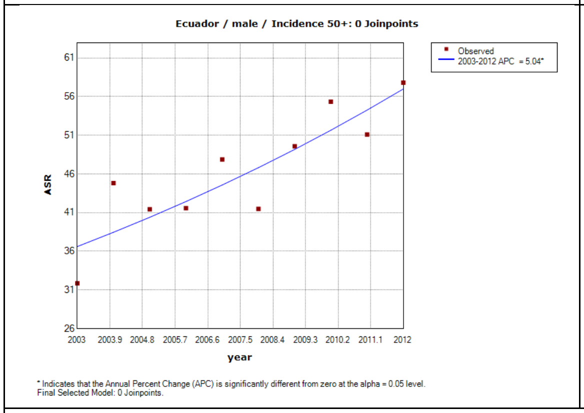

Northern Europe

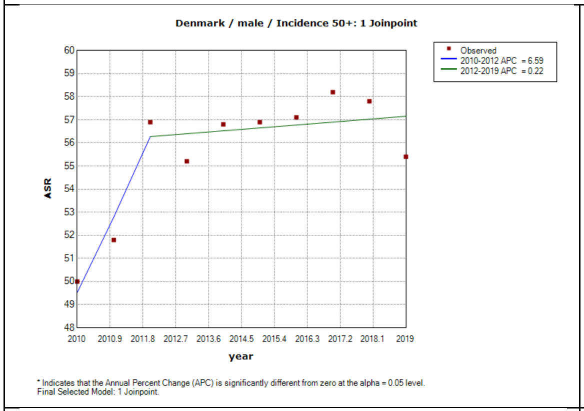

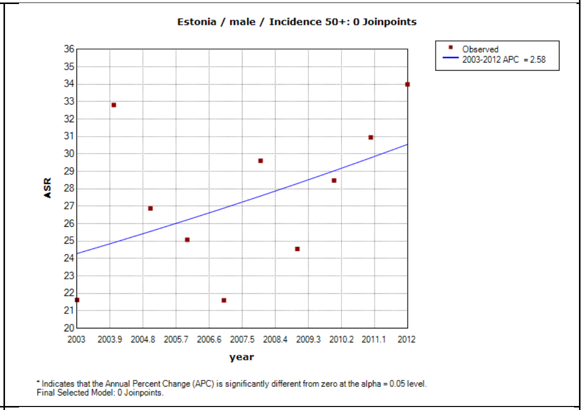

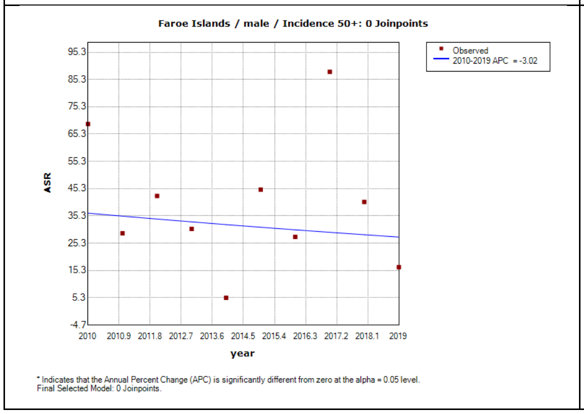

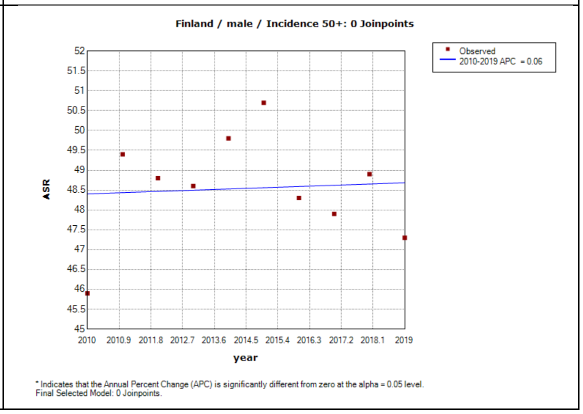

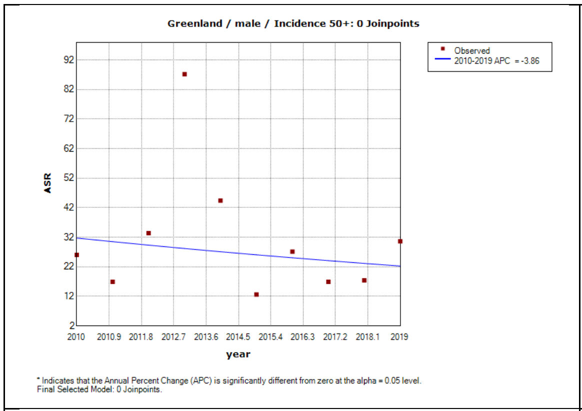

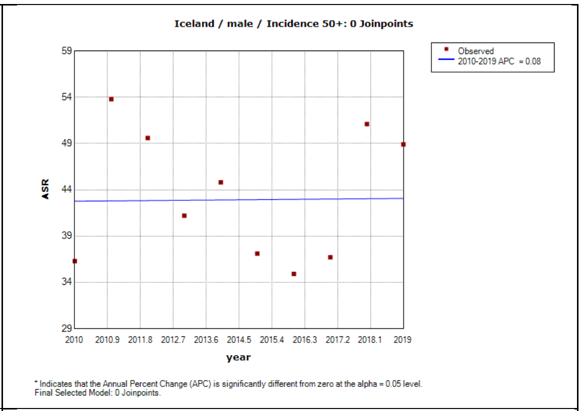

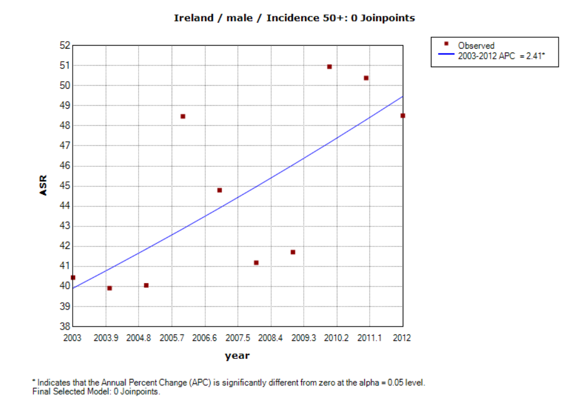

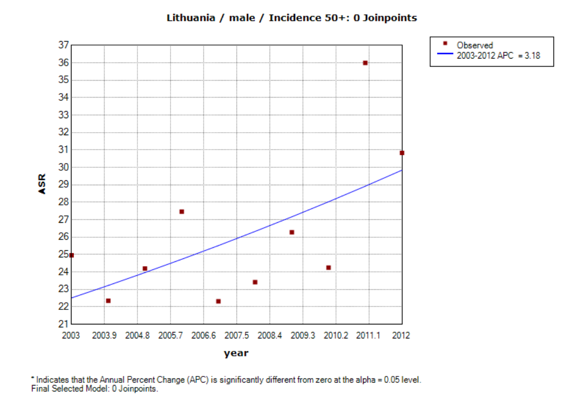

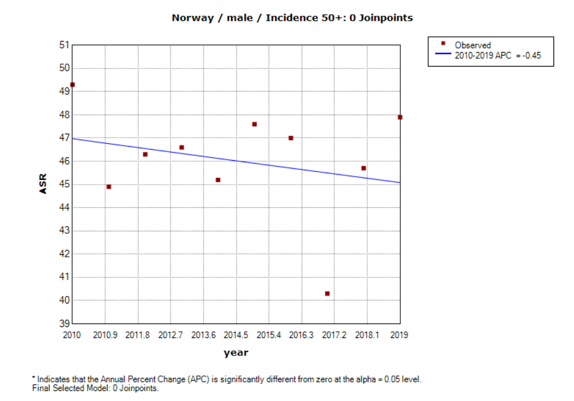

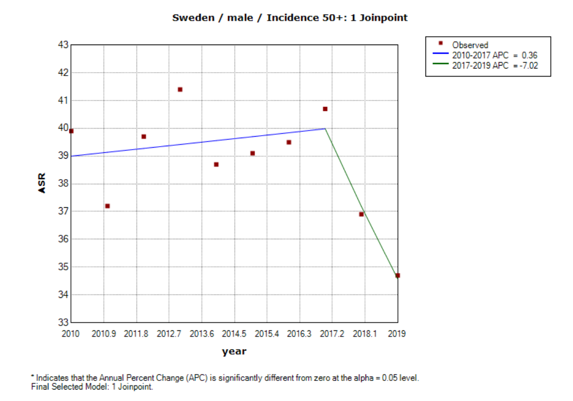

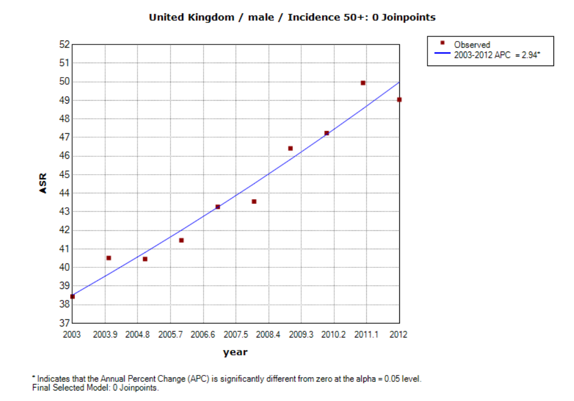

Western Europe

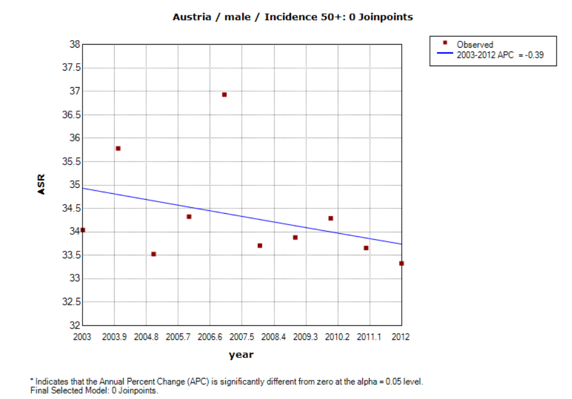

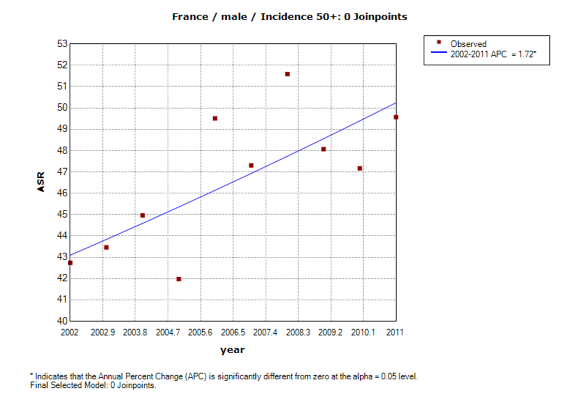

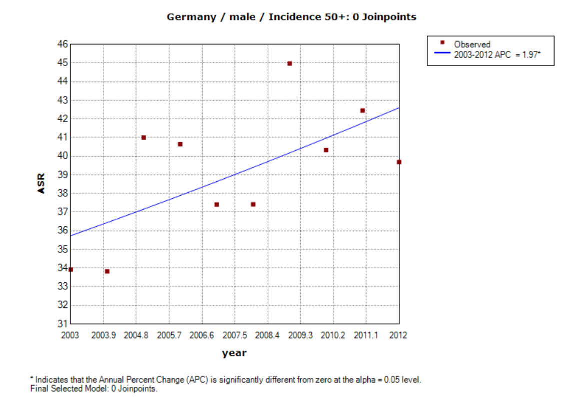

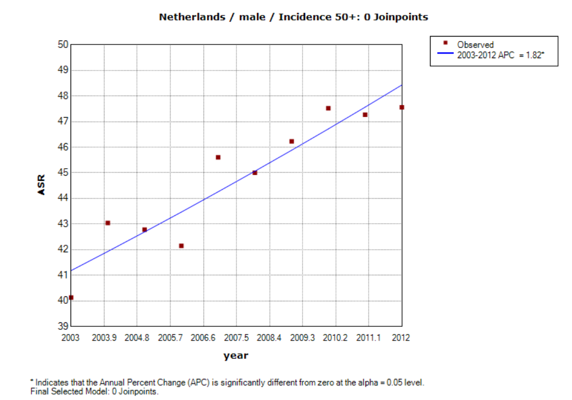

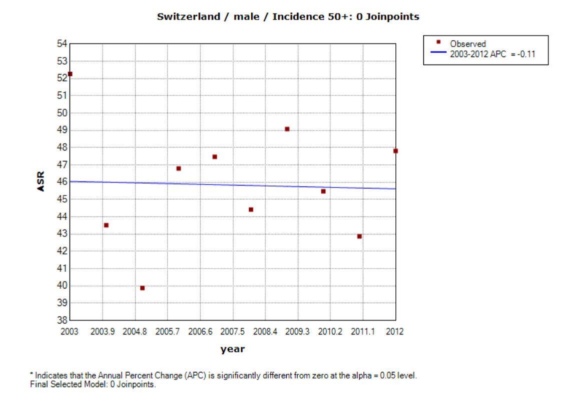

Southern Europe

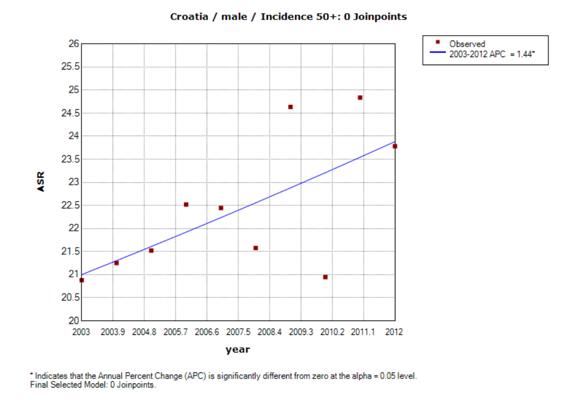

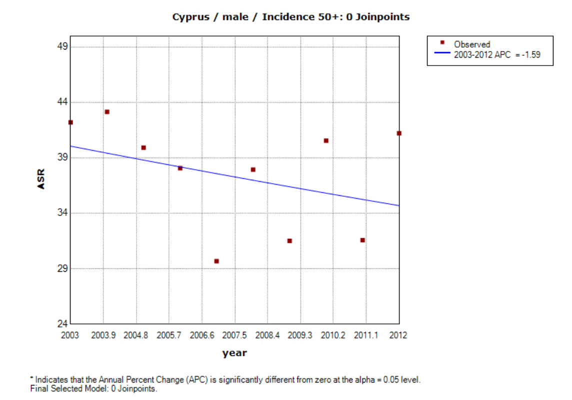

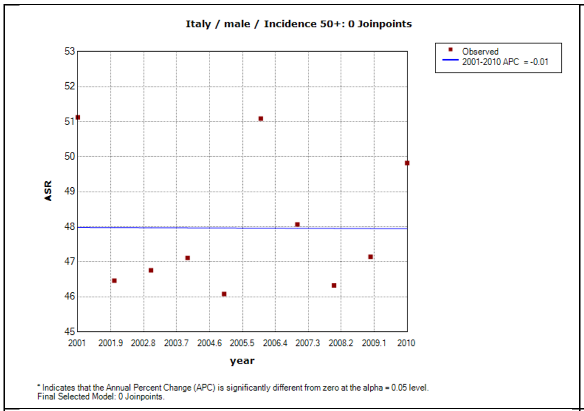

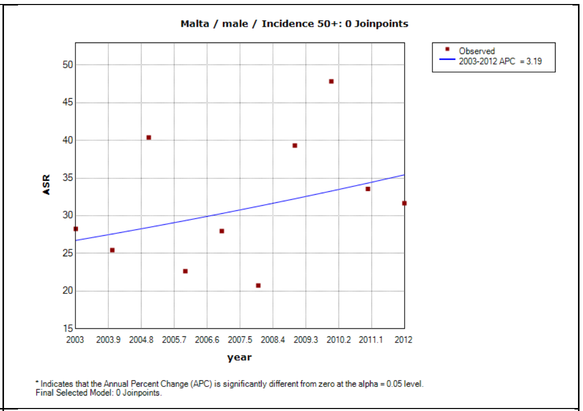

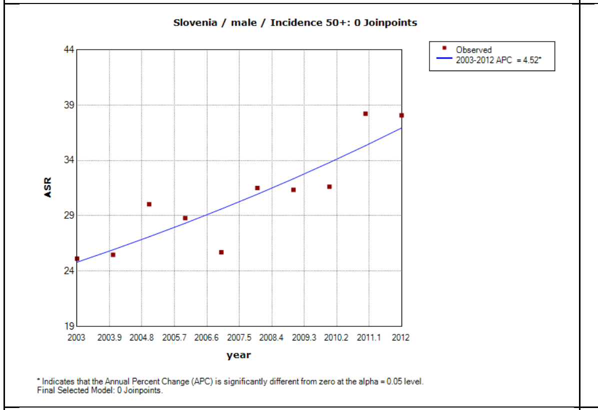

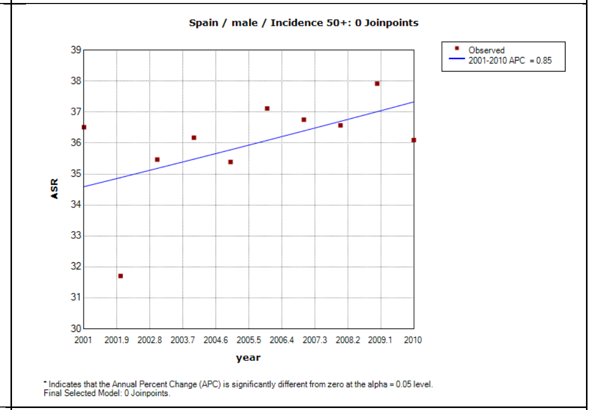

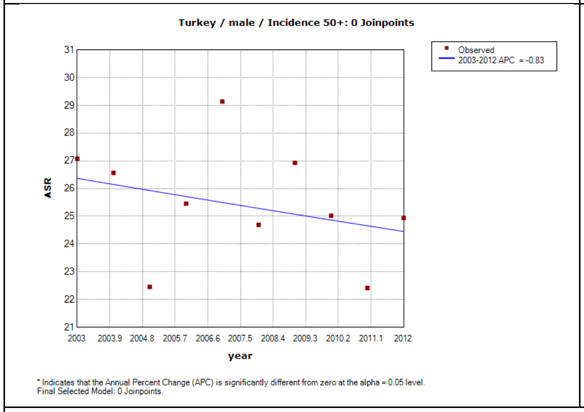

Eastern Europe

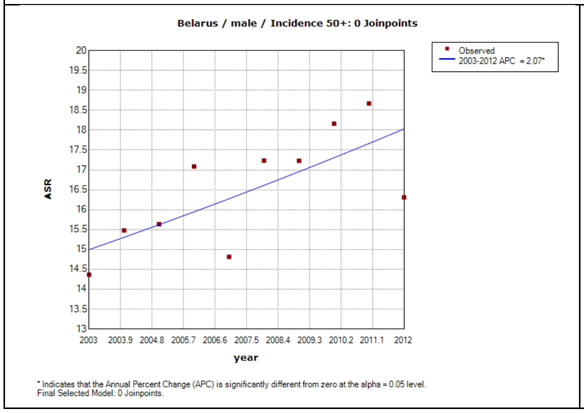

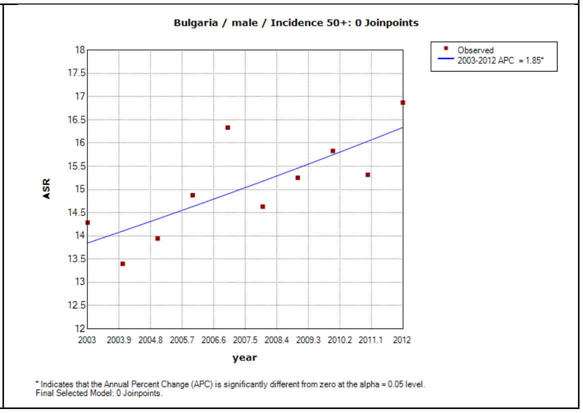

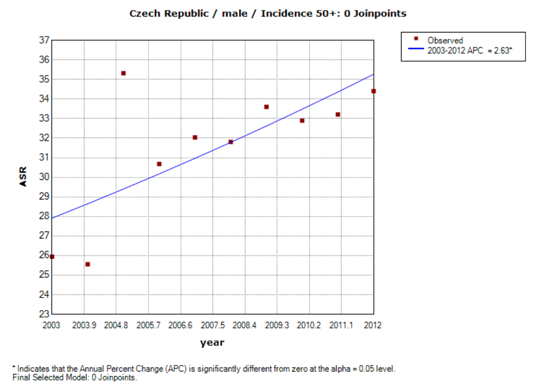
Czech Republic / male / Incidence 50+: 0 Joinpoints
Observed
2003-2012 APC = 2.63*
ASR
year
* Indicates that the Annual Percent Change (APC) is significantly different from zero at the alpha = 0.05 level.
Final Selected Model: 0 Joinpoints.

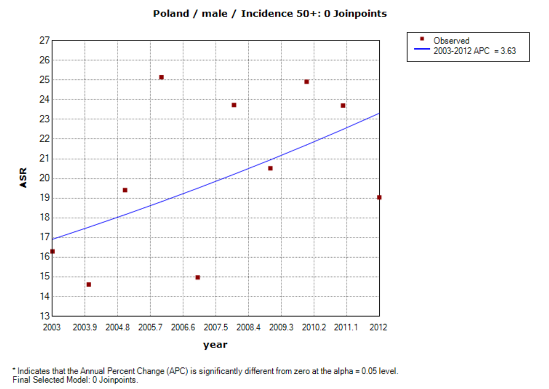
Poland / male / Incidence 50+: 0 Joinpoints
Observed
2003-2012 APC = 3.63
ASR
year
* Indicates that the Annual Percent Change (APC) is significantly different from zero at the alpha = 0.05 level.
Final Selected Model: 0 Joinpoints.

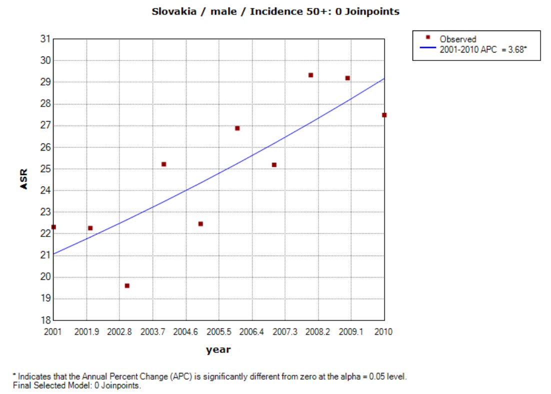
Slovakia / male / Incidence 50+: 0 Joinpoints
Observed
2001-2010 APC = 3.68*
ASR
year
* Indicates that the Annual Percent Change (APC) is significantly different from zero at the alpha = 0.05 level.
Final Selected Model: 0 Joinpoints.

Africa

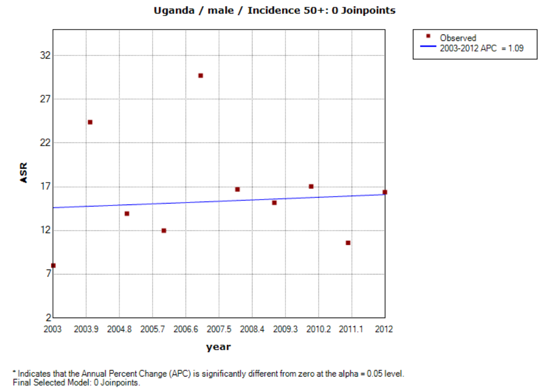
Uganda / male / Incidence 50+: 0 Joinpoints
Observed
2003-2012 APC = 1.09
ASR
year
* Indicates that the Annual Percent Change (APC) is significantly different from zero at the alpha = 0.05 level.
Final Selected Model: 0 Joinpoints.

h.) Incidence female above 50 years old

Asia

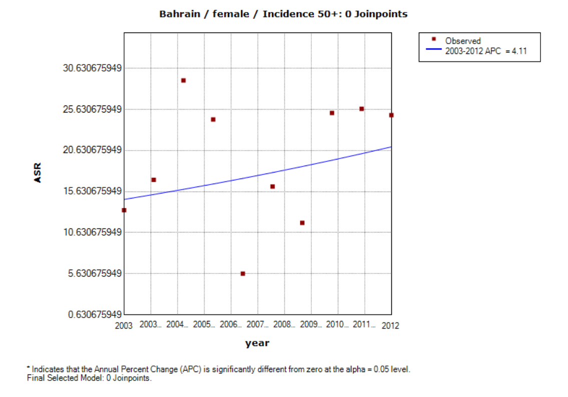

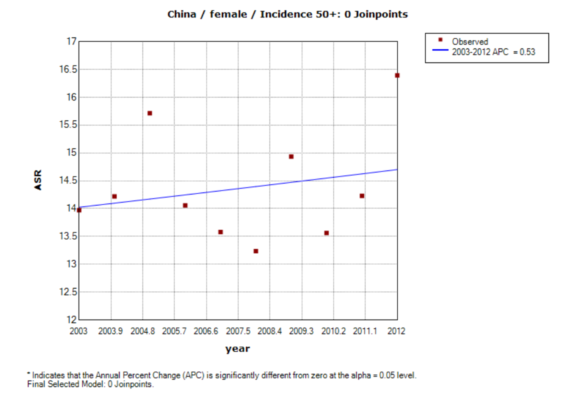

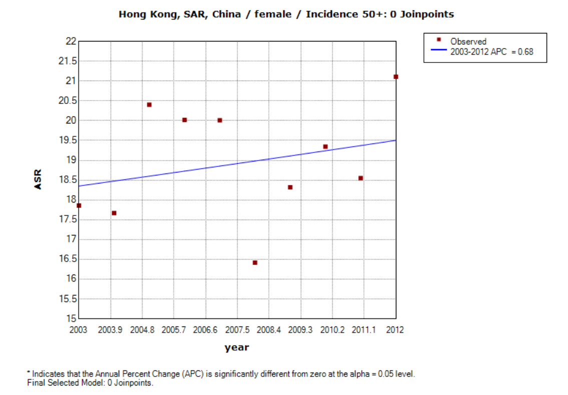

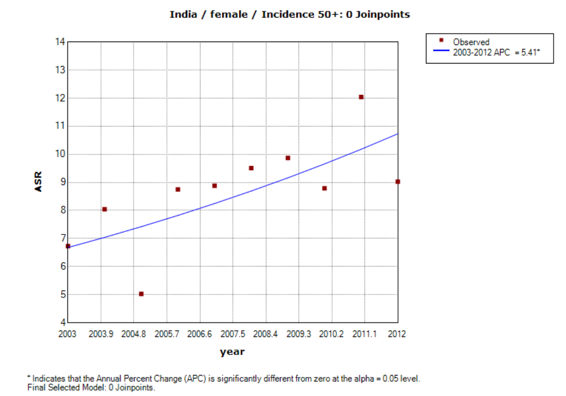

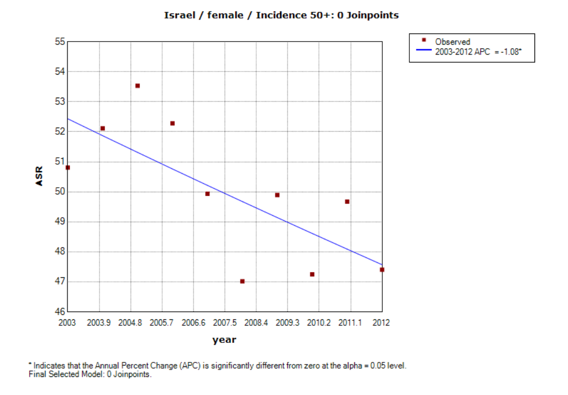

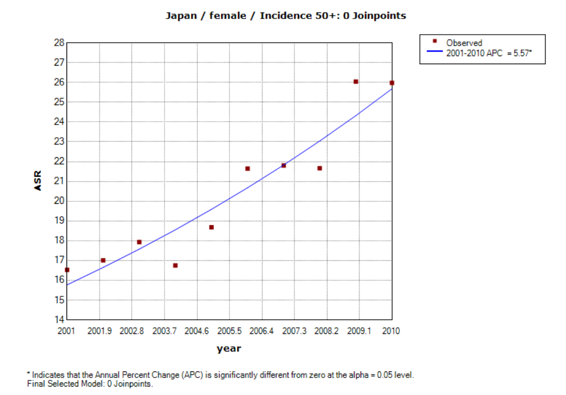

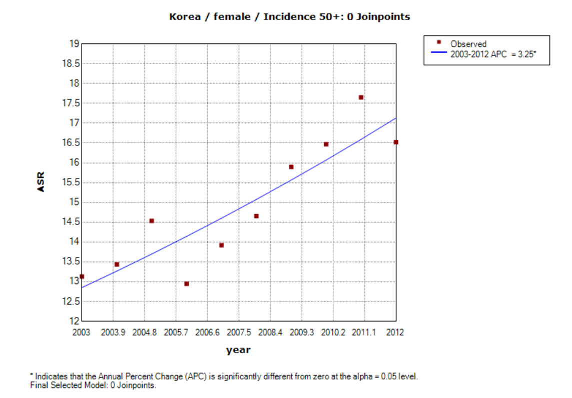
Korea / female / Incidence 50+: 0 Joinpoints
Observed
2003-2012 APC = 3.25*
ASR
year
19 18.5 18 17.5 17 16.5 16 15.5 15 14.5 14 13.5 13 12.5 12
2003 2003.9 2004.8 2005.7 2006.6 2007.5 2008.4 2009.3 2010.2 2011.1 2012
* Indicates that the Annual Percent Change (APC) is significantly different from zero at the alpha = 0.05 level.
Final Selected Model: 0 Joinpoints.

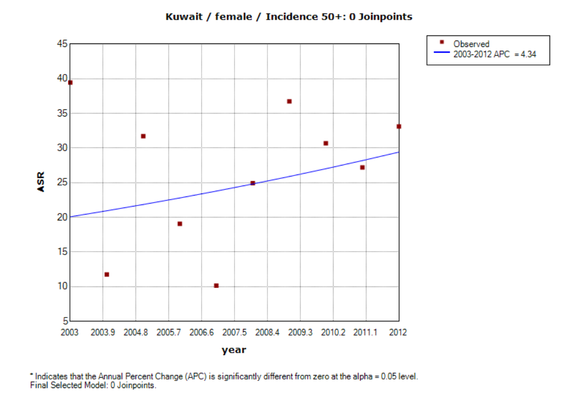
Kuwait / female / Incidence 50+: 0 Joinpoints
Observed
2003-2012 APC = 4.34
ASR
year
45 40 35 30 25 20 15 10 5
2003 2003.9 2004.8 2005.7 2006.6 2007.5 2008.4 2009.3 2010.2 2011.1 2012
* Indicates that the Annual Percent Change (APC) is significantly different from zero at the alpha = 0.05 level.
Final Selected Model: 0 Joinpoints.

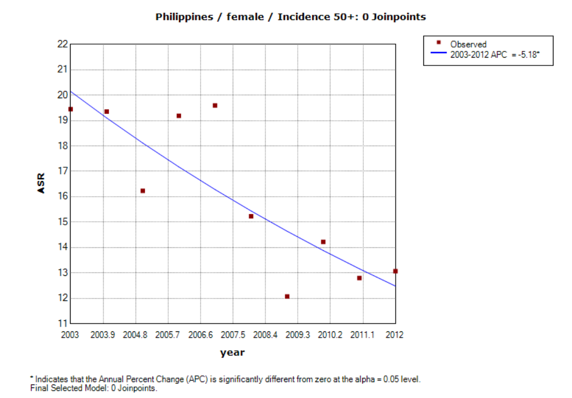
Philippines / female / Incidence 50+: 0 Joinpoints
Observed
2003-2012 APC = -5.18*
ASR
year
22 21 20 19 18 17 16 15 14 13 12 11
2003 2003.9 2004.8 2005.7 2006.6 2007.5 2008.4 2009.3 2010.2 2011.1 2012
* Indicates that the Annual Percent Change (APC) is significantly different from zero at the alpha = 0.05 level.
Final Selected Model: 0 Joinpoints.

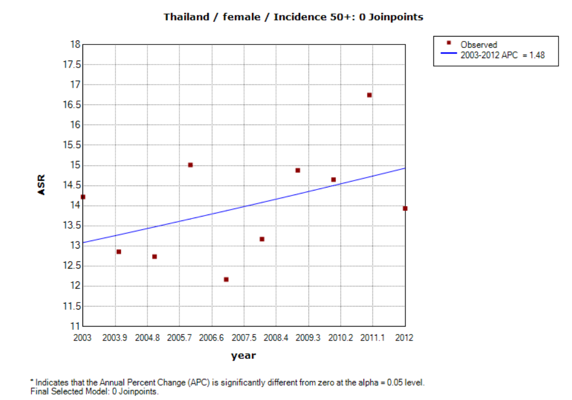
Thailand / female / Incidence 50+: 0 Joinpoints
Observed
2003-2012 APC = 1.48
ASR
year
18 17.5 17 16.5 16 15.5 15 14.5 14 13.5 13 12.5 12 11.5 11
2003 2003.9 2004.8 2005.7 2006.6 2007.5 2008.4 2009.3 2010.2 2011.1 2012
* Indicates that the Annual Percent Change (APC) is significantly different from zero at the alpha = 0.05 level.
Final Selected Model: 0 Joinpoints.

Oceania

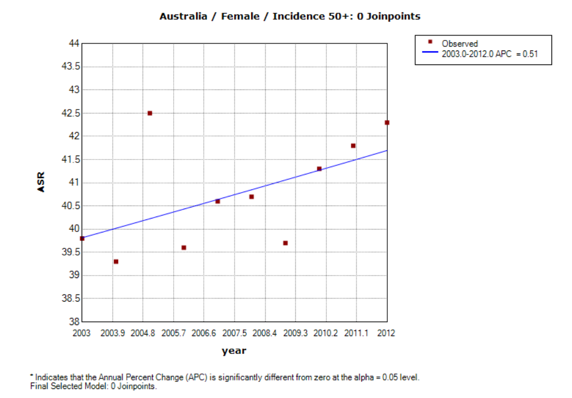
Australia / Female / Incidence 50+: 0 Joinpoints
Observed
2003.0-2012.0 APC = 0.51
ASR
year
44 43.5 43 42.5 42 41.5 41 40.5 40 39.5 39 38.5 38
2003 2003.9 2004.8 2005.7 2006.6 2007.5 2008.4 2009.3 2010.2 2011.1 2012
* Indicates that the Annual Percent Change (APC) is significantly different from zero at the alpha = 0.05 level.
Final Selected Model: 0 Joinpoints.

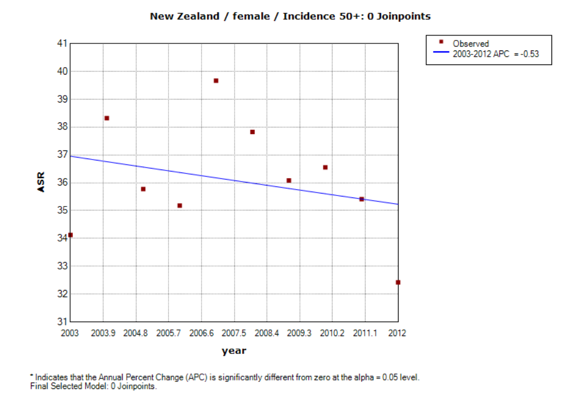
New Zealand / female / Incidence 50+: 0 Joinpoints
Observed
2003-2012 APC = -0.53
ASR
year
41 40 39 38 37 36 35 34 33 32 31
2003 2003.9 2004.8 2005.7 2006.6 2007.5 2008.4 2009.3 2010.2 2011.1 2012
* Indicates that the Annual Percent Change (APC) is significantly different from zero at the alpha = 0.05 level.
Final Selected Model: 0 Joinpoints.

Northern America

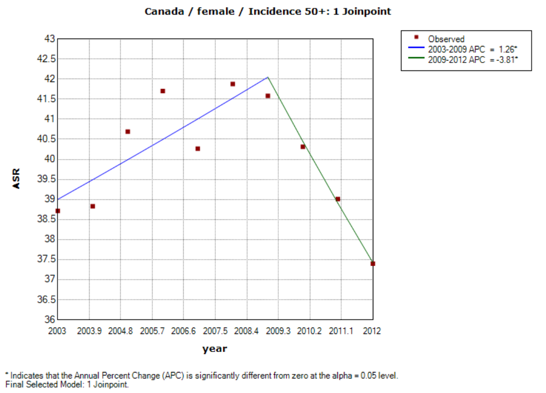

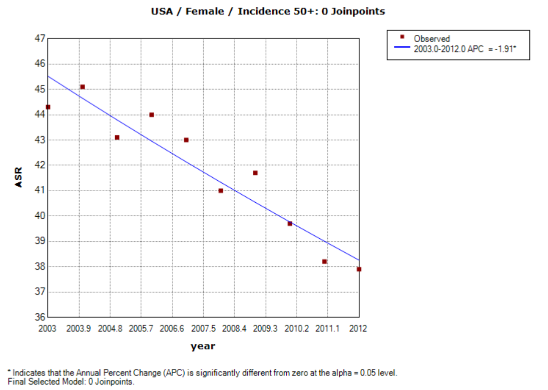

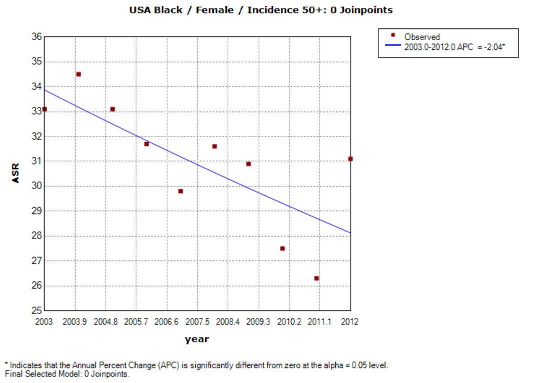

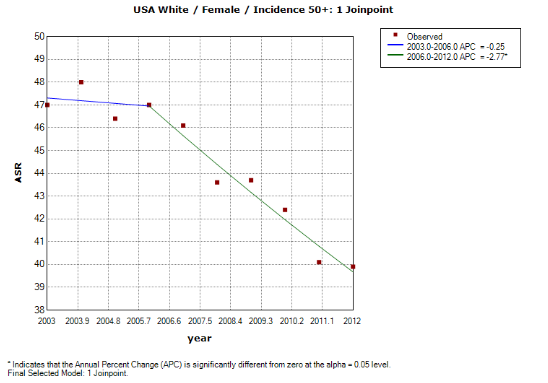

Southern America

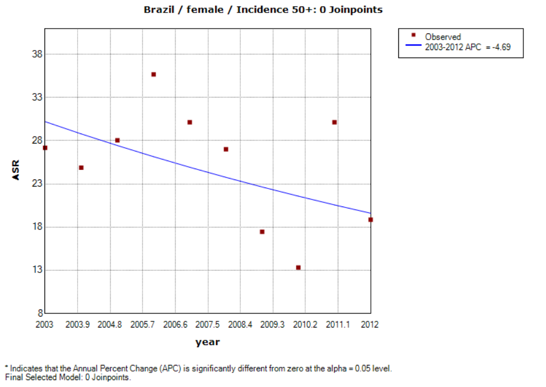

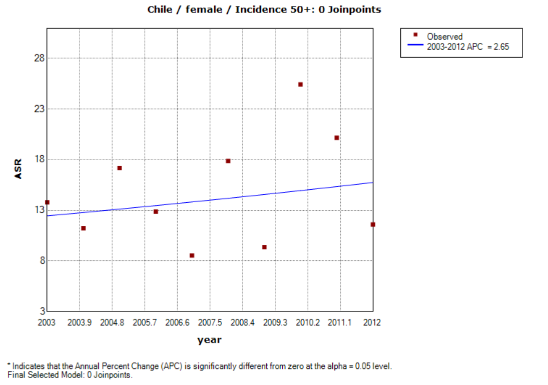

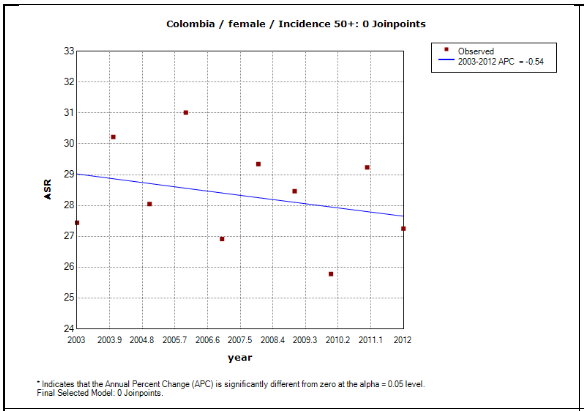

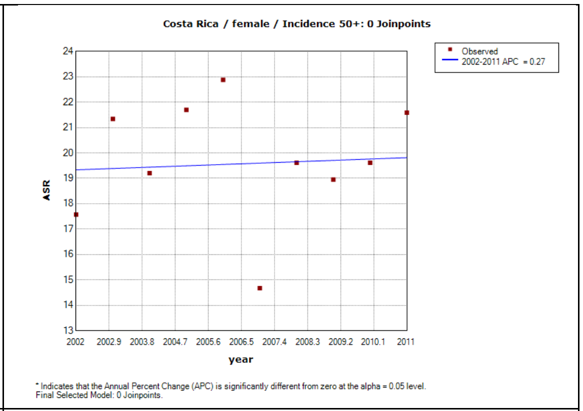

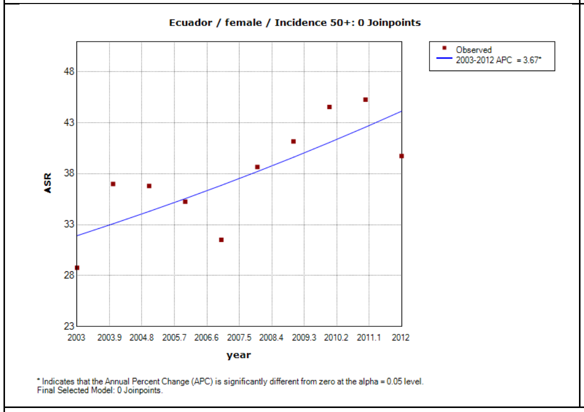

Northern Europe

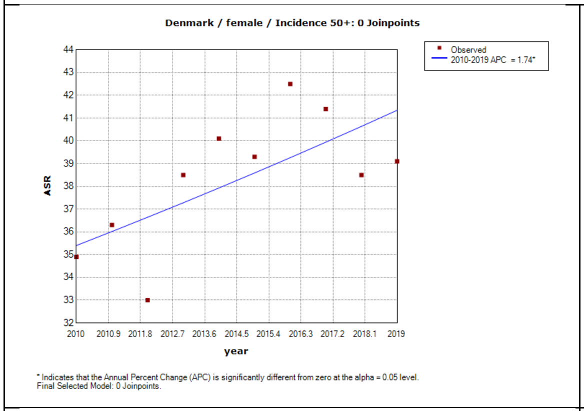

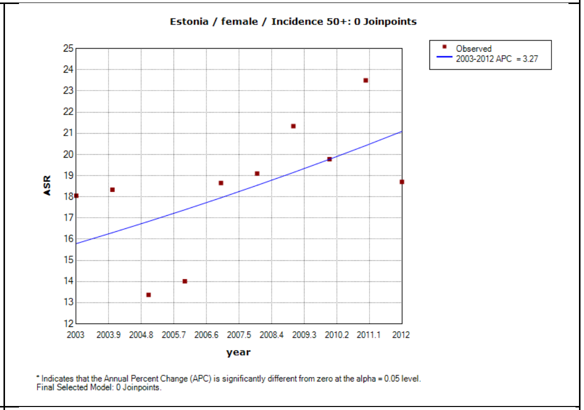

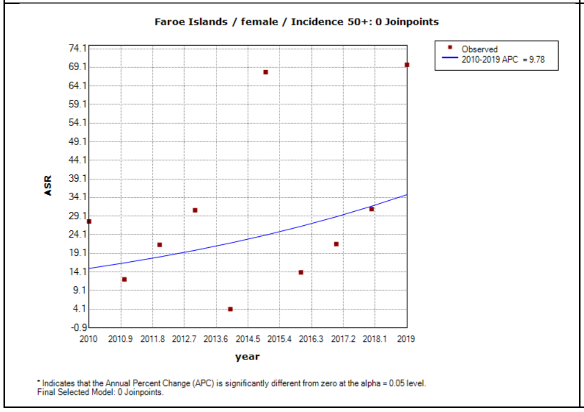

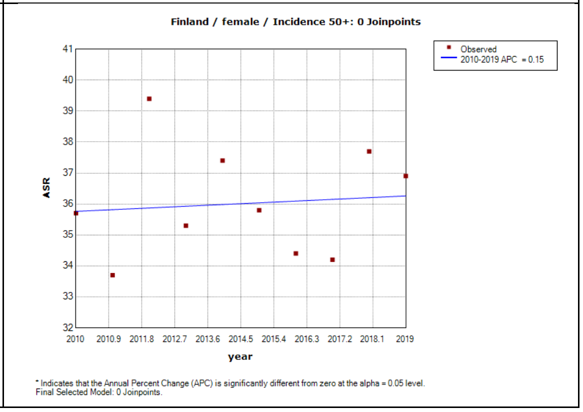

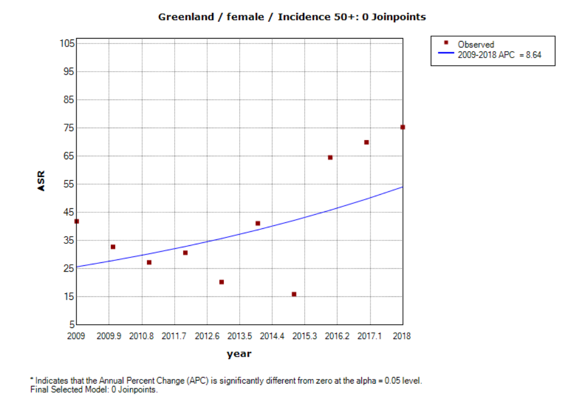

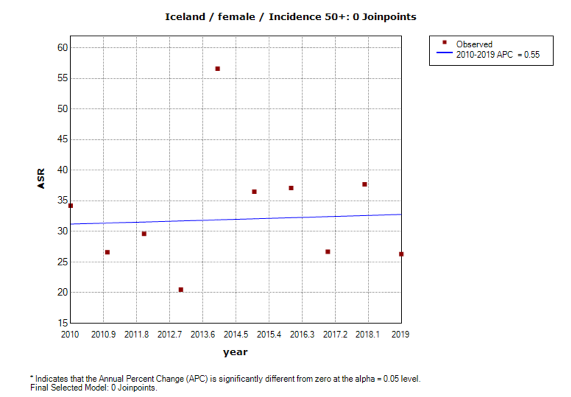

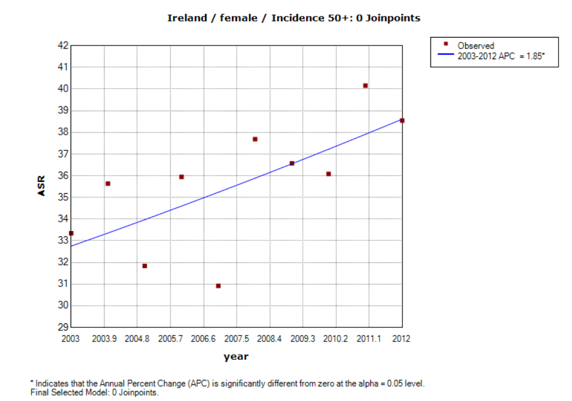

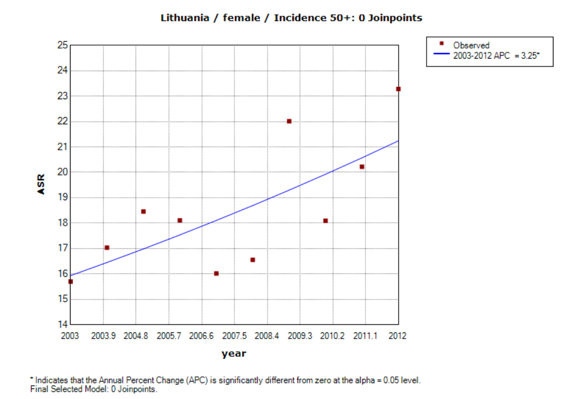

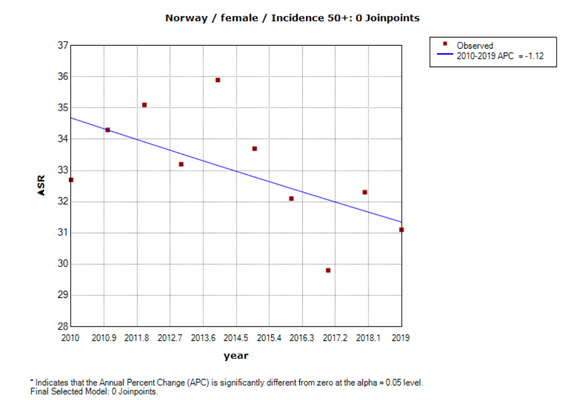

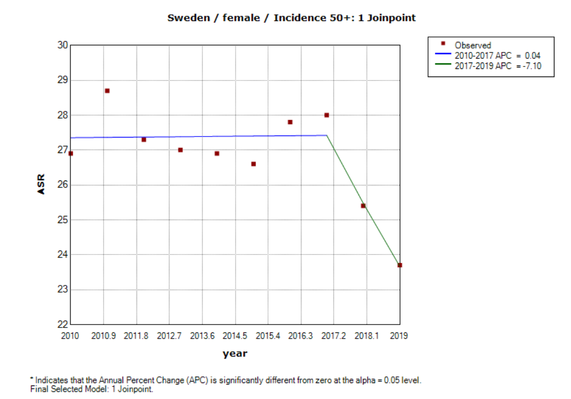

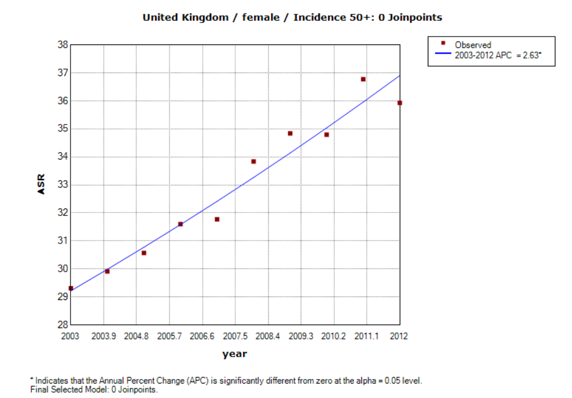

Western Europe

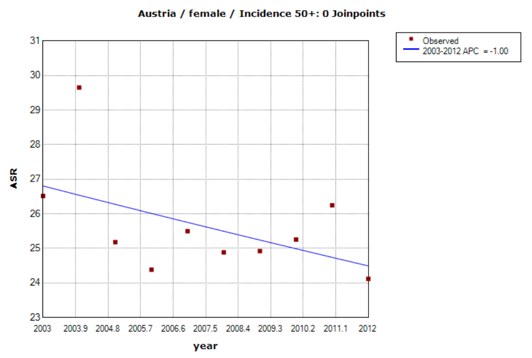

* Indicates that the Annual Percent Change (APC) is significantly different from zero at the alpha = 0.05 level.
Final Selected Model: 0 Joinpoints.

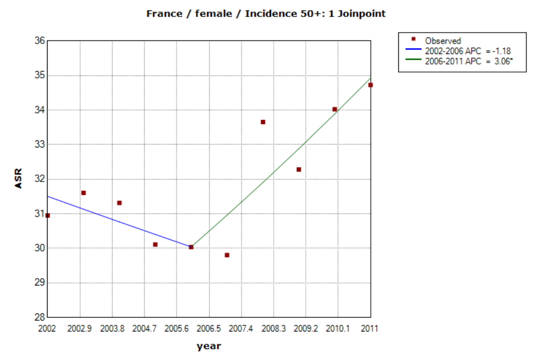

* Indicates that the Annual Percent Change (APC) is significantly different from zero at the alpha = 0.05 level.
Final Selected Model: 1 Joinpoint.

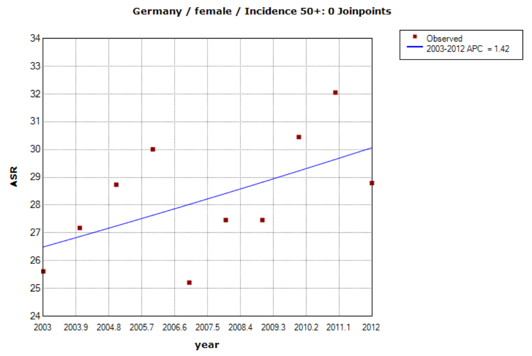

* Indicates that the Annual Percent Change (APC) is significantly different from zero at the alpha = 0.05 level.
Final Selected Model: 0 Joinpoints.

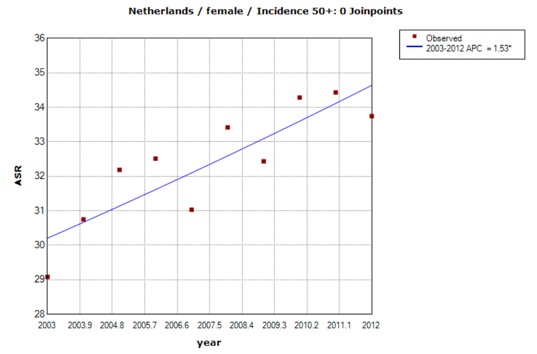

* Indicates that the Annual Percent Change (APC) is significantly different from zero at the alpha = 0.05 level.
Final Selected Model: 0 Joinpoints.

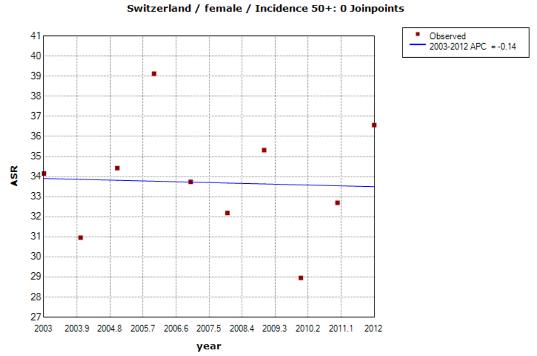

* Indicates that the Annual Percent Change (APC) is significantly different from zero at the alpha = 0.05 level.
Final Selected Model: 0 Joinpoints.

Southern Europe

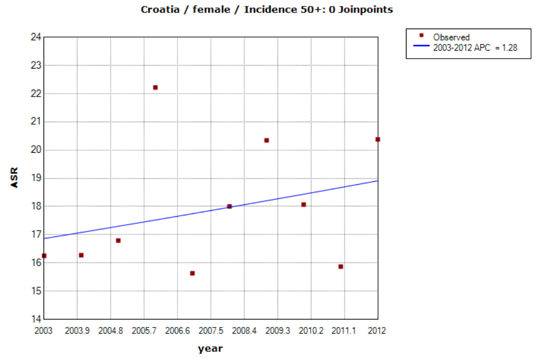

* Indicates that the Annual Percent Change (APC) is significantly different from zero at the alpha = 0.05 level.
Final Selected Model: 0 Joinpoints.

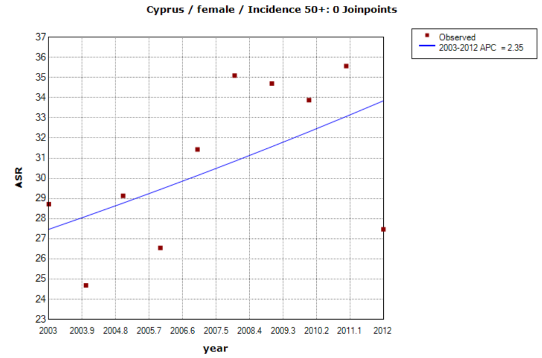

* Indicates that the Annual Percent Change (APC) is significantly different from zero at the alpha = 0.05 level.
Final Selected Model: 0 Joinpoints.

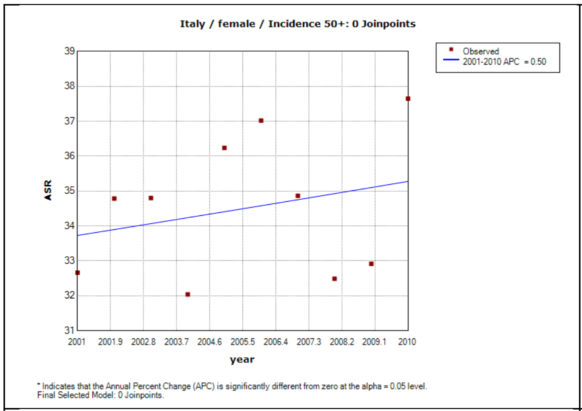

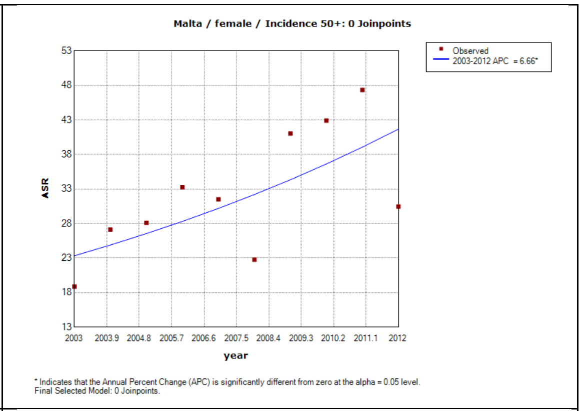

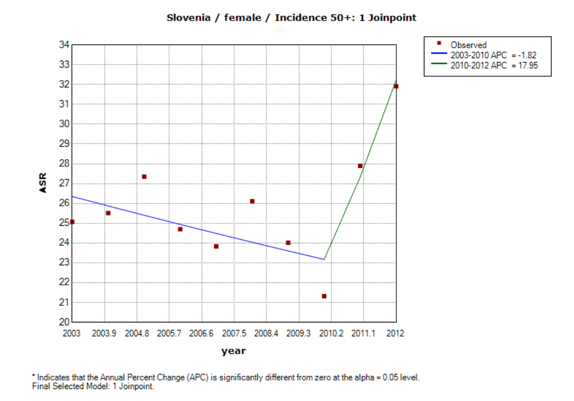

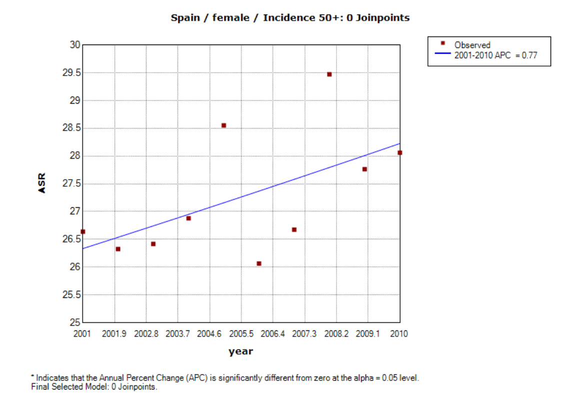

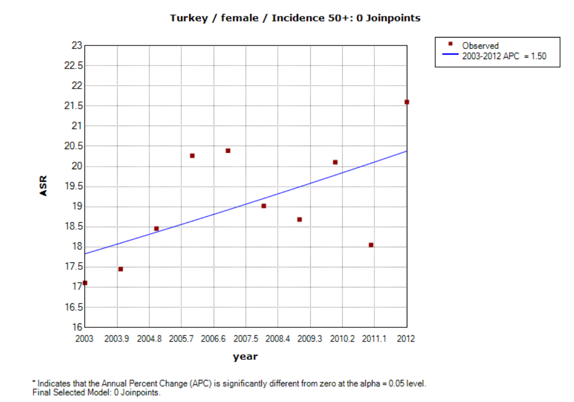

Eastern Europe

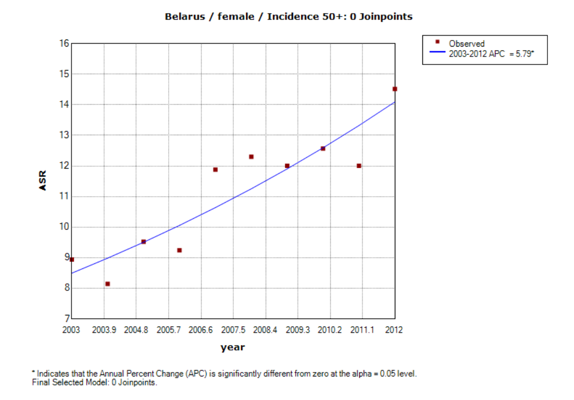

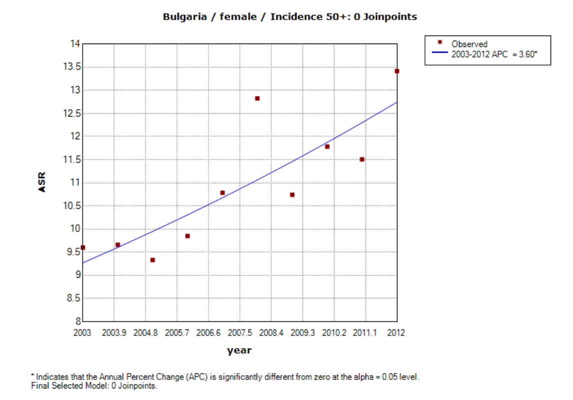

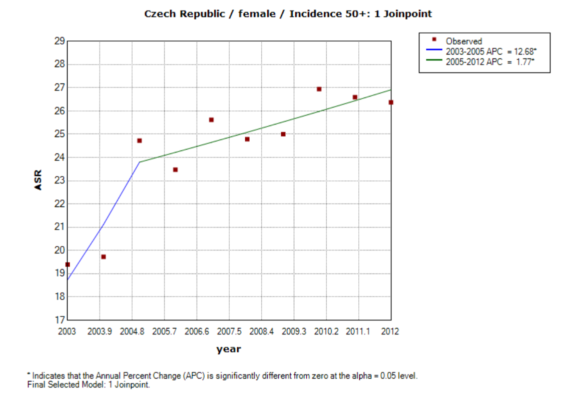
Czech Republic / female / Incidence 50+: 1 Joinpoint
Observed
2003-2005 APC = 12.68*
2005-2012 APC = 1.77*
ASR
year
* Indicates that the Annual Percent Change (APC) is significantly different from zero at the alpha = 0.05 level.
Final Selected Model: 1 Joinpoint.

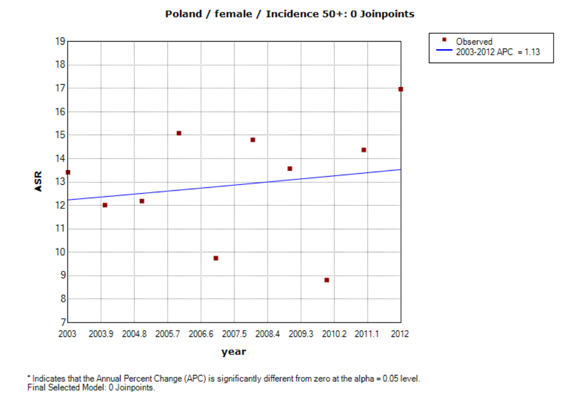
Poland / female / Incidence 50+: 0 Joinpoints
Observed
2003-2012 APC = 1.13
ASR
year
* Indicates that the Annual Percent Change (APC) is significantly different from zero at the alpha = 0.05 level.
Final Selected Model: 0 Joinpoints.

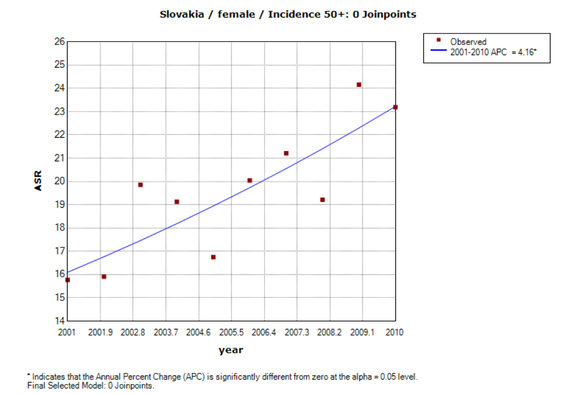
Slovakia / female / Incidence 50+: 0 Joinpoints
Observed
2001-2010 APC = 4.16*
ASR
year
* Indicates that the Annual Percent Change (APC) is significantly different from zero at the alpha = 0.05 level.
Final Selected Model: 0 Joinpoints.

Africa

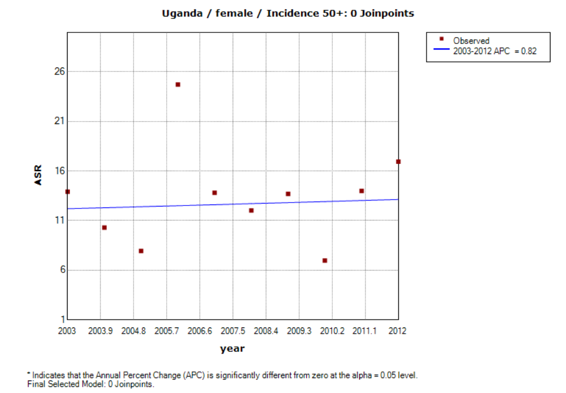
Uganda / female / Incidence 50+: 0 Joinpoints
Observed
2003-2012 APC = 0.82
ASR
year
* Indicates that the Annual Percent Change (APC) is significantly different from zero at the alpha = 0.05 level.
Final Selected Model: 0 Joinpoints.

i.) Mortality male all ages

Asia

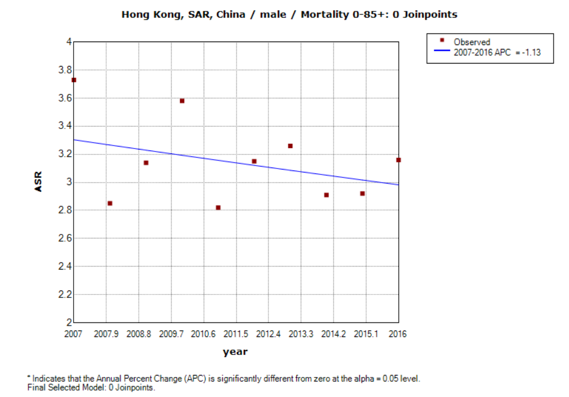

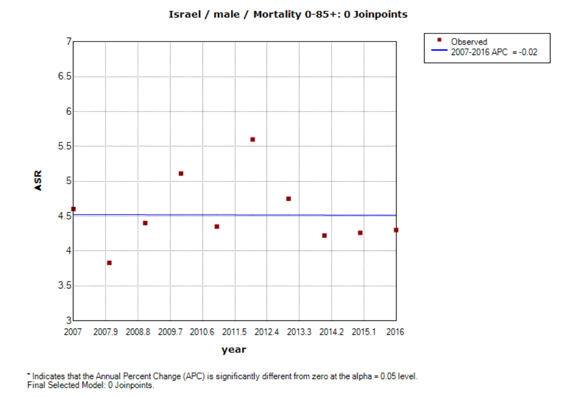

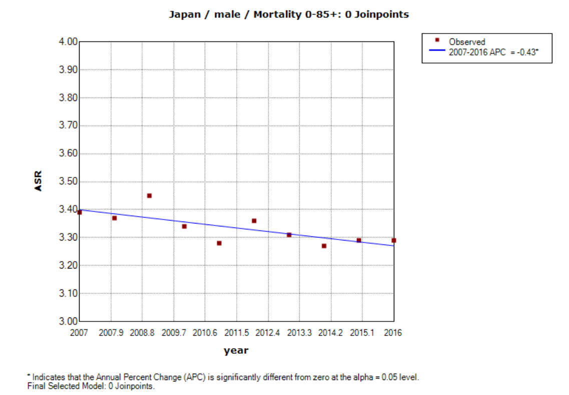

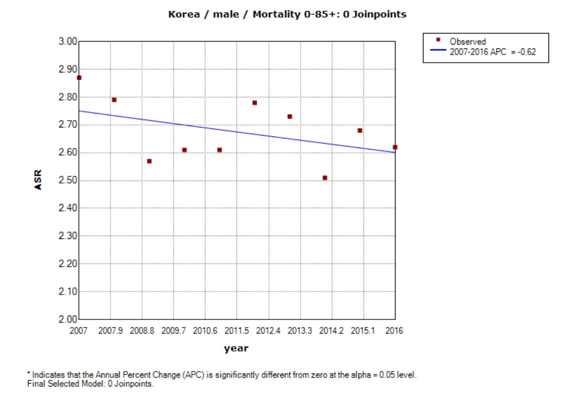

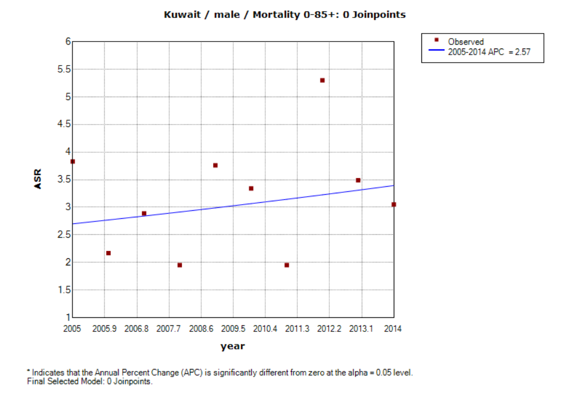

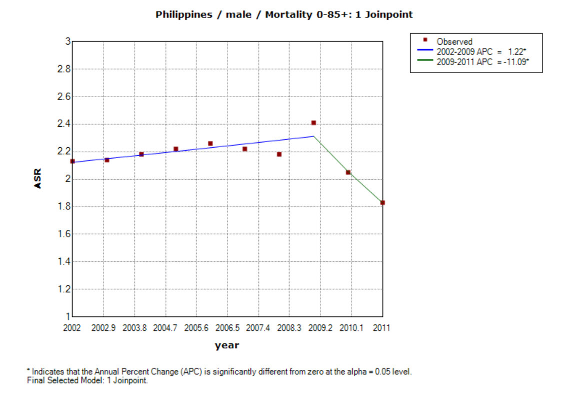

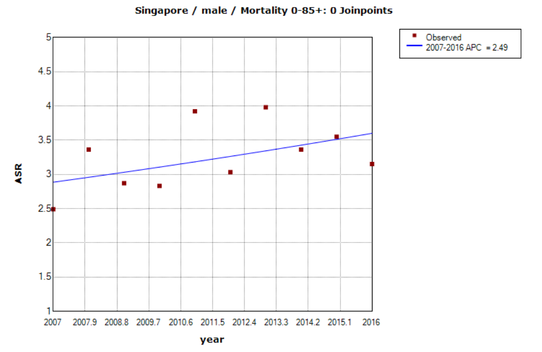

* Indicates that the Annual Percent Change (APC) is significantly different from zero at the alpha = 0.05 level.
Final Selected Model: 0 Joinpoints.

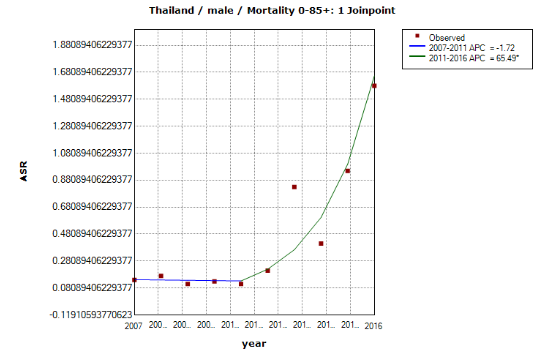

* Indicates that the Annual Percent Change (APC) is significantly different from zero at the alpha = 0.05 level.
Final Selected Model: 1 Joinpoint.

Oceania

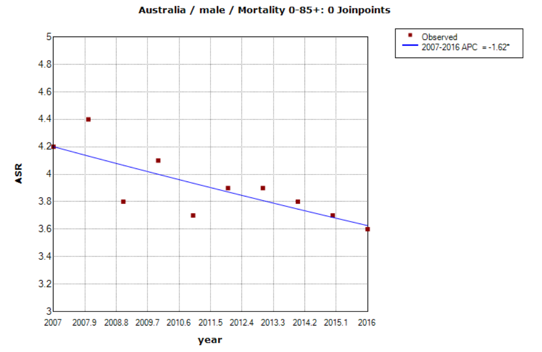

* Indicates that the Annual Percent Change (APC) is significantly different from zero at the alpha = 0.05 level.
Final Selected Model: 0 Joinpoints.

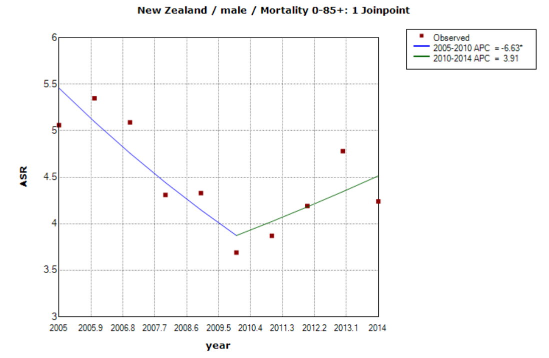

* Indicates that the Annual Percent Change (APC) is significantly different from zero at the alpha = 0.05 level.
Final Selected Model: 1 Joinpoint.

Northern America

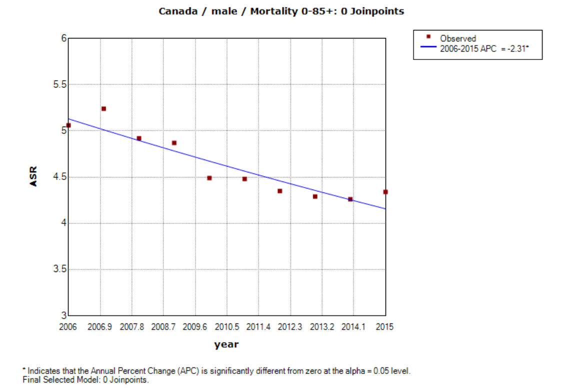

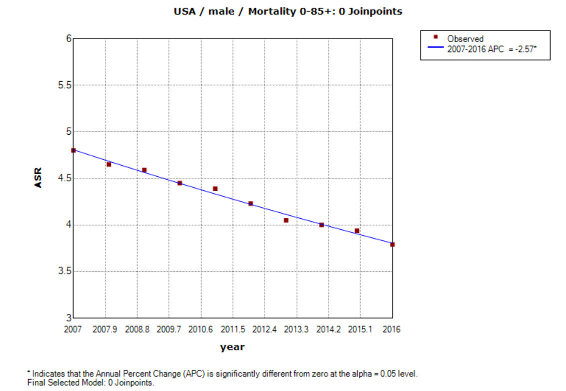

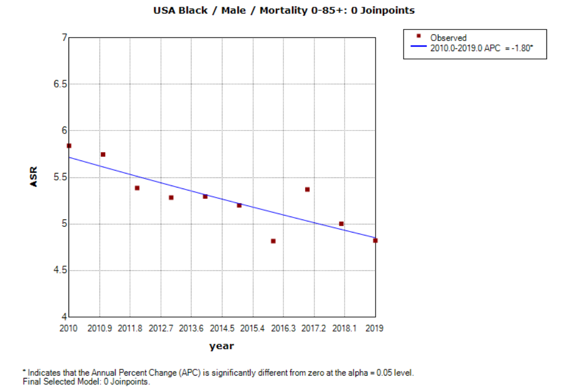

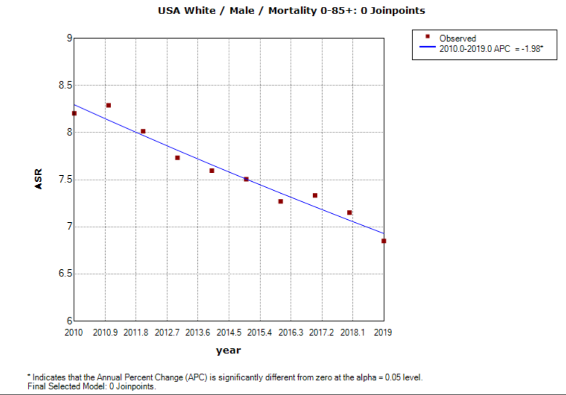

Southern America

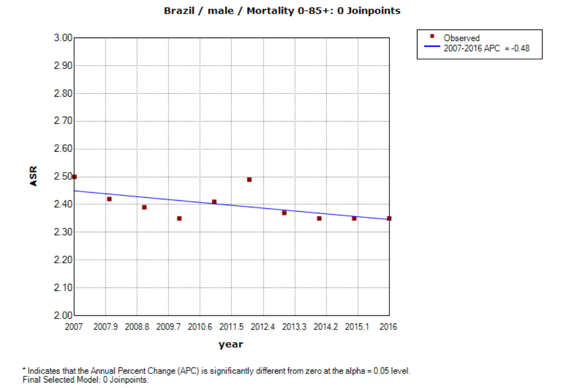

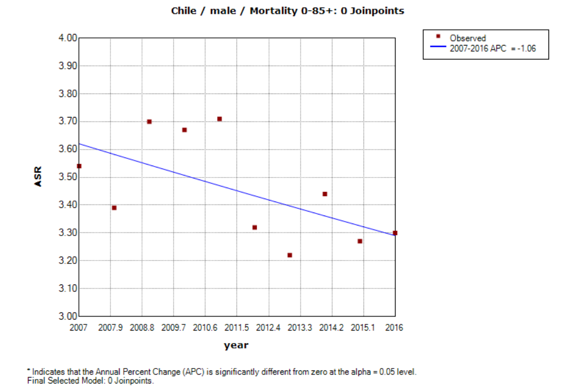

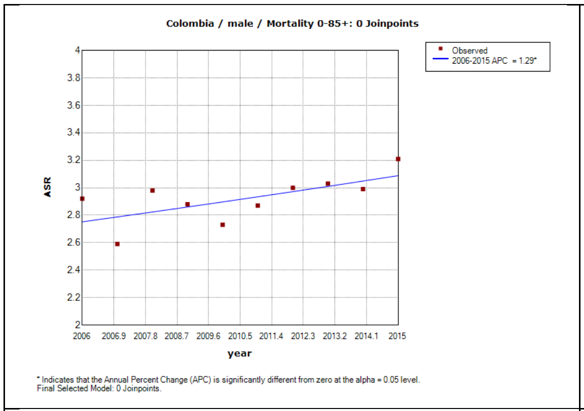

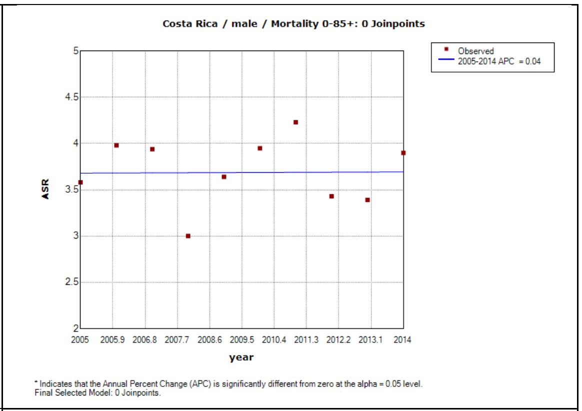

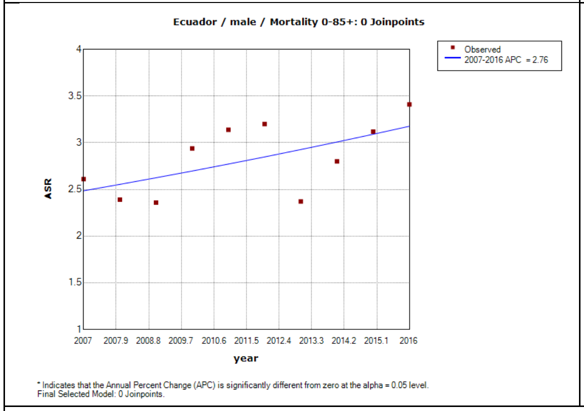

Northern Europe

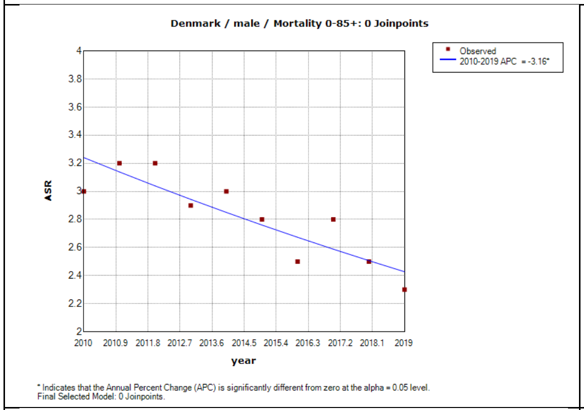

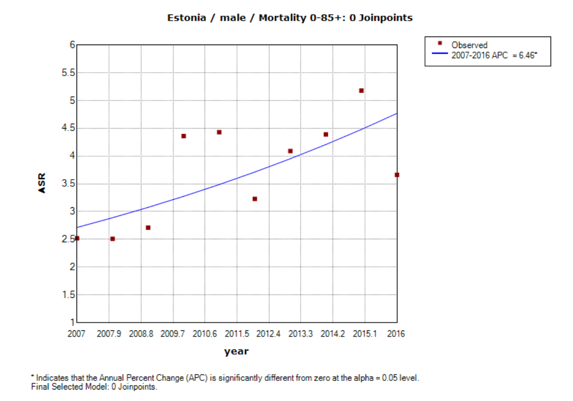

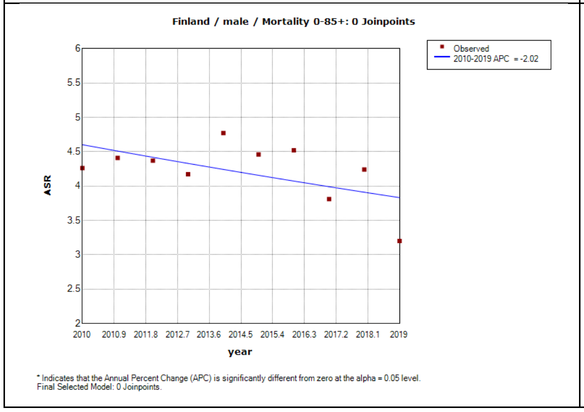

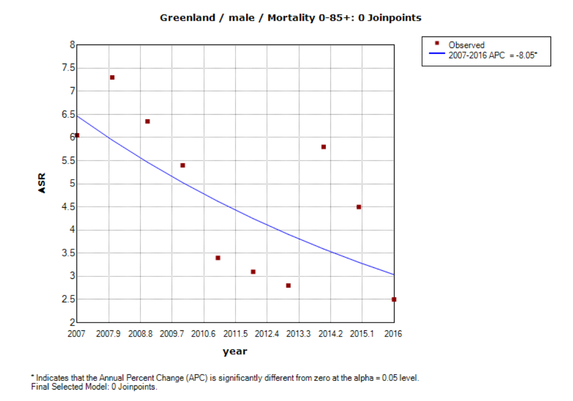

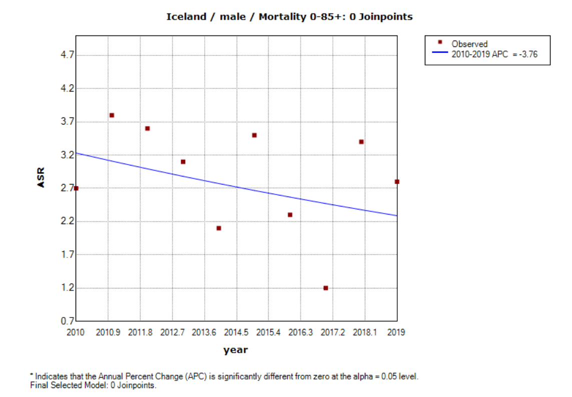

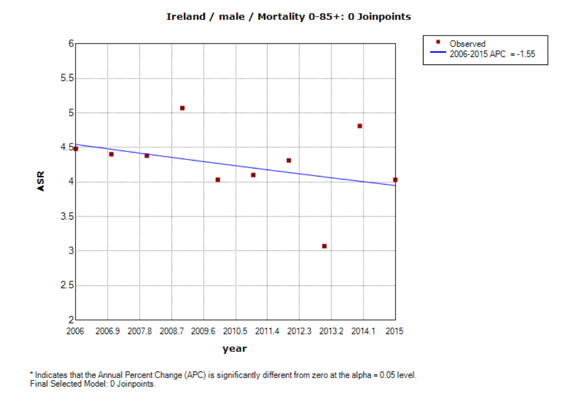

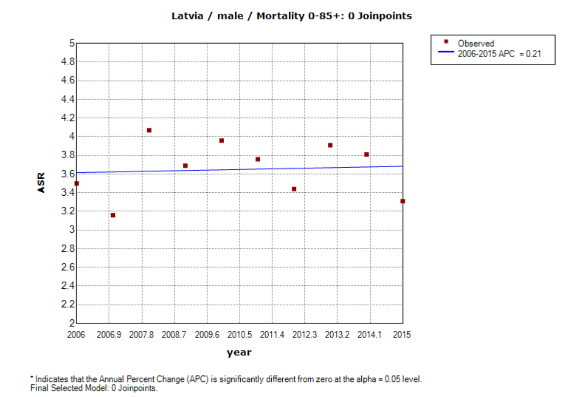

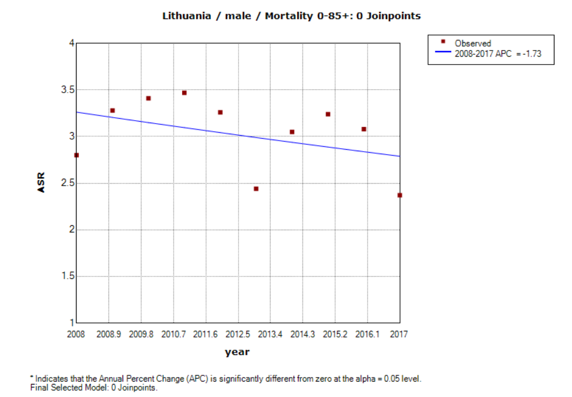

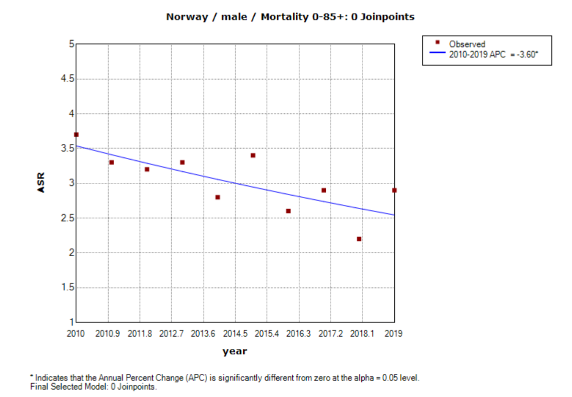

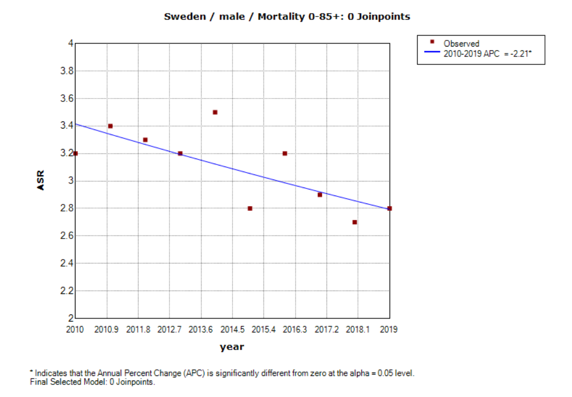

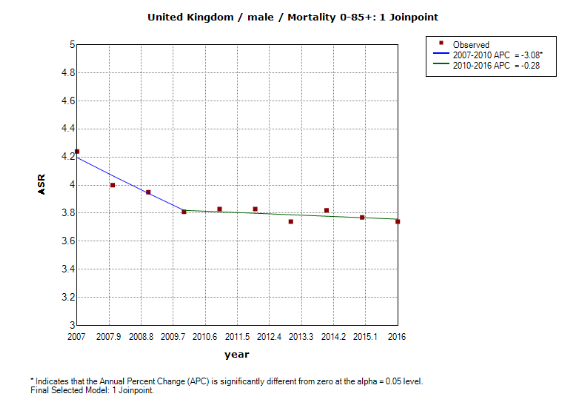

Western Europe

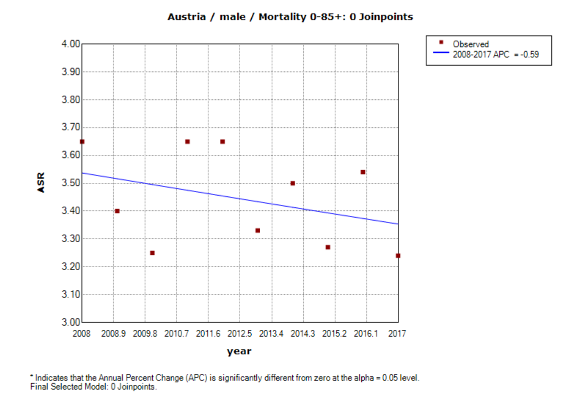

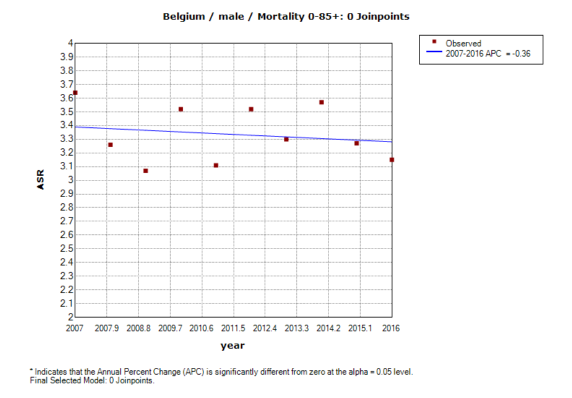

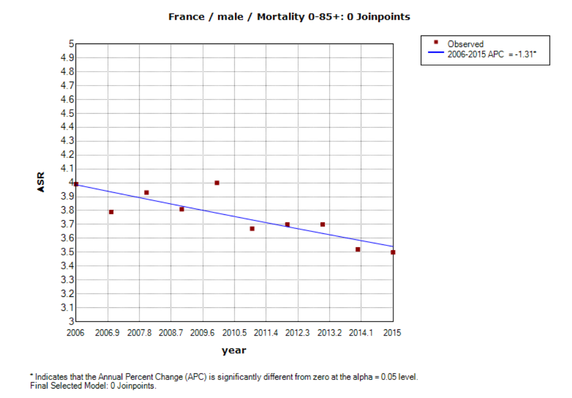

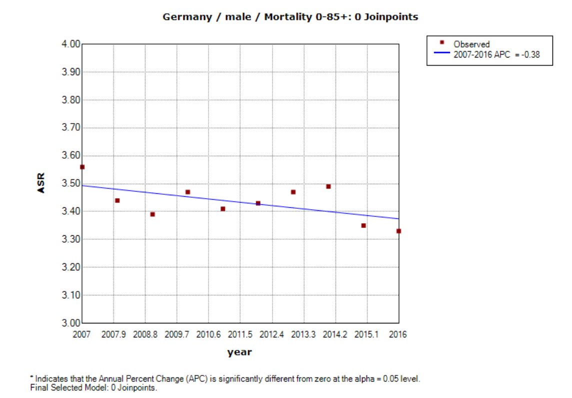

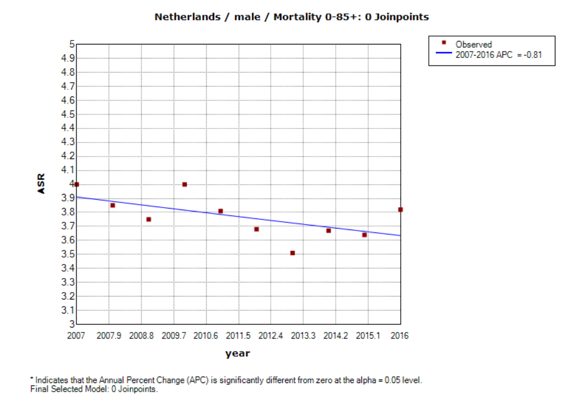

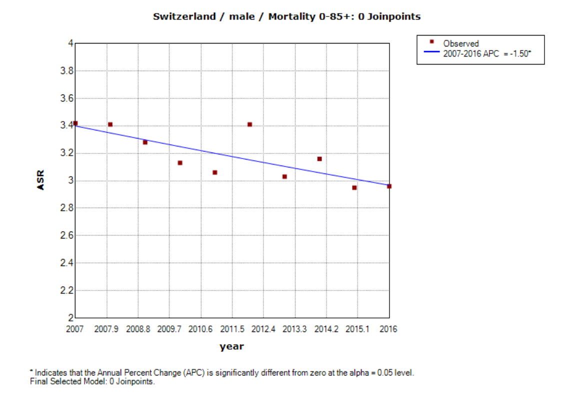

Southern Europe

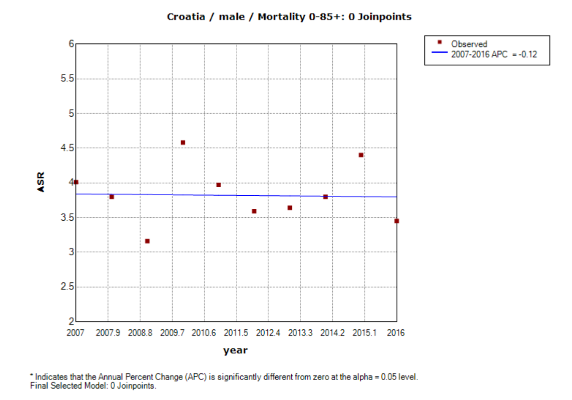

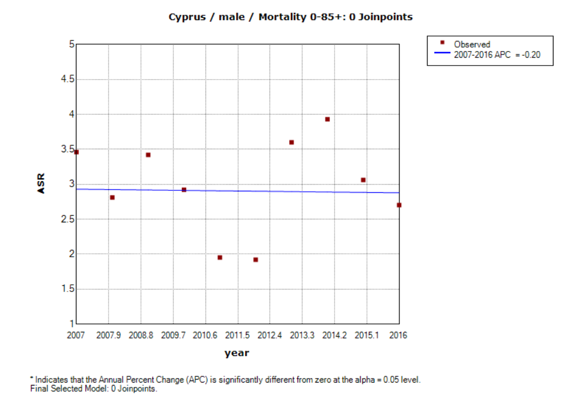

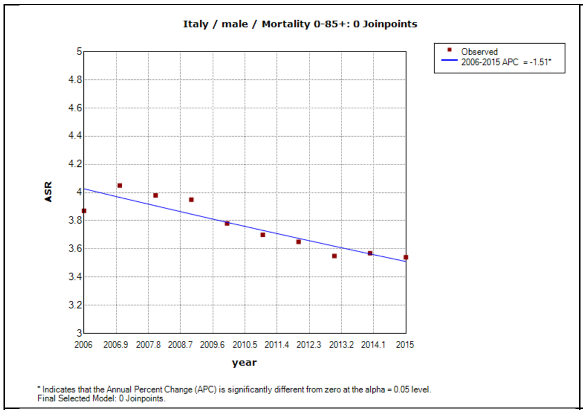

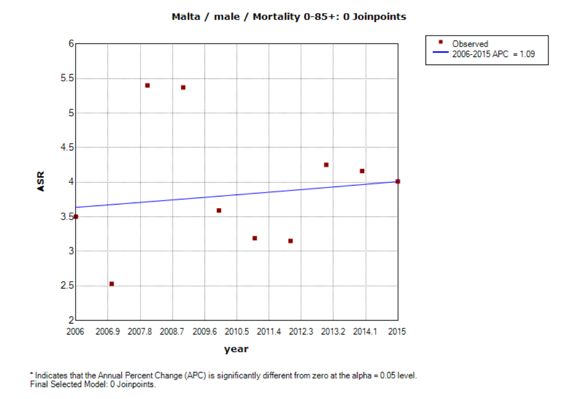

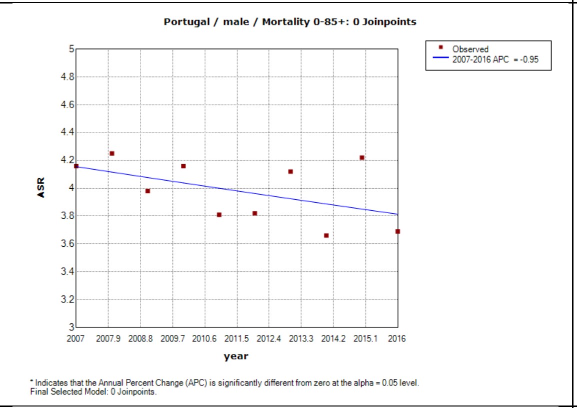

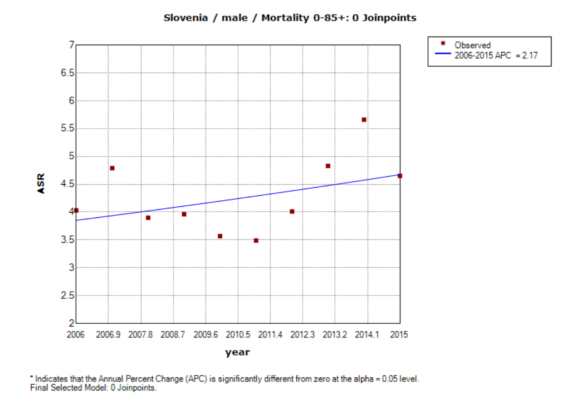

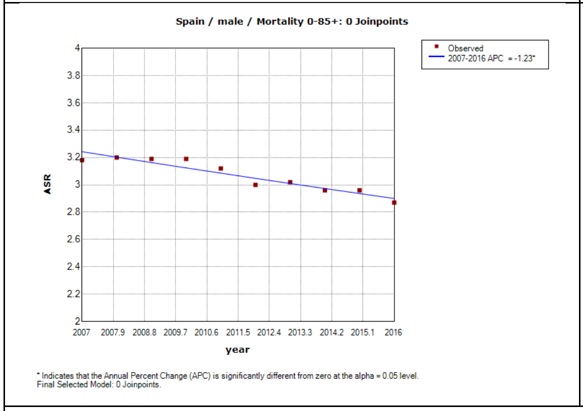

Eastern Europe

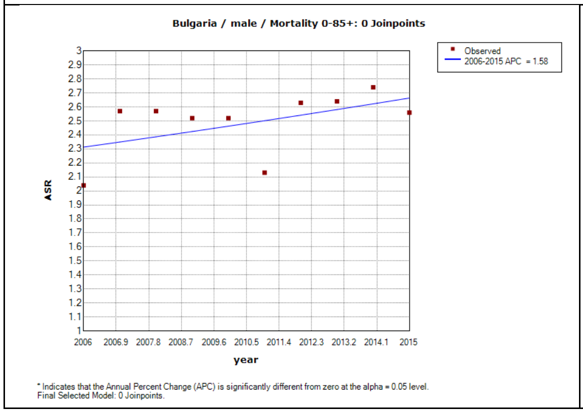

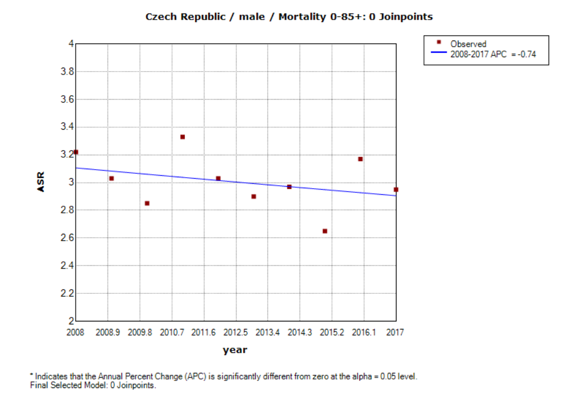

Poland / male / Mortality 0-85+: 1 Joinpoint

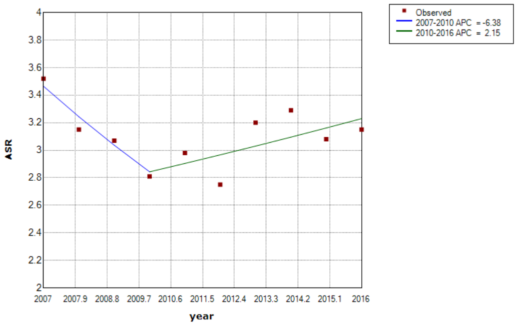

* Indicates that the Annual Percent Change (APC) is significantly different from zero at the alpha = 0.05 level.
Final Selected Model: 1 Joinpoint.

Russian Federation / male / Mortality 0-85+: 0 Joinpoints

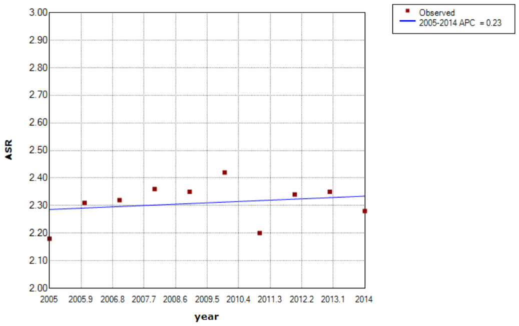

* Indicates that the Annual Percent Change (APC) is significantly different from zero at the alpha = 0.05 level.
Final Selected Model: 0 Joinpoints.

Slovakia / male / Mortality 0-85+: 1 Joinpoint

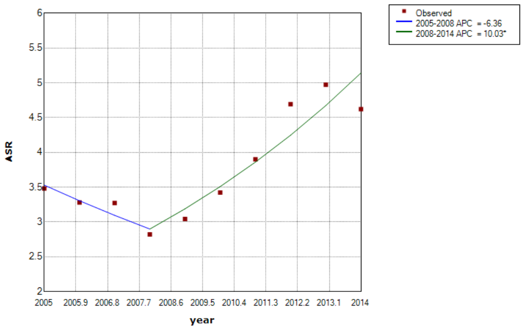

* Indicates that the Annual Percent Change (APC) is significantly different from zero at the alpha = 0.05 level.
Final Selected Model: 1 Joinpoint.

j.) Mortality female all ages

Asia

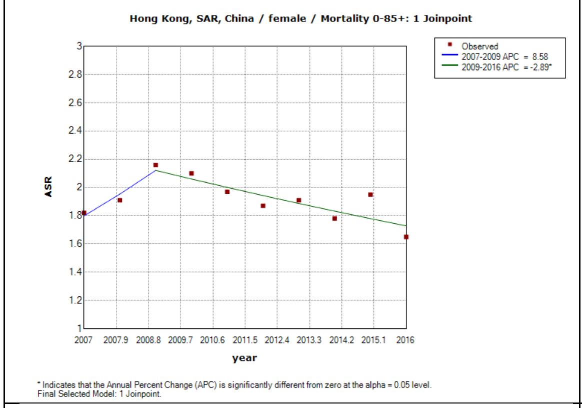

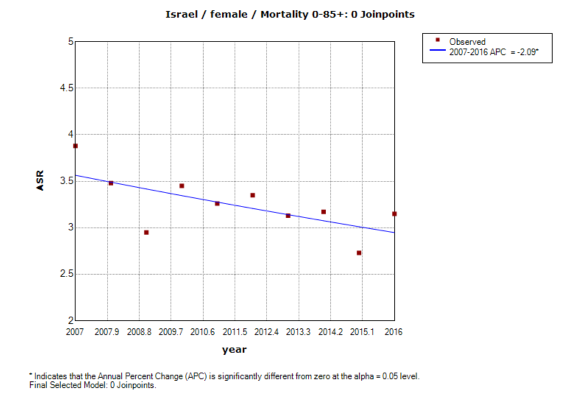

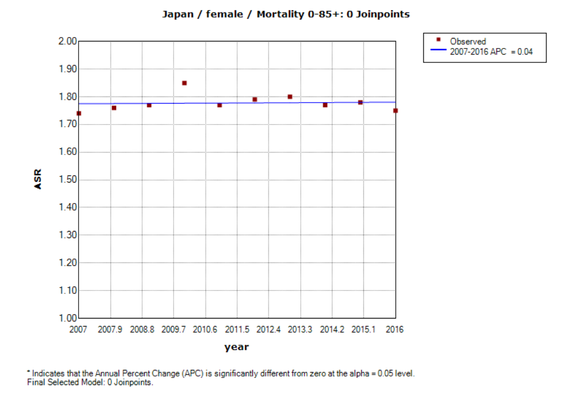

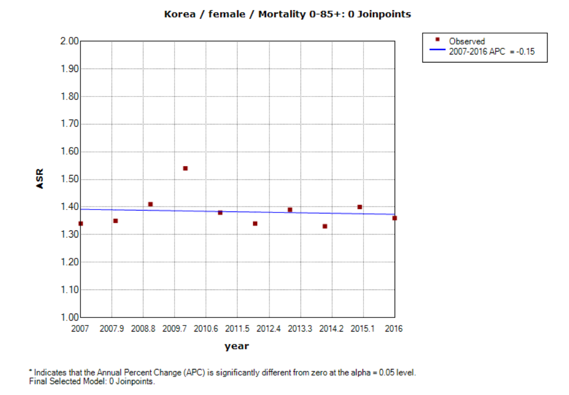

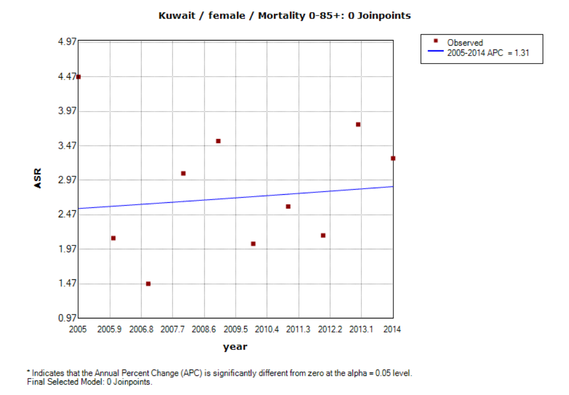

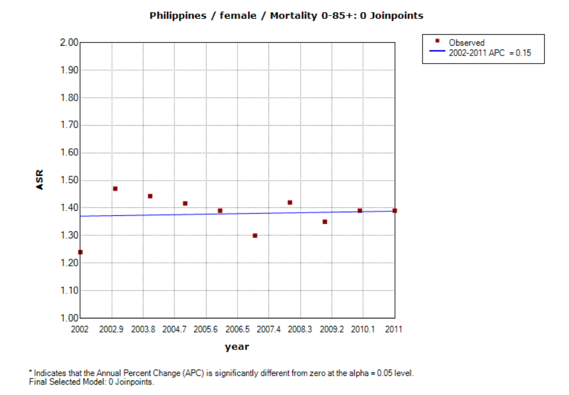

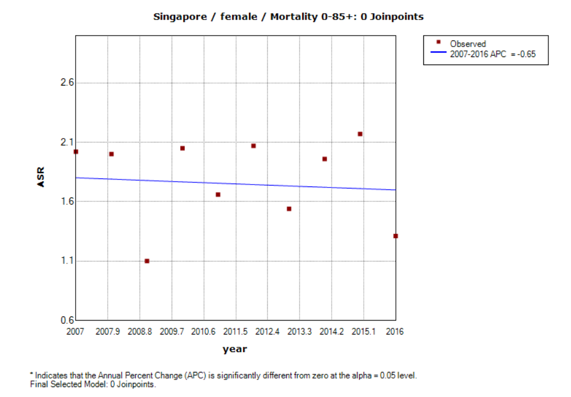
Singapore / female / Mortality 0-85+: 0 Joinpoints
Observed
2007-2016 APC = -0.65
ASR
year
* Indicates that the Annual Percent Change (APC) is significantly different from zero at the alpha = 0.05 level.
Final Selected Model: 0 Joinpoints.

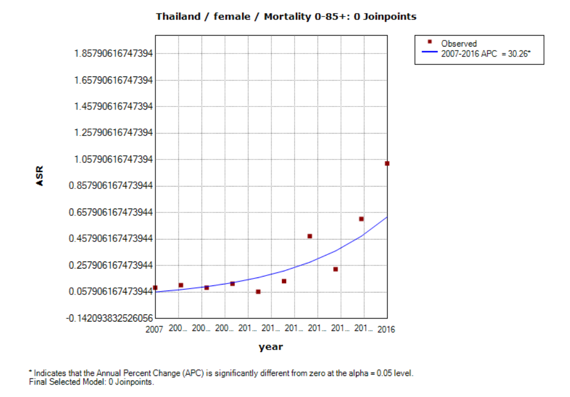
Thailand / female / Mortality 0-85+: 0 Joinpoints
Observed
2007-2016 APC = 30.26*
ASR
year
* Indicates that the Annual Percent Change (APC) is significantly different from zero at the alpha = 0.05 level.
Final Selected Model: 0 Joinpoints.

Oceania

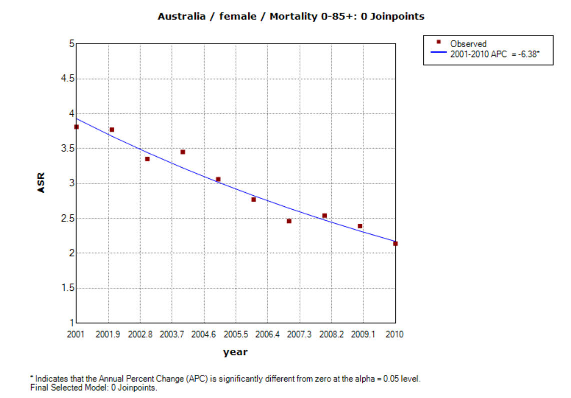
Australia / female / Mortality 0-85+: 0 Joinpoints
Observed
2001-2010 APC = -6.38*
ASR
year
* Indicates that the Annual Percent Change (APC) is significantly different from zero at the alpha = 0.05 level.
Final Selected Model: 0 Joinpoints.

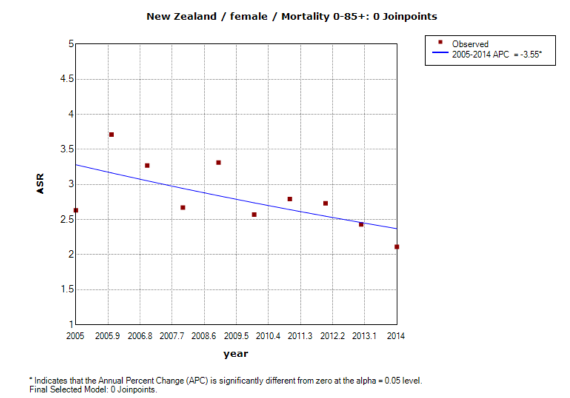
New Zealand / female / Mortality 0-85+: 0 Joinpoints
Observed
2005-2014 APC = -3.55*
ASR
year
* Indicates that the Annual Percent Change (APC) is significantly different from zero at the alpha = 0.05 level.
Final Selected Model: 0 Joinpoints.

Northern America

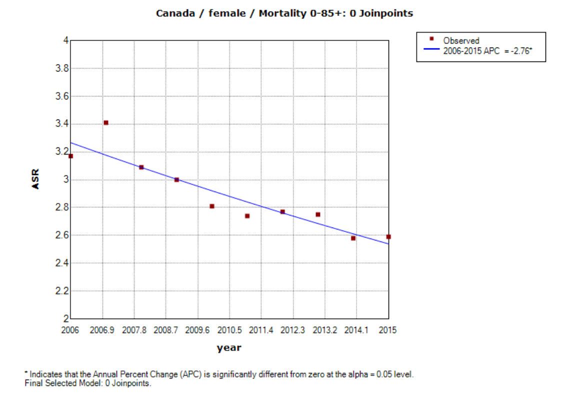

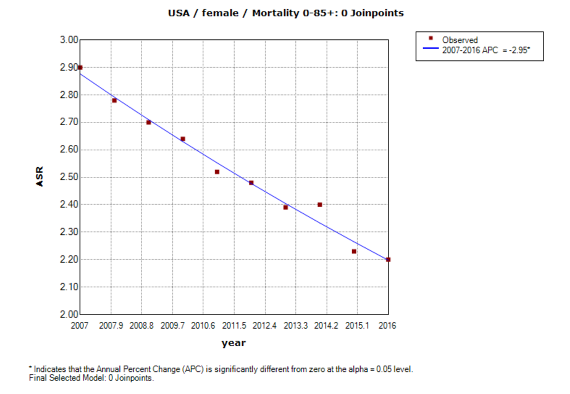

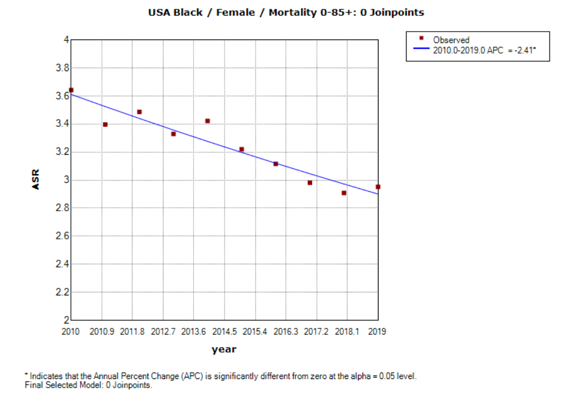

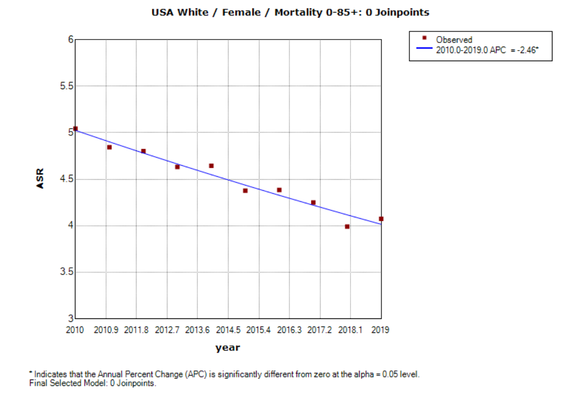

Southern America

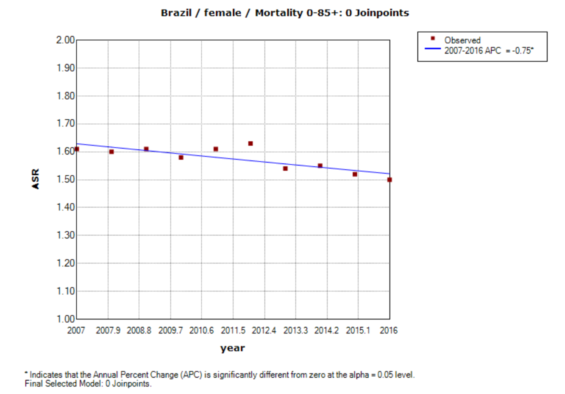

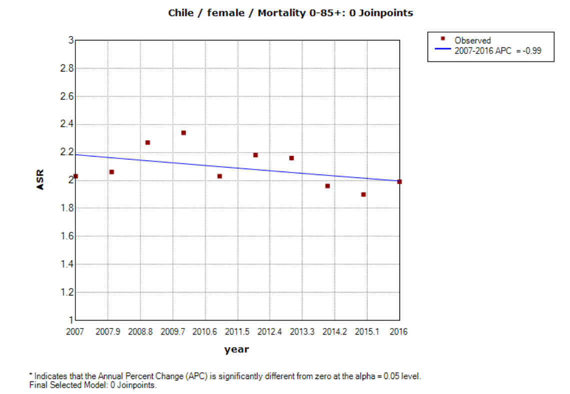

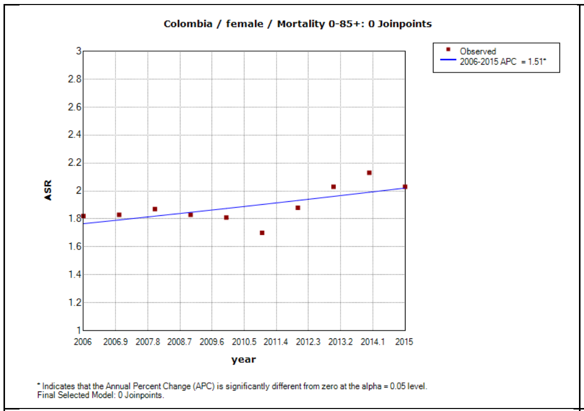

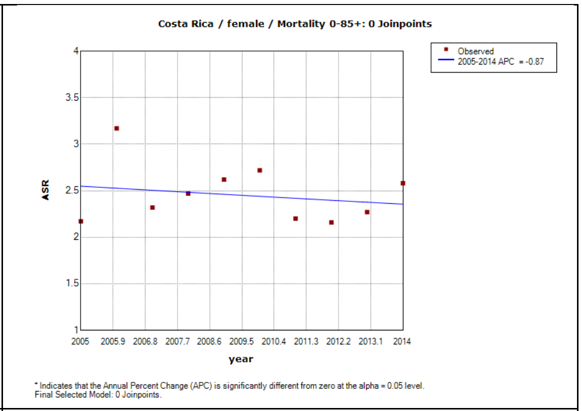

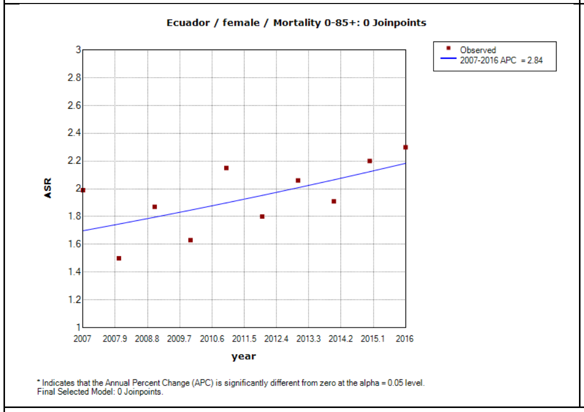

Northern Europe

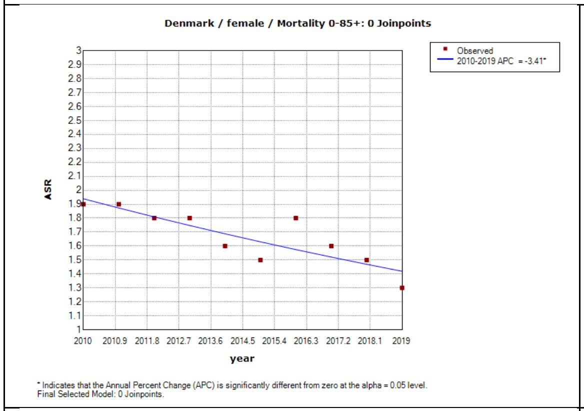

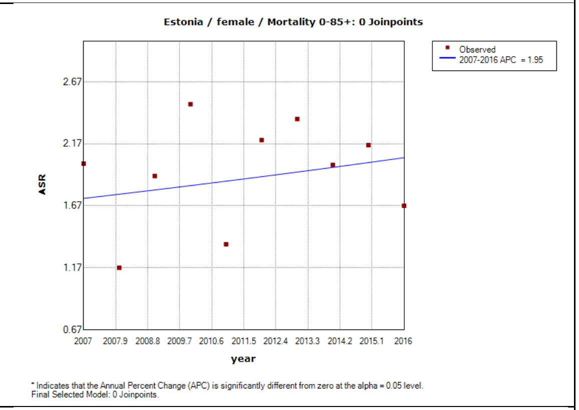

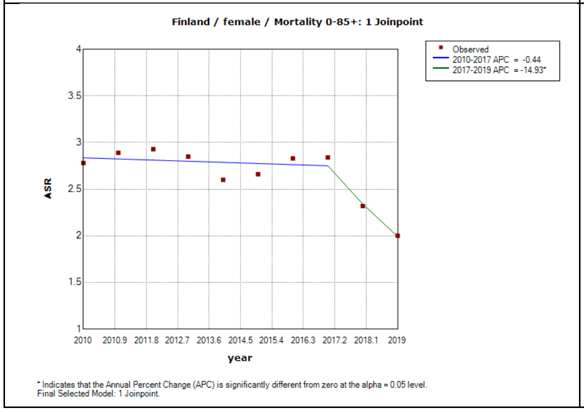

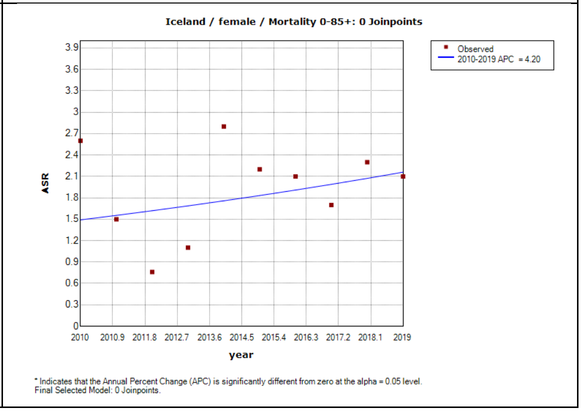

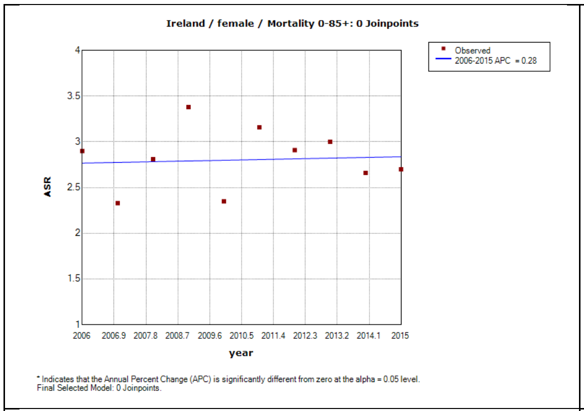

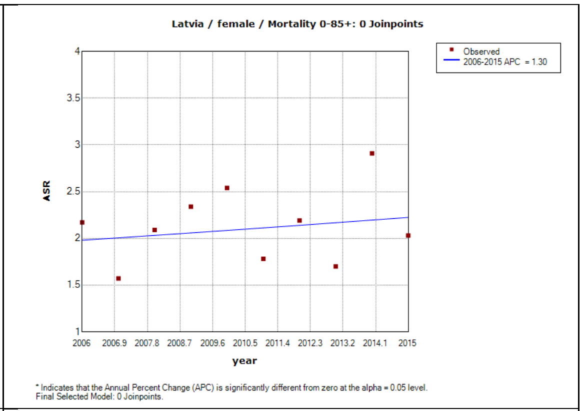

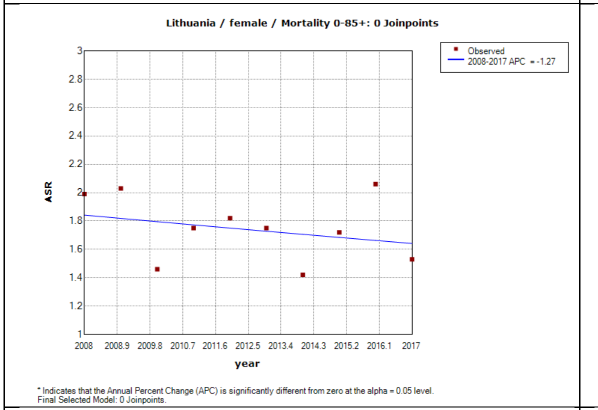

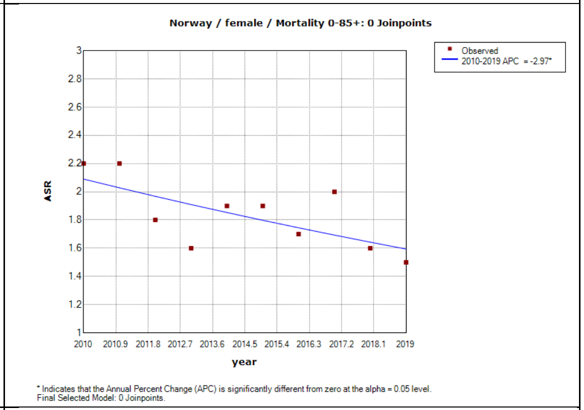

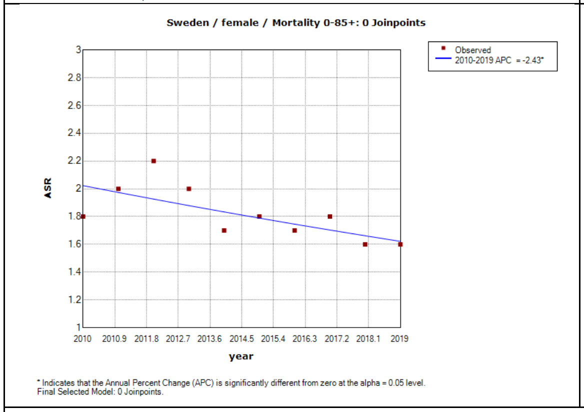

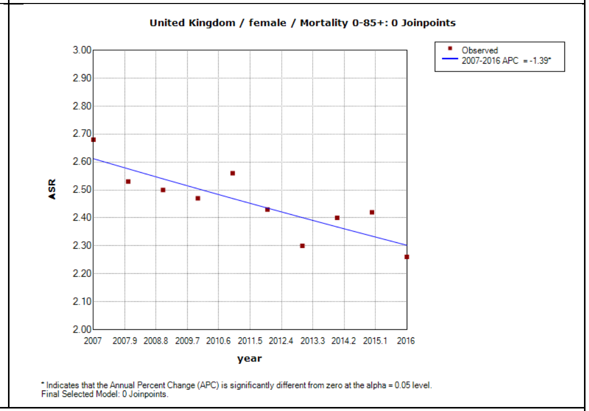

Western Europe

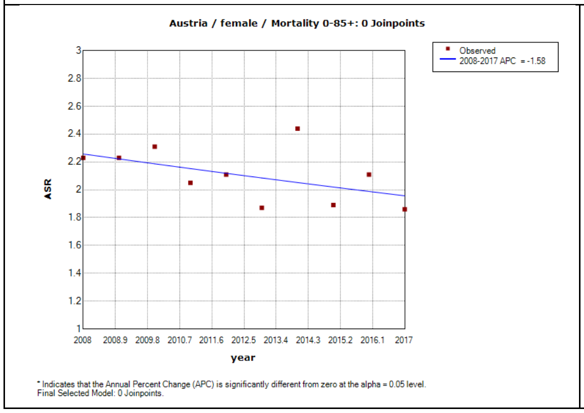

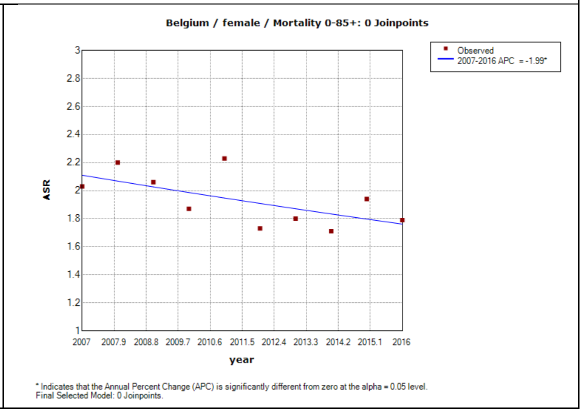

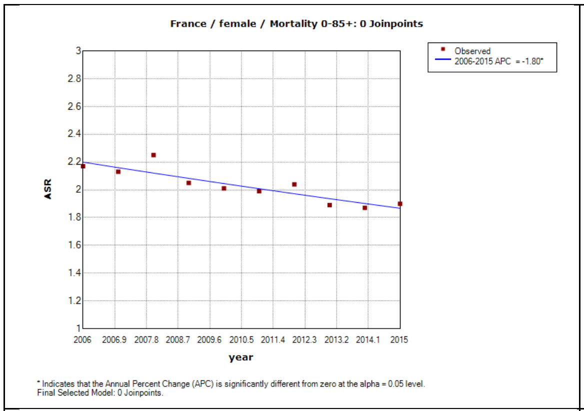

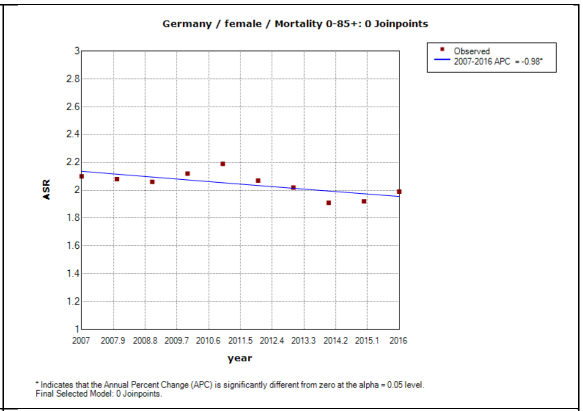

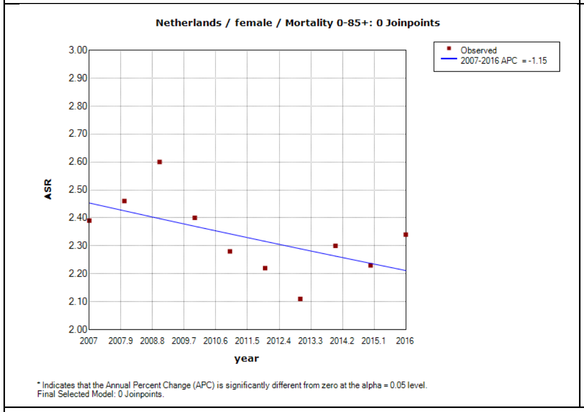

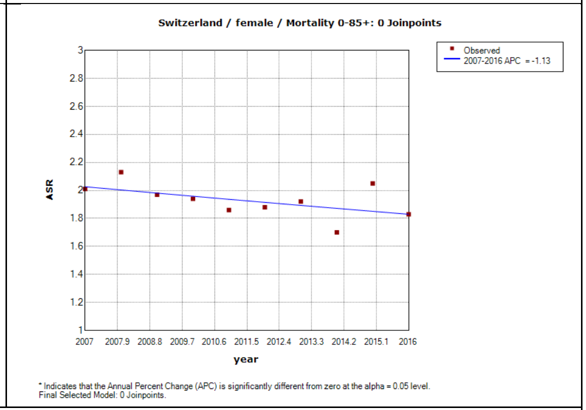

Southern Europe

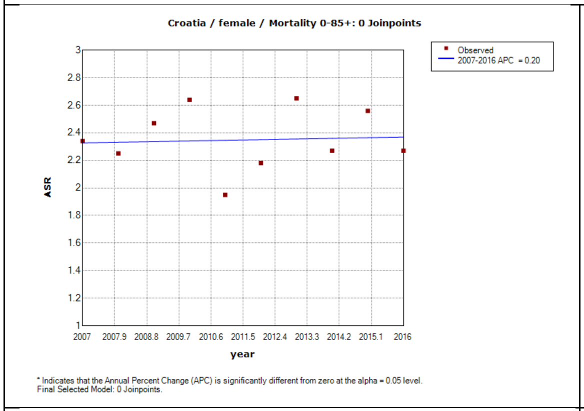

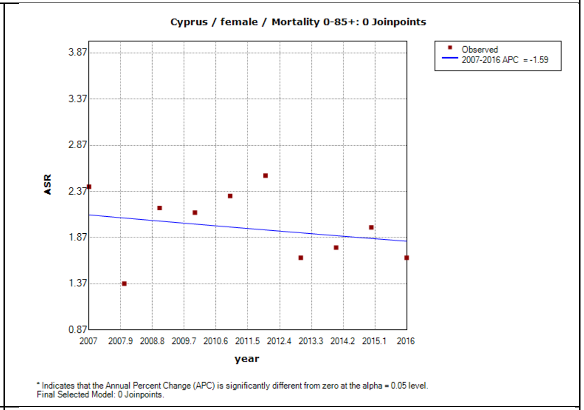

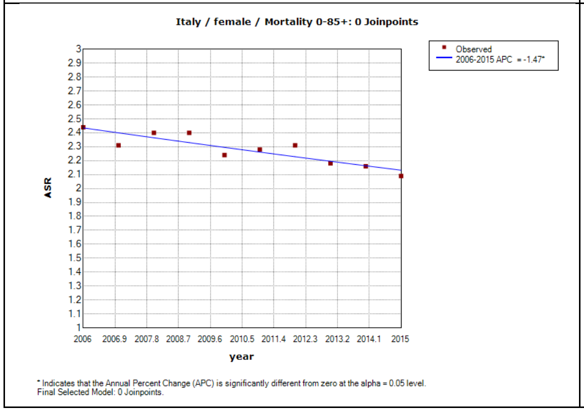

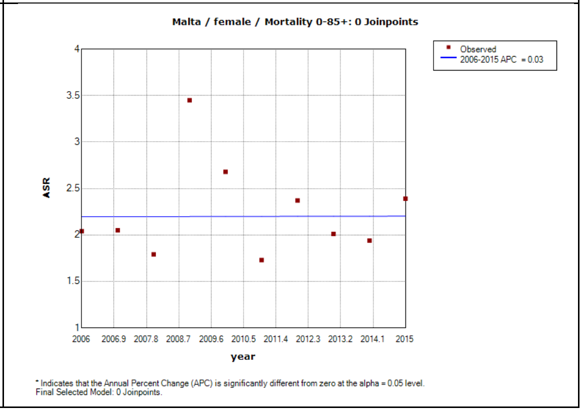

Portugal / female / Mortality 0-85+: 0 Joinpoints

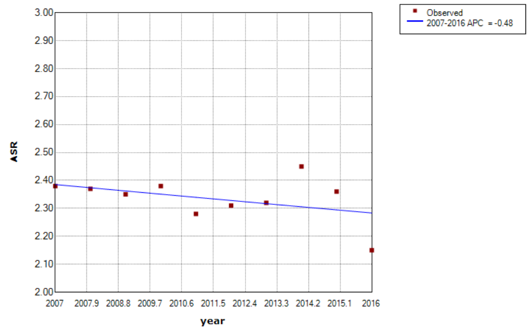

* Indicates that the Annual Percent Change (APC) is significantly different from zero at the alpha = 0.05 level.
Final Selected Model: 0 Joinpoints.

Slovenia / female / Mortality 0-85+: 0 Joinpoints

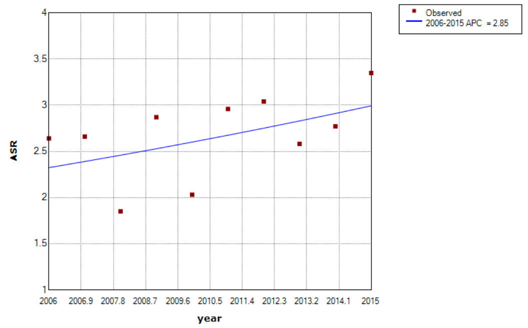

* Indicates that the Annual Percent Change (APC) is significantly different from zero at the alpha = 0.05 level.
Final Selected Model: 0 Joinpoints.

Spain / female / Mortality 0-85+: 0 Joinpoints

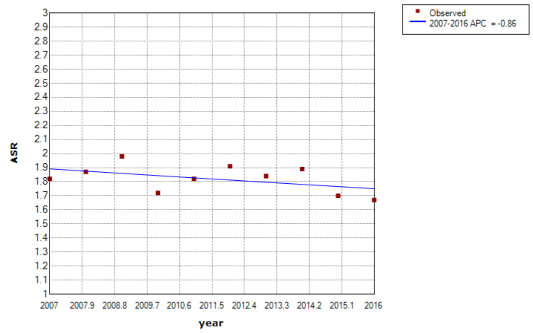

* Indicates that the Annual Percent Change (APC) is significantly different from zero at the alpha = 0.05 level.
Final Selected Model: 0 Joinpoints.

Eastern Europe

Bulgaria / female / Mortality 0-85+: 0 Joinpoints

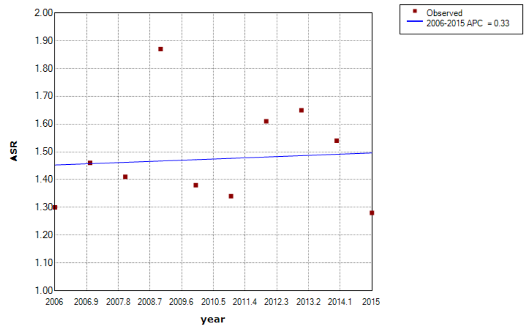

* Indicates that the Annual Percent Change (APC) is significantly different from zero at the alpha = 0.05 level.
Final Selected Model: 0 Joinpoints.

Czech Republic / female / Mortality 0-85+: 0 Joinpoints

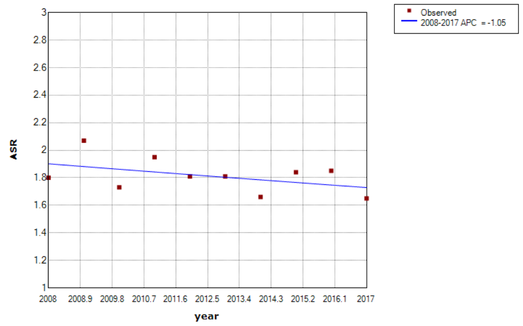

* Indicates that the Annual Percent Change (APC) is significantly different from zero at the alpha = 0.05 level.
Final Selected Model: 0 Joinpoints.

Poland / female / Mortality 0-85+: 0 Joinpoints

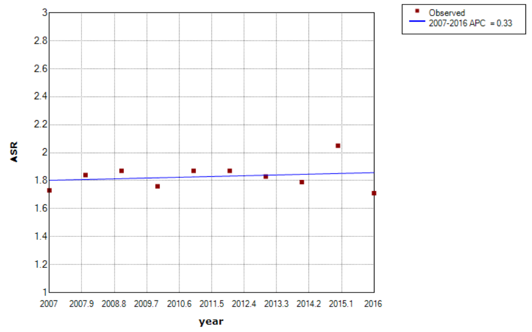

* Indicates that the Annual Percent Change (APC) is significantly different from zero at the alpha = 0.05 level.
Final Selected Model: 0 Joinpoints.

Russian Federation / female / Mortality 0-85+: 0 Joinpoints

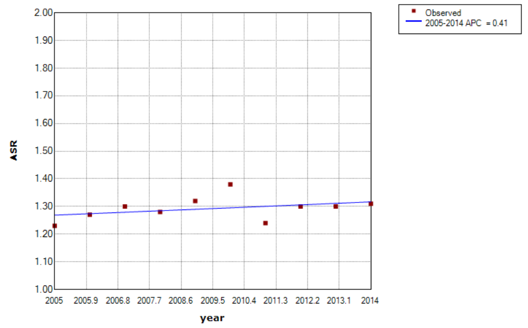

* Indicates that the Annual Percent Change (APC) is significantly different from zero at the alpha = 0.05 level.
Final Selected Model: 0 Joinpoints.

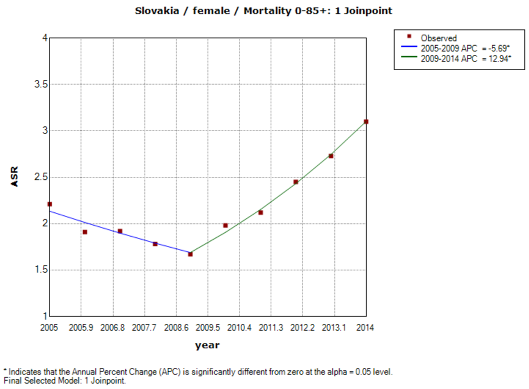
Slovakia / female / Mortality 0-85+: 1 Joinpoint
Observed
2005-2009 APC = -5.69*
2009-2014 APC = 12.94*
ASR
4
3.5
3
2.5
2
1.5
1
2005
2005.9
2006.8
2007.7
2008.6
2009.5
2010.4
2011.3
2012.2
2013.1
2014
year
* Indicates that the Annual Percent Change (APC) is significantly different from zero at the alpha = 0.05 level.
Final Selected Model: 1 Joinpoint.

Supplementary Figure 3. AAPC of incidence of non-hodgkin lymphoma aged 50 years old or above

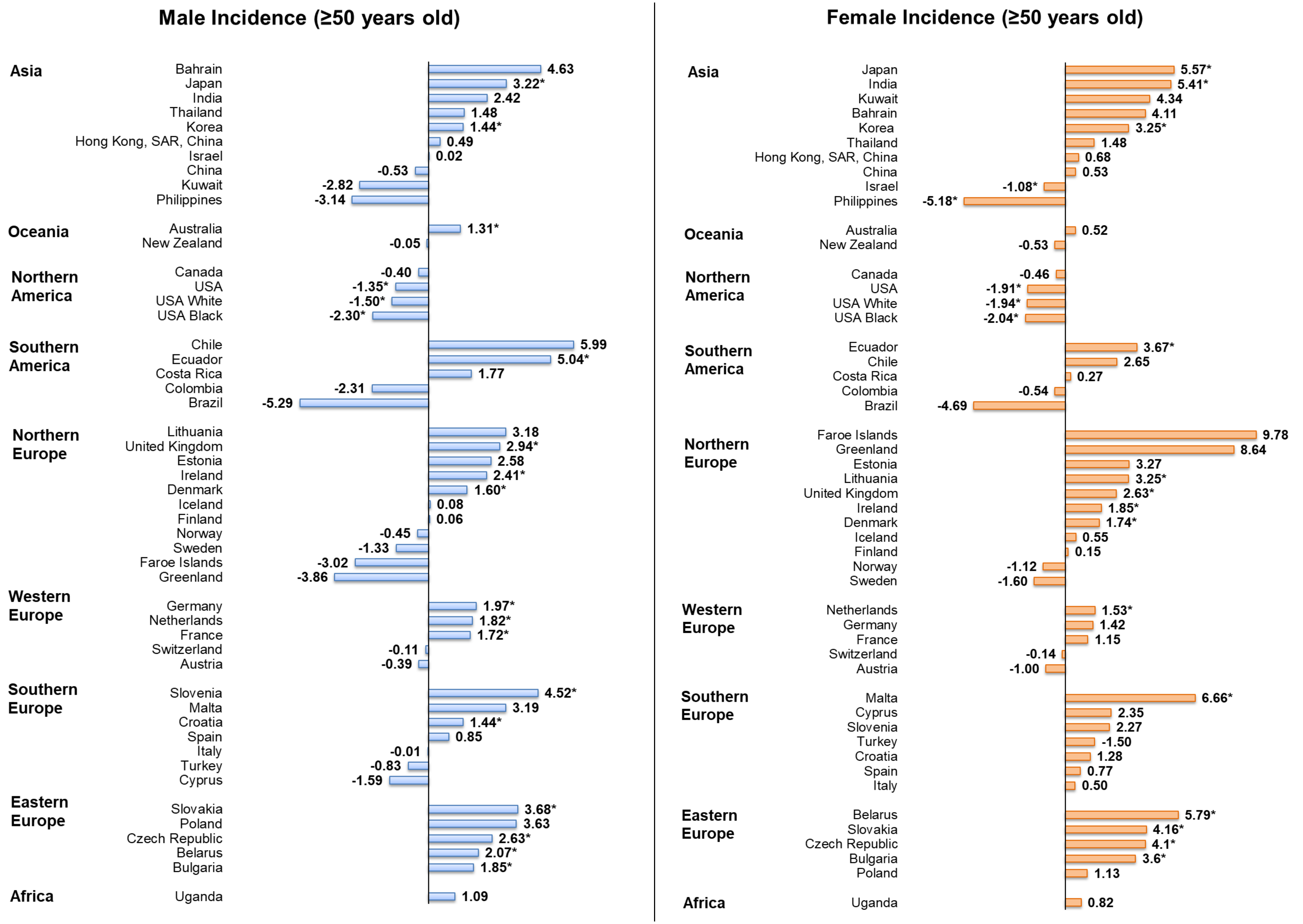

AAPC, annual percentage change; **p* values less than 0.05.

Supplementary Figure 4. AAPC of incidence of non-hodgkin lymphoma aged < 50 years old

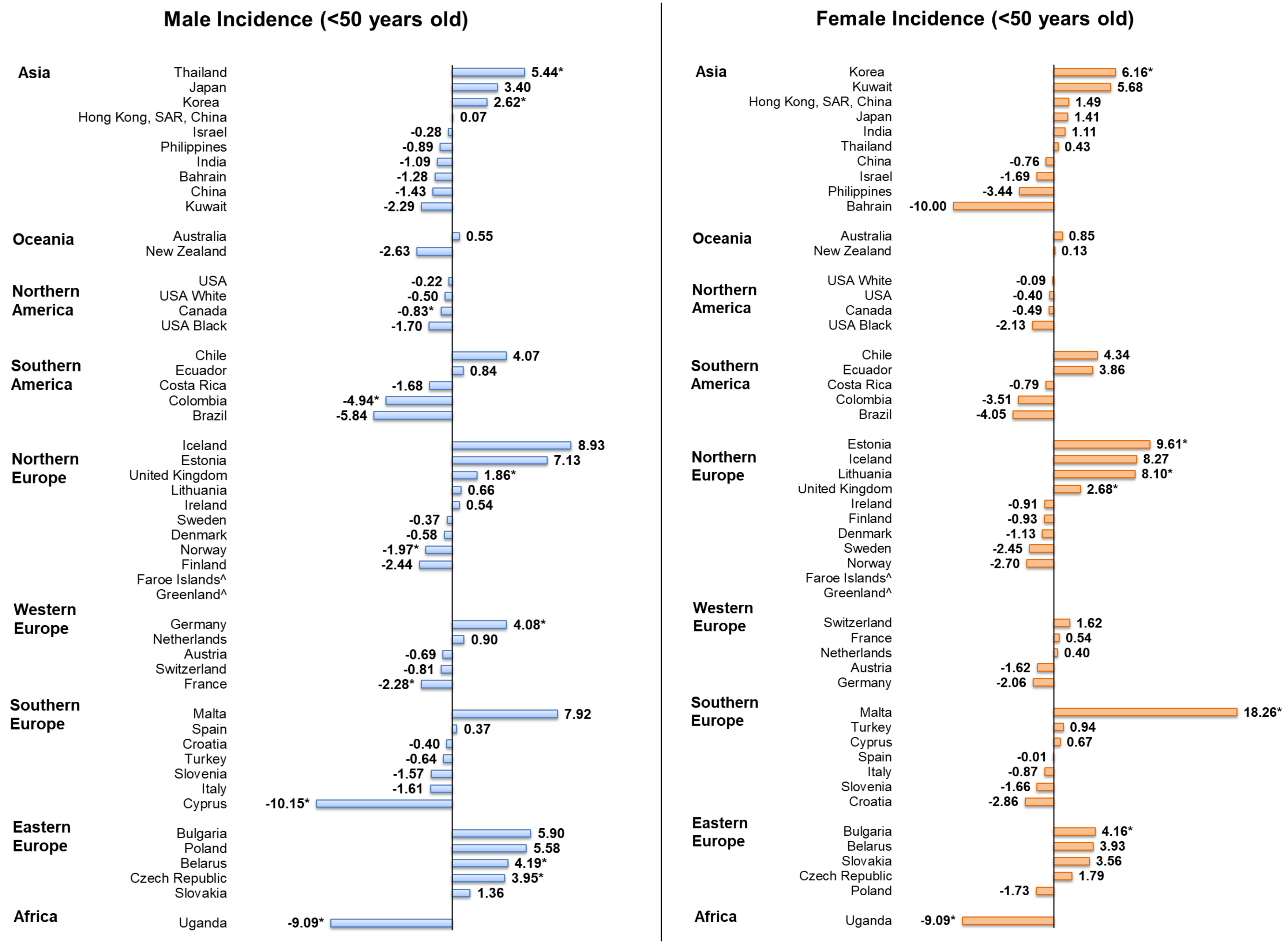

AAPC, annual percentage change; *p values less than 0.05.

Supplementary Figure 5. AAPC of incidence of non-hodgkin lymphoma aged <40 years old

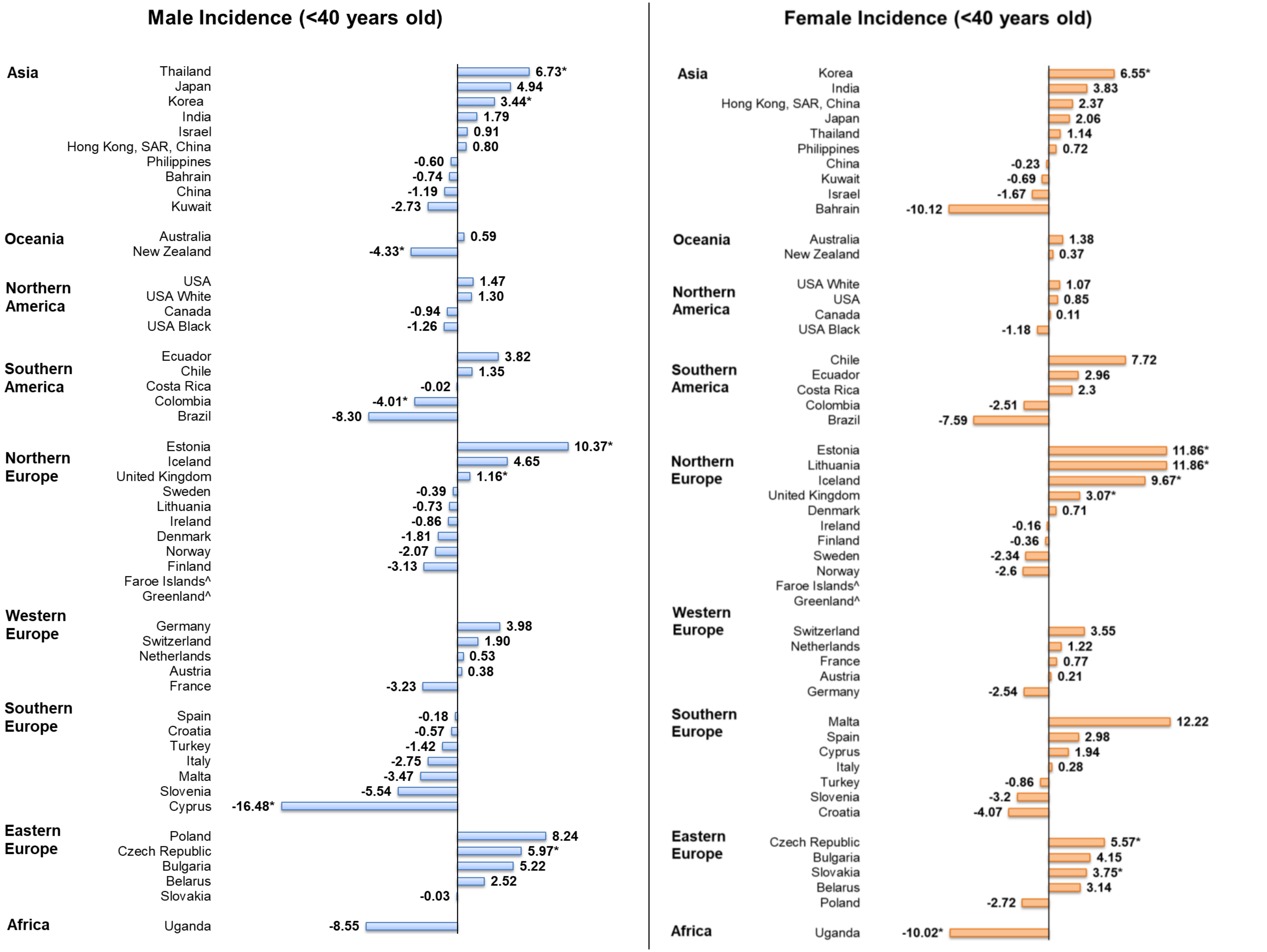

AAPC, annual percentage change; **p* values less than 0.05.

Supplementary Table 1 Data source for the trend analysis.

	**Incidence**	**Mortality**
Austria	CI5 (1998-2012)	WHO (1980-2016)
Australia	CI5 (1993-2012)	WHO (1979-2015)
Bahrain	CI5 (1998-2012)	n/a
Belgium	n/a	WHO (1979-2015)
Brazil	CI5 (1993-2012)[1]	WHO (1979-2015)
Canada	CI5 (1983-2012)[2]	WHO (1979-2013)
Chile	CI5 (1998-2012)	WHO (1997-2016)
China	CI5 (1998-2012)[3]	n/a
Colombia	CI5 (1983-2012)[4]	WHO (1984-2015)
Costa Rica	CI5 (1982-2011)	WHO (1980-2014)
Croatia	CI5 (1988-2012)	WHO (1985-2016)
Czech Republic	CI5 (1983-2012)	WHO (1986-2016)
Denmark	CI5 (1953-2012)	WHO (1994-2015)
Ecuador	CI5 (1985-2012)[5]	WHO (1979-2015)
Estonia	CI5 (1983-2012)	WHO (1981-2015)
Finland	NORDCAN (1953-2015)	NORDCAN (1953-2019)
France	CI5 (1998-2012)[6]	WHO (1979-2014)
Germany	CI5 (1998-2012)[7]	WHO (1990-2016)
Hong Kong SAR, China	CI5 (1983-2012)	WHO (1980-2016)
Iceland	CI5 (1958-2012)	WHO (1981-2016)
India	CI5(1983-2012)[8]	n/a

Ireland	CI5 (1994-2012)	WHO (1979-2014)
Israel	CI5 (1963-2012)[9]	WHO (1979-2015)
Italy	CI5 (1998-2010)[10]	WHO (1979-2015)
Japan	CI5 (1998-2010)[11]	WHO (1979-2015)
Korea	CI5 (1999-2012)	WHO (1985-2016)
Kuwait	CI5 (1998-2012)	n/a
Latvia	n/a	WHO (1980-2015)
Lithuania	CI5 (1988-2012)	WHO (1981-2016)
Malta	CI5 (1993-2012)	WHO (1979-2015)
Netherlands	CI5 (1989-2012)	WHO (1979-2016)
New Zealand	CI5 (1983-2012)	WHO (1979-2013)
Norway	CI5 (1953-2012)	WHO (1986-2015)
Philippines	CI5 (1983-2012)[12]	WHO (1992-2011)
Poland	CI5 (1998-2012)[13]	WHO (1980-2015)
Portugal	n/a	WHO (1980-2014)
Russian Federation	n/a	WHO (1980-2015)
Singapore	n/a	WHO (1979-2015)
Slovakia	CI5 (1971-2010)	WHO (1992-2014)
Slovenia	CI5 (1983-2012)	WHO (1985-2015)
Spain	CI5 (1993-2010)[14]	WHO (1980-2015)
Sweden	NORDCAN (1960-2015)	NORDCAN (1952-2019)
Switzerland	CI5 (1998-2012)[15]	WHO (1995-2015)
Thailand	CI5 (1993-2012)[16]	WHO (1979-2016)
Uganda	CI5 (1993-2012)	n/a

United Kingdom	CI5 (1994-2012)[17]	WHO (1979-2015)
USA	SEER (1975-2015)	SEER (1975-2019)

n/a" not available; CI5: Cancer Incidence in Five Continents V; NORDCAN: Nordic Cancer Registries' SEER: USA: National Institutes of Health (NIH); WHO: World Health Organization

1. Brazil, Goiania
2. Canada (excl. Nunavut, Quebec and Yukon)
3. China (5 registries)
4. Colombia, Cali
5. Ecuador, Quito
6. France (9 registries)
7. Germany (2 registries)
8. India, Chennai
9. Israel: Jews
10. Italy (8 registries)
11. Japan (4 registries)
12. Philippines, Manila
13. Poland, Kielce
14. Spain (9 registries)
15. Switzerland (6 registries)
16. Thailand (4 registries)
17. UK, England

Reference:

1. SEER: http://seer.cancer.gov/data/seerstat/
2. NORDCAN: http://www-dep.iarc.fr/NORDCAN/english/frame.asp
3. WHO: http://apps.who.int/healthinfo/statistics/mortality/whodpms/
4. CI5: http://ci5.iarc.fr/CI5plus/Pages/table1_sel.aspx
